# The CP2K Program Package Made Simple

**DOI:** 10.1021/acs.jpcb.5c05851

**Published:** 2026-01-15

**Authors:** Marcella Iannuzzi, Jan Wilhelm, Frederick Stein, Augustin Bussy, Hossam Elgabarty, Dorothea Golze, Anna-Sophia Hehn, Maximilian Graml, Stepan Marek, Beliz Sertcan Gökmen, Christoph Schran, Harald Forbert, Rustam Z. Khaliullin, Anton Kozhevnikov, Mathieu Taillefumier, Rocco Meli, Vladimir V. Rybkin, Martin Brehm, Robert Schade, Ole Schütt, Johann V. Pototschnig, Hossein Mirhosseini, Andreas Knüpfer, Dominik Marx, Matthias Krack, Jürg Hutter, Thomas D. Kühne

**Affiliations:** † Department of Chemistry, 27217 University of Zurich, CH-8057 Zürich, Switzerland; ‡ Regensburg Center for Ultrafast Nanoscopy (RUN) and Institute of Theoretical Physics, 9147 University of Regensburg, D-93053 Regensburg, Germany; § Center for Advanced Systems Understanding (CASUS), Helmholtz Zentrum Dresden-Rossendorf, D-02826 Görlitz, Germany; ∥ Swiss National Supercomputing Centre (CSCS), 28489 ETH Zurich, CH-6900 Lugano, Switzerland; ⊥ Department of Chemistry, Paderborn University, D-33098 Paderborn, Germany; # Department of Chemistry and Food Chemistry, Technische Universität Dresden, D-01069 Dresden, Germany; ¶ Department of Chemistry, 9179 Christian-Albrechts-University Kiel, D-24118 Kiel, Germany; ∇ Cavendish Laboratory, Department of Physics, University of Cambridge, CB3 0HE Cambridge, U.K.; ○ Center for Solvation Science ZEMOS, 9142 Ruhr-Universität Bochum, D-44801 Bochum, Germany; ⧫ Department of Chemistry, McGill University, Montreal QC H3A 0B8, Canada; †† HQS Quantum Simulations GmbH, D-76131 Karlsruhe, Germany; ‡‡ Paderborn Center for Parallel Computing (PC2), Paderborn University, D-33098 Paderborn, Germany; §§ CP2K Foundation, CH-8057 Zürich, Switzerland; ∥∥ Lehrstuhl für Theoretische Chemie, Ruhr-Universität Bochum, D-44780 Bochum, Germany; ⊥⊥ PSI Center for Scientific Computing, Theory and Data, 28498 Paul Scherrer Institute, CH-5232 Villigen PSI, Switzerland; ## Institute of Artificial Intelligence, Technische Universität Dresden, D-01187 Dresden, Germany; ¶¶ Cluster of Excellence “Physics of Life”, Technische Universität Dresden, D-01307 Dresden, Germany

## Abstract

CP2K is a versatile open-source software package for simulations across a wide range of atomistic systems, from isolated molecules in the gas phase to low-dimensional functional materials and interfaces, as well as highly symmetric crystalline solids, disordered amorphous glasses, and weakly interacting soft-matter systems in the liquid state and in solution. This review highlights CP2K’s capabilities for computing both static and dynamical properties using quantum-mechanical and classical simulation methods. In contrast to the accompanying theory and code paper [*J. Chem. Phys. 152*, 194103 (**2020**)], the focus here is on the practical usage and applications of CP2K, with underlying theoretical concepts introduced only as needed.

## Graphical abstract

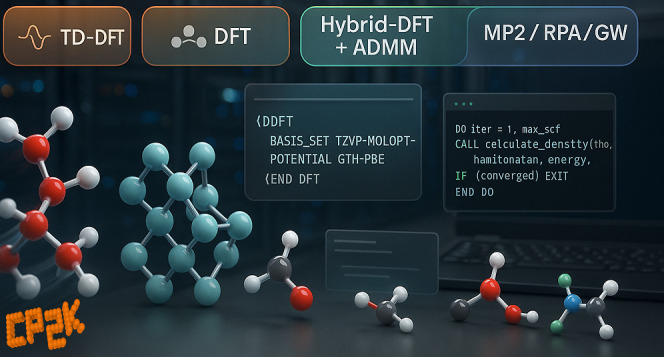

## 1 Introduction

The aim of CP2K is to predict average properties, such as those that arise in statistical mechanics and thermodynamics, of any substance made of interacting electrons and nuclei from first-principles. Although with the advent of quantum mechanics this became possible by solving the nonrelativistic many-body Schrödinger equation, early on Dirac recognized that the exact application of these laws leads to equations much too complicated to be soluble.^1^ Yet, assuming the so-called Born–Oppenheimer approximation, which is underlying most of the methods within CP2K, the full many-body Schrödinger equation can be separated into its electronic and nuclear counterparts.

Consequently, the general structure of CP2K entails a wide variety of different classical and quantum mechanical energy and force methods that can be arbitrarily combined with geometry optimization and transition state search techniques,^2,3^ as well as (rare event) sampling approaches, such as Monte Carlo (MC), meta- and molecular dynamics (MD),^4–6^ to name just a few. The latter approaches dealing with the nuclear motion are specified in the &MOTION section, whereas the former force methods, which are due to the electronic structure of matter, are detailed in the &FORCE_EVAL section. However, since we have elected to limit ourselves to computational schemes that make CP2K unique, the techniques of the &MOTION section, which are well described in various textbooks,^7,8^ will be mostly neglected. The same also applies to ab initio MD (AIMD),^9,10^ in particular the second-generation Car–Parrinello method,^11,12^ which has been extensively covered elsewhere.^13–15^

Due to the user-focused nature of the present review, the paper is organized by the property to be calculated instead of the computational methods used, which will be introduced in the relevant sections where necessary. Hence, first how to compute the total energy and nuclear forces of a system using density functional theory (DFT)^16^ and quantum chemical models^17^ is presented in Section 2. Thereafter, the calculation of the electronic band structure using the pseudopotential plane wave (PP-PW),^18^ projector augmented-wave (PAW),^19^ full-potential linearized augmented plane wave (FP-LAPW),^20^ as well as single-particle Green’s function and the screened Coulomb interaction (*GW*) methods,^21^ is detailed in Section 3. Embedding schemes such as implicit solvation and mixed classical-quantum mechanical approaches, as well as purely quantum mechanical embedding theories, are the content of Section 4. Nuclear magnetic resonance (NMR) and electron paramagnetic resonance (EPR) spectroscopies are described in Section 5, whereas optical spectroscopy methods such as linear-response time-dependent DFT (LR-TDDFT) and the Bethe–Salpeter equation (BSE) are the subject matter of Section 6. Section 7 is dedicated to excited state dynamics in terms of real-time TDDFT (RT-TDDFT), Ehrenfest dynamics and real-time BSE (RT-BSE), whereas X-ray absorption (XAS) and X-ray emission (XES) spectroscopies are described in Section 8. Energy decomposition analysis (EDA) based on absolutely localized molecular orbitals (ALMO) for condensed phase systems is the focus of Section 9. Finite temperature effects using machine learning potentials (MLPs) including nuclear quantum effects (NQEs) by means of path-integral MD (PIMD) simulations are presented in Section 10, before concluding the paper on the extended topic of vibrational spectroscopy.

## 2 Total Energy and Force Methods

Even though total energies and atomic forces are rarely relevant observables for themselves, they are the basis for the theoretical computation of all properties considered here. In the spirit of CP2K, to simulate all possible kinds of matter from isolated molecules to the extended condensed phase, all energy and force methods can not only be employed in vacuo (0D), but also in the presence of arbitrary periodic boundary conditions (PBC), i.e. 1D (chains and nanotubes), 2D (surfaces and interfaces) and 3D (solids and liquids). Moreover, to explicitly include finite temperature and pressure effects by means of dynamical sampling methods, analytical gradients are generally available for all classical and QM electronic structure methods, even in combination with PBCs.

This is made possible by the Quickstep module,^14,22,23^ which is based on the Gaussian and plane wave (GPW) method and its all-electron counterpart denoted as the Gaussian and augmented plane wave (GAPW) approach,^24,25^ and contains all electronic structure methods described in Section 2, i.e. in particular DFT and Hartree–Fock (HF),^26^ but also hybrid-DFT and post-HF schemes such as second-order Møller–Plesset perturbation theory (MP2)^27^ and the random phase approximation (RPA).^28,29^

### 2.1 Density Functional Theory

The unique aspect of the GPW and GAPW methods is their usage of a mixed Gaussian and an auxiliary plane wave (PW) basis set, thereby unifying the computational efficiency of a compact localized basis set with the simplicity of PWs for periodic electronic structure calculations. Within CP2K/Quickstep, the Kohn–Sham (KS) orbitals ϕ_*i*_(**r**) are represented by atom-centered Gaussian functions, whereas the electron density is expanded in PWs on a regular grid. This is to say that the molecular orbitals (MO) are represented by the common linear combination of atomic orbitals (LCAO)

1 $$\phi_{i}\left(r\right)=\sum_{j}c_{ji}\varphi_{j}\left(r\right)$$

where *c*_*ji*_ are the so-called orbital coefficients, and the atomic orbitals (AO)

2 $$\varphi_{j}\left(r\right)=\sum_{k}d_{kj}g_{k}\left(r\right)$$

are themselves expanded in terms of primitive Gaussian functions *g*_*k*_(**r**) and their corresponding contraction coefficients *d*_*kj*_.

The electron density ρ­(**r**) can either be written as

3 $$\rho \left(r\right)=\sum_{\mu \nu }P^{\mu \nu }\varphi_{\mu }\left(r\right)\varphi_{\nu }\left(r\right)$$

or alternatively in an auxiliary PW basis as

4 $$\mathrm{\rho ̃}\left(r\right)=\frac{1}{\Omega }\sum_{G}\rho \left(G\right)e^{iG\cdot r}$$

where Ω is the volume of the unit cell and the expansion coefficients ρ­(**G**) are such that ρ­(**r**) = ρ̃(**r**).

Exploiting the efficiency of the fast Fourier transform (FFT), the KS matrix construction including the periodic long-range electrostatics can be performed in O(Mlog⁡M) with *M* being the number of basis functions, via

5 $$\begin{array}{ccc} P\to \rho \left(r\right) & \overset{FFT}{\to }\rho \left(G\right)\to & 4\pi \frac{\rho \left(G\right)}{G^{2}}\\ & & ∥\\ V\leftarrow V_{H}\left(r\right) & \overset{{FFT}^{-1}}{\leftarrow } & V_{H}\left(G\right) \end{array}$$

*V*_H_(**G**) is the Hartree potential that is the solution of the Poisson equation ∇^2^ *V*_H_(**G**) = −4πρ­(**r**). By substituting ρ­(**r**) with the total charge density, which consists of the electron density plus Gaussian-smeared nuclear charges, all long-range electrostatic energy terms can be coalesced into a single Hartree-like expression that has to be augmented by compensating overlap and self-energy terms generated by these Gaussian distributions. Similarly, the exchange and correlation (XC) functional is evaluated on the very same uniform density grid as the Hartree energy, so that starting from ρ­(**r**), the combined potential for the long-range electrostatic and XC energies can be computed using FFT techniques.^14,24,25^

#### 2.1.1 Basis Set Convergence Using the Quickstep Method

This is why in a typical Quickstep calculation, which is activated via &FORCE_EVAL%METHOD QUICKSTEP, the basis has to be defined in two sections. On the one hand, the localized Gaussian basis set is defined in the &FORCE_EVAL%SUBSYS%KIND section and, on the other hand, the PW density cutoff in the &FORCE_EVAL%DFT%MGRID section. It is important to recognize, however, that the latter is a density cutoff and therefore 4 times higher than a comparable energy cutoff typically specified in PW codes, which is a manifestation of Nyquist’s sampling theorem together with the fact that the electron density is the squared modulus of the wave function (WF). Also, contrary to conventional PW codes, the complete basis set limit cannot be reached just by increasing the density cutoff of the auxiliary PW basis only. Instead, by increasing CUTOFF the limit of the finite Gaussian set is approached, which is why the convergence of the auxiliary PW basis depends on the underlying Gaussian basis and both have to be balanced and increased concurrently to yield the complete basis set limit.

An important aspect of the Quickstep method is the usage of real-space integration grids to represent the electron density and product Gaussian functions in conjunction with multigrid techniques, so that wide and smooth Gaussian functions are mapped onto a coarser grid than narrow and sharp Gaussians. However, the electron density is always mapped onto the finest grid. Hence, choosing a fine enough integration grid is crucial for any calculation to obtain highly accurate results as efficiently as possible. All settings related to multigrids are controlled within the &MGRID subsection. The number of multigrid levels is defined by NGRIDS, with 5 typically being a suitable value for most applications. As alluded to above, the keyword CUTOFF defines the PW density cutoff (in Rydbergs, Ry), the corresponding cutoffs for subsequent grid levels (from finer to coarser) are defined via

6 $$E_{cut}^{i}=\frac{E_{cut}^{1}}{\alpha^{i-1}}$$

where *i* is the corresponding multigrid level and α the progression factor controlled by PROGRESSION_FACTOR. Hence, the higher the value of CUTOFF, the finer the grids of all multigrid levels. To determine which product Gaussians are mapped onto which multigrid level, REL_CUTOFF defines the PW cutoff of a reference grid covered by a Gaussian with unit standard deviation, i.e. exp­(|**r**|^2^). Therewith, a Gaussian is mapped onto the coarsest level of the multigrid, on which the function will require a number of grid points greater than or equal to the number of grid points exp­(|**r**|^2^) will cover on a reference grid defined by REL_CUTOFF. In this way, CP2K tries to map each Gaussian onto a grid such that the number of grid points covered by the Gaussian, no matter how wide or narrow, is roughly the same. Hence, the two most important keywords affecting real-space integration grids and, as such, the convergence of the auxiliary PW basis are CUTOFF and REL_CUTOFF, respectively. A prototypical CP2K input section, using the GPW method, reads as follows:
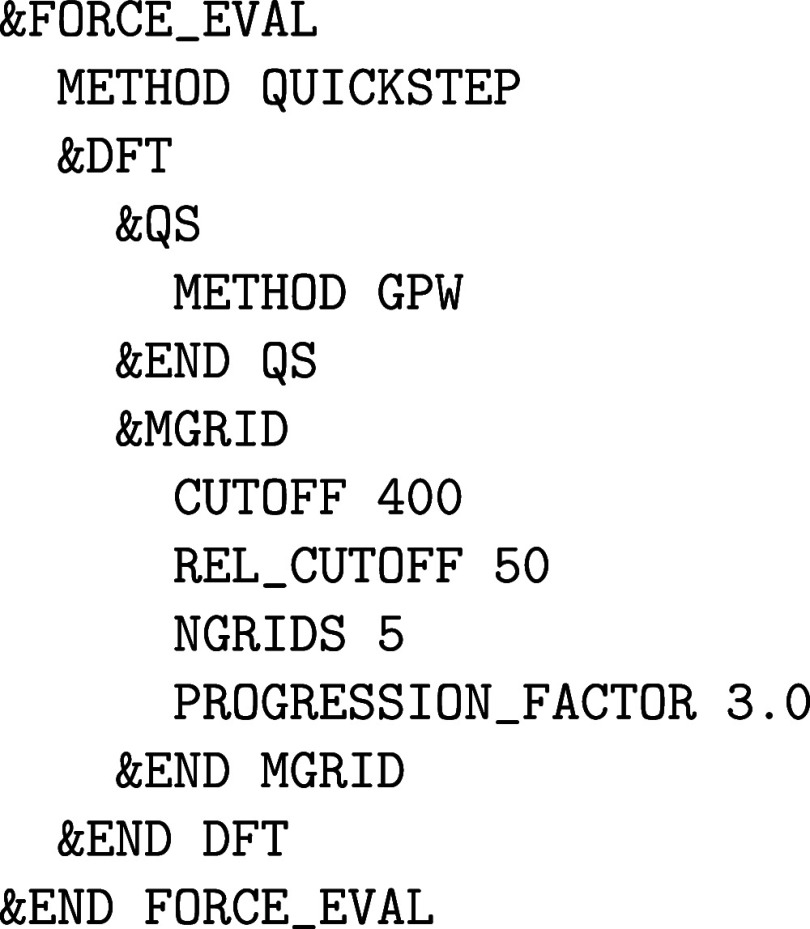


#### 2.1.2 Pseudopotentials and Basis Sets

Besides the auxiliary PW basis, the primary Gaussian basis set and optionally also the employed pseudopotentials (PP) are essential for the accuracy of any Quickstep calculation. For that purpose, in recent years the so-called “UZH protocol” has been developed that contains both molecularly optimized Gaussian basis sets for all-electron and PP calculations,^30^ as well as corresponding separable dual-space Goedecker-Teter-Hutter (GTH) PPs that are highly transferable and norm-conserving.^31–33^

In addition to accelerating the calculation of the electronic structure by reducing the number of electrons and the basis set size, the usage of PPs also allows for the inclusion of relativistic effects. The separable dual-space GTH PPs of the “UZH protocol” are fully nonlocal with Gaussian-type projectors because of their simplicity and efficiency due to the usage of analytic integrals and FFTs. The local part, however, is represented by a Gaussian form in real-space, which can be well combined with Gaussian basis sets. Hence, the fully analytical form of the GTH PPs requires only a small set of parameters for each element that is optimized with respect to an atomic all-electron WF of scalar relativistic DFT reference calculations,^34^ as obtained by the CP2K ATOM code. The “UZH protocol” contains GTH PPs for the periodic table up to Rn for generalized gradient approximation (GGA) (PBE^35^), meta-GGA (SCAN^36^), and hybrid functionals (PBE0 ^37^). Special purpose GTH PPs for lanthanides and actinides,^38–41^ as well as including nonlinear core corrections and spin–orbit coupling parameters, are also available at https://github.com/cp2k/cp2k/tree/master/data.^42^ In addition, via the libgrpp library to evaluate molecular integrals over Gaussian functions,^43^ most effective core potentials (ECP) provided by the EMSL Basis Set Exchange can be employed.^44^

The basic idea of the molecular-optimized MOLOPT basis set is to use generally contracted Gaussian basis sets, including diffuse primitives, fully optimized on molecular calculations.^30^ The absence of lone diffuse functions ensures a low condition number of the overlap matrix, leading to better self-consistent field (SCF) convergence and a sparser density matrix, whereas the inclusion of diffuse primitive functions entails a particularly small basis set superposition error. By molecularly optimizing the basis functions, small but accurate basis sets are obtained, suitable for large-scale isolated gas and condensed phase calculations. As with PPs, in addition to the standard general purpose “UZH protocol”, more specialized MOLOPT basis sets for lanthanides and actinides,^38–41^ solids,^45–49^ and for the later described auxiliary density matrix method (ADMM),^50^ *GW*,^51,52^ and resolution of the identity approaches (RI),^53,54^ such as RI-MP2 and RI-RPA, are also available in the same repository. In addition, the “UZH protocol” also contains all-electron MOLOPT basis sets, although all other basis sets that can be downloaded at http://basissetexchange.org in CP2K format are also supported.^44^

A typical section for gold as an example looks as follows:
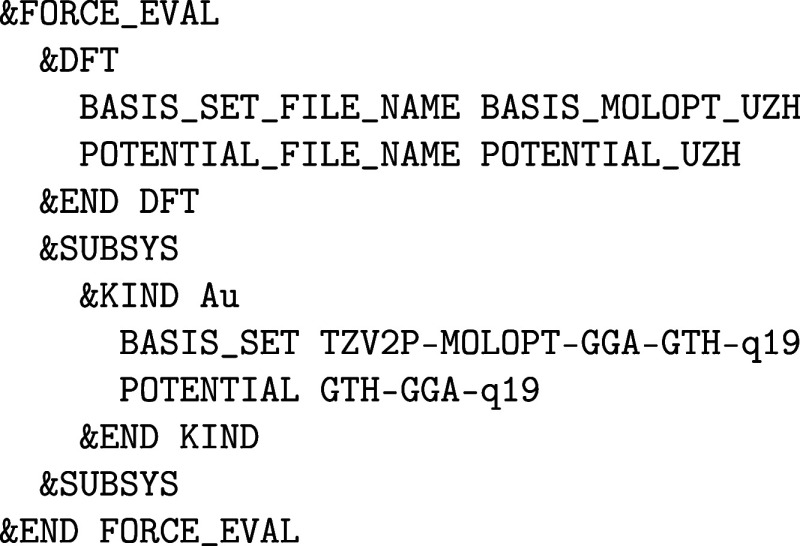


The accuracy of the “UZH protocol” can best be assessed by means of all-electron FP-LAPW calculations at the complete basis set limit using the CP2K-integrated PW code SIRIUS, which is described in Section 3.1.

The deviation between SIRIUS and Quickstep employing a TZV2P MOLOPT basis set and corresponding GTH PPs is shown in Figure 1 for a large number of different unary crystal structures and quantified in terms of the previously suggested metric

7 $$\varepsilon \left(a,b\right)=\sqrt{\frac{\sum_{i}{\left[E_{a}\left(V_{i}\right)-E_{b}\left(V_{i}\right)\right]}^{2}}{\sqrt{\sum_{i}{\left[E_{a}\left(V_{i}\right)-\left\langle E_{a}\right\rangle \right]}^{2}\sum_{i}{\left[E_{b}\left(V_{i}\right)-\left\langle E_{b}\right\rangle \right]}^{2}}}}$$

where the index *i* runs over multiple calculations of *E*(*V*) within a given space group.^55^ Since this quantity is insensitive to the magnitude of the bulk modulus, it provides a uniform metric across a large variety of structural and chemical environments.

**Figure 1:**
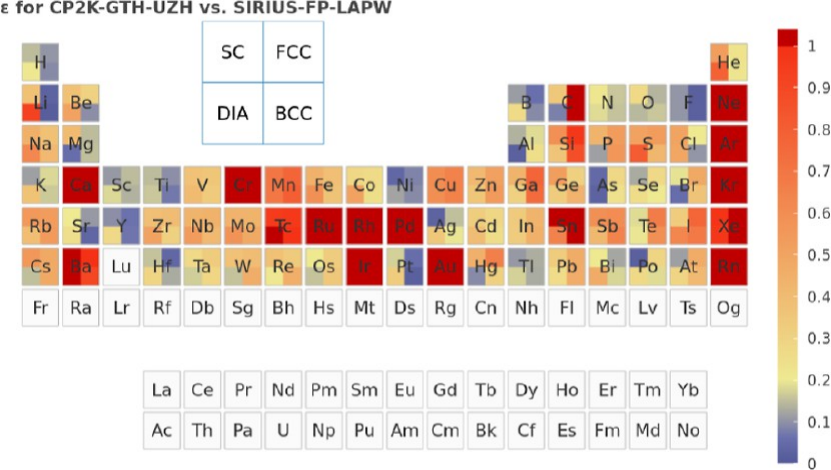
Values of the comparison metric ε for CP2K/Quickstep using the “UZH protocol” with respect to all-electron FP-LAPW calculations using the CP2K/SIRIUS code. For each element up to Rn four monoelemental cubic crystals were considered.^55^

#### 2.1.3 All-Electron Calculations Using the GAPW Method

All-electron calculations with CP2K/Quickstep necessitate the use of the GAPW method,^25,56^ because a description of the core electrons with PWs becomes rapidly computationally prohibitive. Likewise, small-core and sometimes even medium-core PPs can only be used with GAPW efficiently. There are various possibilities to devise a PP for heavy elements such as uranium with 92 electrons:

- large-core PP ([86 core] 6 valence electrons)
- [Rn] 7s^2^ 5f^3^ 6d^1^
- medium-core PP ([78 core] 14 valence electrons)
- [Xe 4f^14^ 5d^10^] 6s^2^ 6p^6^ 7s^2^ 5f^3^ 6d^1^
- small-core PP ([60 core] 32 valence electrons)
- [Kr 4d^10^ 4f^14^] 5s^2^ 5p^6^ 5d^10^ 6s^2^ 6p^6^ 7s^2^ 5f^3^ 6d^1^

The large-core PP for uranium is known to be inaccurate due to the non-negligible overlap of the outer valence orbitals with the semicore 6s and 6p orbitals, which does not allow keeping these semicore orbitals as frozen. Medium-core PPs for uranium are mostly used by PW codes^57^ and with GPW,^58^ because these PPs are sufficiently accurate and the semicore 6s and 6p orbitals are not too hard for an efficient expansion by PWs. However, this is not possible for small-core uranium PPs that require GAPW.

Furthermore, the calculation of several properties, especially spectroscopic properties involving core electrons, requires the explicit inclusion of the core electrons.^59,60^ The GAPW method must be activated explicitly in the &DFT%QS section of the input, i.e.
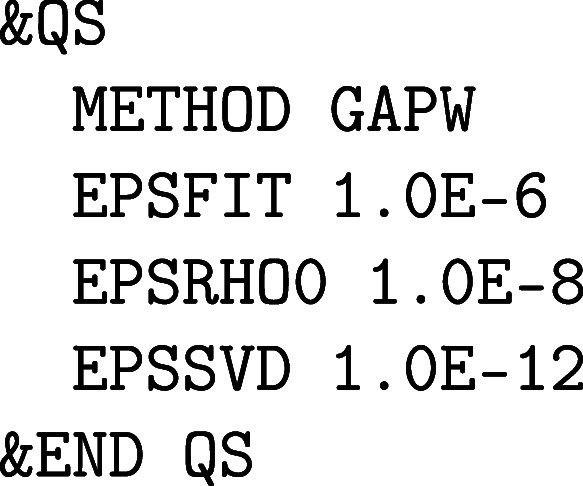


The GAPW specific parameters EPSFIT, EPSRHO0 and EPSSVD are optional and their default values are 10^–4^, 10^–6^ and 10^–8^, respectively. These parameters are used to control the accuracy of a GAPW calculation as indicated by the electron count printed after SCF convergence has been achieved. EPSFIT controls the split of Gaussian basis sets into a hard and a soft basis set for each atomic kind in the system. Smaller values for EPSFIT promote the inclusion of larger Gaussian exponents in the soft basis. Only the part of the electron density defined by the soft basis set is expanded in PWs, whereas the hard basis set describes the hard (frozen) part of the electron density close to the nuclei. A GAPW setup seamlessly turns into a GPW one when all exponents are included in the soft basis sets and all hard basis sets are “empty”. In this case, the default METHOD GPW can be used, since GAPW is not required. Gaussian exponents up to 5 should always be included in the soft basis set. Check the values in the first column of the Gaussian basis set definition or the basis set printout in the CP2K output for PRINT_LEVEL MEDIUM or higher. The electron count should show at least an accuracy of 1.0 × 10^–6^ . For improved accuracy, Gaussian exponents in the range 5–10 can be added to the soft basis by lowering the value of EPSFIT. The inclusion of larger exponents should always be accompanied by an adequate increase of the density CUTOFF in &MGRID. EPSRHO0 and EPSSVD improve the numerical precision of *V*(*ρ*_0_ – *ρ*_0_^soft^) and the singular value decomposition (SVD) of the PAW projector matrix, respectively. In this way, an electron count of 1.0 × 10^–8^ or even less can be achieved. Gaussian exponents up to about 20 can still be expanded in PWs using large CUTOFF values, but the expansion of larger exponents quickly becomes computationally prohibitive or even impossible.

A few atomic kind-specific settings complete a GAPW all-electron setup:
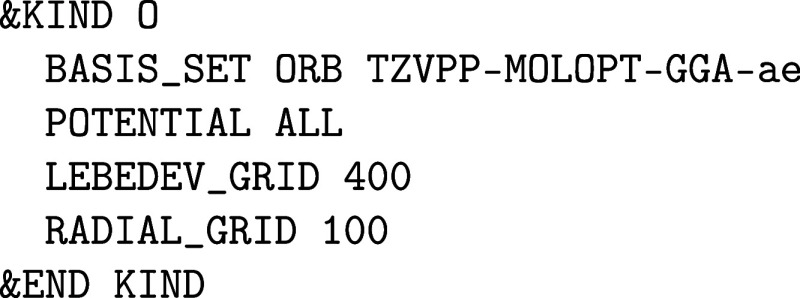


The atomic POTENTIAL must be set to ALL and an all-electron ORBital basis set has to be selected. Note that most of these all-electron basis sets are made for isolated (0D) systems such as molecules or clusters in the gas phase, but there are also a few all-electron basis sets suited for condensed phase calculations with CP2K/Quickstep, as described in Section 2.1.2. For heavier elements, ECPs are employed that require GAPW like small core GTH PPs, since an all-electron description for condensed phase systems with heavy elements quickly becomes computationally intensive. There are usually Gaussian basis sets specifically optimized for such ECPs.

Optionally, the size of the atom-centered spherical Lebedev grids can be increased to improve the accuracy of the GAPW electron count for atomic densities and, therefore, also for total energies and nuclear forces. The number of RADIAL_GRIDS can be increased from the default value 50 to 100 or more, whereas only spherical Lebedev grids with certain sizes exist. The atomic grids are automatically generated in an adaptive manner.^61^ The default value for the maximum LEBEDEV_GRID is 50 and thus is rather small. The following Lebedev grids are implemented in CP2K: 6, 14, 26, 38, 50, 86, 110, 146, 194, 302, 434, 590, 770, 974. The grids close to and far from the nuclei are small because the electron density is rather spherical at these distances, while the selected maximum grid is employed in the valence regions. For instance, LEBEDEV_GRID 400 limits the maximum employed Lebedev grid for an atomic kind to 302.

#### 2.1.4 Wave Function Optimization

Since the Hamiltonian of effective single-particle theories, such as DFT, HF, semiempirical quantum chemistry (SQC) and tight-binding (TB), depends on the electron density, i.e. on their own solution, the corresponding equations have to be solved iteratively using the SCF approach. The ability to converge this SCF cycle and to achieve self-consistency depends on multiple factors such as the band gap and dimensionality of the system, as well as the locality of the employed basis functions.

CP2K/Quickstep provides two general techniques to yield self-consistency: traditional diagonalization and the orbital transformation (OT) method.^62^ The former approach entails the direct diagonalization of the Hamiltonian followed by a mixing strategy, such as the Kerker, Pulay, multisecant, and Broyden techniques.^63–66^ In these damped preconditioned fixed-point iteration schemes, the electron density of the next step is constructed in terms of a linear combination of previous densities, in order to achieve a robust SCF convergence with the fewest number of steps. For hardly converging systems, such as metallic solids for which finite electron temperature smearing and **k**-point sampling may also be required, Broyden mixing is typically the best choice, and the associated settings in the following input snippet are a good starting point:
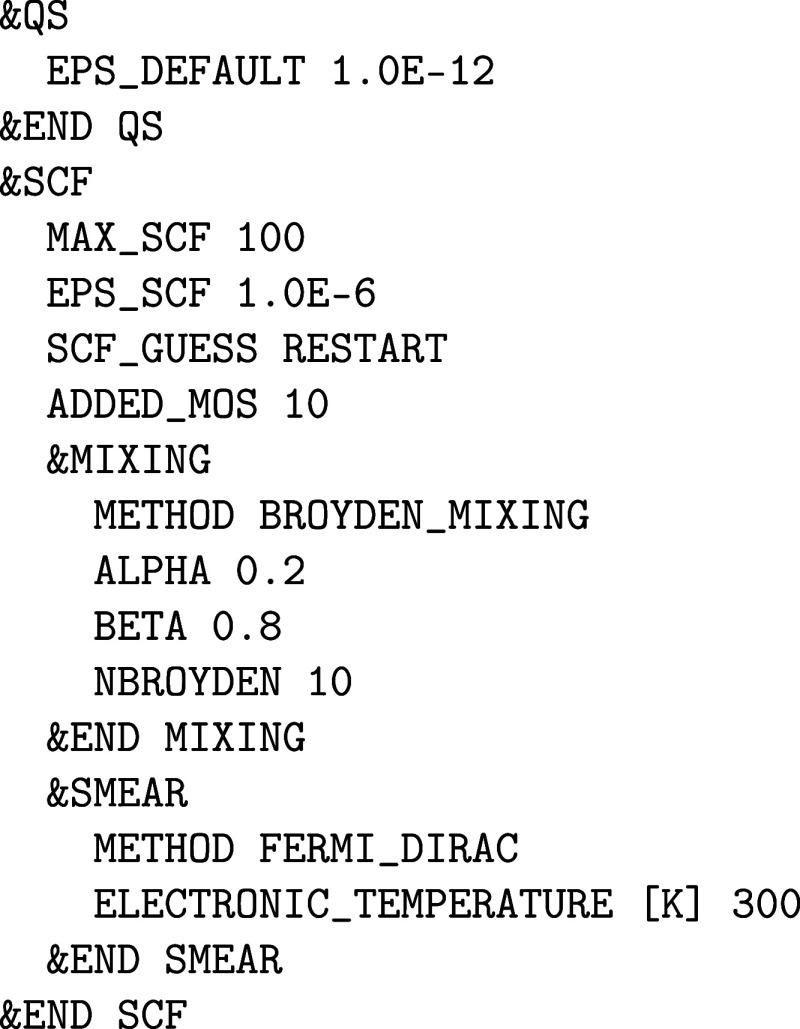


Therein, the keyword EPS_SCF denotes the SCF convergence threshold ∥**P**_*i*_ – **P**_*i*–1_∥_max_, where **P**_*i*_ is the density matrix of the i-th iteration. Most other threshold values of the &DFT%QS section are connected to EPS_DEFAULT, whose value should be approximately that of EPS_SCF squared to ensure a sufficiently high numerical accuracy in order to reach the desired SCF convergence threshold. For metallic systems, finite electron temperature or smearing the density of states, which is specified in the SMEAR section, is often used to improve the convergence with respect to Brillouin zone sampling that is described in Section 2.1.5. Besides the temperature, however, the occupation function must be given, whose natural choice is the physical Fermi–Dirac distribution that would lead to the grand-canonical extension to DFT of Mermin.^67^

However, for gapped systems, a computationally much more efficient way to locate the electronic ground state is by variationally minimizing the total energy with respect to the orbital coefficient matrix **C** subject to the orthonormality constraint **C** ^*T*^ **SC** = **I** since electrons are Fermions. Inspired by the exponential transformation,^68^ the occupied orbitals are parametrized in terms of an auxiliary variable **X**, i.e.

8 C(X)=CUcos(U)+Xsin(U)

where

9 $$U=\sqrt{X^{T}SX}$$

This allows for an unconstrained optimization using the OT method, given that **X** obeys the linear constraint **X** ^*T*^ **SC** = **0**.

In addition to the minimization scheme employed, the choice of a preconditioner is essential. In this regard, the ability to converge the KS equations has to be carefully balanced against the number of necessary minimization steps, which should not be confused with SCF iterations, and the computational effort to construct the preconditioner. Hence, for well converging large-scale systems, preconditioners based on the Cholesky inversion of **H** – **ϵ**_**0**_ **S** (FULL_SINGLE_INVERSE),^62^ or **T** + **ϵ**_**0**_ **S** (FULL_KINETIC),^69^ where **ϵ**_0_ is an estimate of the highest eigenvalue of **C** ^*T*^ **HC** and **T** the kinetic energy matrix, are typically offering the computationally best price/performance ratio. However, contrary to the aforementioned SCF approach, the convergence criterion employing OT is tested in terms of the preconditioned mean gradient deviation, which is why it has to be chosen approximately 1 order of magnitude tighter to achieve a comparable convergence than using direct diagonalization. In case convergence is not foreseeable after approximately 20 OT minimization steps, an additional loop can be introduced via an &OUTER_SCF section, whose purpose is not to conduct an outer-loop optimization, but solely to update the preconditioner. For even more difficult cases, the diagonalization-based FULL_ALL preconditioner should be used, which relies strongly on an estimate of the HOMO – LUMO gap. Since this must not overestimate the true band gap, ENERGY_GAP can hardly be set too low.

Among the various implemented optimization techniques, the direct inversion of the iterative subspace (DIIS) scheme is the method of choice as long as the fixed-point iteration is convergent.^64^ The SAFE_DIIS option automatically switches to the steepest descent (SD) approach whenever a DIIS step points away from the minimum. But, since this leads to an unreasonably slow convergence behavior, this should be prevented from the outset by setting STEPSIZE 0.1 or even smaller, i.e.
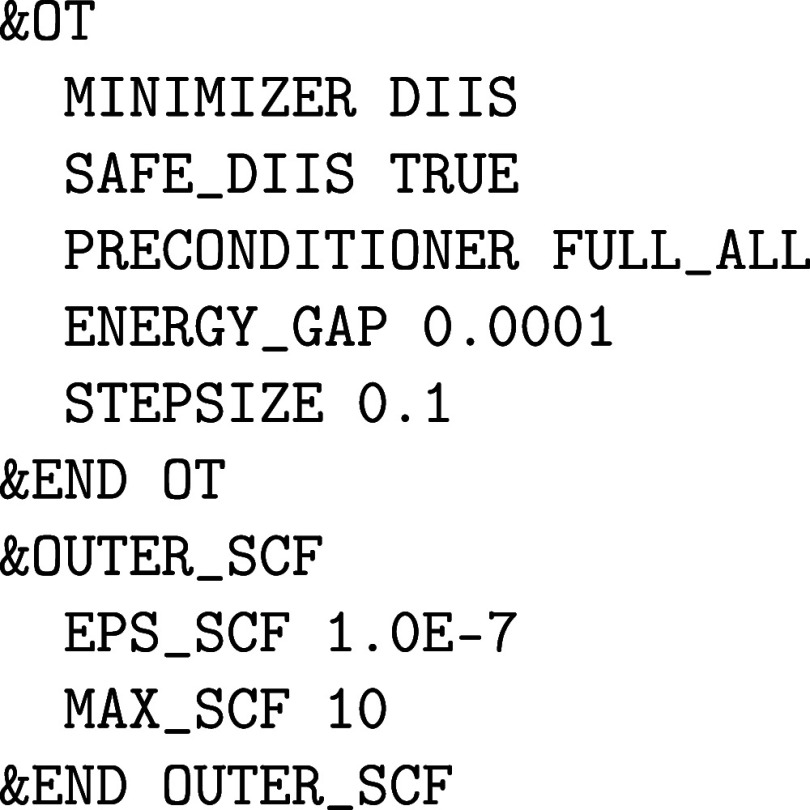


If this is not successful, it is advisible to shift to the conjugate gradient (CG) method in conjunction with LINESEARCH ADAPT, and otherwise to the traditional diagonalization alluded to above.

#### 2.1.5 Brillouin-Zone Sampling Using **k**-Points

It is possible to run periodic calculations with **k**-point sampling in CP2K, with both the GPW and GAPW methods. For each **k**-point in the first Brillouin zone, a generalized complex Hermitian eigenvalue problem must be solved, i.e.

10 $$K^{k}C^{k}=S^{k}C^{k}\varepsilon^{k}$$

where **K** ^**k**^, **S** ^**k**^, **C** ^**k**^ and **ε** ^**k**^ are the **k**-point dependent KS matrix, overlap matrix, MO coefficients and eigenvalues, respectively. In CP2K/Quickstep, the KS and overlap matrices are first calculated in real-space, before being Fourier transformed to reciprocal-space. Hence, the overlap matrix elements read as

11 $$S_{\mu \nu }^{k}=\sum_{R}e^{ik\cdot R}S_{\mu \nu }^{R}$$

with

12 $$S_{\mu \nu }^{R}=\int \varphi_{\mu }\left(r\right)\varphi_{\nu }\left(r-R\right)dr$$

where **R** is a unit cell translation vector. Similarly, *K*_*μν*_^**R**^ is the KS matrix element of an AO φ_μ_ in the unit cell and φ_ν_ in a periodic cell translated by **R**, respectively. Note that the number of translation vectors **R** depends on the diffuseness of the basis set: periodic images are considered as long as one of their basis functions overlaps with the unit cell. The same amount of work is done in Γ-point calculations, except that contributions from neighboring cells are added up in a single matrix.

A standard Γ-point DFT input file can be modified for a **k**-point calculation by adding a &KPOINTS subsection in &DFT:
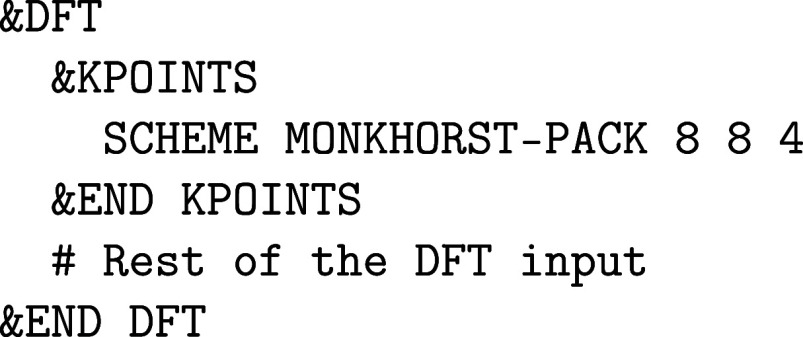


In this example, an 8 × 8 × 4 Monkhorst–Pack **k**-point mesh is generated.^70^ Note that every **k**-point can also be specified individually with its coordinates and weight using the KPOINT keyword. At the time of writing, CP2K does not automatically exploit symmetries to reduce the number of **k**-points (except for time-reversal symmetry, i.e. **k** = – **k**). After a converged SCF calculation, band structure data can be generated by adding the following &PRINT subsection:
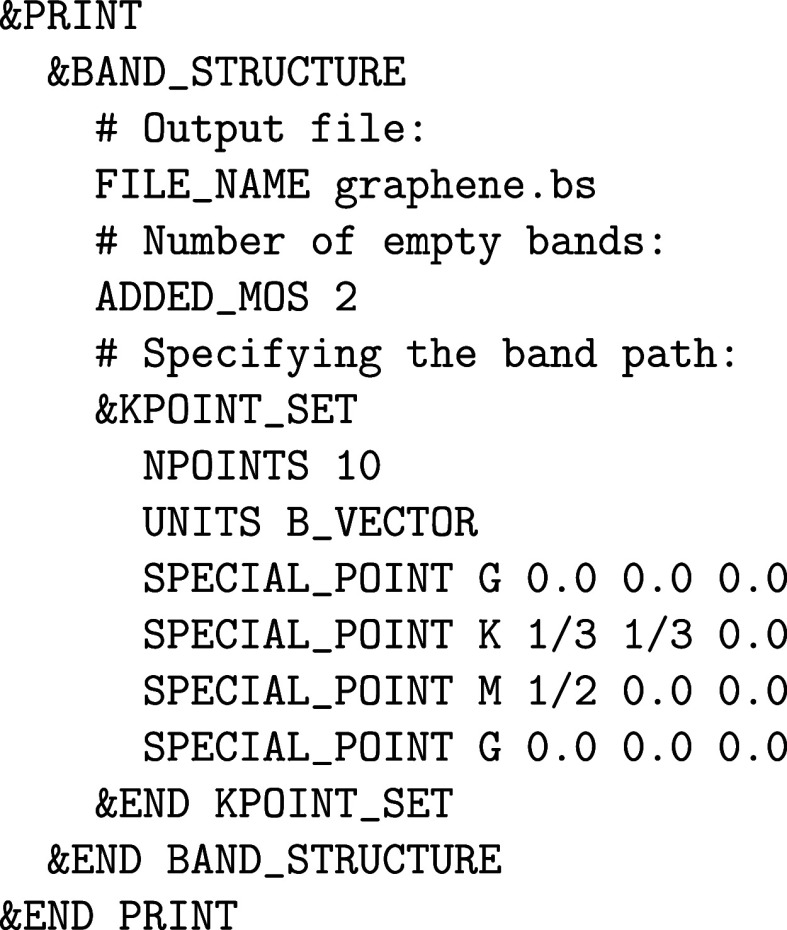


Here, a graphene band structure is calculated along the Γ–K–M−Γ path, with 10 **k**-points along each segment. Note that any path of any density can be generated, since all relevant quantities are calculated in real-space first, before being Fourier transformed to the specified path. The only requirement is that the original SCF uses a sufficiently converged **k**-point mesh, as defined in &KPOINTS.

Finally, note that **k**-points are not yet implemented for all electronic structure methods, but primarily for DFT, resolution-of-the-identity HF exchange (RI-HFX), and the *GW* approximation. It also has limited support with respect to SCF schemes: only full diagonalization (with DIIS) is available, but OT is not. As for Γ-point calculations, it is possible to print binary WF restart files via the &SCF%PRINT%RESTART section when running **k**-point calculations. However, due to different format and content, restarting a **k**-point calculation from a Γ-point restart file (and vice versa) is not possible.

### 2.2 Hartree–Fock and Hybrid Density Functional Theory

The central quantity in HF and hybrid DFT calculations is the Fock matrix **F**. For nonperiodic calculations, it is simply defined as

13 $$F_{\mu \nu }=\sum_{\sigma ,\lambda }P_{\sigma \lambda }\left(\mu \sigma |\nu \lambda \right)$$

where μ, ν, σ, λ defines elements of the AO basis, whereas *P*_σλ_ is the density matrix, and (μσ|νλ) represents 2-electron 4-center electron repulsion integrals (ERI), which in the Mulliken notation reads as

14 (μσ|νλ)=∫dr∫dr′φμ(r)φσ(r)×g(|r−r′|)⁡φν(r′)φλ(r′)

Therein *g*(|**r** – **r′**|) is the interaction potential, conventionally chosen to be the 1/|**r** – **r′**| Coulomb potential.

In periodic HF calculations, however, contributions from periodic images of the unit cell need to be explicitly accounted for. Elements of the Fock matrix that involve an AO φ_μ_(**r**) in the unit cell and an AO φ_ν_(**r** – **b**) in a periodic image translated by **b** are defined as

15 $$F_{\mu \nu }^{b}=\sum_{\sigma ,\lambda }\sum_{a,c}P_{\sigma \lambda }^{c}\left(\mu^{0}\sigma^{a}|\nu^{b}\lambda^{a+c}\right)$$

where contributions from AOs translated by **a** and **a** + **c** are summed. In the special case of a Γ-point calculation, the sum over **c** can be carried out first, and the resulting periodic density matrix extracted from the sum. A subsequent sum over **a** and **b** yields the periodic Γ-point Fock matrix

16 Fμν=∑σ,λPσλ∑a,b,c(μ0σa|νbλa+c)=∑σ,λPσλ⁡Tμ,σ,ν,λ

where the 4-index tensor *T*_μ,σ,ν,λ_ is obtained by summing ERIs involving AOs located in various periodic images. The number of periodic images necessary depends on the diffuseness of the basis set and the range of the interaction potential *g*(|**r** – **r′**|). For a nonzero ERI, μ^**0**^ and σ^**a**^ (respectively ν^**b**^ and λ^**a**+**c**^) must overlap and the product μ^**0**^σ^**a**^ must be within the range of the product ν^**b**^λ^**a**+**c**^. The HF exchange (HFX) energy is obtained by contracting the density matrix with the Fock matrix

17 $$E_{HFX}=-\frac{1}{2}\sum_{\mu ,\nu }P_{\mu \nu }F_{\mu \nu }$$

and the nuclear forces are obtained by analytic differentiation of the ERIs with respect to the atomic positions.

The implementation of HFX in CP2K is documented in ref 71. Hybrid DFT and HF calculations require the &HF input subsection of &DFT%XC. The FRACTION keyword defines the amount of exact exchange that is added to the calculation. Note that it is always necessary to define a XC functional in the &XC_FUNCTIONAL subsection. For example, the input for a pure HF calculation looks like
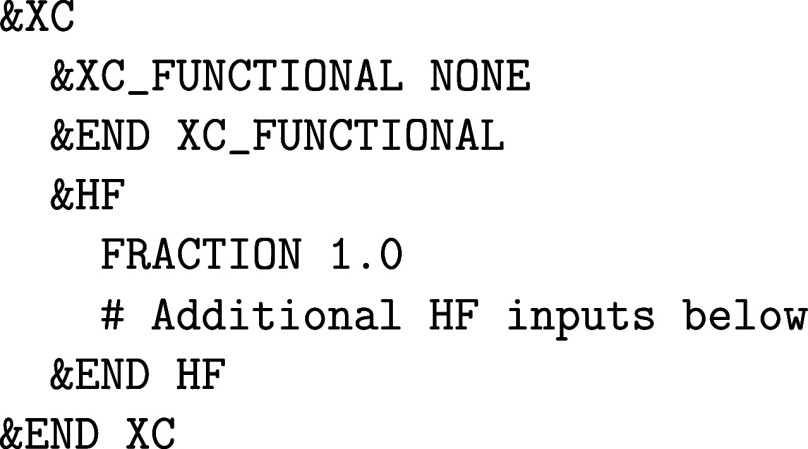


whereas the input for the PBE0 functional reads as
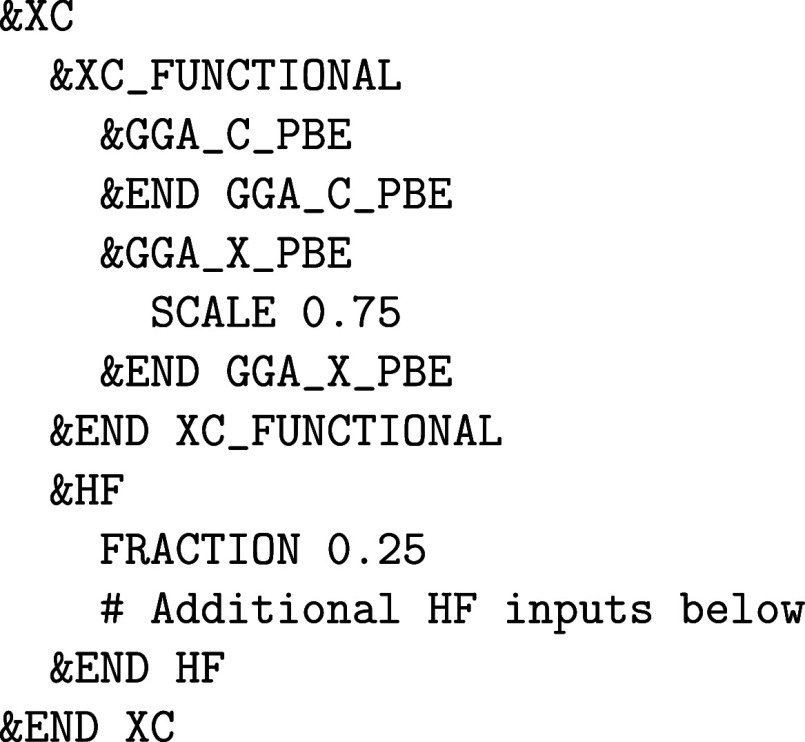


An important input subsection within the &DFT%XC%HF section is &INTERACTION_POTENTIAL, which controls the parameters of *g*(|**r** – **r′**|) in the 2-center 4-electron ERIs of eq 14. The POTENTIAL_TYPE keyword selects among the various interaction potentials implemented, while the remaining keywords in this subsection refer to the specifics of the chosen potential. The default value of POTENTIAL_TYPE is COULOMB for the standard 1/|**r** – **r′**| potential. However, this choice is typically not suitable for periodic calculations, as it introduces spurious exchange interactions between an electron and its periodic images. A possible choice is TRUNCATED, which refers to the truncated Coulomb potential

18 $$g\left(r\right)=\{\begin{array}{l} 1/r,\mathrm{if}\;r\leq R_{C}\\ 0,\mathrm{if}r>R_{C} \end{array}$$

which vanishes beyond a cutoff radius *R*_C_. For a cubic cell of length *L*, *R*_C_ ≤ *L*/2 must hold. This is to say that the truncation radius must be shorter than half the smallest cell dimension, but large enough for the exchange energy to converge. Values of 6 Å are typically enough for systems with a large band gap. Systems with smaller band gaps, however, might require larger cutoff values. If the simulation cell is not large enough for both requirements, a supercell must be constructed instead. For ideal performance, the shortest cutoff that converges the energy should be taken. The value of *R*_C_ is set with the keyword CUTOFF_RADIUS (in Angstroms). Periodic calculations with global hybrids like PBE0 or B3LYP should use this potential.^37,72,73^

An alternative choice is POTENTIAL_TYPE SHORTRANGE, which comes from the decomposition of the Coulomb potential in a short- and a long-range term

19 $$\frac{1}{r}=\frac{1-\mathrm{erf}\left(\omega \right)}{r}+\frac{\mathrm{erf}\left(\omega \right)}{r}$$

The OMEGA keyword controls the range-separation parameter. The HSE06 functional, for instance, involves the short-range potential with a value of 0.11 for ω. More involved range-separated hybrid functionals can be configured with the MIX_CL_TRUNC interaction potential, which mixes the long-range part of eq 19 and the truncated Coulomb potential. For example, the exact exchange part of the rCAM-B3LYP functional looks like:
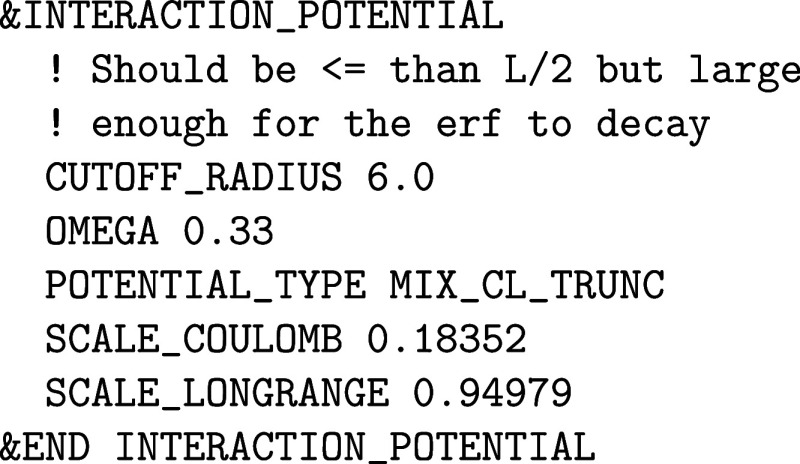


The evaluation of the ERIs *T*_μ,σ,ν,λ_ in eq 16 can be very demanding and is often the bottleneck of calculations requiring exact exchange. Various screening methods can help reduce the computational cost. Most importantly, Schwarz screening provides an upper bound to any 2-electron 4-center ERI. The EPS_SCHWARZ keyword of the &XC%HF%SCREENING section sets a threshold, and the evaluation of any ERI estimated to be smaller is skipped a priori. The default value of 10^–10^ is rather safe, and increasing that number can lead to significant performance gains. Yet, a too large value will lead to numerical instabilities and eventually even inaccurate results. The EPS_SCHWARZ_FORCES keyword does the same for ERI derivatives and is defined as a function of EPS_SCHWARZ by default.

Gaussian type orbital (GTO) basis sets have an exponentially vanishing tail, theoretically extending to infinity. Hence, another important screening threshold is &DFT%QS%EPS_PGF_ORB, which sets these tails to zero once a GTO reaches the value of EPS_PGF_ORB, thereby effectively defining the range *D*_μ_ of an AO

20 $$\{\begin{array}{ll} \varphi_{\mu }\left(r\right)=\varepsilon_{PGF} & \mathrm{if}\;\left|r\right|=D_{\mu }\\ \varphi_{\mu }\left(r\right)=0 & \mathrm{if}\;\left|r\right|>D_{\mu } \end{array}$$

This is to say that two AOs, φ_μ_ centered on atom A at position **R**_**A**_ and φ_ν_ centered on atom B at position **R**_**B**_, only overlap if |**R**_**A**_ – **R**_**B**_| ≤ *D*_μ_ + *D*_ν_. This and the choice of interaction potential effectively define the number of periodic cells to consider in the sum of eq 16. Hence, the impact on the computational performance can be significant. The default value of 10^–5^ for EPS_PGF_ORB is usually a good compromise between accuracy and speed. A value of 10^–6^ is already considered to be rather safe, whereas an even tighter threshold may negatively impact computational efficiency without necessarily improving accuracy. Since AO overlaps are key to screening, the choice of basis set can also affect performance. In particular, it is not recommended to use diffuse basis sets for HFX calculations, such as those from the previously described MOLOPT family. Instead, the ADMM and/or RI-HFX methods, described below, should be used with diffuse basis sets.

Since the ERIs only depend on atomic positions, it is in principle possible to precalculate and store them all in main memory to simply contract them with the new density matrix at each SCF step. The ERIs only need to be recalculated when the atoms move, e.g. at each AIMD or geometry optimization step. However, due to the 4-index nature of the *T*_μ,σ,ν,λ_ tensor, the memory requirements for the storage of the ERIs can become very large. The MAX_MEMORY keyword of the &XC%HF%MEMORY section caps the memory consumption of the whole HFX code. This value corresponds to the maximum allowed memory usage per MPI rank in MiB. After allocating various buffers and work arrays, the remaining memory is used to store as many ERIs as necessary and possible. To maximize the performance, the value of MAX_MEMORY should be as high as possible, while leaving enough space for the rest of the calculation. Usually, allocating 50–75% of the available memory to HFX works well. If there is not enough space to store all ERIs, some of them will be calculated on-the-fly at each SCF step, potentially slowing down the calculation. It is recommended to track the output of a HFX calculation to verify this (look for HFX_MEM_INFO in the output). If the number of spherical ERIs calculated on-the-fly is nonzero, significant gains can be expected by raising the value of MAX_MEMORY, or the total amount of memory by using more computing nodes. However, as of the publication of this paper, the HFX code runs only on CPUs. It is recommended to restart HFX calculations from a GGA level converged WF of the same system in order to reduce the number of necessary SCF steps.

#### 2.2.1 Auxiliary Density Matrix Method

In most HFX based calculations, the evaluation of *T*_μ,σ,ν,λ_ in eq 16 is the main bottleneck. Unfortunately, the cost of evaluating 2-electron 4-center ERIs is tightly connected to the size and diffuseness of the selected basis set. Performing calculations with high quality basis sets can become untractable, especially for periodic systems. The ADMM is designed to tackle this issue. In this approximation, the exact exchange energy is calculated as

21 EHFX=ExHF[P̂]+(ExHF[P]−ExHF[P̂])≈ExHF[P̂]+(ExGGA[P]−ExGGA[P̂])

where **P** is the density matrix in the primary basis and **P̂** is the density matrix expressed in an auxiliary basis that is smaller and/or less diffuse than the former basis set. The exact exchange energy is efficiently evaluated in the smaller auxiliary basis, while the difference in exchange energy due to the change of basis set is approximated at the DFT GGA level. The approximation is assumed to be accurate, provided that **P** ≈ **P̂**.

Given a choice of auxiliary basis set and GGA exchange functional, there exist different flavors of ADMM. In its simplest form, known as ADMM2, the density is simply projected from the orbital basis onto the auxiliary basis set. On the other hand, ADMM1 involves a purification of the auxiliary density matrix to ensure its idempotency and particle conservation. Both ADMM1 and ADMM2 are described in detail in the original publication by Guidon and co-workers.^50^ Subsequent work by Merlot and co-workers introduced additional three flavors that are ADMMP, ADMMQ, and ADMMS, respectively.^74^ The latter impose various physically motivated constraints to the approximation in a simpler way than the ADMM1 purification scheme. Note that most post-SCF methods, which can be used in conjunction with ADMM (RPA, MP2, TD-DFT, density-corrected DFT, etc.), only support ADMM2.

To run an ADMM calculation, a valid HFX input file can be modified as follows. The &AUXILIARY_DENSITY_MATRIX_METHOD subsection should be added to &DFT, i.e.
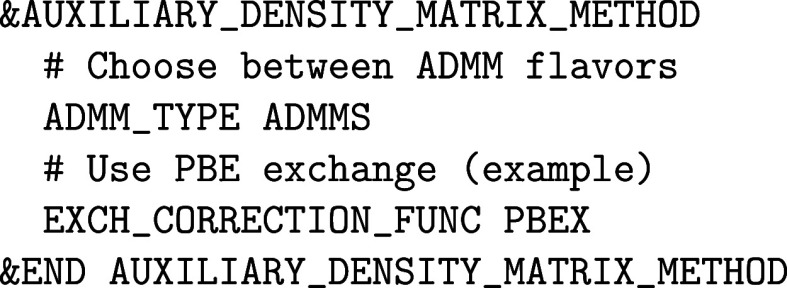


Note that various other keywords exist, but their usage is not recommended. Indeed, ADMM_TYPE automatically sets their value for the desired ADMM flavor.

A critical aspect of running ADMM calculations is the choice of auxiliary basis set. The FIT basis family was originally developed to run in conjunction with MOLOPT basis sets. These basis sets can be found in the BASIS_ADMM_MOLOPT file, and their hierarchy is described in ref 50. More recently, correlation-consistent ADMM basis sets were developed to be used with the ccGRB family, which can be found in the BASIS_ADMM_UZH and BASIS_ccGRB_UZH files, respectively. The usage of the more recent basis sets is recommended. Note that all-electron auxiliary basis sets are also available in the file BASIS_ADMM_ae to be used in conjunction with Jansen’s pcseg basis family.^75,76^ The ADMM basis set file must be added to the input file, and the specific choice of basis specified for each atomic species, hence:
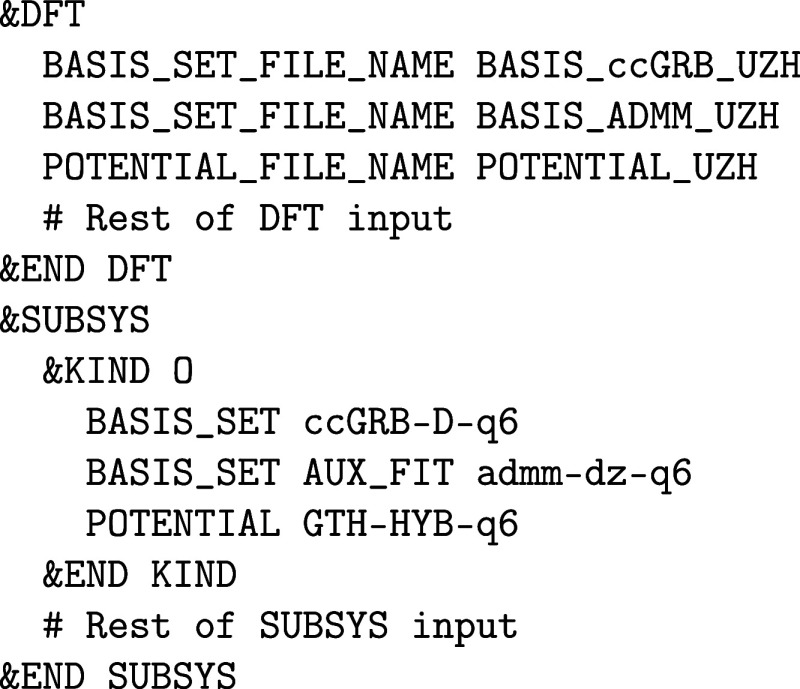


Note that with the ADMM approximation, a full-fledged HFX calculation is still being carried out, albeit with a smaller base set. All input considerations discussed in the &HF section apply here as well.

#### 2.2.2 Resolution-of-the-Identity Hartree–Fock Exchange

In the resolution-of-the-identity (RI) approximation, the 2-electron 4-center ERIs can be expressed in terms of 2- and 3-center integrals, i.e.

22 (λσ|μν)≈(λσ⌊P)⁡(P⌊Q)−1(Q|R)⁡(R⌊S)−1(S⌊μν)=(λσ⌊P)⁡VPQ−1(Q|R)⁡VRS−1⁡(S⌊μν)

where the Einstein summation convention is used for the sum over the RI basis elements *P*, *Q*, *R*, *S*. The | and ⌊ symbols represent the HF interaction potential and RI metric, respectively. The RI approximation is exact if the RI basis spans the same space as all possible AO products, and if the RI metric is the same as the interaction potential. This approach allows to shift the computational effort from ERI evaluations to sparse tensor contractions, which can be efficiently performed on GPUs.^77^ Note that RI-HFX is not always a suitable choice, and the standard 4-center approach remains relevant in most cases. In practice, RI-HFX shows a performance edge for nonperiodic calculations of molecules with large basis sets (where both the interaction potential and the RI metric are the standard 1/*r* Coulomb measure), and for periodic calculations of small to medium sized dense solids. The CP2K/Quickstep implementation, as well as a discussion on the choice of RI metric and application cases, can be found in ref 78. The RI-HFX method is compatible with all flavors of ADMM, and analytic gradients to compute the nuclear forces are implemented.

The RI-HFX method can be invoked by adding the following subsection to the &HF section of the CP2K input file:
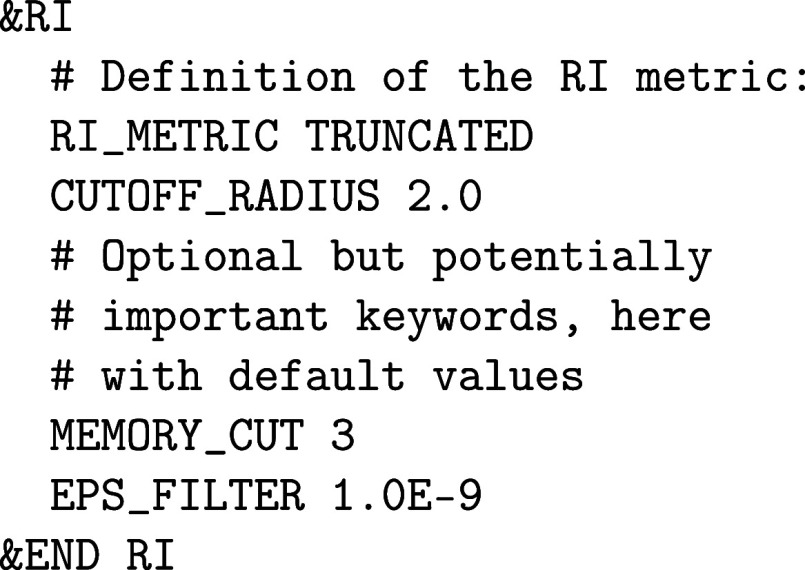


This input snippet defines the RI metric to be used in a similar fashion to the &INTERACTION_POTENTIAL subsection of the same parent &HF section. By default, the RI metric takes the same parameters as the latter. The RI metric is usually chosen to be a very short-ranged interaction in order to increase sparsity and to reduce the computational effort. The range of the RI metric is a trade-off between performance and accuracy, with the overlap as the most extreme case, which is known as the RI-SVS approximation.^79^ The MEMORY_CUT keyword controls batching in the contraction of eq 22: potentially very large tensors are necessary to hold intermediate results, and batching allows processing them with relatively limited resources. Note that this keyword only concerns the previously mentioned contraction step, but it does not affect the total memory footprint of CP2K. Since batched tensor contractions come with an overhead, it is recommended to keep the value of MEMORY_CUT as low as the employed resources allow for. It is generally more efficient (but not always practical) to increase the amount of resources, rather than increasing the keyword’s value. Note that RI-HFX calculations of large systems can be memory intensive, and that unlike the standard 4-center method, there is no easy way to predict the required memory. The EPS_FILTER keyword controls the filtering of small elements out of the sparse tensors. The default value of 10^–9^ is a good compromise between accuracy and efficiency. Increasing this value will lead to performance gains, but it is recommended to proceed with care. The rest of the &HF input section can remain the same. Note, however, that the &MEMORY and the &SCREENING subsections have no effect during a RI-HFX calculation. All considerations on the choice of interaction potential or EPS_PGF_ORB discussed for the original 4-center method also apply here.

The choice of RI basis set is important for this type of calculations, both in terms of performance and accuracy. It is generally recommended to use optimized bases when possible (e.g., cc-pVTZ-JKFIT with the cc-pVTZ orbital basis).^80^ The cc-pVTZ all-electron basis set, however, is not distributed with CP2K: it would need to be downloaded independently at the EMSL Basis Set Exchange for instance.^44^ If not available, the RI basis is generated on-the-fly using the method of Stoychev and co-workers.^81^ This type of basis is safe to use and accurate, but is generally larger, and thus less efficient, than its optimized counterparts. The CP2K input file can be modified as follows to specify the RI basis:
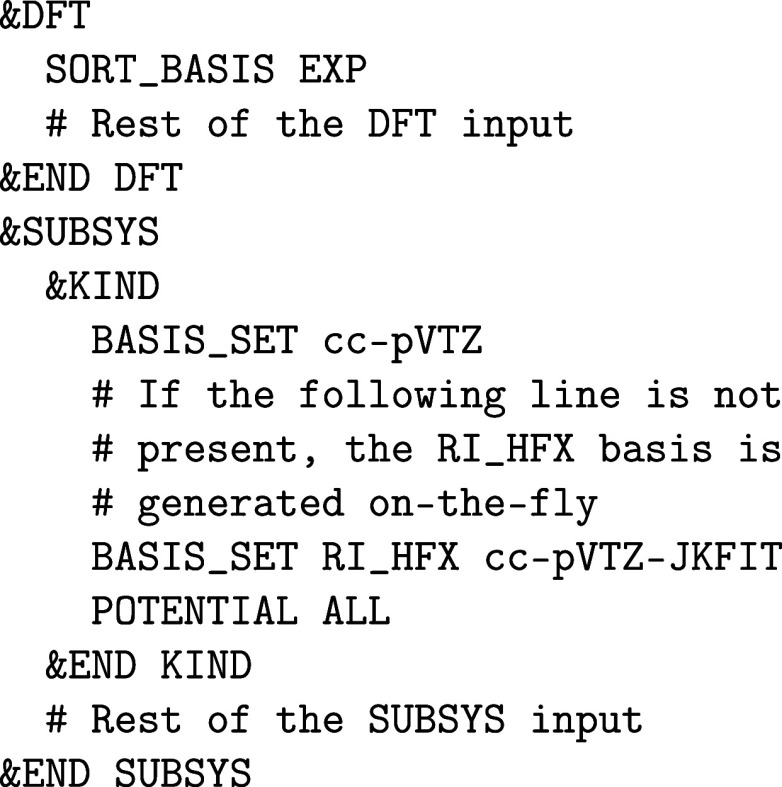


Note the SORT_BASIS keyword, use is highly recommended, as it reorders the AO according to their Gaussian exponent, thus increasing sparsity.

#### 2.2.3 Resolution-of-the-Identity Hartree–Fock Exchange with **k**-Point Brillouin-Zone Sampling

The RI-HFX with **k**-point sampling (RI-HFXk) method allows for periodic HFX calculations of small unit cells with **k**-point sampling. As for **k**-point sampling using DFT (see Section 2.1.5), the **k**-point dependent Fock matrix is first calculated in real-space before it is Fourier transformed, i.e.

23 $$F_{\mu \nu }^{k}=\sum_{R}e^{ik\cdot R}F_{\mu \nu }^{R}$$

where **R** is a unit cell translation vector and *F*_μν_^**R**^ is defined in eq 15. Contrary to Γ-point calculations, the sum over periodic images cannot be performed a priori, and the results are stored in a single 4-index tensor. As a result, storing the integrals is intractable, and all 4-center 2-electron ERIs would need to be calculated on-the-fly at each SCF step. Although possible, such an approach would be very expensive. Thus, an efficient alternative using an atom-specific RI scheme was developed.^82^

The RI-HFXk method can be invoked by adding a &KPOINTS section to a RI-HFX input file. The **k**-point specific input is the same as described in Section 2.1.5, including the optional band structure information. It is compatible with the ADMM approximation, and nuclear forces are implemented. As for any HFX method, it is generally recommended to restart from a converged GGA level (**k**-point) WF. Extra care must be paid to some parts of the input file, as listed below:

- &HF%INTERACTION_POTENTIAL: As for any periodic HF calculation, a short-range potential must be selected. The *L*/2 requirement now refers to the Born–von Kármán supercell, i.e. a supercell obtained by multiplying the unit cell by the number of **k**-points in each direction. As for Γ-point HFX, the potential range should be long enough for convergence, while kept as short as possible for performance.
- &RI%RI_METRIC: In the special case of RI-HFXk, the range of the RI metric barely impacts the performance. It is hence recommended to always take it to be the same as the interaction potential for maximal accuracy (default option).
- &QS%PW_GRID_BLOCKED: It is recommended to set this keyword to FALSE. This ensures that calculations of small unit cells with a lot of CPUs can go ahead without crashing.
- &SCF%EPS_DIIS: The default threshold value to start DIIS is 0.1. Experience has shown that better convergence is achieved when this value is lower, typically around 0.05.
- &RI%KP_NGROUPS: There is a lot of computational work to be done, and most of it can be efficiently parallelized over MPI subgroups. This is controlled with the KP_NGROUPS keyword, which should be a divisor of the total number of ranks. Using *N* subgroups should accelerate the calculations by a factor ∼*N* and increase its memory usage by the same factor.
- &RI%KP_STACK_SIZE: Most of the computational effort is spent on contracting tensors. In some cases, more efficient batched contractions can take place, at the cost of greater memory usage. The default value of 32 is meant for small systems of a few atoms. Larger systems will require more memory overall and might not have enough room for the storage of temporary tensors (in which case, use a smaller stack size).

Generally, the RI-HFXk method is very demanding, both in terms of compute and memory. It was designed and optimized for small to medium size systems with dense **k**-point meshes (e.g., 2D materials, highly symmetric crystals, etc.). The computational cost of the method scales as $O\left(N_{atoms}^{3}N_{img}^{2}\right)$ , where *N*_img_ refers to the number of periodic images required (depends on the diffuseness of the base set and the range of the interaction potential). The scaling of the memory usage is harder to predict, but experience suggests that it is steep, too. For large systems, a Γ-point calculation with an equivalent supercell will be much more efficient due to the asymptotical linear scaling of the 4-center HFX implementation. The details of the RI-HFXk method are not trivial, and it is recommended to read ref 82 before use.

### 2.3 Post-Hartree–Fock and Double-Hybrid Density Functional Theory

In addition to incorporating exact exchange via HFX, as described in Section 2.2, electron correlation beyond conventional DFT can be added via efficient MP2 and RPA methods described in this section. Most of them are available for condensed phase systems including nuclear forces, if not explicitly mentioned otherwise.

#### 2.3.1 Second-Order Møller–Plesset Perturbation Theory

The MP2 method computes

24 $$E^{\left(2\right)}=-\sum_{ijab}\frac{\left(ia|jb\right)\left[2\left(ia|jb\right)-\left(ib|ja\right)\right]}{\epsilon_{a}+\epsilon_{b}-\epsilon_{i}-\epsilon_{j}}$$

employing canonical HF reference orbitals, each with the real-space representation $\phi_{p}\left(\mathrm{r⃗}\right)$ and an orbital energy ϵ_p_. The ERIs are usually calculated in the AO basis first and then in four steps transformed to the MO basis. CP2K/Quickstep provides three different implementations: a direct/canonical implementation, a GPW-based implementation^83^ and a RI-based implementation.^84^ All MP2 implementations scale with the fifth power of the number of atoms. The canonical implementation is available for GPW and GAPW reference calculations allowing core-corrections. However, it is the most costly implementation and analytical gradients (nuclear forces, stress tensors, etc.) are not available.

All flavors of MP2 calculations are available via the &MP2 subsection in the &DFT%XC%WF_CORRELATION section and activating the respective method. For direct canonical MP2, it is METHOD DIRECT_CANONICAL. Further keywords, which are also of interest for all other implementations, are

- &WF_CORRELATION%MEMORY: the allowed memory for the MP2 calculation.
- &WF_CORRELATION%GROUP_SIZE: it needs to be a divisor of the total number of processes. Lower values reduce communication costs, but increase memory requirements.
- &WF_CORRELATION%INTEGRALS: optional section to configure the ERI method via the ERI_METHOD keyword and by the INTERACTION_POTENTIAL subsection the corresponding interaction potential (default: Coulomb potential).

The direct MP2 implementation serves as a reference method for medium-sized systems, as its computational demands become easily untractable for larger systems. This implementation also does not benefit from GPU acceleration.

For better performance, the GPW-based implementation should be used.^83^ It skips the first two transformation steps by calculating half-transformed ERIs from the product density $\phi_{i}\left(\mathrm{r⃗}\right)\phi_{a}\left(\mathrm{r⃗}\right)$ directly. It is available by setting &MP2%METHOD MP2_GPW. The GPW-integration is configured with the &WFC_GPW subsection inside the &WF_CORRELATION%INTEGRALS section. As with the original GPW method, there are two tunable parameters CUTOFF and REL_CUTOFF with the same meaning and should be tuned as in the original DFT-based GPW approach described in Section 2.1.1. Yet, due to the different requirements on the accuracy and the properties of the densities, primary cutoffs of 150 to 300 Ry are typically sufficient for most applications. Using the grid and the distributed block compressed sparse row (DBCSR) libraries, the GPW-based implementation runs efficiently on GPUs, enabling MP2 calculations on systems containing hundreds of atoms.

The RI approximation to the ERIs allows for further computational savings.^85^ If we expand products of orbitals in a set of auxiliary functions denoted by *P*, *Q*, ..., the ERI tensor factorizes approximately into a rank-3 tensor *B*, i.e.

25 $$\left(ia|jb\right)\approx \sum_{P}B_{iaP}B_{jbP}$$

where *B* is calculated from the respective three-center integrals (*P*|*Q*) and (*P*|*ia*). These can be calculated using the GPW-approach in a similar fashion as discussed above, or alternatively using a Minimax-Ewald (MME) approach or the Obara–Saika (OS) scheme, which can be set by the previously mentioned &INTEGRALS%ERI_METHODS keywords. For that, the RI_MP2 section replaces the MP2 section.
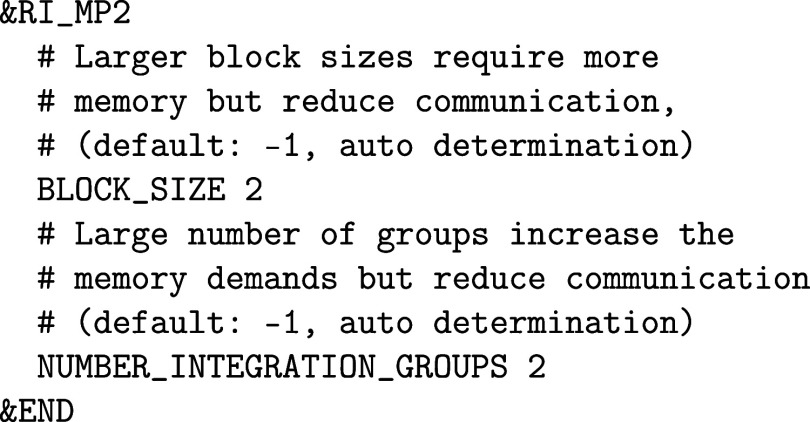


The most important keywords are BLOCK_SIZE and NUMBER_INTEGRATION_GROUPS. By default, these parameters are determined automatically from the available memory, but their tuning may be of interest for calculations on a larger number of systems. Large block sizes and large numbers of integration groups reduce communication costs at the expense of a higher memory demand. The number of integration groups must be a divisor of the number of subgroups (number of MPI ranks divided by the GROUP_SIZE). See ref 84 for further information on the technical details and GPU acceleration.

As before, the RI approximation requires the user to specify an auxiliary basis set. In general, the auxiliary basis needs to be adapted to the primary basis set to yield an acceptable accuracy and sufficient speed-up compared to the canonical implementations. CP2K has several options: preoptimized, self-optimized, and auto-optimized basis sets. Optimization of the auxiliary basis sets is available according to ref 86. A compilation of preoptimized basis sets is available in the previously mentioned data directory of the CP2K GitHub repository. If none is available for a certain basis set or the optimization is not required, we recommend auto-optimized basis sets. They are available by omitting the specification of the auxiliary basis set in the &SUBSYS%KIND section and setting the AUTO_BASIS keyword in the &DFT section.

For the RI-MP2 implementation, analytical gradients and stress tensors are available.^87,88^ Its implementation is based on the Lagrangian formalism of the MP2 energy by introducing the &WF_CORRELATION%CANONICAL_GRADIENTS section. The Lagrangian formalism requires the solution of the coupled-perturbed HF (CPHF) equations configured in the &CANONICAL_GRADIENTS%CPHF subsection:
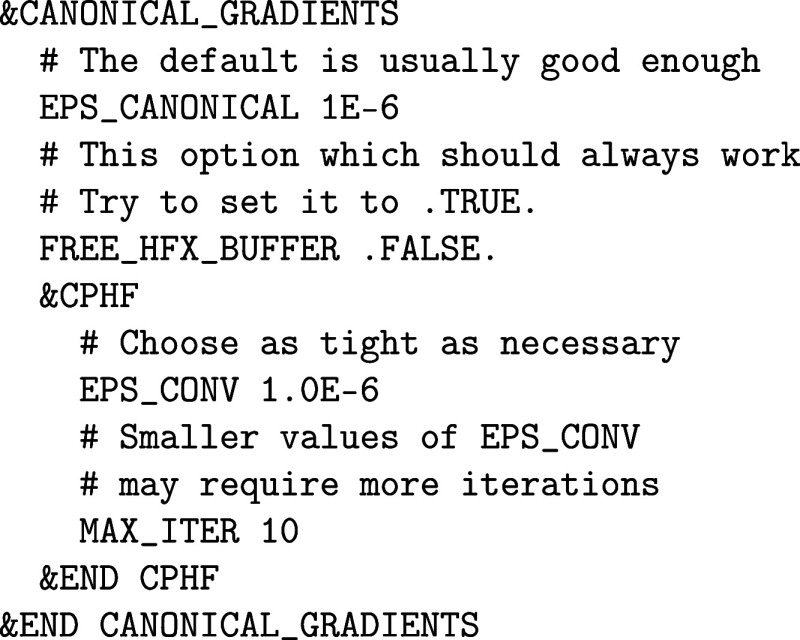


Therein, EPS_CANONICAL is a MP2 specific parameter affecting the numerical stability of MP2 analytic gradient calculations. Higher values improve the accuracy, but increase the computational costs significantly. The FREE_HFX_BUFFER keyword frees the HF buffers before the gradient calculations to increase the amount of available memory necessary for it. By default, it is turned off because the integrals cannot always be recovered for technical reasons. The parameters of the &CPHF section affect the numerical solution of the CPHF equations. The EPS_CONV parameter affects the accuracy of the solution of the CPHF equations, which are to be chosen as tightly as necessary and as high as possible. The MAX_ITER parameter determines the maximum number of iteration steps. If convergence is not observed, then the orbitals are not sufficiently converged, or the MP2 method is not suitable for the given system because of too high electron correlation.

The accuracy of MP2 calculations is mostly affected by the size of the primary basis set. In contrast to HF or DFT calculations, where the basis set error decays exponentially with the number of basis functions, the error of the MP2 energy decays with the reciprocal number of basis functions.^89^ Thus, either sufficiently large basis sets or an extrapolation to the complete basis set limit is required. Independently of an extrapolation scheme, basis sets should have a quality of at least triple-ζ or augmented double-ζ for accurate results. Finally, the basis sets should contain enough polarization functions as in correlation-consistent basis sets.

The performance of MP2 calculations in CP2K depends on several factors. Conceptually, the number of operations increases linearly to quadratically with the number of auxiliary basis functions, suggesting that optimized auxiliary basis sets should be employed whenever possible. The calculation of the integrals using the GPW approach relies on the efficient grid library and sparse matrix-contractions with DBCSR.^90^ The actual contraction of the ERIs to the MP2 energy relies on a well-optimized BLAS implementation for the matrix–matrix multiplications. For GPU acceleration, CP2K is either linked to an accelerated BLAS implementation or switches to the SpLA library.^91^

Although the calculation of the MP2 energy shows a steeper scaling than a HF calculation, the HF kernel can still dominate the computational costs in case of diffuse basis functions. This can be overcome by the ADMM,^50^ which is available for MP2 energy and gradient calculations.^92^

Double-hybrid functionals improve upon MP2 by improving the description of short-ranged correlation effects using DFT.^93^ Instead of starting from a HF description, double-hybrid functionals start from hybrid DFT and then add a certain amount of spin-rescaled MP2 energy. In CP2K/Quickstep, this is achieved with the following modifications inside the &XC section:

- &XC_FUNCTIONAL: set up the respective DFT part. In addition to the natively implemented XC functionals, an even larger selection of functionals is available through the LibXC library.^94^
- &HF: set up the respective HF interaction operator. For simple functionals, the adjustment of the FRACTION parameter is sufficient. For more elaborate functionals consider the parameters in the &INTERACTION_POTENTIAL subsection.
- &WF_CORRELATION: the keywords SCALE_S and SCALE_T determine the weight of singlet and triplet contributions. The &INTEGRALS%INTERACTION_POTENTIAL subsection allows the adjustment of the operator.

Beware that CP2K does not read any HF related information from LibXC such that all parameters have to be looked up and set up manually. We recommend testing the setup against reference values for the respective double-hybrid functional as the setup is error-prone. Some examples can be found at https://github.com/cp2k/cp2k/tree/master/data/xc_section. Nuclear forces and stress tensors are analytically available throughout for double-hybrid functionals.^92,95^

#### 2.3.2 Random Phase Approximation and Laplace-Transformed Scaled Opposite-Spin Second-Order Møller–Plesset Perturbation Theory

The RI approach to the direct RPA (dRPA) method given by its correlation energy contribution

26 EdRPA=−14π∫−∞∞dω⟨log(1+Q(ω))−Q(ω)⟩QRS(ω)=2∑iaBiaRϵa−ϵi(ϵa−ϵi)2+ω2BiaS

and the RI-based Laplace-transformed scaled opposite-spin MP2 (SOS-MP2)

27 ESOS‐MP2=−∫0∞dτTr(Q̅(τ)2)Q̅RS(τ)=∑iaBiaRe−(ϵa−ϵi)τBiaS

are implemented similarly due to the integration of a matrix-valued function and the contraction of the RI-tensor **B** to a frequency-dependent or time-dependent intermediate matrix **Q**(ω). Their energy-only implementation is discussed in ref 84. Although the integration may be performed analytically via the Plasmon equation,^96^ or the MP2 energy equation (see eq 24), we pursue the path of numerical integration due to its reduced scaling behavior of fourth power with respect to the number of atoms, as compared to the alternative with fifth order scaling.

CP2K/Quickstep implements two quadrature schemes: Clenshaw–Curtis^97^ and Minimax.^98^ The Clenshaw–Curtis quadrature is easily accessible and converges for all subsequent methods, but requires a larger number of quadrature points. In contrast to that, the Minimax quadrature rules are difficult to optimize and are thus pretabulated. The main advantage of Minimax quadrature rules lies in their higher accuracy than the Clenshaw–Curtis quadrature rules and are the recommended choice for RPA calculations and the only available option for SOS-MP2 calculations. The setup of these methods is similar to the setup of an (RI-)­MP2 calculation. The main difference is that the &RI_RPA section
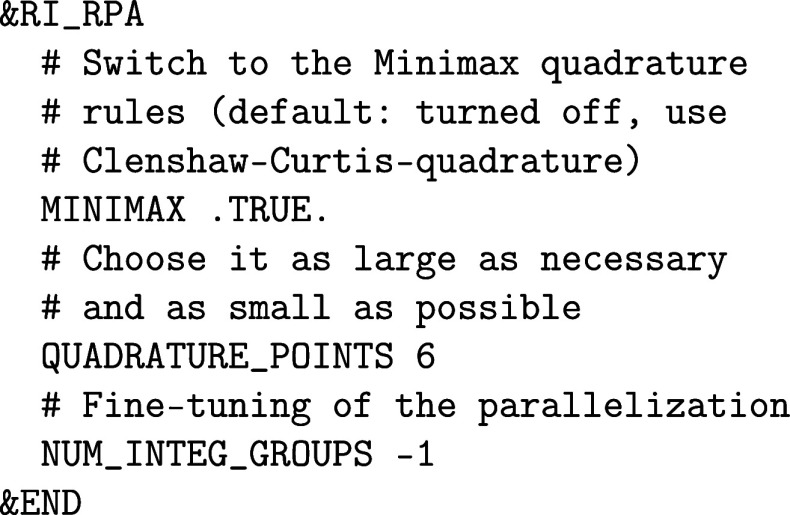
or the RI_SOS_MP2 section
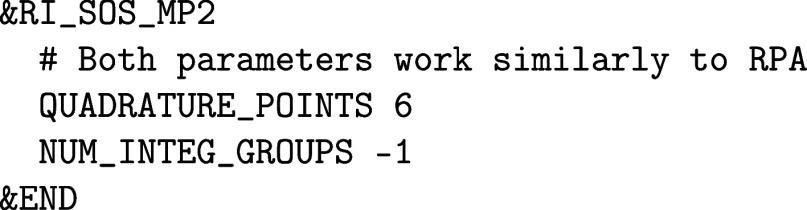
replaces the &MP2 or &RI_MP2-sections, respectively. Note that the Minimax quadrature has to be turned on manually for RPA calculations. This difference is related to RPA calculations being the precursor of *GW* bandstructure calculations, which are described in Section 3.2, requiring quadratures suitable for different kinds of integration kernels.

The RPA correlation energies are usually combined with exact exchange energies. In CP2K, the &RI_RPA section includes a &HF section, which is set up similarly to the ordinary &HF section used in HF and hybrid functional calculations. Because the ERIs are needed only once here, the MAX_MEMORY keyword in the &MEMORY should be set to zero in case of a simple RPA energy calculation to save memory. The ADMM acceleration can also be applied for the calculation of exact exchange energies by activating the ADMM keyword in the &RI_RPA section. We recommend the ADMM approximation for calculations with diffuse basis functions, which would otherwise increase the computation time tremendously due to their larger extent.

On top of the exact exchange and the dRPA correlation energy, one can also apply a beyond-RPA scheme. The RSE keyword activates the renormalized single excitations (RSE) correction to account for nondiagonal Fock-matrix elements.^99^ In addition to that, the &RI_RPA%EXCHANGE_CORRECTION section turns on either the approximate exchange kernel (AXK),^100^ or the second-order screened exchange (SOSEX) corrections to reduce the self-correlation errors of the dRPA method.^101^ The corrections are set up as shown below.
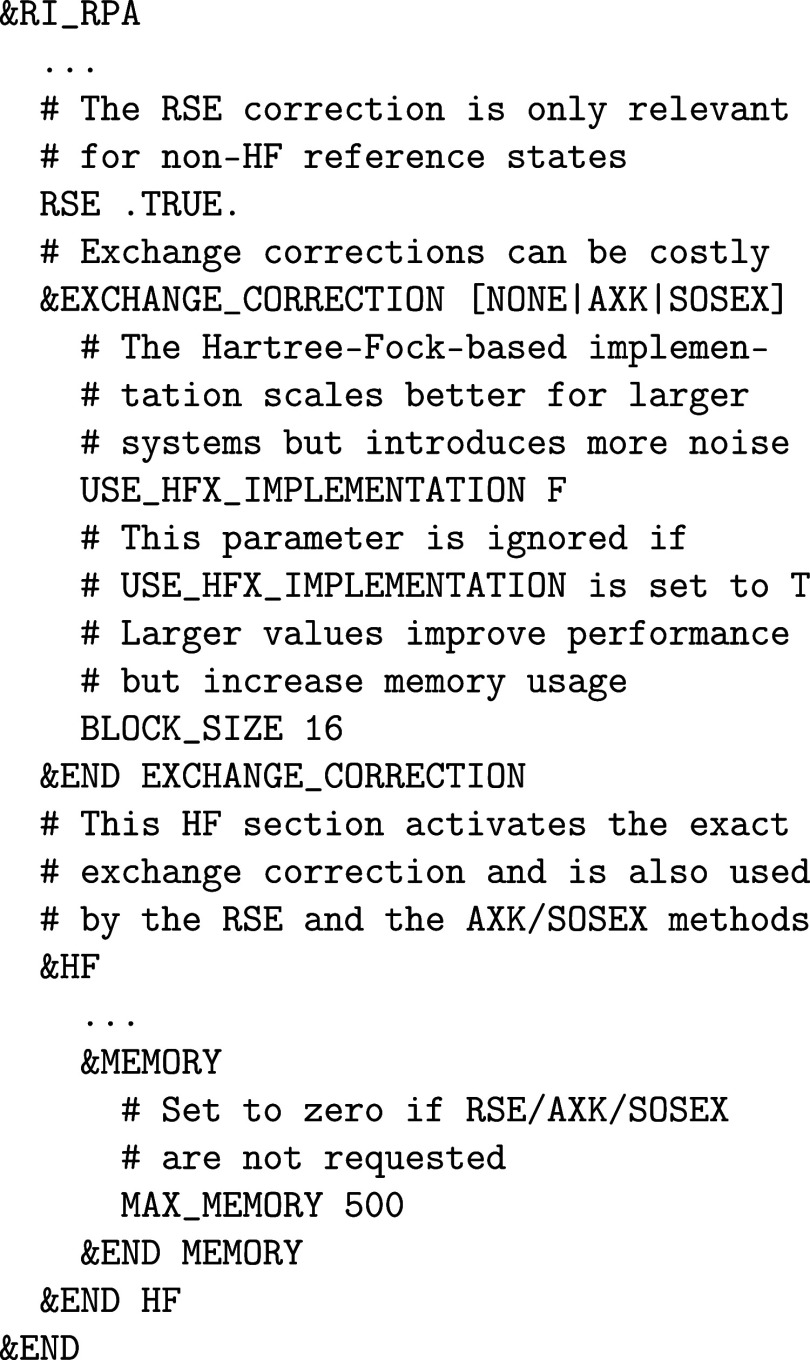


As is customary in CP2K, it is important to use the lone section name as a keyword to specify the actual method. The USE_HFX_IMPLEMENTATION keyword in the &RI_RPA%EXCHANGE_CORRECTION section switches between a better scaling HF-based implementation and an RI-MP2-based implementation. The first one should be preferred for larger systems, and the latter one should be preferred for smaller systems. Note that the HF-based implementation recalculates the integrals using the HF code. In the periodic case, this requires a truncated Coulomb potential, whereas the integrals for the RI methods can be calculated using the ordinary Coulomb potential. In case of the SOSEX-correction, this makes the implementation not self-correlation-free although the SOSEX method theoretically shows this property.

For the dRPA and SOS-MP2 implementations, CP2K implements analytical nuclear forces and stress tensors.^102^ The general setup is similar to RI-MP2 gradient calculations, but adds the MAX_PARALLEL_COMM keyword to the &WF_CORRELATION%CANONICAL_GRADIENTS section for RI-RPA gradient calculations. It determines the number of parallel communication channels for the nonblocking communication scheme. Larger numbers improve the performance, but increase the memory requirements. We find that a maximum value of 3 is sufficient. Note that analytical gradients are not available for the beyond-RPA methods.

The computationally most costly part of dRPA and SOS-MP2 calculations is the contraction of **B** to **Q**(ω). Because this step is necessary for each quadrature point, the number of quadrature points should be chosen as low as possible. Therefore, we apply the Minimax quadrature rule. It is also a convenient option for the beyond-RPA schemes. Because CP2K relies heavily on parallel matrix–matrix multiplications using ScaLAPACK, a well-optimized (and GPU accelerated) implementation of this library should be employed if possible. However, please consult Section 11 for more details on the support of various libraries within CP2K. Alternatively, CP2K can employ the communication-optimal COSMA library for even higher performance in exchange for increased memory usage.^103^ If CP2K was compiled with COSMA support, it is used automatically. All of this holds for all kinds of RPA- or SOS-MP2-based methods.

As with RI-MP2, both RI-RPA and SOS-MP2 rely on partial replication of the data via a NUM_INTEG_GROUPS keyword inside the corresponding sections. It is by default chosen automatically based on the available memory, but can be tuned manually. It must be a divisor of the number of MPI ranks and the number of quadrature points. A larger number of integration groups reduces the communication, but increases the memory demands. This feature is also available for analytic gradient calculations.

#### 2.3.3 Low-Scaling Post-Hartree–Fock

As already mentioned, post-HF methods suffer from the steep scaling of their computational cost, thus limiting their application to small and medium-size systems. Reworking the fundamental equations, using the RI approximation and exploiting the inherent sparsity of AOs, it is possible to reduce that scaling. In CP2K, low-scaling implementations of RI-dRPA and RI-SOS-MP2 are available, for both energies^104^ and nuclear forces.^78^ It is straightforward to transform a canonical RI-dRPA or RI-SOS-MP2 input file into a low-scaling one, simply by adding the following to the &WF_CORRELATION section:
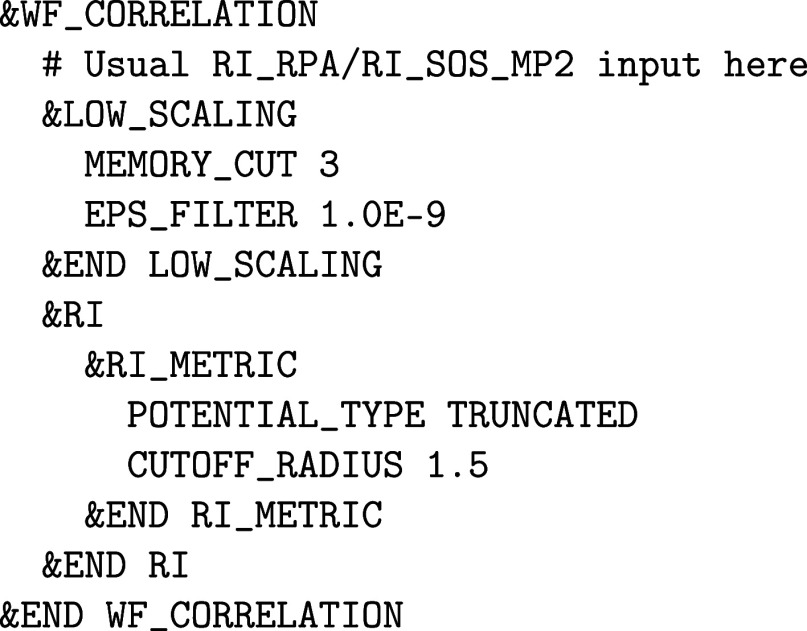


The input parameters specified in &RI_RPA or &RI_SOS_MP2 also apply in the low-scaling case. Note that the two subsections used in the above example are already discussed in Section 2.2.2 on the RI-HFX, since both methods used the same machinery and a similar theory. All performance aspects, such as the use of SORT_BASIS EXP or the choice of EPS_PGF_ORB also apply here. The &INTEGRALS subsection of a canonical post-HF calculation is also used in the low-scaling case, but only for 2-center integrals. The rest is calculated analytically with the Libint library.^105^

The nominal scaling of these methods is cubic. The exploitation of sparsity allows for a subcubic scaling in most cases. Note that energy-only calculations typically have a more advantageous scaling than nuclear force calculations, which still benefit from a cubic scaling upper bound. A lot of overhead is necessary to run such calculations, making them more expensive than their quartic scaling counterparts for small systems. The choice of the RI metric also adds an additional level of approximation. It is therefore always recommended to use the canonical versions of dRPA or SOS-MP2 whenever possible, and only use the low-scaling implementations for very large systems with hundreds of atoms. If in doubt, it might be worth simply trying both approaches.

### 2.4 Density Functional Theory plus Hubbard *U*

In spite of its success, standard local and semilocal DFT shows shortcomings in describing some physical phenomena such as London dispersion interactions or strong electron–electron correlation. The latter phenomenon is dominant in transition metals (TM), lanthanide and actinide oxides, such as NiO, CoO, CeO_2_ or UO_2_, resulting in a poor description of the electronic structure of these materials only using standard DFT. Anisimov et al. proposed to add a Hubbard *U* term as a correction to the standard DFT to improve the description of these materials.^106^ Since then, many variants of this kind of correction have been proposed. One of the most popular corrections, called DFT + *U*, was introduced by Dudarev et al.^107,108^ It is rotationally invariant, computationally inexpensive, and introduces only an effective Hubbard parameter *U*_eff_ = *U* – *J* as an atomic parameter for the atomic sites concerned. The Dudarev approach is implemented in CP2K/Quickstep.^58^ It is based on orbital occupations derived from a Mulliken,^109^ or Löwdin population analysis.^110^ DFT + *U* is globally activated with the keyword PLUS_U_METHOD in the &FORCE_EVAL%DFT section:
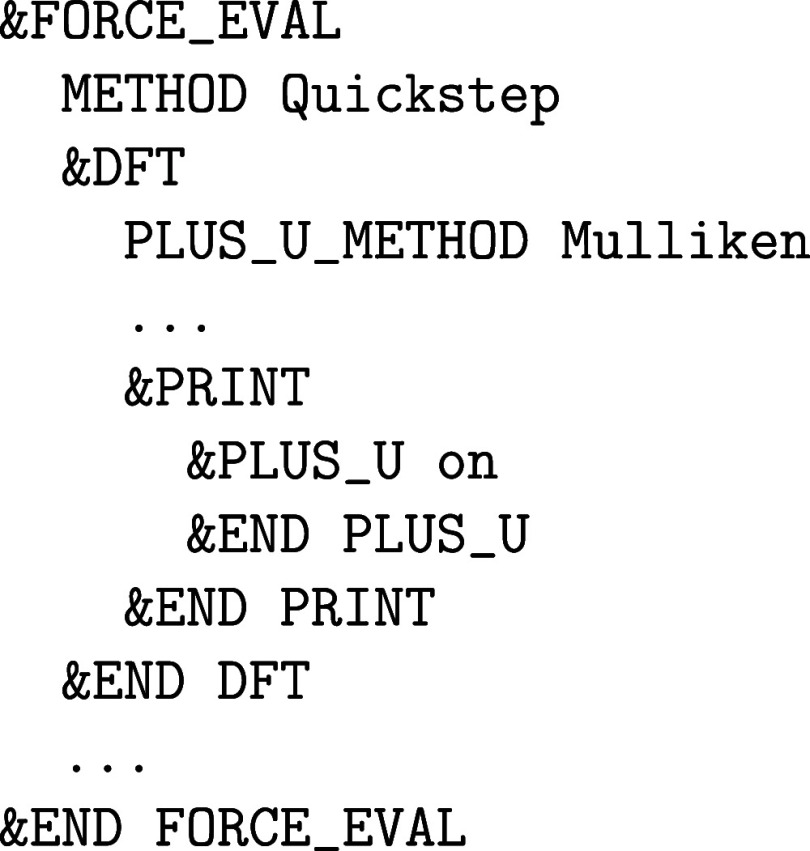
and the DFT + *U* specific printout is controlled via the corresponding &PRINT section within &PLUS_U. The *U*_eff_ parameter U_MINUS_J and the angular momentum number L must be defined in an atomic &KIND section. Here is an example that applies an effective Hubbard *U*_eff_ of 2 eV to the 5f orbitals (*l* = 3) of the uranium atoms assigned to the atomic kind *U*_a_:
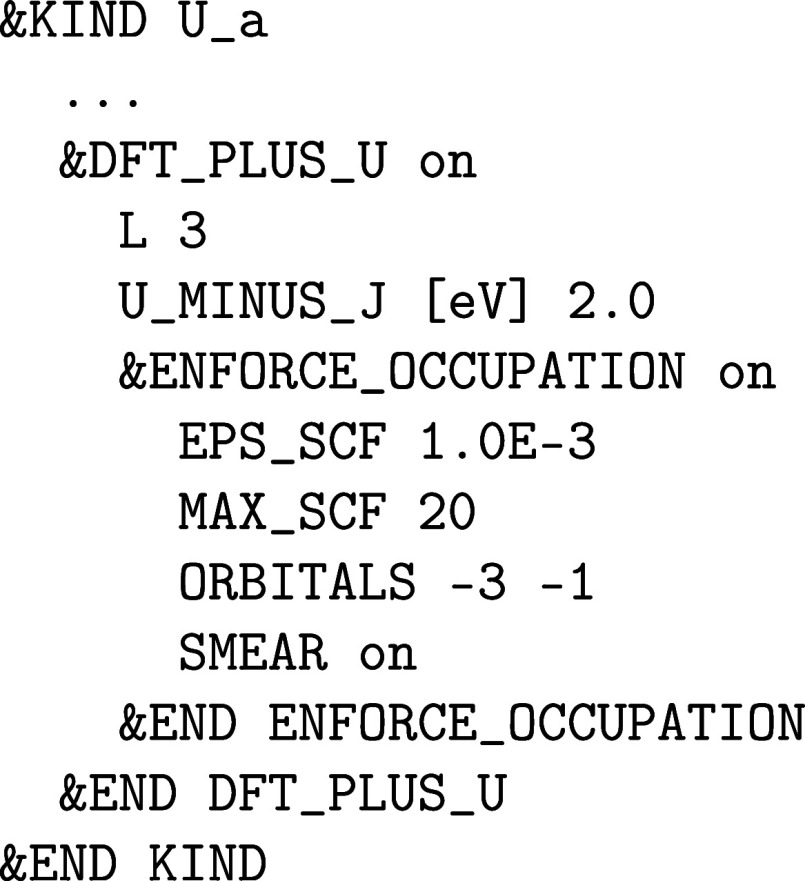


It is possible to enforce certain initial (desired) orbital occupations for a number of MAX_SCF SCF iteration steps or until a certain SCF convergence threshold EPS_SCF is reached as in the example above for the 5f orbitals of the atomic kind U_a_ in which the actual 5f electron density is smeared equally among the 5*f*_–3_ and 5*f*_–1_ orbitals. In this way, the desired orbital occupation patterns can be “enforced” when a straightforward convergence to the electronic ground state does not take place or is not expected due to the presence of metastable states,^57,58^ in which case an initial occupation matrix control (OMC) is required. After an initial SCF run (via RUN_TYPE energy) has been converged with &ENFORCE_OCCUPATION on, a SCF run without any OMC constraint (i.e., &ENFORCE_OCCUPATION off) can be restarted using the WF restart file of the initial constraint SCF run.

The “enforcement” is performed based on the initial orbital occupation of an atomic kind, which can be changed via the &KIND%BS section
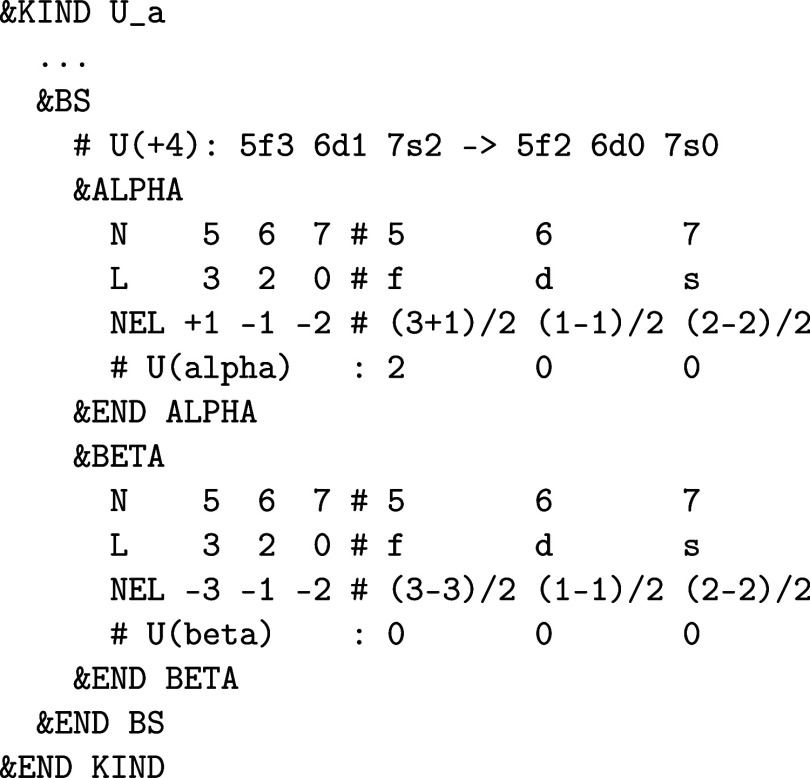
to create a spin-up on-site 5f^2^ triplet for a U^4+^ cation. For the corresponding spin-down 5f^2^ cation, the definitions in the sections &ALPHA and &BETA for spin-up and spin-down electrons have to be exchanged. The MULTIPLICITY in the &DFT section must be adapted if the number of spin-up and spin-down U atoms is not equal, which is needed for ferri- and ferromagnetic materials. The corresponding initial orbital occupation for the O^2–^ anion in UO_2_ can be specified with
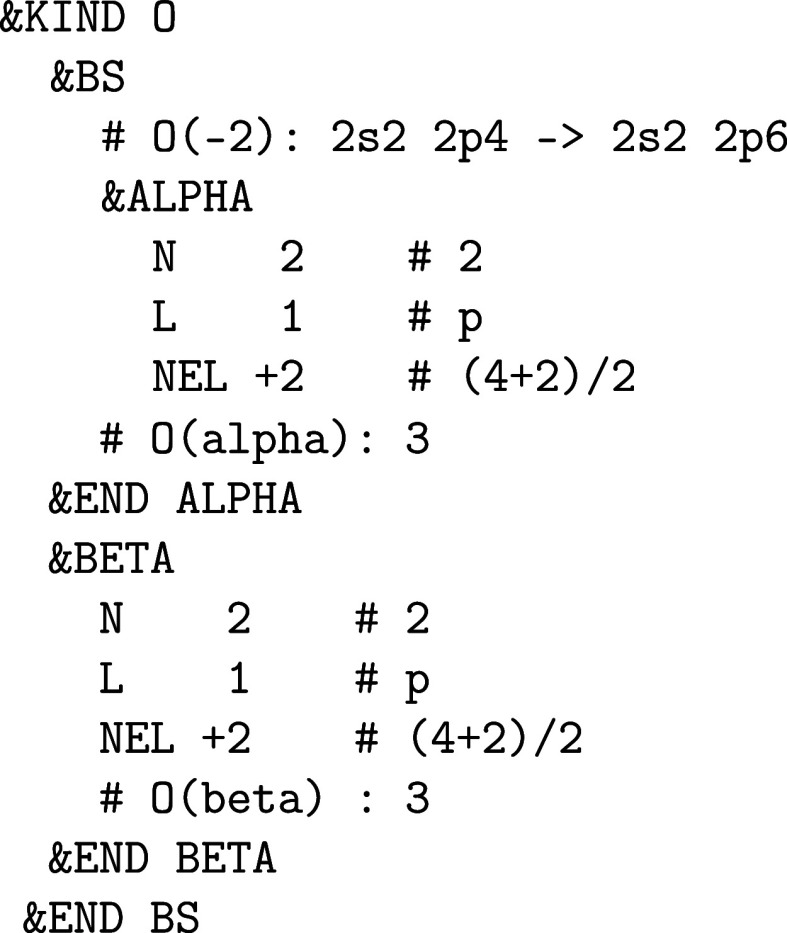


Atomic forces are only implemented for the PLUS_U_METHOD Mulliken and **k**-points are not available with DFT + U, yet.

## 3 Electronic Band Structure

Beside the CP2K/Quickstep code, more accurate electronic band structure calculations can be performed using the domain-specific electronic structure library SIRIUS, which implements the FP-LAPW and PP-PW methods,^18,20^ or the *GW* method.^21^

### 3.1 SIRIUS

With the interface to SIRIUS, CP2K has the ability to run FP-LAPW and PP ground state calculations, similar to Wien2k,^111^ FLEUR,^112^ ELK^113^ and exciting,^114^ to mention just a few.

SIRIUS supports:

- norm-conserving,^115^ ultrasoft,^116^ as well as PAW PPs
- APW^117^ and LAPW^20^ basis sets with an arbitrary number of local orbitals composed of up to third-order energy derivatives of radial functions
- evaluation of stress tensor and nuclear forces
- collinear and noncollinear magnetism
- symmetrization of lattice-periodic functions and on-site matrices
- generation of irreducible **k**-point meshes
- local-density approximation (LDA)^118^ and GGA flavours of XC potentials via the LibXC library^94^

Let us start with the example of PP-PW calculation setup using the second version of unified PP format (UPF) files, which are stored in XML format and can be read by SIRIUS directly. The example below demonstrates the input file for diamond:
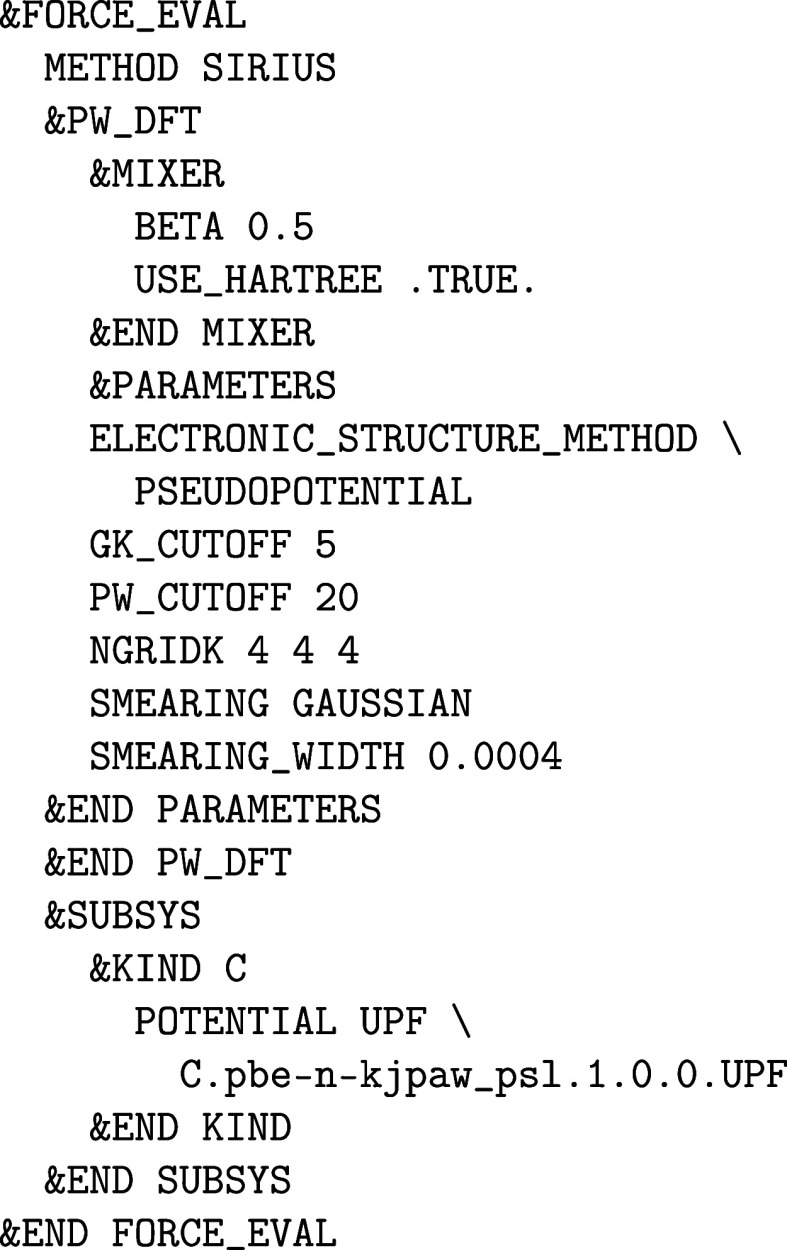


With a small change in parameters and atomic kind input sections, it is straightforward to switch to the FP-LAPW mode and compute the same structure with a high-accuracy full-potential method:
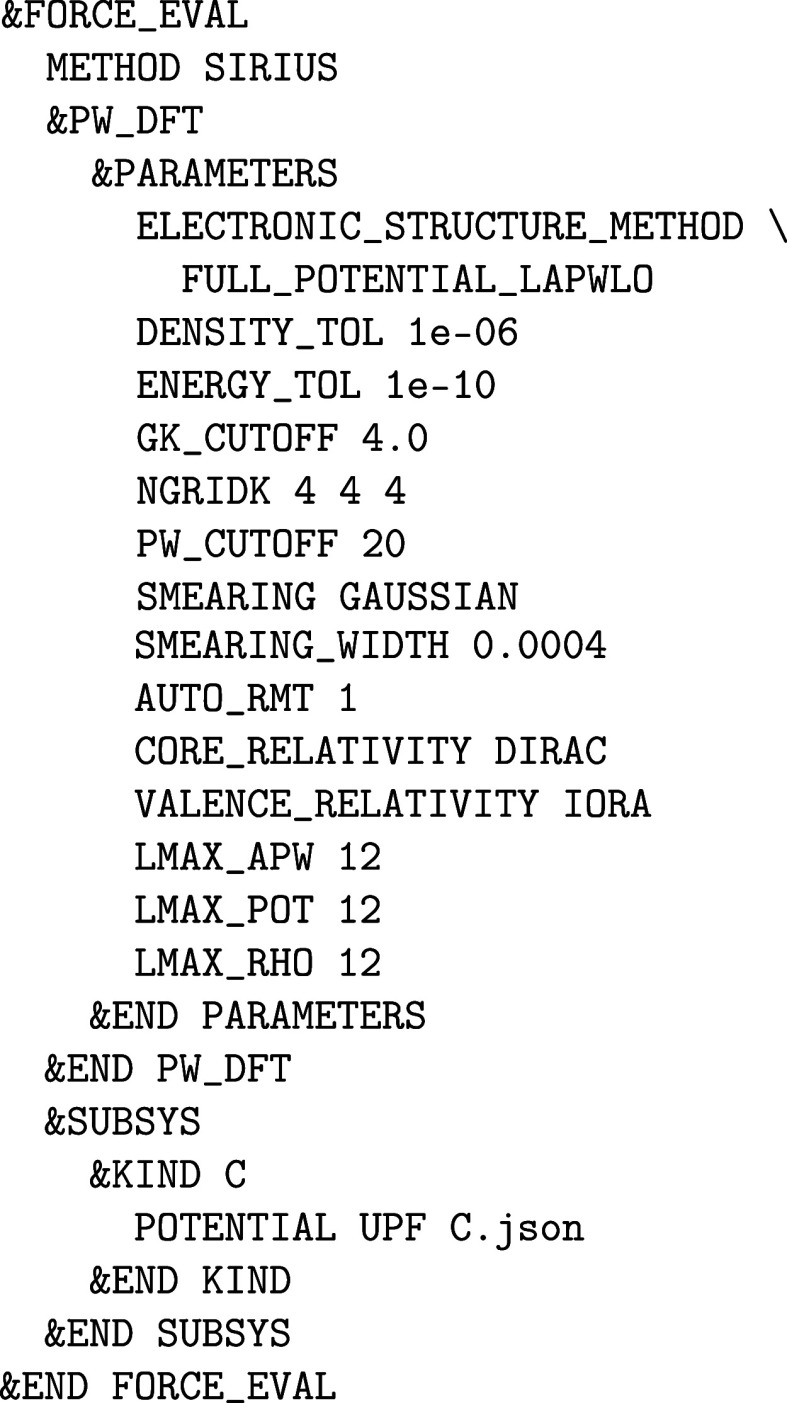


Full-potential atomic species JSON files for SIRIUS are available at https://github.com/electronic-structure/species/tree/master/FP/v1. They are similar to UPF files and contain information about the radial grid and LAPW+lo basis set. In the following, we provide an overview of the most relevant variables in the &PW_DFT%PARAMETERS section:

- ELECTRONIC_STRUCTURE_METHOD: type of calculation, i.e. FULL_POTENTIAL_LAPWLO, or PSEUDOPOTENTIAL.
- CORE_RELATIVITY: specifies if the core states in FP-LAPW are treated relativistically via the DIRAC equation or not.
- VALENCE_RELATIVITY: type of relativistic treatment of valence FP-LAPW radial basis functions and local orbitals. Possible values are NONE, KOELLING_HARMON, ZORA, or IORA.
- NUM_BANDS: number of ”bands” (KS states) to compute during Hamiltonian diagonalization.
- SMEARING: type of smearing used to compute the Fermi level, such as GAUSSIAN, COLD, FERMI_DIRAC, GAUSSIAN_SPLINE and METHFESEL_PAXTON.
- SMEARING_WIDTH: Width (in Ha) of the smearing function.
- PW_CUTOFF: PW cutoff (in a.u.^–1^) for expansion of electron density and potential.
- GK_CUTOFF: PW cutoff (in a.u.^–1^) for |**G + k**|.
- NUM_MAG_DIMS: number of magnetic dimensions in the system, i.e. 0—nonmagnetic calculations, 1—spin-collinear case, 3—noncollinear magnetic calculation.
- LMAX_APW: maximum orbital quantum number for LAPW basis functions.
- LMAX_RHO: maximum orbital quantum number for charge density expansion in muffin-tins (LAPW only).
- LMAX_POT: maximum orbital quantum number for KS potential expansion in muffin-tins (LAPW only).
- NGRIDK: dimensions of the **k**-point mesh. In other words, this is a division of the first Brillouin zone into microcells for **k**-point integration.
- ENERGY_TOL: SCF convergence tolerance of total energy.
- DENSITY_TOL: SCF convergence tolerance of charge density.
- GAMMA_POINT: Specifies if this is a Γ-point calculation (PP case only). NGRIDK must be set to 1 1 1 in that case.

Similarly, in section &PW_DFT%MIXER:

- TYPE: denotes the type of the density mixer, i.e. LINEAR, ANDERSON, ANDERSON_STABLE, or BROYDEN2.
- BETA: determines mixing parameter, which is a real value in the interval [0, 1].
- USE_HARTREE: logical variable that controls what is used as estimation of charge density difference. TRUE: Hartree energy of density residual $\frac{1}{2}\iint \frac{\Delta \rho \left(r\right)\Delta \rho \left(r^{\prime }\right)}{\left|{r-r}^{\prime }\right|}drdr^{\prime }$ is used as a measure of density convergence (works only for PP calculation). FALSE: normalized inner product of density residuals $\frac{1}{\Omega }\int \Delta \rho \left(r\right)\Delta \rho \left(r\right)dr$ is used as a measure of density convergence.

Computational aspects of CP2K/SIRIUS are configured in section &PW_DFT%CONTROL:

- MPI_GRID_DIMS: A 2-dimensional array that defines how the band parallelization is performed. The first dimension of the grid specifies the size of the FFT communicator used in the transformation of individual WFs. The second dimension defines the number of independent FFT communicator groups used to parallelize FFTs across different WFs. The product of the two dimensions defines the total number of MPI ranks used for the band parallelization. An orthogonal communicator will be built from the total available number of MPI ranks to perform **k**-point parallelization. Additionally, if the MPI grid is square, for example {3, 3}, a parallel eigensolver will be used to diagonalize the subspace Hamiltonian matrix.

### 3.2 The *GW* Method

The *GW* method is particularly well suited for high-accuracy computations of the electronic band structure of solids and MO energies of molecules.^119^ We first describe the band gap problem of DFT to motivate the usage of *GW*, before briefly discussing the theoretical framework of *GW* and our *GW* implementation within CP2K. We also provide input sections of *GW* calculations performed with CP2K.

#### 3.2.1 The Band Gap Problem of Density Functional Theory

When considering a solid under PBCs, the KS equations read as

28 $$\left(-\frac{\nabla^{2}}{2m}+v_{H}\left(r\right)+v_{XC}\left(r\right)\right)\;\psi_{nk}\left(r\right)=\varepsilon_{nk}^{DFT}\psi_{nk}\left(r\right)$$

where *v*_H_(**r**) is the Hartree potential and *v*_XC_(**r**) is the XC potential. These KS equations are solved to obtain the KS orbitals ψ_*n* **k**_(**r**) and the KS eigenvalues ε_*n* **k**_^DFT^ with band index *n* and crystal momentum **k**. For a molecule, the KS orbitals ψ_*n*_(**r**) and the KS eigenvalues ε_*n*_^DFT^ only carry a single quantum number, the MO index *n*. These KS eigenvalues ε_*n* **k**_^DFT^ are often used to approximate the electronic band structure of a solid. But, this approximation comes with limitations.

First, when using one of the common GGA XC functionals, the band gap in the KS-DFT band structure ε_*n* **k**_^DFT^ is much too small compared to experimental band gaps.^119^ Even with the exact XC functional, KS-DFT underestimates the fundamental gap due to the derivative discontinuity.^120^

Second, the GGA band structure ε_*n* **k**_^DFT^ is insensitive to screening by the environment. As an example, the GGA eigenvalue gap ε_LUMO_^DFT^ – ε_HOMO_^DFT^ between the lowest unoccupied MO (LUMO) and the highest occupied MO (HOMO) is almost identical for a molecule in the gas phase and a molecule on a surface. In experiment, however, the surface induces an image-charge effect, which can reduce this HOMO – LUMO gap of the molecule by several eV compared to the gas phase. In more general terms, this is a nonlocal screening effect by the surface that is absent in common approximate GGA XC functionals. A similar band gap reduction due to nonlocal screening is present when two materials are brought close to each other, for example, when two sheets of atomically thin materials are stacked on top of each other.

Third, one might use hybrid XC functionals to obtain band gaps from KS eigenvalues that align more closely with experimental values than GGA band gaps.^121–124^ However, the issue remains that nonlocal screening effects by the environment are not included in hybrid functionals. The above issues are known as the band gap problem of DFT.

#### 3.2.2 Theory of *GW* Band Structure Calculations

Green’s function theory offers a framework for calculating electron removal and addition energies, known as quasiparticle energies. Hedin’s equations provide an exact method for computing these quasiparticle energies within Green’s function theory.^51^ The *GW* approximation simplifies Hedin’s equations by approximating the self-energy Σ as the product of the Green’s function *G* and the screened Coulomb interaction *W*, i.e.

29 $$\Sigma^{GW}\left(r_{1},r_{2},t\right)=iG\left(r_{1},r_{2},t\right)W\left(r_{1},r_{2},t\right)$$

The advantage of *GW* is that it captures nonlocal screening effects on the electronic band structure, as previously discussed, and that band gaps computed by *GW* are often in excellent agreement with experiments.

*GW* calculations in CP2K start from a KS-DFT calculation, i.e. we assume that the above KS equations have been solved. In the *G*_0_ *W*_0_ approach, we use KS orbitals and their KS eigenvalues to compute the Green’s function *G*_0_ of noninteracting electrons and the screened Coulomb interaction *W*_0_ in the RPA. The *G*_0_ *W*_0_ self-energy $\Sigma^{G_{0}W_{0}}\left(t\right)$ is then obtained by replacing *G* → *G*_0_ and *W* → *W*_0_ in eq 29, followed by a Fourier transform from time *t* to frequency (or energy) *ε*, yielding $\Sigma^{G_{0}W_{0}}\left(\varepsilon \right)$ . In the *G*_0_ *W*_0_ method we further approximate that KS orbitals are the quasiparticle WFs. Then, the *G*_0_ *W*_0_ quasiparticle energies are

30 $$\varepsilon_{nk}^{G_{0}W_{0}}=\varepsilon_{nk}^{DFT}+\left\langle \psi_{nk}\left|\sum^{G_{0}W_{0}}\left(\varepsilon_{nk}^{G_{0}W_{0}}\right)-v_{XC}\right|\psi_{nk}\right\rangle$$

We might interpret this as removing the spurious XC contribution of DFT, i.e. ⟨ψ_*n* **k**_|*v*_XC_|ψ_*n* **k**_⟩, from the DFT eigenvalue *ε*_*n* **k**_^DFT^ and adding the XC contribution from *G*_0_ *W*_0_, i.e. ⟨*ψ*_*n* **k**_|Σ^*G*_0_ *W*_0_^(*ε*_*n* **k**_^*G*_0_ *W*_0_^)|*ψ*_*n* **k**_⟩.

CP2K also allows performing eigenvalue self-consistency in *G* (ev*GW*_0_) and eigenvalue self-consistency in *G* and in *W* (ev*GW*), where especially the *G*_0_ *W*_0_ quasiparticle energies can be strongly influenced by the DFT XC functional. In general, we recommend using ev*GW*_0_, starting from the PBE functional,^35^ for both molecules and solids, motivated by the discussion in ref 125.

CP2K contains three different *GW* implementations: *GW* for molecules,^104^ *GW* for computing the band structure of a solid with a small unit cell with **k**-point sampling in DFT,^126^ and *GW* for computing the band structure of a large cell in a Γ-only approach.^127^ In the following, we will discuss the details and usage of all of these *GW* implementations.

#### 3.2.3 *GW* for Molecules

To start a *G*_0_ *W*_0_, ev*GW*_0_ or ev*GW* calculation for a molecule, one needs to set the keyword RUN_TYPE ENERGY in the &GLOBAL section and the following segment:
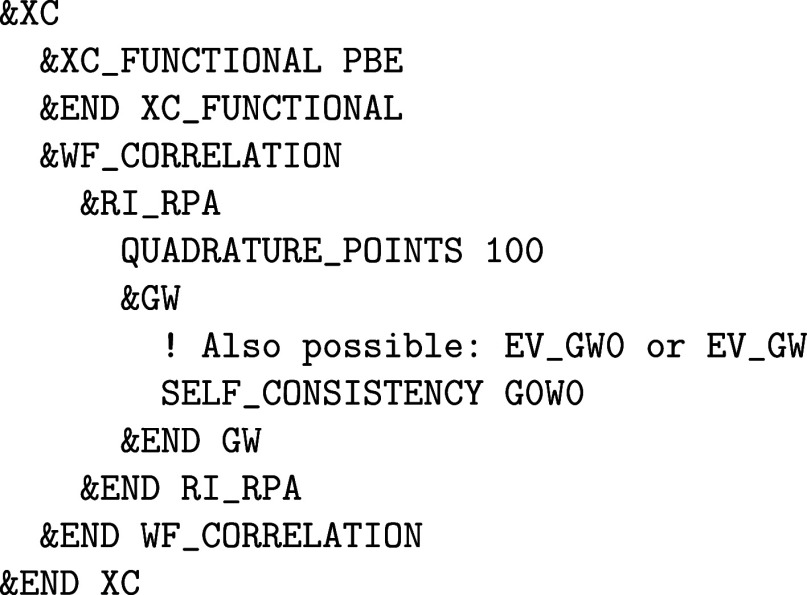


Therein, the following keywords have been used:

- QUADRATURE_POINTS: number of imaginary-frequency points for computing the self-energy, see eq 21 in ref 104. Usually, 100 points converge the quasiparticle energies within 10 meV.
- SELF_CONSISTENCY: determines which *GW* self-consistency variant (*G*_0_ *W*_0_, ev*GW*_0_ or ev*GW*) is used to calculate the *GW* quasiparticle energies.

The numerical precision of the *GW* implementation is 10 meV compared to reference calculations, for example, on the *GW*100 test set.^125,128^ Furthermore, the following DFT settings will also have an influence on *GW* quasiparticle energies:

- XC_FUNCTIONAL: starting XC functional for the *G*_0_ *W*_0_, ev*GW*_0_ or ev*GW* calculation, respectively. We recommend to use ev*GW*_0_@PBE, as discussed in ref 125. For further guidance on selecting an appropriate DFT starting functional and self-consistency scheme for your system, you may consult ref 119.
- BASIS_SET: the basis set is of Gaussian type and can have a strong influence on the quasiparticle energies. For computing quasiparticle energies, a basis set extrapolation is necessary,^104^ and we recommend all-electron GAPW calculations with correlation-consistent basis sets cc-pVDZ, cc-pVTZ, cc-pVQZ from the EMSL Basis Set Exchange.^44^ For computing the HOMO – LUMO gap from *GW*, we recommend the usage of augmented basis sets, for example aug-cc-pVDZ and aug-cc-pVTZ. As RI_AUX basis set, we recommend the RIFIT basis sets from the EMSL database, for example aug-cc-pVDZ-RIFIT.

The computational effort of the *GW* calculation increases with $O\left(N^{4}\right)$ , where *N* is the system size. The memory requirement increases with $O\left(N^{3}\right)$ . For large-scale calculations, we recommend starting with a small molecule. After successfully completing the *GW* calculation for the small molecule, you can gradually increase the size of the molecule. The computational resources needed for larger molecules can then be estimated using the $O\left(N^{4}\right)$ scaling for computation time and $O\left(N^{3}\right)$ scaling for memory. The output provides a useful lower limit of the required memory, e.g.
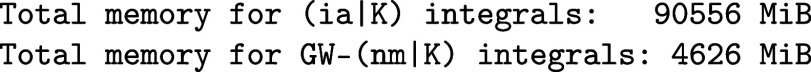


When facing an out-of-memory error, please increase the number of nodes for your calculation.

#### 3.2.4 *GW* for Small Unit Cells with **k**-Point Sampling

For a periodic *GW* calculation, **k**-point sampling is required. In the following example of a 2D-periodic cell, **k**-point sampling is included in the DFT section:
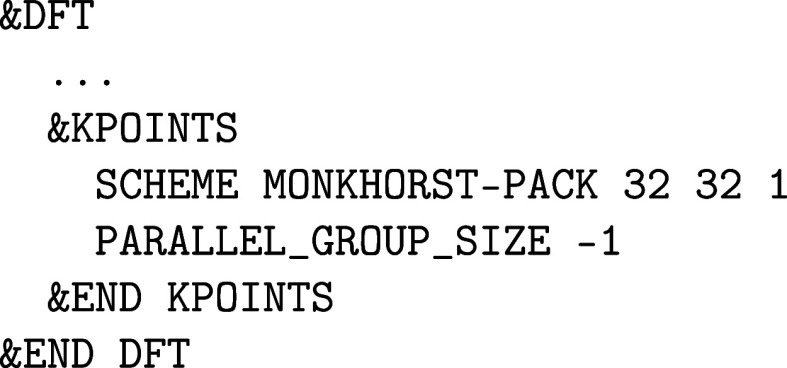


The **k**-point mesh size is a convergence parameter, and 32 × 32 is expected to reach convergence of the *GW* band gap within 10 meV for a 2D material.

A periodic *GW* calculation is activated via the &BANDSTRUCTURE section, e.g.
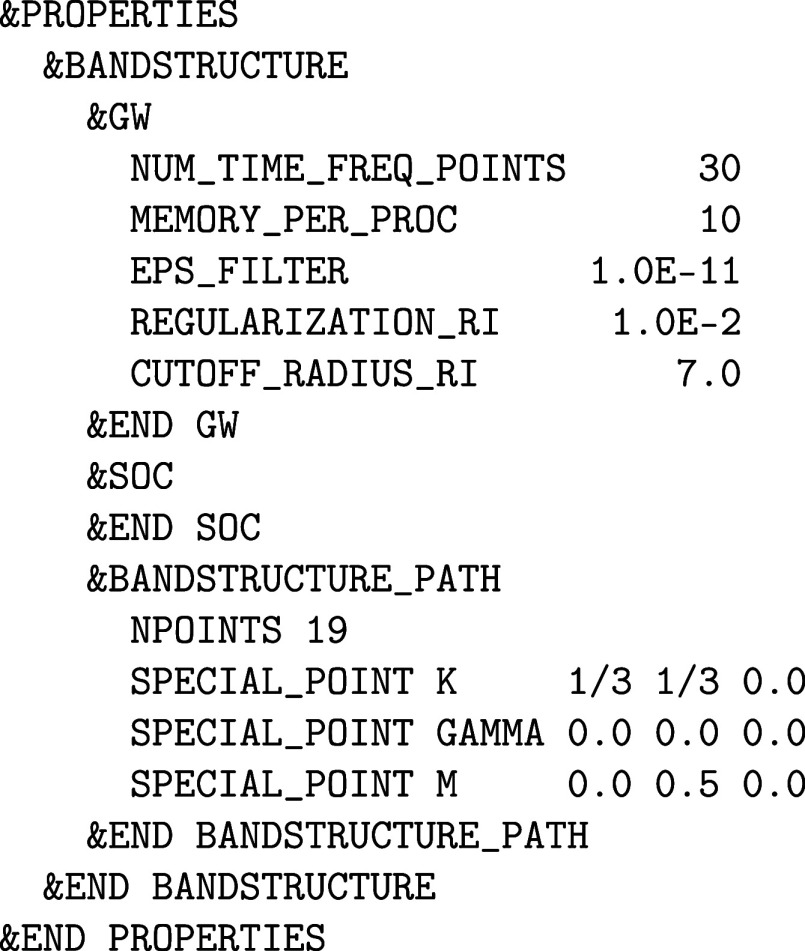


All parameters from above have been chosen to converge the *GW* band gap within 10 meV, see also convergence tests in ref 126:

- NUM_TIME_FREQ_POINTS: number of imaginary-time and imaginary-frequency points used for computing the self-energy. Between 20 and 30 points are usually enough for converging quasiparticle energies within 10 meV. Grids up to 34 points are available.
- MEMORY_PER_PROC: specifies the available memory per MPI process. A larger MEMORY_PER_PROC can increase performance.
- EPS_FILTER: filter for three-center integrals, 10^–11^ should be well-converged.
- REGULARIZATION_RI: regularization parameter for RI basis set. For a big RI basis set (>50 RI function per atom), we recommend 10^–2^ to prevent linear dependencies. For a small RI basis set, one can turn RI regularization off by setting 0.0.
- CUTOFF_RADIUS_RI: cutoff radius of truncated Coulomb metric in Å. A larger cutoff leads to faster RI basis set convergence, but also the computational cost increases. A cutoff of 7 Å is an accurate choice.
- &SOC: activates spin–orbit coupling (SOC) from GTH PPs.^32^ But, the usage of SOC also needs POTENTIAL_FILE_NAME GTH_SOC_POTENTIALS.
- &BANDSTRUCTURE_PATH: specify the **k**-path in the Brillouin zone for computing the band structure. Relative **k**-coordinates are needed, which you can retrieve for your crystal structure from ref 129.

We recommend the TZVP-MOLOPT basis sets together with GTH PPs.^126^ At present, 2D PBCs are supported, while 1D- and 3D PBCs are work in progress.

The *GW* band structure is written to the files bandstructure_SCF_and_G0W0 and bandstructure_SCF_and_G0W0_plus_SOC, respectively. The direct and indirect band gaps are also listed in the CP2K output file. When facing an out-of-memory crash, please increase MEMORY_PER_PROC.

#### 3.2.5 *GW* for Large Cells in Γ-Only Approach

For a large unit cell, a Γ-only *GW* algorithm is available in CP2K. The requirement on the cell is that elements of the density matrix decay by several orders of magnitude when the two basis functions of the matrix element have a distance similar to the cell size. As a rule of thumb, for a 2D material, a 9 × 9 unit cell is large enough for the Γ-only algorithm.^127^

The input file for a Γ-only *GW* calculation is identical to that of *GW* for small cells with **k**-point sampling except that the &KPOINTS section in DFT needs to be removed. The computational parameters from such an input file reach a numerical convergence of the band gap within ∼50 meV (TZVP basis set, 10 time and frequency points).^127^ The code prints restart files with ending .matrix that can be used to restart an interrupted calculation.

## 4 Embedding Methods

In this section, multiscale methods that permit embedding part of a system, which is described using a quantum mechanical electronic structure method, into a surrounding environment at a lower level of theory, are presented and discussed in detail. However, instead of the simple mechanical coupling approach, which can be easily implemented using the &FORCE_EVAL%MIXED framework^130^ and adaptive resolution simulation methods,^131–133^ the focus here will be on more sophisticated methods including nontrivial interactions with the environment.

### 4.1 Implicit Solvation Methods

The simulation of a solute like a molecule or a cluster with an explicit solvent captures all the details of the solute–solvent interaction, as well as the dynamics and fluctuations within the solvent. However, often embedding the solute within an implicit solvent is sufficient, especially when only an approximate (averaged) inclusion of polarization effects by dielectric screening is required. Representing the solvent as a continuous dielectric medium significantly reduces the number of degrees of freedom and avoids sampling of potentially uninteresting fluctuations within the solvent, which can also lead to great computational time savings. Moreover, the preparation and equilibration of a solvated system in a simulation box becomes also unnecessary. A large number of continuum solvation models can be found in the literature,^134^ but these models usually employ a rigid cavity for the solute, introducing a discontinuity in the dielectric function at the vacuum–cavity interface. Such discontinuities cause kinks in the atomic forces which are detrimental in structure relaxations and AIMD simulations. Fattebert and Gygi proposed a smooth dielectric function based on the electron density to palliate the problem.^135,136^ In their approach, the response to the electronic density change is self-consistently taken into account in each SCF iteration step, introducing a nested SCF loop within each SCF WF optimization step, which is the price to pay for getting rid of the explicit solvent molecules. This self-consistent continuum solvation (SCCS) model of Fattebert–Gygi and its revised form of Andreussi et al.,^137^ are both implemented in CP2K.^138,139^ The SCCS input parameters are defined in the &FORCE_EVAL%DFT%SCCS section. An SCCS input snippet for a molecule immersed in water (ε_0_ = 78.36) looks like:
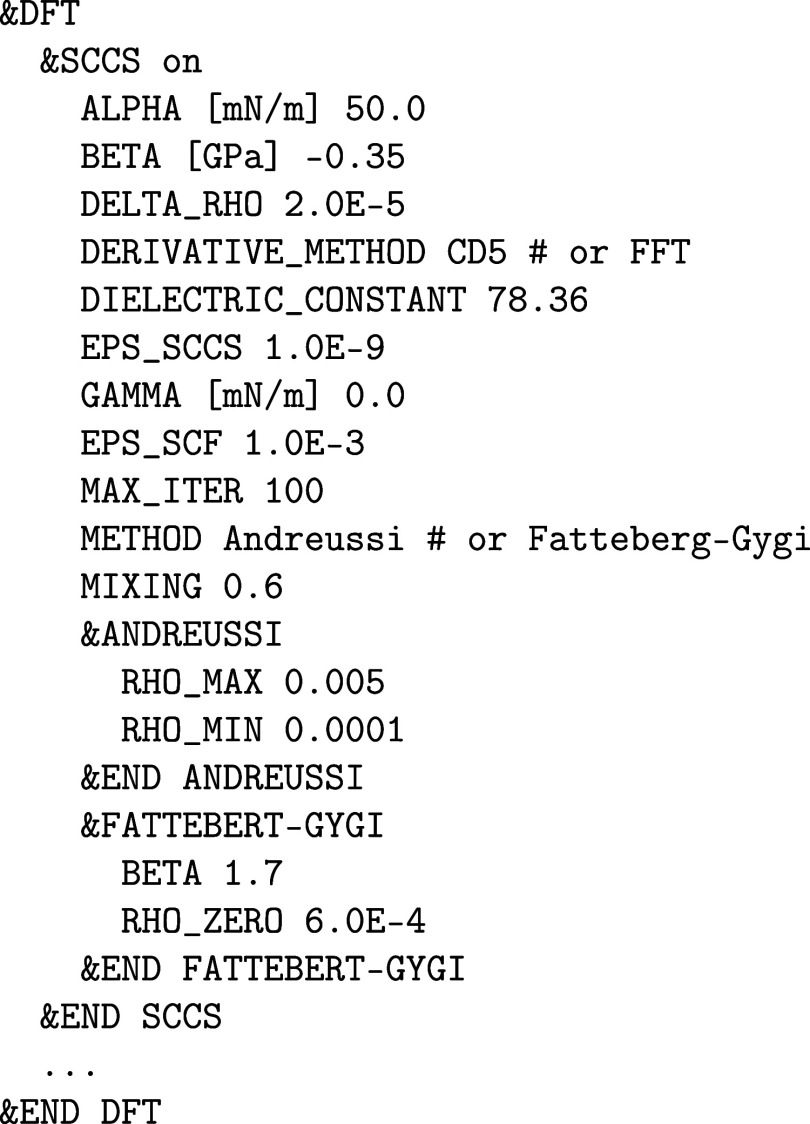


The values for the keywords RHO_MAX and RHO_MIN determine the smoothing of the dielectric function for the revised SCCS method of Andreussi et al.^137^ Likewise, BETA and RHO_ZERO are the smoothing parameters for the original SCCS method of Fattebert and Gygi.^135^ They are solute dependent, and especially charged solutes require different values for the revised SCCS model.^140^ While for neutral solutes 0D, 2D, 1D, and 3D PBCs can be applied, charged systems should be run with 0D PBC using a Poisson solver like MT:^141^ 
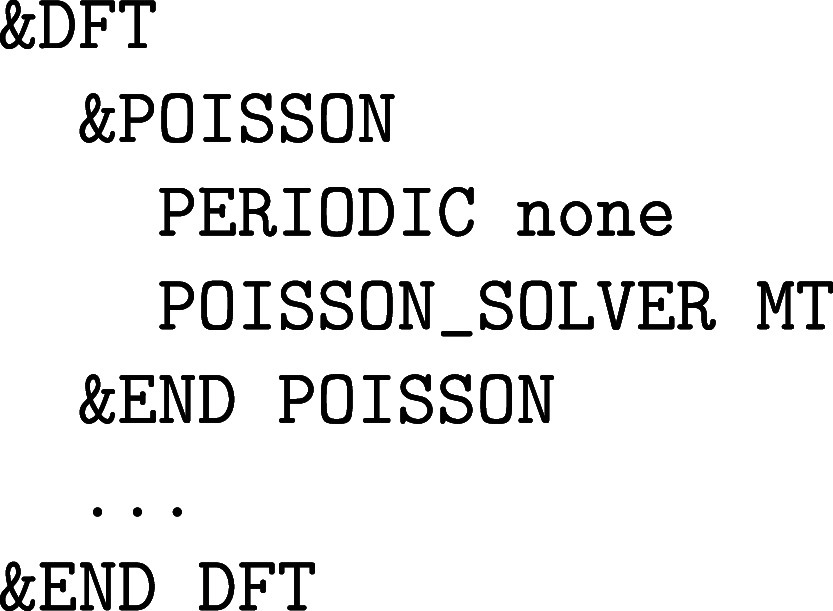
while keeping the periodicity defined in the &CELL section identical, i.e.
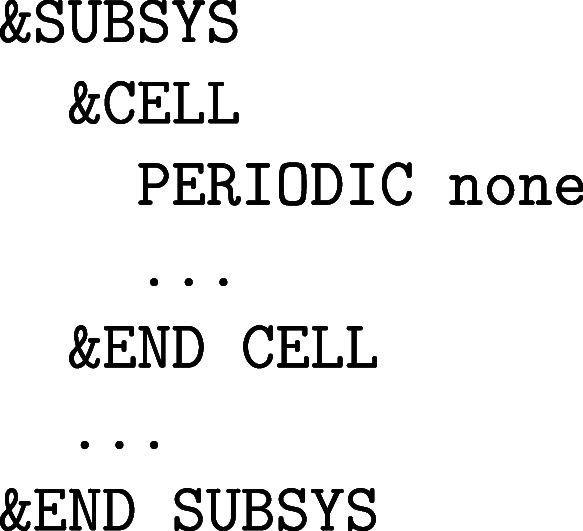


The first WF optimization steps are performed without SCCS. The SCCS input parameter EPS_SCF determines the SCF convergence threshold for the onset of the nested (inner) self-consistent iteration of the polarization potential, and EPS_SCCS defines the corresponding convergence threshold for the requested numerical accuracy, performing a maximum of MAX_ITER SCCS iteration steps.

The keywords ALPHA, BETA, and GAMMA are optional parameters for additional solvation model terms besides the electrostatic contribution Δ*G* ^el^ defining the solvation free energy

31 $$\Delta G^{sol}=\Delta G^{el}+G^{cav}+G^{rep}+G^{dis}$$

with a cavitation term

32 $$G^{cav}=\gamma S$$

which can be computed based on the “quantum surface” introduced by Cococcioni et al.^142^ The latter reads as

33 $$S=\int \left\{\vartheta_{\rho_{0}-\Delta /2}\left[\rho^{elec}\left(r\right)\right]-\vartheta_{\rho_{0}+\Delta /2}\left[\rho^{elec}\left(r\right)\right]\right\}\times \frac{\left|\nabla \rho^{elec}\right|}{\Delta }dr$$

where Δ and γ are defined by the input parameters DELTA_RHO and GAMMA, respectively.

The terms accounting for the Pauli repulsion *G* ^rep^ and the dispersion interactions *G* ^dis^ can be computed based on the surface *S* and the volume *V* of the solute cavity, i.e.

34 $$G^{rep}+G^{dis}=\alpha S+\beta V$$

The prefactors ALPHA and BETA are solvent-specific tunable parameters. This ultimately allows the calculation of a solvation free energy

35 $$\Delta G^{sol}=\Delta G^{el}+\left(\alpha +\gamma \right)S+\beta V$$

Finally, the SCCS printout is controlled by the input section FORCE_EVAL%DFT%PRINT%SCCS:
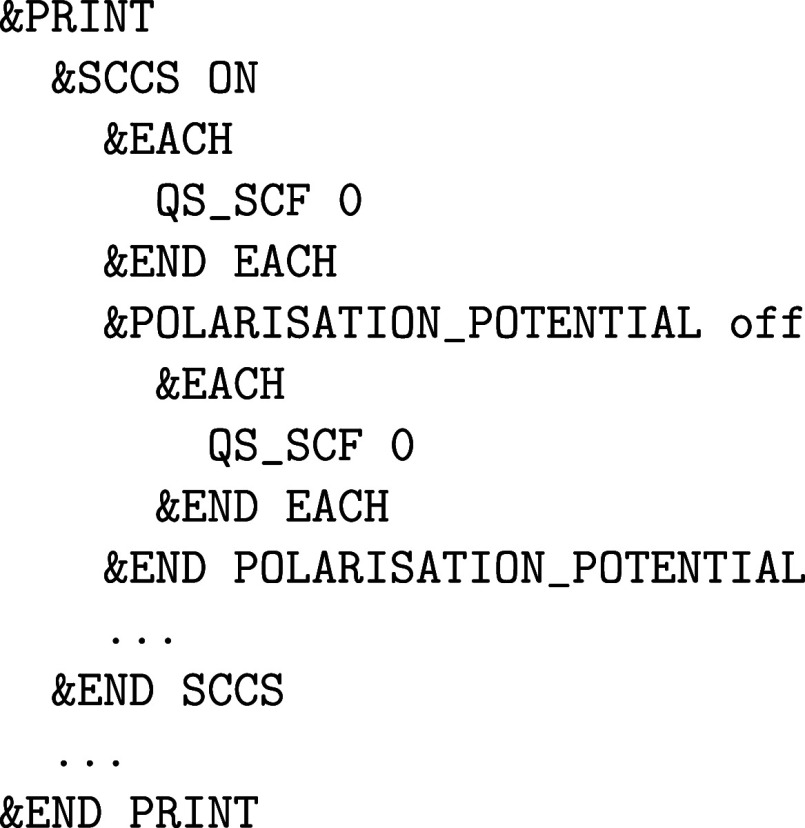
allowing for printing the polarization potential and the dielectric function in cube file format.

### 4.2 Quantum Mechanics/Molecular Mechanics Methods

In quantum mechanics/molecular mechanics (QM/MM) hybrid schemes,^143^ the system is divided into two subsystems: one is treated at the quantum mechanical (QM) level of theory and the other at the molecular mechanics (MM) level. In CP2K, an additive QM/MM scheme is implemented, which partitions the QM/MM total energy *E*_totQM/_^MM^ into the following three terms

36 $$E_{tot}\left(R_{\alpha },R_{a}\right)=E_{QM}\left(R_{\alpha }\right)+E_{MM}\left(R_{a}\right)+E_{QM/MM}\left(R_{\alpha },R_{a}\right)$$

where **R**_α_ and **R**_*a*_ label the coordinates of the QM and MM nuclei, respectively. The QM contribution is evaluated using the Quickstep module,^23^ typically employing KS-DFT. Alternatively, SQC methods, such as PM6,^144^ self-consistent-charge density-functional tight-binding (SCC-DFTB),^145^ or extended tight-binding (xTB) approaches can be used.^146^ MM contributions are obtained from classical force fields, either using the built-in Fist program, or by an interface to Gromacs.^147,148^

The QM/MM energy includes nonbonded terms and, if the QM system is connected to the MM region by covalent bonds, bonded terms as well. Nonbonded terms comprise van der Waals (vdW) and electrostatic (elec) interactions, i.e.

37 EQM/MM=EQM/MMbonded+EQM/MMnon‐bonded=EQM/MMbonded+EQM/MMvdw+EQM/MMelec.

Speaking about bonded terms, special care has to be taken with respect to unsaturated bonds that are occurring when cutting bonds of covalently coupled subsystems. For that purpose, CP2K offers two general options. The simplest approach is to saturate the dangling bond of the QM atom with a monovalent atom (link atom), typically a hydrogen atom. The QM calculations are then performed on the QM atoms plus the link atom. The interaction between the covalently bound QM and MM atom is modeled with classical MM potentials. This approach is often termed the link-atom technique in the literature and referred to as the integrated MO molecular mechanics (IMOMM) method in CP2K.^149^ Alternatively, a linking atom with a monovalent PP can replace the MM frontier atom at the QM/MM boundary.^149–152^

Turning to the nonbonded terms, the vdW interactions between QM and MM atoms are often described using a simple Lennard-Jones potential.^149^ The treatment of electrostatic interactions, however, is the most challenging aspect of the QM/MM Hamiltonian and can be approached with varying levels of complexity.^149^ Generally, we distinguish between three different approaches, which are all available in CP2K. The simplest approach is a mechanical embedding scheme, in which precalculated partial atomic charges are assigned to both QM and MM atoms. The charge–charge interactions are then computed using classical expressions, neglecting the response of the QM charge density to the electrostatic potential generated by the MM charges. The most popular scheme, and the default treatment in CP2K, is electrostatic embedding.^153,154^ In this approach, an additional term is added to the QM Hamiltonian, allowing the MM charges to polarize the QM region. For QM calculations at the KS-DFT level with GPW and GAPW, CP2K implements a particularly efficient variant of electrostatic embedding called Gaussian expansion of the electrostatic potential (GEEP), which is explained in more detail below. The next level of sophistication is polarized embedding schemes, where the QM atoms can polarize the QM part nonself-consistently or fully self-consistently. CP2K incorporates polarized embedding schemes to some extent. For example, it offers a fully self-consistent image-charge augmented QM/MM (IC-QM/MM) scheme,^155^ specifically suited for adsorbate/metal systems. A nonself-consistent core–shell model, which can be applied to a broad range of systems, is also available.^156^

A minimal example of a QM/MM calculation in a periodic cubic simulation box of length 25 Å, where atoms with indices 1 to 6 are described at the KS-DFT level, looks as follows:
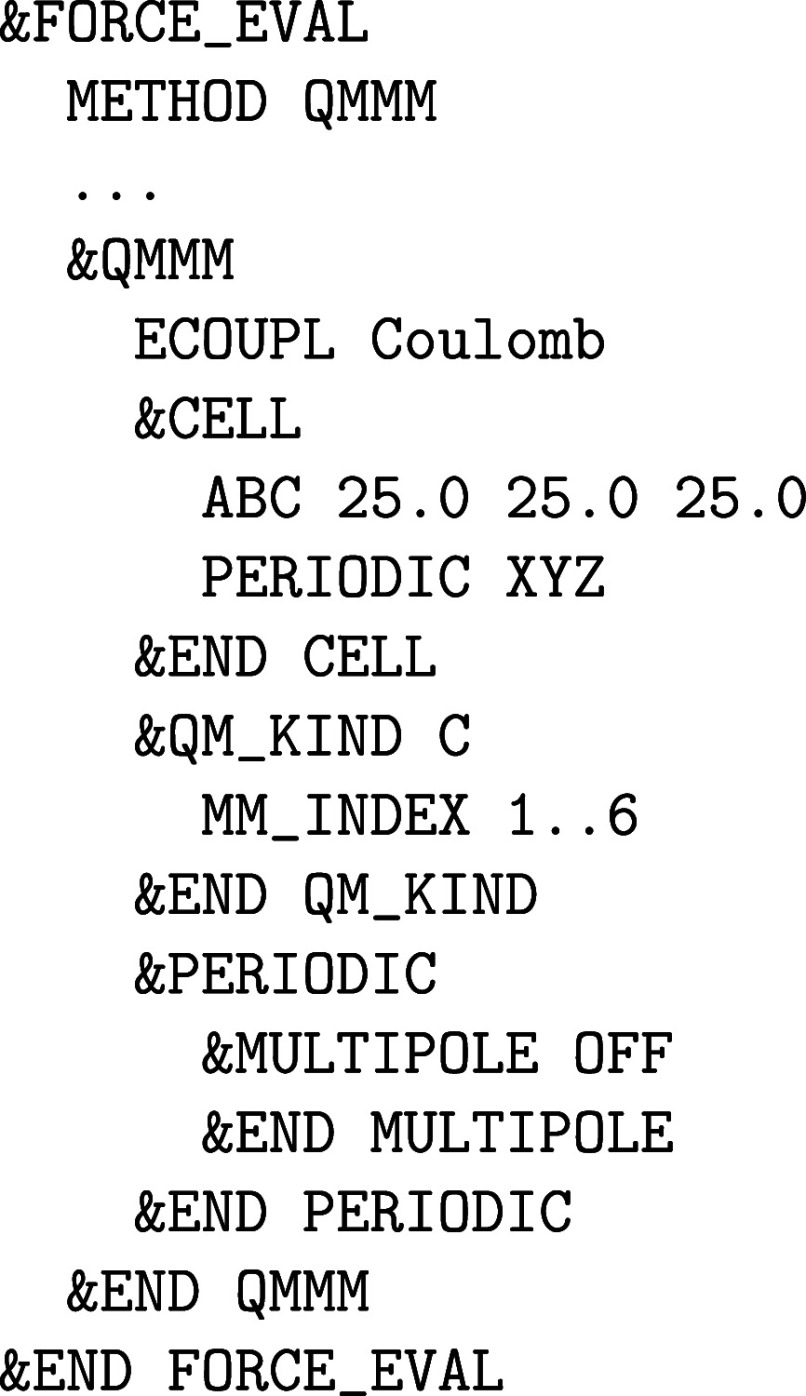


In addition to that, the DFT section must be defined for the QM part of the calculation, as well as the MM section with all classical force field parameters, including the classical interactions between the QM and MM subsystems, such as the vdW interactions. The relevant keywords and subsections within the &QMMM section are

- ECOUPL: defines the type of electrostatic treatment. On the one hand GAUSS enables the electrostatic embedding for KS-DFT/MM simulations based on GEEP (see next section). On the other hand COULOMB enables electrostatic embedding for SQC methods, or SCC-DFTB/xTB. Mechanical embedding schemes can be used by setting the keyword to NONE.
- CELL: defines the cell for the QM calculation, which must be orthorhombic.
- QM_KIND: defines the QM system and the corresponding indices are given for each atomic type separately.
- PERIODIC: applies the periodic potential. The MULTIPOLE section turns on the coupling/recoupling of the QM periodic images, as described later. In the previous example, it was turned off because the QM box and the MM box are identical in size.

In our simple example, no covalent bonds are cut through the subsystem boundaries. For cases where covalent bonds are cut, the corresponding techniques and link atoms can be set through the &LINK section.

#### 4.2.1 Electrostatic Embedding by the Gaussian Expansion of the Electrostatic Potential Method

The calculation of electrostatics terms in QM-MM poses problems related to both short-range and long-range behavior. The problems of short-range behavior are related to the electron spill-out. Classical atoms are normally represented by a simple point charge. If a QM atom comes close, the electrons can be trapped into the point-like classical potential energy source. The use of diffuse basis sets (or PWs) can enhance this behavior. The ad-hoc generated pseudopotential-like approach has often been applied to remedy the unphysical problem of overpolarization that arises when MM atoms are in close contact with the QM region. This approach requires the definition of frontier MM and frontier QM atoms, which could result in being cumbersome and not sufficiently adaptable to the dynamic evolution of the system. In CP2K, the spill-out is avoided by assigning to all MM atoms a finite-width charge density in the form of a Gaussian distribution

38 $$n\left(r,R_{MM}\right)={\left(\frac{r_{c,MM}}{\sqrt{\pi }}\right)}^{3}e^{-{\left(\left|r-R_{MM}\right|/r_{c,MM}\right)}^{2}}$$

The width of the charge density depends on the atom type and can be expected to be similar to the covalent radius. The exact potential originating from the Gaussian charge distribution, as commonly employed in the Ewald method, is

39 $$v_{MM}\left(r,R_{MM}\right)=\frac{\mathrm{Erf}\left(\frac{\left|r-R_{MM}\right|}{r_{c,MM}}\right)}{\left|r-R_{MM}\right|}$$

This potential tends to 1/*r* at large distances and converges smoothly to a constant at zero. The electrostatic interaction between the QM and the MM atoms can then be computed onto the QM grid by multiplying the QM charge and this potential. For a more efficient calculation, though, the MM electrostatic potential is decomposed in a series of *N*_g_ Gaussians of different cutoffs plus a residual, which is why we call this algorithm GEEP.^153^ Hence

40 $$\frac{\mathrm{Erf}\left(\frac{r}{r_{c}}\right)}{r}=\sum_{N_{g}}A_{g}e^{-{\left(r/G_{g}\right)}^{2}}+R_{low}\left(r\right)$$

In this expression, *A*_g_ is the Gaussian amplitude, *G*_g_ its width, and *R*_low_ is the smooth residual function that can be mapped onto a grid with spacing 1 order of magnitude larger than the one needed for the potential. The advantage of adopting the Gaussian expansion is that commensurate grids with different spacing can be used for the different contributions, i.e. sharper Gaussians to finer grids and coarser grids for smoother components. All contributions are finally interpolated onto the finest QM grid, by means of real-space splines, and summed up. The interpolation depends only on the number of grid points and not on the number of MM atoms, so
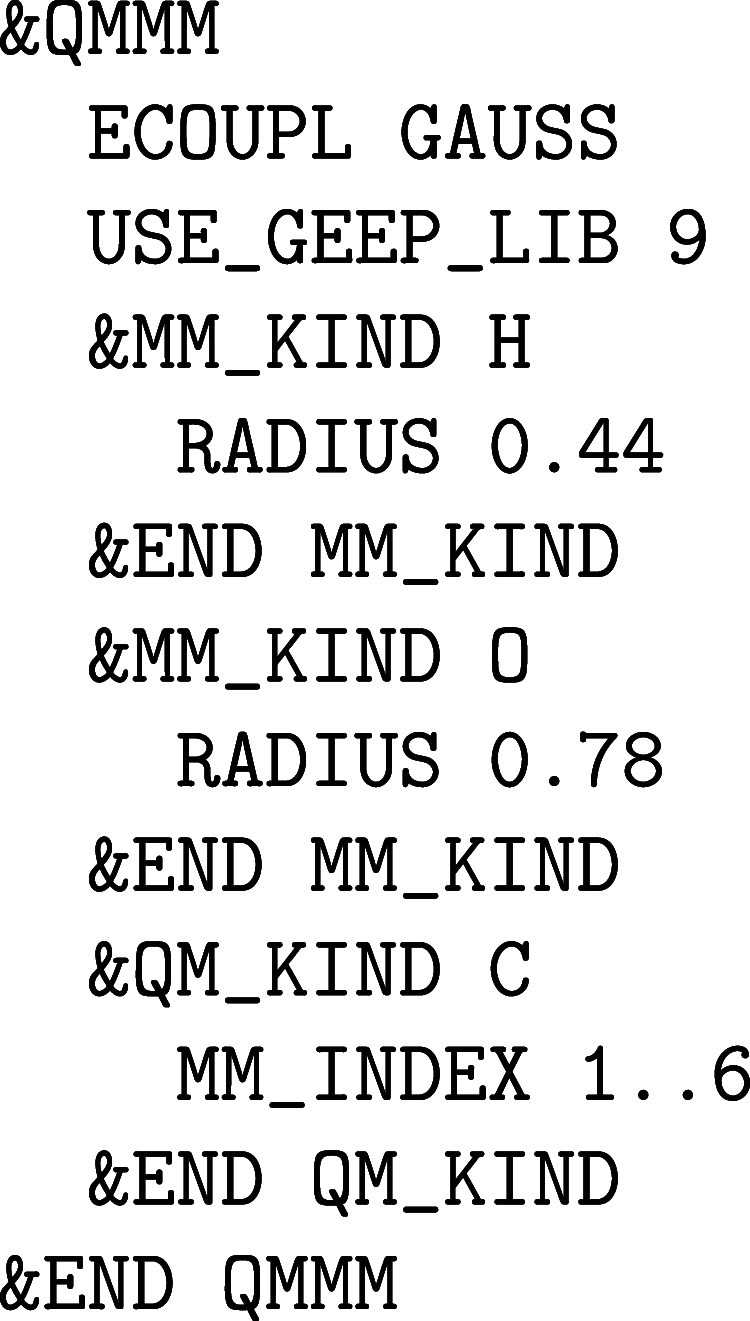


The treatment of long-range forces in conjunction with PBC is much less well established for QM/MM and most of the implementations use a truncation scheme, neglecting interactions beyond a certain cutoff. The reaction field approach combines truncation and polarizable continuum beyond a given cutoff. Ewald techniques are usually implemented only for QM-MM interactions and not for QM–QM, though long-range QM–QM may play a significant role, in particular for materials science applications. The electrostatic energy in CP2K is then composed of three terms, i.e. *E*_ES_ = *E* ^MM^ + *E* ^QM^ + *E* ^QM/MM^. The first term is evaluated using standard techniques, such as particle–particle or particle-mesh schemes. The second term is the evaluation of the energy of the QM subsystem. Since the total energy of the QM subsystem is usually evaluated using a smaller cell, care needs to be taken to include the correct electrostatics. The last term is the evaluation of the periodic electrostatic potential *v*_MM_(**r**, **R**_MM_) discussed above, divided into a real-space contribution and a periodic correction. The real-space term contains the interactions due to the short-range part of the electrostatic potential of the MM charges. Only MM atoms close to the QM region will contribute to this term. Since we use the Gaussian expansion plus the residual term, the radius of the Gaussian is such that only a few terms in the lattice sum are needed. The effect of the periodic replicas is only in the long-range term and it comes entirely from the residual function, thus
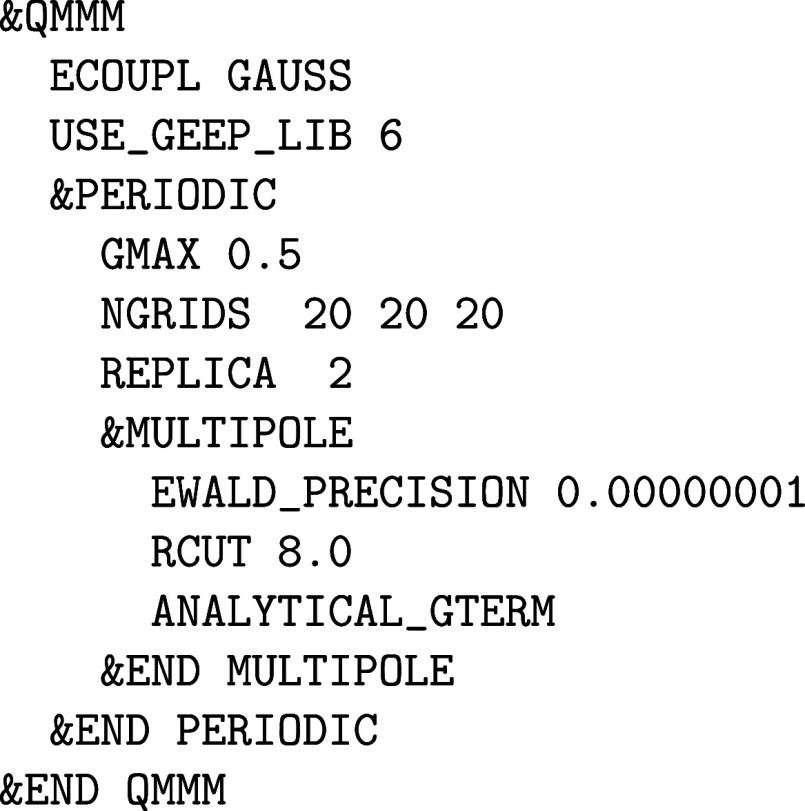


The residual function is represented in the Fourier space and can be evaluated analytically. Furthermore, since the long-range contribution is very smooth, the Fourier transform is zero for all *G* vectors larger than a well-defined maximum. In this example, G_MAX specifies the maximum *G* vector in reciprocal space for the Ewald sum and depends strongly on the number of Gaussian functions used in the GEEP scheme. The number of grid points used for the interpolation of the *G*-space term is given by NGRIDS, whereas REPLICA is the number of cell replicas to take into consideration for the real-space sum. Usually, 3–4 grid levels are used corresponding to a speed-up of 10^2^ times faster than the simple collocation algorithm (interpolations and restrictions account for a negligible amount of computing time). Since the residual function is different from zero only for a few vectors, the sum in reciprocal space is restrained to a few points. Possible sources of error are the cutoff of the finest grid level to properly map the sharpest Gaussian functions, the cutoff of the coarse grid level related to the cutoff of the long-range residual function, and the error in cubic spline interpolation.

When computing the QM electrostatic term, unless the quantum box and the MM box have the same dimensions, the QM images, which are interacting by PBC implicitly in the evaluation of the Hartree potential, have the wrong periodicity. To correct for this error, CP2K implements the so-called Blöchl scheme for decoupling and recoupling according to the correct periodicity.^157,158^ Spherical Gaussians, which are QM atom centered and reproduce the correct multiple expansion, are derived by a density fitting scheme in *G*-space. They can be more than just one per atom site with different exponents. These charges reproduce the correct long-range electrostatics and are used for the decoupling and recoupling procedure. Indeed, since the electrostatic interaction of separated charge distributions (the array of periodic QM charge densities) depends only on its multipole moments, the model charge density is used to modify the Hartree potential and cancel the electrostatic interactions between the periodic images. The MULTIPOLE section sets up this scheme and is activated by default for periodic calculations. It should be switched off when QM and MM boxes are the same size, to avoid unnecessary computational costs.

#### 4.2.2 Image-Charge Augmented Quantum Mechanics/Molecular Mechanics Method

The IC-QM/MM approach is intended to simulate adsorbed molecules on metallic surfaces.^155^ The molecular adsorbates are described at the QM level, specifically using KS-DFT, while the metal is treated at the MM level. In the IC-QM/MM scheme, as implemented in CP2K, the electrostatic response of the metal to the presence of the adsorbate is described by an image charge distribution

41 $$\rho_{m}\left(r\right)=\sum_{a}c_{a}g_{a}\left({r,R}_{a}\right)$$

where **R**_*a*_ is the position of the metal atom *a* and *g*_*a*_ represents a Gaussian function centered at *a*. The charge distribution ρ_m_ generates the potential *V*_m_, while the charge distribution of the adsorbates produces the electrostatic potential *V*_e_. The coefficients *c*_*a*_ are determined self-consistently by enforcing the constant-potential condition, where *V*_m_(**r**) screens *V*_e_(**r**) within the metal such that *V*_e_(**r**) + *V*_m_(**r**) = *V*_0_. Therein, *V*_0_ denotes a constant potential, which is typically zero unless an external potential is explicitly applied.

To run an IC-QM/MM calculation, the following &IMAGE_CHARGE subsection must be added:
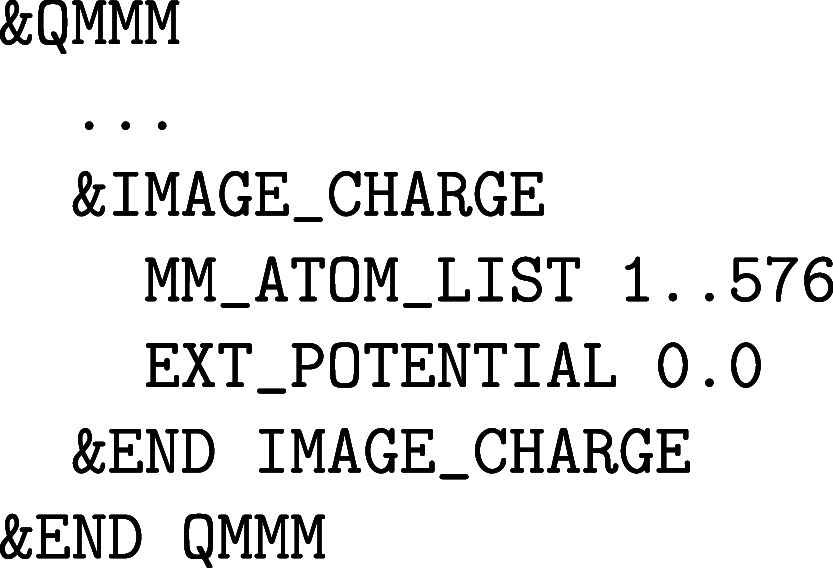


In an IC-QMM/MM calculation, the MM system is typically the whole metallic slab. Hence, QM and MM cells are thus required to have the same size. The relevant keywords are

- MM_ATOM_LIST: defines the list of MM atoms that carry the Gaussian charge *g*_*a*_. These should be the atoms of the metallic slab.
- EXT_POTENTIAL: sets the external potential *V*_0_.

There are several other keywords related to print options and to acceleration techniques of an IC-QM/MM calculation for specific run types, see online tutorial for more details.^159^ Note that the IMAGE_CHARGE section only accounts for the electrostatic interaction between molecules and metal atoms. Other contributions, such as vdW interactions, must be defined separately.

#### 4.2.3 Partial Atomic Charges from Restrained Electrostatic Potential Fitting

Restrained electrostatic potential (RESP) fitting is a widely used method to determine partial atomic point charges *q*_*a*_.^160^ In QM/MM simulations, these charges are required for the MM atoms when using the default electrostatic embedding of CP2K, and for both QM and MM atoms when using the less common mechanical embedding.

These charges are determined such that the potential *V*_RESP_ generated by *q*_*a*_ reproduces a given QM potential *V*_QM_ within a specified region of space. In practice, this is achieved by minimizing the residual *R*_esp_ through a least-squares fitting procedure for a set of predefined real-space grid points **r**_*k*_, where *R*_esp_ is given by

42 $$R_{esp}=\frac{1}{N}\sum_{k}^{N}{\left(V_{QM}\left(r_{k}\right)-V_{RESP}\left(r_{k}\right)\right)}^{2}$$

where *N* denotes the total number of real-space grid points **r**_*k*_. The resulting *q*_*a*_ are treated as point charges in nonperiodic RESP fittings. For periodic systems, however, the charges are represented by Gaussian functions *g*_*a*_ of fixed width, centered on atom *a*, resulting in the charge distribution

43 $$\rho_{RESP}=\sum_{a}q_{a}g_{a}$$

For details on the GPW-based periodic RESP implementation in CP2K, see ref 161. The choice between nonperiodic and periodic RESP fitting is made automatically, depending on the periodicity of the reference potential *V*_QM_.

The QM potential *V*_QM_ is obtained from a previous DFT or HF calculation. The RESP fit starts as a postprocessing step and is enabled by
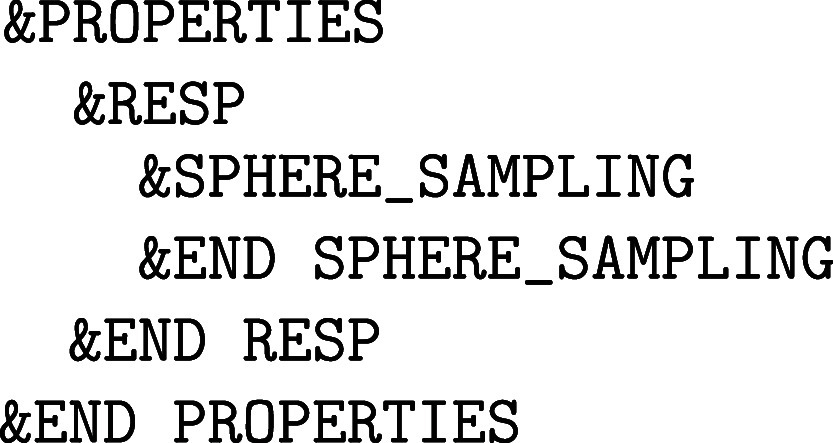


The choice of sampling points **r**_*k*_ is crucial to obtain meaningful charges that accurately reproduce the electrostatic potential in spatial regions relevant to interatomic interactions. As a general guideline, regions where the QM potential *V*_QM_ varies rapidly (e.g., within the vdW radii of atoms) should be avoided. The specific choice of sampling regions is system-dependent. The following options are available:

- SPHERE_SAMPLING: the real-space points **r**_*k*_ are sampled in spherical shells around each atom. The shells are defined by a minimal and maximal radius, which can be set by the RMIN and RMAX keywords in this section. This option should be used for molecules, molecular liquids, or porous periodic systems like metal–organic frameworks.
- SLAB_SAMPLING: the option should be used for slab-like systems, where it is important to reproduce the potential well above the surface, e.g. to study adsorption processes. The **r**_*k*_ grid is then sampled as a thin slice above the surface. In that case, keywords defining the surface atoms, the direction, and the thickness of the slice need to be set.

A set of constraints and restraints is typically employed to avoid unphysical values for *q*_*a*_ and to stabilize the fit. The total residual *R*, which is minimized, is *R* = *R*_esp_ + *R*_rest_ + *R*_const_. For the restraint, CP2K uses a harmonic penalty function

44 $$R_{rest}=\beta \sum_{j}{\left(q_{j}-t_{j}\right)}^{2}$$

where β denotes the strength of the restraint and *t*_*j*_ is the anticipated target charge. Restraints can be explicitly set by
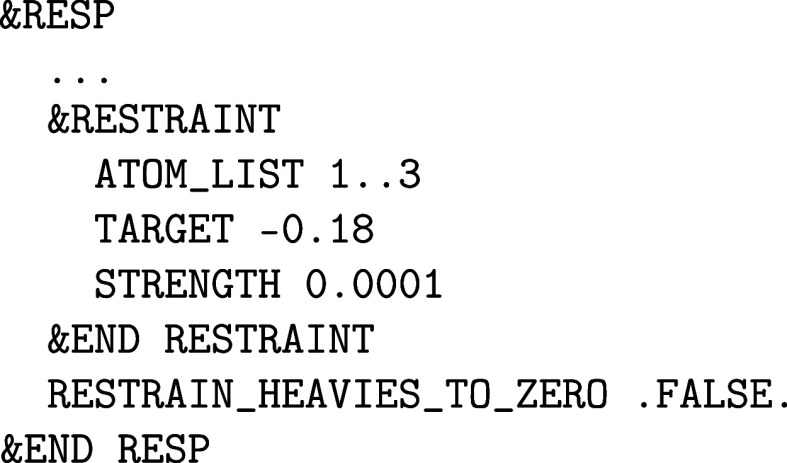


In this example, the target charges *t*_*j*_ in eq 44 are set to −0.18 for atoms with the index 1 to 3. The strength β of the restraint is defined by the keyword STRENGTH. When explicitly defining restraints, the default restraint RESTRAIN_HEAVIES_TO_ZERO, which sets *t*_*j*_ to zero for all elements but hydrogen, should be turned off. Different constraints are possible, yet by default, CP2K constrains the sum of all fitted charges to the total charge *q*_tot_ of the system, i.e.

45 $$R_{const}=\lambda \sum_{j}\left(q_{j}-q_{tot}\right)$$

Further explicit constraints can be given by adding
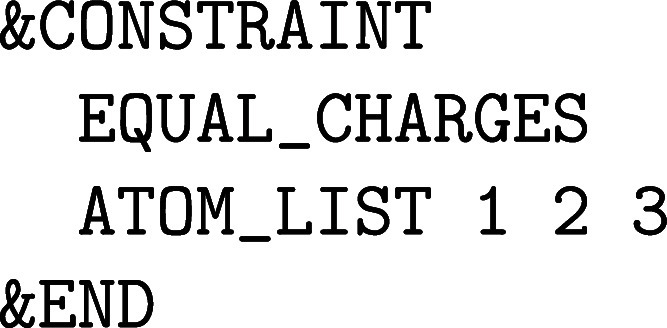
to the &RESP section. This is to say that the atoms with indices 1, 2, and 3 carry the same charge. More details on how to set constraints can be found in the online tutorial.^162^

CP2K also features a GPW implementation of the so-called repeating electrostatic potential extracted atomic (REPEAT) method,^163^ which is an adaptation of the RESP approach for periodic systems. When using the REPEAT method, eq 42 is modified so that the variance of the potentials is fitted rather than the absolute difference. Fitting the variance is generally easier and helps stabilize the fit for periodic systems. We note that the REPEAT method was originally introduced to handle the arbitrary offset of the electrostatic potentials *V*_QM_ and *V*_RESP_ in infinite systems.^163^ In CP2K, both potentials are computed with the GPW method, sharing thus the same offset. The REPEAT method is activated by adding the keyword USE_REPEAT_METHOD to the &RESP section. More details on the REPEAT implementation in CP2K can be found in ref 14 and in the online tutorial.^162^

### 4.3 Density Functional Embedding Theory

Density functional embedding theory (DFET) separates the systems into the relevant cluster and an environment. The cluster is described with the high-level electronic structure method, often employing a correlated WF (CW) ansatz, while the environment and the interaction between the cluster and the environment are described with DFT via the uniquely defined local embedding potential *v*_emb_(**r**).^164^ The total energy of the system is calculated within first-order perturbation theory, i.e.

46 $$E_{total}^{DFET}=E_{total}^{DFT}+\left(E_{cluster,emb}^{CW}-E_{cluster,emb}^{DFT}\right)$$

where *E*_total_^DFT^ and *E*_cluster,emb_^DFT^ are the DFT energies of the entire system and the embedded subsystem, respectively, while *E*_cluster,emb_^CW^ is the energy of the embedded cluster at the CW level of theory. All these entities are computed with an additional one-electron embedding term ∫d**r** *V*_emb_ ρ­(**r**) in the Hamiltonian.

The embedding potential is obtained in a top-down approach from the condition that the sum of embedded subsystem densities should reconstruct the DFT density of the total system. This can be achieved by maximizing the Wu–Yang functional with respect to *v*_emb_(**r**),^165^ i.e.

47 W[Vemb]=Ecluster[ρcluster]+Eenv[ρenv]+∫dr⁡Vemb(ρtotal−ρcluster−ρenv)

with the functional derivative being identical to the density difference such that $\frac{\delta W}{\delta V_{emb}}=\rho_{total}-\rho_{cluster}-\rho_{env}$ .

#### 4.3.1 General Procedure

The DFET workflow starts with obtaining the embedding potential *v*_emb_(**r**). First, one calculates the total density of the system before DFT calculations on the various subsystems with the current updated embedding potential are performed. Then, the potential is updated and the step is repeated until the total DFT density is matched by a sum of the densities of the embedded subsystems. When this condition is fulfilled, the embedded higher-level theory calculation is performed on the isolated cluster.

#### 4.3.2 Implementation

The DFET implementation is available for closed and open shell systems, in terms of unrestricted and restricted open-shell formalisms, respectively. It is limited to GPW calculations only with PPs describing the core electrons. All electronic structure methods implemented within CP2K/Quickstep are available as a higher-level method, including hybrid DFT, MP2, and RPA. It is possible to perform property calculations on the embedded cluster using an externally provided *v*_emb_(**r**). The subsystems can employ different basis sets, although they must share the same PW grid.

DFET calculations are activated via the &MULTIPLE_FORCE_EVALS section in the root:
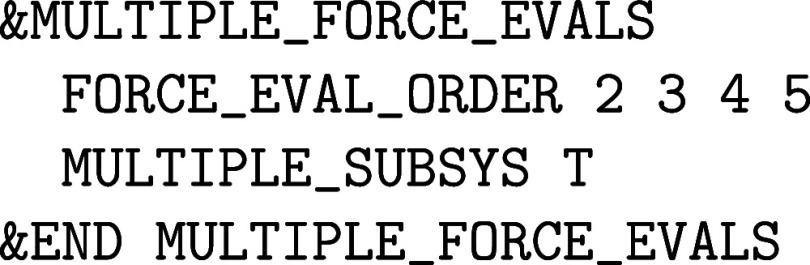


The order of force evaluations is arbitrary, but it is recommended to leave it as is. Here, &FORCE_EVAL 2 refers to the environment, 3 to the embedded cluster, and 4 to the total system, all of which are calculated at the DFT level. The isolated cluster computed at a higher-level of theory is denoted as &FORCE_EVAL 5. The first force evaluation in &FORCE_EVAL defines the general embedding framework:
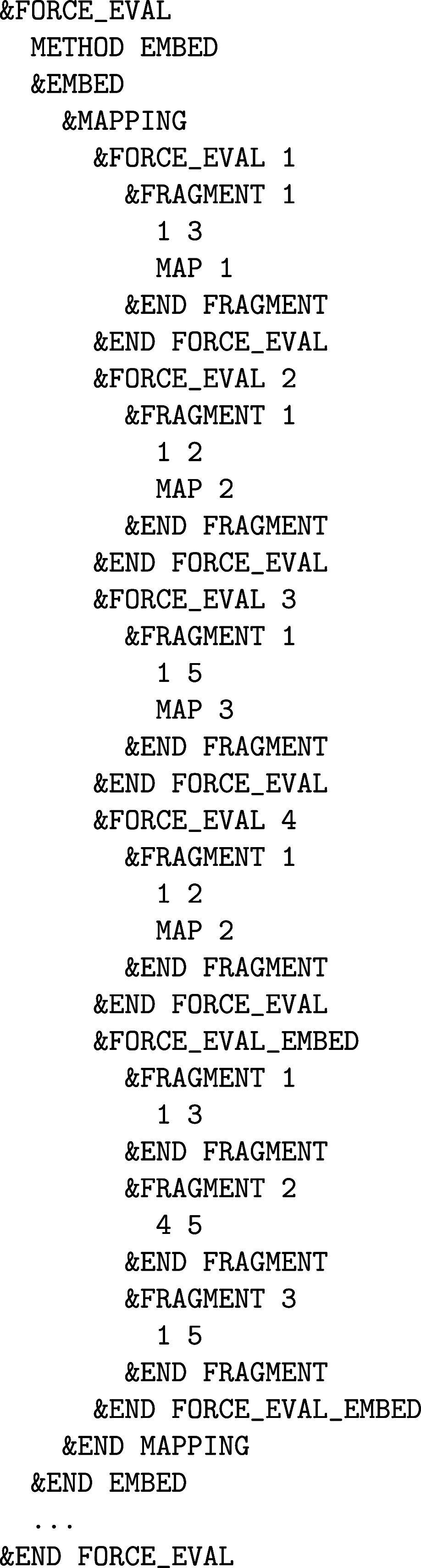


The mappings define the correspondence of atoms in the parts and in the total system, i.e. atoms 1 and 2 (MAP 2 of &FORCE_EVAL 2) correspond to atoms 4 and 5 in the total system (&FRAGMENT 2 in the &FORCE_EVAL_EMBED section). In addition, this &FORCE_EVAL section must represent the total system as defined in the &SUBSYS section. The next two &FORCE_EVAL sections specify the environment and the cluster via standard DFT input sections. The latter one has the following additional keyword in the &QS section:
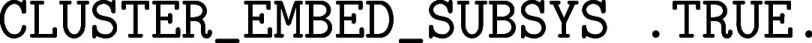


The next &FORCE_EVAL section to be defined is the total system, marked by the keyword
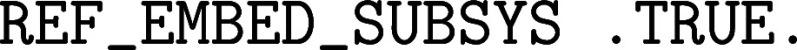
in the &QS section. In this force evaluation, the options for optimizing the embedding potential are to be specified. This is done by inserting a &OPT_EMBED section within the &QS section:
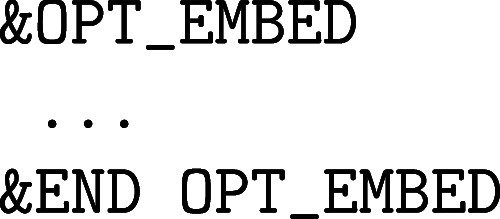


The final &FORCE_EVAL section represents the higher-level calculation of the embedded cluster. It can be either a standard hybrid/double-hybrid DFT or a CW calculation with the following additional keyword in the &QS section:
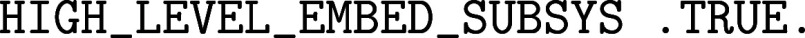


Several options for representing and optimizing the embedding potential are implemented in CP2K. Most importantly is the &OPT_EMBED%GRID_OPT keyword, which defines whether the potential is represented on the real-space grid, or expanded in the auxiliary basis set. The first option is more accurate and computationally cheaper, and is therefore recommended for all practical purposes.

In addition, several techniques for optimizing *v*_emb_(**r**) are available, including standard gradient-based optimization schemes (e.g., SD, quasi-Newton, and level-shifting methods), as well as an iterative van Leeuwen–Baerends update,^166^ which is an alternative to the previously mentioned Wu–Yang functional.^165^ Despite its simplicity, the SD approach is typically relatively robust for potential optimizations and is therefore the default. Although several options for the initial guess of the embedding potential are available, starting with the zero potential is a rather reliable procedure, which is typically quickly converging.

The embedding potential is saved as a volumetric Gaussian cube file that can be used for visualization purposes and can be read in for restarting the optimization. For open-shell calculations, CP2K defines two potentials: one interacts with the electron density, whereas the other interacts with the spin density. Both are saved and should be specified for restarting the calculation. In addition, the embedding potential can be used for standalone embedded calculations, as in the following example for an open-shell system:
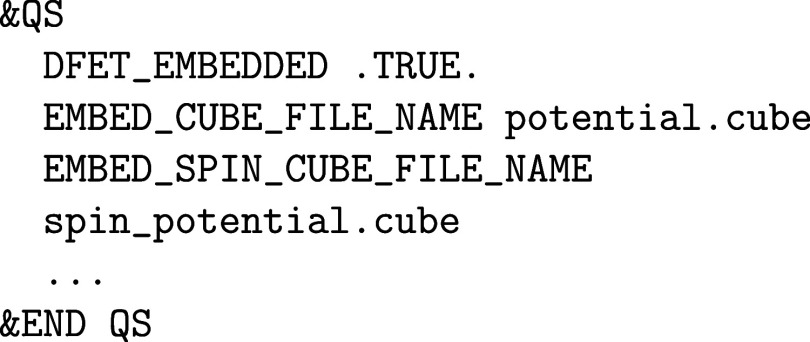


## 5 Nuclear Magnetic and Electron Paramagnetic Resonance Spectroscopy

The energy levels probed in magnetic spectroscopy correspond to transitions among nuclear and electronic spin eigenstates in the presence of an external magnetic field. The calculation of magnetic response properties in CP2K is based on variational density functional perturbation theory (DFPT).^167–169^

### 5.1 Density Functional Perturbation Theory

Generally speaking, CP2K/Quickstep uses DFPT to compute the perturbative corrections to the zeroth-order orbitals ψ_*k*_^(0)^. In case of a perturbing magnetic field *B*

48 $$\psi_{k}=\psi_{k}^{\left(0\right)}+B\psi_{k}^{\left(1\right)}+...$$

where ψ_*k*_^(1)^ is the first-order orbital correction, which provides energy corrections up to third order according to Wigner’s (2*n* + 1) rule.

The first-order orbitals can be obtained via the inhomogeneous set of coupled (Sternheimer) equations

49 $$-\sum_{i}^{N_{occ}}\left(H^{\left(0\right)}\delta_{ij}-\left\langle \psi_{i}^{\left(0\right)}\left|H^{\left(0\right)}\right|\psi_{j}^{\left(0\right)}\right\rangle \right)|\psi_{i}^{\left(1\right)}\rangle =H^{\left(1\right)}|\psi_{j}^{\left(0\right)}\rangle$$

Therein, the indices *i*, *j* run over the occupied orbital manifold {ψ}. This expression already anticipates the possibility that the employed zeroth-order orbitals might not be the canonical KS orbitals. Expanding the first-order orbitals in terms of atomic basis functions ϕ_*l*_

50 $$\psi_{l}^{\left(1\right)}=\sum_{l}c_{li}\phi_{l}^{\left(1\right)}$$

and projecting eq 49 on ϕ_*k*_

51 $$-\sum_{i}^{N_{occ}}\sum_{l}^{N_{basis}}\left(H_{kl}^{\left(0\right)}\delta_{ij}-S_{kl}\left\langle \psi_{i}^{\left(0\right)}\left|H^{\left(0\right)}\right|\psi_{j}^{\left(0\right)}\right\rangle \right)c_{li}^{\left(1\right)}=\sum_{l}H_{kl}^{\left(1\right)}c_{lj}^{\left(0\right)}$$

we obtain a set of *N*_basis_ × *N*_occ_ simultaneous equations. In case of magnetic responses, the first-order orbitals are imaginary, and one explicitly includes $i=\sqrt{-1}$ on the left-hand side, so that the expansion coefficients *c* ^(1)^ are real. It is to be noted that with atom-centered basis functions and vibrational perturbations (dipole derivatives, Born charges), the Sternheimer equation acquires an additional contribution on the right-hand side, due to the derivatives of the overlap matrix.

In CP2K, eq 51 are solved self-consistently using a CG-based minimizer.^167,170^ In the employed parallel-transport gauge, the first-order orbitals are orthogonal to the occupied orbital manifold.^170^ This can be imposed by projecting the right-hand sides of the equations onto the unoccupied orbital manifold, which amounts to solving the equations while imposing the orthogonality between the trial first-order orbitals and the occupied orbitals.

A prototypical input for such a linear response calculation looks like:
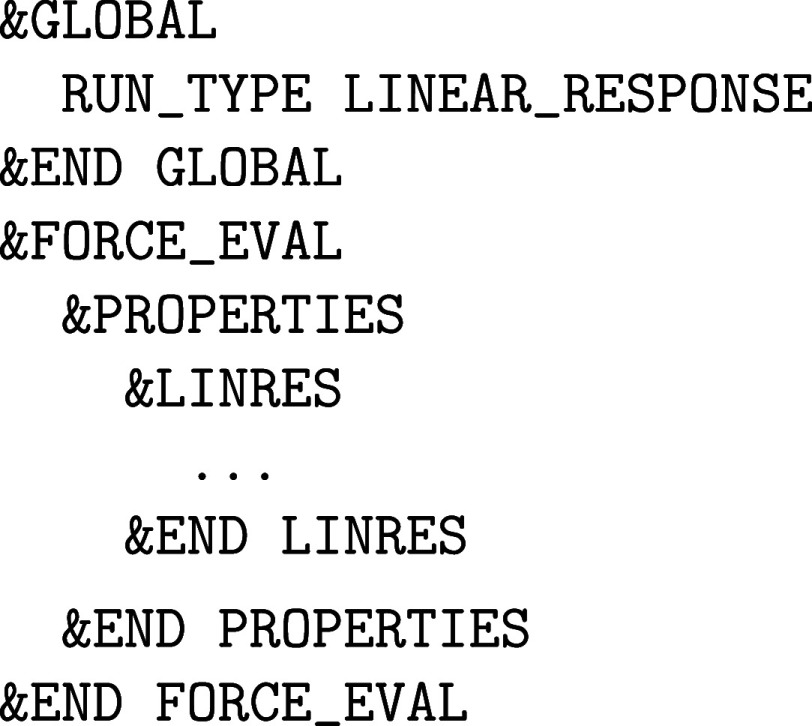


In the relevant &LINRES input section, which is located inside the &PROPERTIES section, one can set the parameters of the preconditioned CG optimizer. The general principles on how to choose the specific preconditioner, maximum number of iterations, and convergence threshold are similar to Section 2.1.4. Yet, the default values typically provide a robust starting point. Nevertheless, the convergence behavior of different response properties can differ, and it is advisible to check whether the desired response property is converged with respect to the chosen CG convergence threshold.

Also inside the &LINRES section, different subsections give access to various response properties. As will be explained later, when calculating magnetic field-induced current densities (required for several magnetic response properties) maximally localized Wannier functions (MLWF) are employed as the zeroth-order orbitals. The corresponding &LOCALIZE input section is also found within the &LINRES section, as are

- &CURRENT: the induced current density in response to an external homogeneous magnetic field.^168^
- &LOCALIZE: computes localized orbitals, which are required if the perturbation includes the position operator.
- &NMR: the nuclear magnetic shielding tensors, or more generally, the nucleus-independent chemical shifts (NICS).^171^
- &EPR: the electronic g-tensor.^172^
- &POLAR: computes the polarizability.^173^
- &VCD: enables the calculation of vibrational circular dichroism (VCD).^174^
- &DCDR: analytical gradients of the dipole moments, e.g. for Born effective charges and atomic polar tensors.

Note that the calculation of some response properties might require the explicit inclusion of more than one of the above, e.g. &CURRENT requires &LOCALIZE. These two sections are in turn prerequisites for &NMR and &EPR sections, respectively. Detailed information relating to individual input sections for magnetic response properties will be given in the following.

### 5.2 The Magnetic Shielding Tensor

The interaction of electrons with an external magnetic field **B** leads to an induced current density **j**(**r**), which in turn leads to an induced magnetic field

52 $$B^{ind}\left(r\right)=\frac{1}{c}\int dr\frac{\left(r^{\prime }-r\right)}{{\left|r^{\prime }-r\right|}^{3}}\times j\left(r^{\prime }\right)$$

where *c* is the speed of light, and Gaussian atomic units are being used. The perturbing field can be an external magnetic field **B** ^ext^ (taken to be spatially homogeneous), or the magnetic moments associated with spins. We generally assume in this section a closed-shell system with vanishing total electron spin. In this case, the only spins in the system are the nuclear ones **m** ^*N*^

53 $$B^{ind}\left(r,B^{ext},\left\{m^{N}\right\}\right)=-\sigma \left(r\right)B^{ext}-\sum_{i}^{Nuclei}K_{i}\left(r\right)m_{i}^{N}\left(r_{i}^{N}\right)+...$$

The reduced indirect spin–spin coupling is denoted as **K**(**r**) in eq 53, while the tensor field

54 $$\sigma \left(r\right)=-\frac{\partial B^{ind}\left(r\right)}{\partial B^{ext}}$$

is known as the magnetic shielding tensor. Experimentally, the induced magnetic field can only be probed at the position of a nuclear spin **r** ^*N*^. Being a ratio, **σ**(**r**) is conventionally reported in units of parts per million. The experimental value is almost always referenced to the isotropic average $\left(\frac{1}{3}Tr\left(\sigma \left(r\right)\right)\right)$ of the magnetic shielding tensor of some chosen standard for the respective nucleus, i.e.

55 $$\delta \left(r_{i}^{N}\right)=\sigma_{i,ref}^{iso}I-\sigma \left(r_{i}^{N}\right)$$

which is known as the chemical shift tensor, and its isotropic average δ is called the chemical shift. Moreover, **I** is the unit matrix.

At lowest order, the energy of the system is linear in the total magnetic field, thus one can also define the shielding tensor as the second derivative of the energy with respect to the nuclear magnetic moment **m** ^**N**^ and the external magnetic field

56 $$\sigma \left(r^{N}\right)={\frac{\partial^{2}E}{\partial B^{ext}\partial m^{N}}|}_{\left|B^{ext}\right|=\left|m^{N}\right|=0}$$

Given that the magnetic shielding is a tensor field, it can be computed in any arbitrarily chosen spatial position, even though it is experimentally measurable only at the nuclear positions. These are known as nucleus-independent chemical shifts (NICS), which are also available in CP2K.^171^

From eqs 52 and 54, the elements of the shielding tensor are given by

57 $$\sigma_{xy}\left(r\right)=\frac{1}{c}\int_{\Omega }dr{\left[\frac{r^{\prime }-r}{|r^{\prime }-r{|}^{3}}\times j_{x}\left(r^{\prime }\right)\right]}_{y}$$

where the integration is over the whole material, including all periodic replicas in the case of extended systems. It is obvious that the induced current density is the key ingredient in computing magnetic shieldings. Once **j**(**r**) is known, the induced field, and hence the shielding tensor as well, is available as a simple three-dimensional integral.

Using the approach developed by Sebastiani and Parrinello,^168^ CP2K evaluates the magnetic perturbation using MLWFs in combination with DFPT.^172^ As explained later, the localized nature of the Wannier functions allows the use of the position operator in the perturbation Hamiltonian. An underlying core assumption here is that each Wannier function is contained entirely within the simulation cell once it is centered. For insulators, it is known that the Gaussian functions decay exponentially, so that for sufficiently large simulation cells, this assumption is not irrelevant.^175^ In comparison to other approaches developed for condensed phase systems,^168,176^ CP2K differs in its use of local atom-centered Gaussian functions (see Section 2), allowing for reduced complexity algorithms and hence large scale calculations of magnetic resonance parameters. With the GAPW method, CP2K/Quickstep is also able to compute all-electron magnetic resonance parameters. One can also use embedding techniques such as QM/MM methods, and even further decompose the QM part into GAPW/GPW regions. The reliance on the localization properties of Wannier functions means, however, that this implementation is not suitable for conductors, where the Wannier functions decay only algebraically.

In order to compute the shielding tensors one first needs to use the GAPW method with an all-electron basis set (the calculation will also be possible using GPW, but then the contributions of the core electrons to the induced current density are missing). Inside the &LINRES section one then also needs to include the &LOCALIZE section to compute the MLWFs, which are then provided to the linear response module as the zero-order orbitals. Otherwise, CP2K will exit with an error if the user requests the calculation of the current density or the shielding tensors, without activating the &LOCALIZE section. Second, the &CURRENT section should be included for computing the induced current density using DFPT.^167^ Finally, the &NMR section requests the printing of the shielding tensors or NICS maps by performing the integration in eq 57. However, before discussing the various input options in these sections, we provide a quick overview of the specifics of the DFPT-based implementation for computing the induced current density in CP2K.^172^

For a homogeneous magnetic field, the vector potential **A** can be chosen as

58 $$A\left(r\right)=\frac{1}{2}B\times \left(r-r_{0}\right)$$

where **r**_0_ is an arbitrary gauge origin. Using the explicit form of the vector potential given in eq 58 leads to perturbations involving the position operator, which is problematic under PBC, as it jumps discontinuously at the edges of the cell. The solution suggested by Sebastiani and Parrinello is to use maximally localized Wannier functions as the zeroth-order orbitals ψ^(0)^(**r**).^168^ If the simulation cell is chosen to be larger than the decay length of the Wannier functions (which is exponentially decaying for insulators) and choosing for each Wannier function ψ_*i*_ a coordinate system such that its center is at the origin (via a translation vector **d**_*i*_), one avoids the problematic behavior of the position operator at the cell boundaries. It should be noted that this choice of a different origin for the entire coordinate system for individual Wannier functions is not a gauge transformation, as both **r** and **r**_0_ in eq 58 are being simultaneously shifted. The current is invariant under arbitrary orbital-specific translations.

With the introduction of orbital-specific translations **d**_*i*_, one ends up with three perturbation operators.^168^ The linear momentum operator

59 $${Ĥ}^{P}=\mathrm{p̂}$$

the orbital angular momentum operator

60 $${Ĥ}^{Li}=\left(\mathrm{r̂}-d_{i}\right)\times \mathrm{p̂}$$

and the full correction operator

61 $${Ĥ}^{\Delta i}=\left(d_{i}-d_{j}\right)\times \mathrm{p̂}$$

where in the last equation **d**_*i*_ – **d**_*j*_ is computed using the minimum image convention. The last two operators are labeled with the orbital index *i* to denote their orbital dependence via the chosen orbital centers **d**_*i*_. All three operators are vector operators, leading to nine contributions per orbital. Each contribution from the first two operators can be obtained essentially at the cost of one total energy calculation. The full correction operator requires one such calculation per orbital, making it by far the computationally most expensive term for any system with more than a handful of orbitals.

Once these perturbation operators are used in the inhomogeneous set of equations shown in eq 49, and all the contributions to the linear response orbitals have been obtained, the linear response current density vector is computed for the three directions of the magnetic field, allowing the computation of all the components of the shielding tensor. The *x*-component of the linear current density response induced by an external magnetic field applied along the *y*-axis is given by a sum of a paramagnetic *j*_*xy*_^*p*^(**r**) and a diamagnetic *j*_*xy*_^*d*^(**r**) contribution,^172^ i.e.

62 jxyp(r)=−12c∑ikl[Cki(0)(CliLy+(r0−di)xCliPz−(r0−di)zCliPx−CliΔiy)×{(∇xχk(r))χl(r)−χk(r)∇xχl(r)}]

63 $$j_{xy}^{d}\left(r\right)={\left(r-r_{0}\right)}_{z}\rho \left(r\right)$$

and the other components are obtained analogously.

With our choice of the vector potential in eq 58, each of these two oppositely signed contributions to **j**(**r**) is linear in the gauge origin **r**_0_; their sum, however, should be invariant to it. With the local atomic basis functions commonly used in quantum chemistry, it is extremely difficult to converge the sum of the two terms at a reasonable computational cost, if one chooses a single fixed gauge origin, and the computed value of the current density becomes parametrically dependent on **r**_0_. This is the well-known gauge origin problem. To address this issue, a distributed gauge origin is used during the computation, with different methods making different choices regarding how this distribution is done. In CP2K the gauge origin is tackled using the individual gauge for atoms in molecules (IGAIM), where the gauge origin is the position of the nearest atom,^177^ or alternatively, with the continuous set of gauge transformations (CSGT), where the gauge origin is the current position **r**.^178^ It is obvious that the CSGT approach makes the diamagnetic contribution in eq 63 vanish.

The choice of the gauge origin is set within the &CURRENT input section:
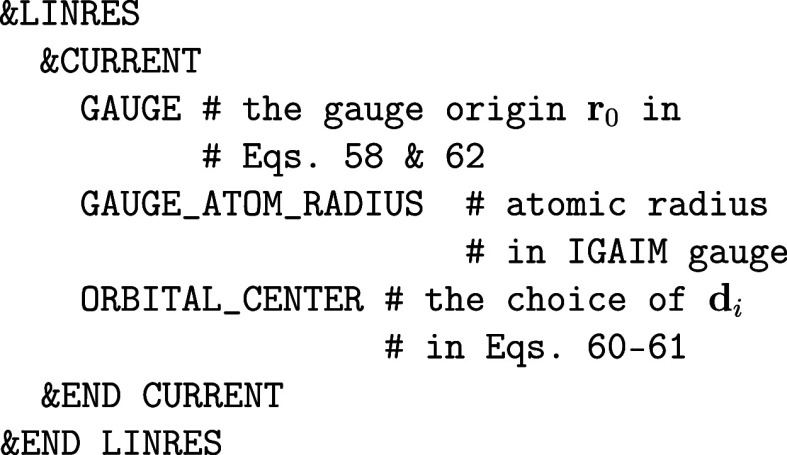


For the choice GAUGE R, one has the option to use CSGT for both the soft and local contributions (see eq 64 below) to the current density. GAUGE ATOM uses IGAIM centered on the nearest atom to the current grid point, and a combination of IGAIM for the hard part and CSGT for the soft part can be chosen with GAUGE R_AND_STEP_FUNCTION. For the IGAIM method, the radius of the atomic domain is set by the keyword GAUGE_ATOM_RADIUS. Although the choice of CSGT **r** = **r**_0_ is appealing, as it makes the diamagnetic term in eq 63 identically vanish, both empirical evidence and theoretical considerations indicate that other choices provide better results.^179^

With the keyword ORBITAL_CENTER, one can also control the choice of the orbital centers **d**_**i**_ used in the perturbation operators eqs 60 and 61. The choice WANNIER uses the position of the respective Wannier center and is the most expensive, requiring 3*M* + 6 response calculations, where *M* is the number of MOs. All other choices offer means to cluster orbital centers together, potentially leading to a substantial reduction in the required computational effort. At variance, ATOM uses the atomic positions as centers, hence the Wannier functions are clustered on the nearest atom, whereas BOX divides the simulation cell into sub-boxes and clusters the centers within each sub-box (the keyword NBOX sets the number of boxes along each dimension). Finally, COMMON uses a common center, whose position is specified by the keyword COMMON_CENTER, which only works for isolated molecules.

With the keywords
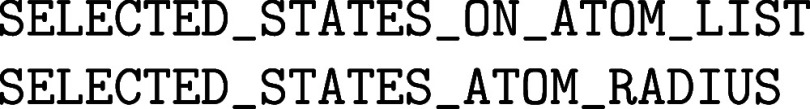
one can further choose to perform the response calculation using only the Wannier functions that are within a certain distance from some chosen list of atoms. If only the magnetic shieldings of some particular atoms are of interest, this can lead to enormous reductions in computational time. Naturally, one needs to ascertain that the selected radius provides acceptable results for the atoms of interest, which depends on the respective chemical environments.

Finally, the calculation of the magnetic shielding tensor requires the evaluation of the integral in eq 57. For a periodic system, the current density is also periodic and the computation can be done efficiently using the GAPW method (Section 2). The current density is decomposed in a manner analogous to the GAPW electron density, thus

64 $$j\left(r\right)=\mathrm{j̃}\left(r\right)+\sum_{A}^{atoms}\left(j\left(r\right)-\tilde{j_{A}\;}(r)\right)$$

where **j̃** is the soft contribution to the current density, **j**_*A*_ is the local hard contribution at atom *A*, and $\tilde{j_{A}\;}$ is the local soft contribution to prevent double counting.

The soft contribution **j̃** is computed in reciprocal space on the PW grid.^168^ One has to note here, however, that the **G** = 0 component cannot be computed under PBC. This term is commonly called the susceptibility correction and depends on the bulk magnetic susceptibility. It is determined by macroscopic magnetostatics from the macroscopic shape of the sample. In CP2K this term is calculated from the susceptibility due to **j̃**, assuming a spherical sample shape, which is also the experimental convention^180^

65 $$\chi_{xy}=\frac{2\pi }{\Omega_{c}c}\int dr{\left[r\times {\mathrm{j̃}}_{x}\left(r\right)\right]}_{y}$$

For the local part of the current density, the contribution to σ_*xy*_ of the nucleus at position **r** ^*N*^, arising from the induced local current densities, is evaluated as

66 $$\sigma_{xy}\left(r^{N}\right)=\frac{1}{c}\sum_{B}\int_{\Omega_{B}}dr\;{\left[\frac{r-r^{N}}{|r-r^{N}{|}^{3}}\times \left(j_{x,B}\left(r\right)-{\mathrm{j̃}}_{x,B}\left(r\right)\right)\right]}_{y}$$

where the sum over atoms *B* is restricted to the nuclei that are within a radius *R*_*c*_ from **r** ^*N*^ (see keyword &NMR%SHIFT_GAPW_RADIUS below). The integration over the atomic domain Ω_*B*_ is performed numerically on a spherical grid with a logarithmic radial and a Lebedev angular grid. The numerical integration converges rapidly with respect to the number of grid points, and about 10000 grid points per atom are enough to converge the chemical shift below 0.1 ppm.^172^ The size of the atomic integration grid is set inside the &KIND subsection, and for the computation of the shielding tensors it can be beneficial to use values that are higher than the defaults, e.g.
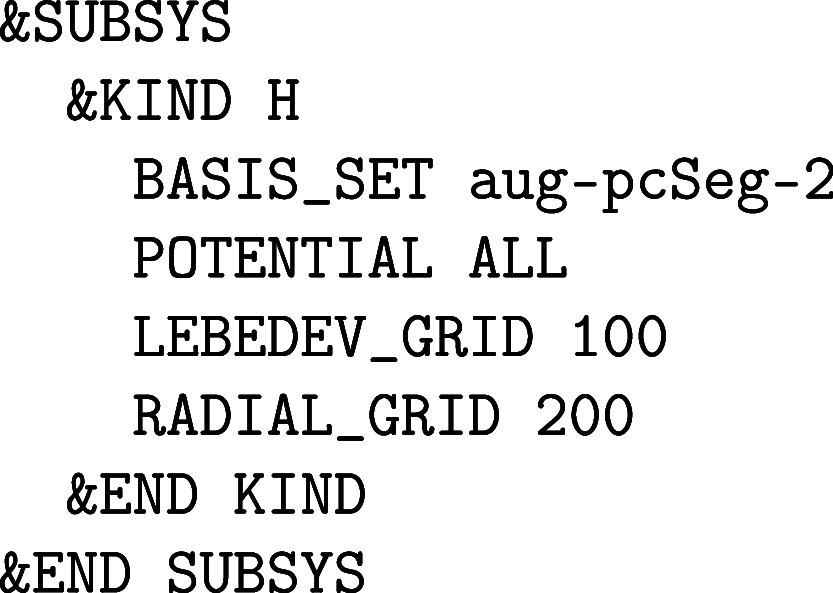


In the &NMR input section, one can set the value of *R*_*c*_, request a NICS calculation, and specify the spatial points for the latter:
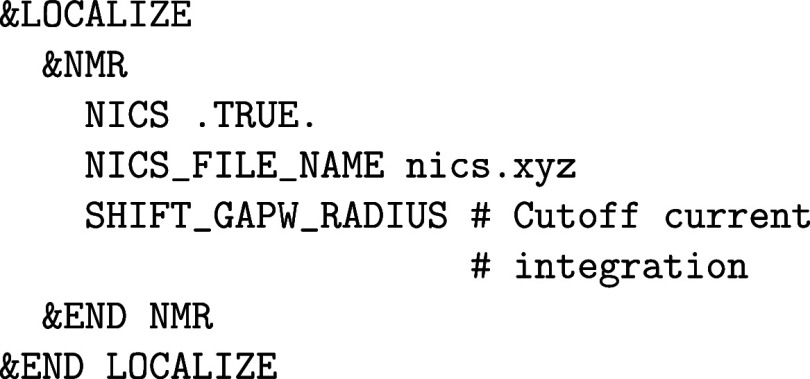


### 5.3 The Electron Paramagnetic Resonance g-Tensor

CP2K also offers an implementation of the g-tensor under PBC, which employs the same DFPT approach to compute the induced current densities. This makes it feasible to perform large-scale calculations, including with QM/MM (e.g., paramagnetic active sites in enzymes), to investigate paramagnetic defects in solids under PBC, and also to perform large-scale computations of paramagnetic NMR shifts.^181^

Effectively, the g-tensor plays in EPR spectroscopy a role similar to that played by the shielding tensor in NMR spectroscopy. This is clear in an effective spin-Hamiltonian framework, which gives the coupling between an effective electronic spin **S** and an external magnetic field **B** ^ext^ as the bilinear term

67 $${Ĥ}_{eff}=\frac{1}{2c}B^{ext}\cdot g\cdot S$$

and like the shielding tensor, the g-tensor is also a second partial derivative of the energy with respect to the external field and a spin magnetic moment, in this case the electronic spin. The corresponding ab initio expression is obtained from the minimal coupling electronic Hamiltonian including relativistic corrections up to $O\left(\alpha^{3}\right)$ , where α is the fine structure constant. Identification of the relevant Hamiltonian terms produces the expression for the g-tensor, in the form of spatially anisotropic relativistic correction terms Δ ***g*** to the free electron *g*-value *g*_e_. The implementation in CP2K is based on the Schreckenbach–Ziegler DFT-based approach,^182^ and its extension by Pickard and Mauri.^183,184^ One can identify three contributions to each component of Δ ***g***, i.e.

68 $$g_{xy}=g_{e}\delta_{xy}+\Delta g_{xy}^{ZKE}+\Delta g_{xy}^{\mathrm{SO}}+\Delta g_{xy}^{SOO}$$

The correction terms are the Zeeman kinetic energy (ZKE) term

69 $$\Delta g_{xy}^{ZKE}=-\frac{g_{e}}{c^{2}}\left(T^{\alpha }-T^{\beta }\right)\delta_{xy}$$

which is a purely kinematic scalar relativistic correction, the spin–orbit (SO) term (usually the dominant term)

70 $$\Delta g_{xy}^{\mathrm{SO}}=\frac{\left(g_{e}-1\right)}{c}\int_{\Omega_{c}}dr[j_{x}^{\alpha }\left(r\right)\times \nabla V_{eff}^{\alpha }\left(r\right)-j_{x}^{\beta }\left(r\right)\times \nabla V_{eff}^{\beta }\left(r\right){]}_{y}$$

and the spin-other-orbit (SOO) term

71 $$\Delta g_{xy}^{SOO}=\frac{1}{S}\int_{\Omega_{c}}drB_{y,B_{x}}^{corr}\left(r\right)\left[\rho^{\alpha }\left(r\right)-\rho^{\beta }\left(r\right)\right]$$

where α and β denote the spin channels, *S* the total spin, and *T* is the kinetic energy. Moreover, $B_{y,B_{x}}^{corr}$ is the *y*-component of the magnetic field due to the total induced current density by a field (see eq 52) in the *x*-direction minus a self-interaction correction

72 $$B_{y,B_{x}}^{corr}\left(r\right)=\frac{1}{c}\int_{\Omega }dr^{\prime }{\left[\frac{r^{\prime }-r}{|r^{\prime }-r{|}^{3}}\times \left(j_{x}\left(r^{\prime }\right)-j_{x}^{\alpha -\beta }\left(r^{\prime }\right)\right)\right]}_{y}$$

with **j** ^α–β^ = **j** ^α^ – **j** ^β^. The effective potential *V*_eff_ in the SO contribution is the sum of the external potential, the Hartree potential, and the XC potential.^182^ Currently, the CP2K implementation only supports LDA and GGA functionals; hybrid XC functionals are not yet supported.

The ZKE contribution is computed from the kinetic energy of the spin-polarized KS orbitals in the Gaussian basis set. The SO and SOO terms are similar to the shielding tensor in that they require the (spin-dependent) current density. For the latter, the same techniques based on the aforementioned Sebastiani–Parrinello approach,^168^ discussed in the context of the magnetic shielding tensor section, are also employed here. One subtlety with respect to the SOO term is that it requires the induced field over all space, not only at the positions of the nuclei. The nonlocal nature of the dipolar term in eq 52 makes it very taxing to create a GAPW representation of the induced magnetic field. Hence, the implementation employs an approximation of the SOO term, whereby the contributions from atom-centered (local) current densities to the **G** ≠ 0 components of the induced field are ignored.

The g-tensor is generally much less sensitive to the choice of gauge than nuclear magnetic shieldings, and even the computationally convenient CSGT (&CURRENT%GAUGE R) can be used. Tables 1 and 2 in ref 181 offer a comparison of g-tensors calculated by CP2K with the IGAIM gauge versus those calculated by ORCA^185^ and MAG-ReSpect^186^ using a variety of gauge origins and spin–orbit treatments.

It is important to note that if the simulation cell contains more than one paramagnetic center, the individual center g-tensors erroneously add up. This is a problem in existing implementations of g-tensors in condensed phases.^181,187^ Hence, in this case one needs to normalize the g-tensor of the simulation cell

73 $$g=g_{e}1+\frac{1}{n}\Delta g$$

with *n* being the number of paramagnetic centers in the cell.

To activate the calculation of the g-tensor in the input file, one has to request the spin density from an unrestricted KS calculation (keyword &DFT%UKS). One also needs to include the &CURRENT, &LOCALIZE, and &EPR subsections within the &LINRES section. The various control parameters related to the calculation of the induced current density, in the input section &CURRENT, were already discussed for the magnetic shielding tensors. The input subsection
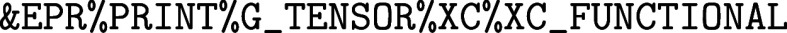


within the &LINRES section sets the functional for the effective XC potential in eq 70. Other XC potentials are activated in a similar fashion, for example XALPHA corresponds to the Dirac–Slater potential, whereas BECKE88 together with LYP will use the BLYP XC potential.

### 5.4 Hyperfine Couplings

The effective spin-Hamiltonian term for the hyperfine coupling is bilinear in the nuclear and electron spin

74 Ĥ=S·A·I

The ab initio expression for the hyperfine coupling tensor, or **A**-tensor, is obtained from relativistic quantum mechanics, as in the case of the g-tensor. For any nucleus *N*, the dominant terms are an isotropic (Fermi contact) term

75 $$A_{iso,N}=\frac{4\pi }{3}\frac{g_{e}\mu_{e}g_{N}\mu_{N}}{\left\langle S_{z}\right\rangle }\int dr\rho^{\alpha -\beta }\left(r\right)\delta_{T}\left(r\right)$$

and an anisotropic dipolar term

76 $$A_{ani,N}=\frac{1}{2}\frac{g_{e}\mu_{e}g_{N}\mu_{N}}{\left\langle S_{z}\right\rangle }\times \int dr\rho^{\alpha -\beta }\left(r\right)\frac{3r_{i}r_{j}-\delta_{ij}r^{2}}{r^{5}}$$

where μ_e_ is the Bohr magneton, μ_*N*_ the nuclear gyromagnetic ratio, μ_*N*_ the nuclear magneton, ⟨*S*_*z*_⟩ is the expectation value of the *z*-component of total electronic spin, and **r** is taken relative to the position of the nucleus *N*. Moreover, ρ^α–β^(**r**) and *g*_e_ have already been introduced, whereas δ_*T*_ is a smeared-out delta function which results from scalar relativistic corrections (in the nonrelativistic limit, it collapses to a Dirac delta function), i.e.

77 $$\delta_{T}\left(r\right)=\frac{1}{4\pi r^{2}}\frac{2}{Z\alpha^{2}}\frac{1}{{\left(1+\frac{2r}{Z\alpha^{2}}\right)}^{2}}$$

where *Z* is the nuclear charge and α is the fine structure constant. Equations 75–77 are the basis for computing hyperfine couplings in CP2K.^188^ In addition to these two first-order terms, there is also a second-order spin–orbit contribution to the hyperfine coupling, which can become important for heavy atoms.

It is obvious that the isotropic term particularly requires an accurate description of the electron density at the position of the nucleus; hence, hyperfine couplings are implemented within CP2K’s GAPW method.^188^ The Fermi contact term at atom *N* is simply computed by integrating eq 75 inside the local atomic domain *U*_*N*_ using the hard spin density (for detailed definitions of these quantities, see ref 25). The anisotropic hyperfine coupling is again split into a soft contribution to be computed in the PW basis and local spin-density contributions. The contribution of the local spin density of atom *N* itself is integrated inside the domain *U*_*N*_, since the total local density of any atom outside its domain is, by definition, zero. The local densities of other atoms *M* ≠ *N* make a small contribution to the hyperfine coupling of atom *N* (see Table 4 in ref 188). These “cross terms” needs to be integrated into the respective domains *U*_*M*_. For this small contribution, only near-neighboring atoms around *N* need to be included, using a cutoff distance with a default value of 10 Bohr, which the user can modify.

Unlike the magnetic properties discussed so far, the hyperfine coupling in terms of eqs 75–77 is a first-order property, obtained as an expectation value over the unperturbed spin density. Therefore, the input section for requesting the printing of the hyperfine coupling tensors is found directly under the DFT section via
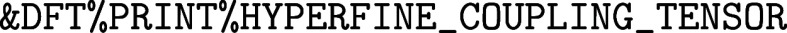


In addition to various print control options, the INTERACTION_RADIUS can be set inside this input section, which specifies the cutoff radius mentioned above.

## 6 Optical Spectroscopy

Optical spectroscopy is a technique that is used to study the interaction between light and matter. It involves measuring the absorption, emission, or scattering of light by molecules, atoms, or materials. The resulting spectra provide valuable information about the electronic structure, energy levels, and dynamics of the system under investigation. One of the key quantities of interest is the excitation energy Ω^(*n*)^, which is the energy required to excite a molecule from its ground state to an excited state. These excitation energies are directly related to the positions and intensities of spectral lines observed in absorption and emission spectra.

### 6.1 Linear-Response Time-Dependent Density Functional Theory

Optical properties are calculated by solving a generalized eigenvalue problem that involves the block matrix *ABBA*,^189^ hence

78 $$\left(\begin{array}{ll} A & B\\ B & A \end{array}\right)\left(\begin{array}{l} X^{\left(n\right)}\\ Y^{\left(n\right)} \end{array}\right)=\Omega^{\left(n\right)}\left(\begin{array}{ll} 1 & 0\\ 0 & -1 \end{array}\right)\left(\begin{array}{l} X^{\left(n\right)}\\ Y^{\left(n\right)} \end{array}\right)$$

Therein, *A* and *B* are matrices with indices *A*_*ia*,*jb*_, i.e. they have *N*_occ_ *N*_empty_ rows and *N*_occ_ *N*_empty_ columns. The eigenvector (**X** ^(*n*)^, **Y** ^(*n*)^) has elements *X*_*ia*_^(*n*)^ and *Y*_*ia*_^(*n*)^, which quantify the contribution of the transition from occupied orbital *i* to an empty orbital *a* in the excitation *n*. The matrix elements of **A** and **B** are

79 $$\begin{array}{l} A_{ia,jb}=\delta_{ij}\delta_{ab}\left(\epsilon_{a}-\epsilon_{i}\right)+\left(ia|jb\right)+\left(ia\left|f_{xc}\right|jb\right)\\ B_{ia,jb}=\left(ia|bj\right)+\left(ia\left|f_{xc}\right|bj\right) \end{array}$$

respectively.

The Hermitian matrix **A** includes, as a zeroth-order term, the differences within the KS orbital energies. At first order, it incorporates kernel contributions, which vary depending on the chosen density functional approximation. These contributions consist of Coulomb and exact exchange terms, as well as components arising from the XC potential and its associated kernel *f*_xc_. The implementation in CP2K/Quickstep is based on the Tamm–Dancoff approximation (TDA),^190,191^ which involves constraining **B** = 0 and **Y** = 0 in eq 79, thus resulting in the Hermitian eigenvalue problem

80 AX=ΩX

The TDDFT module facilitates the calculation of excitation energies and excited state properties within the TDA, supporting both GGA and hybrid XC functionals, as well as simplified semiempirical TDA kernels. The optical spectrum calculation with TDDFT requires setting &GLOBAL%RUN_TYPE ENERGY and specifying the subsequent &TDDFPT subsection within &FORCE_EVAL%PROPERTIES as follows:
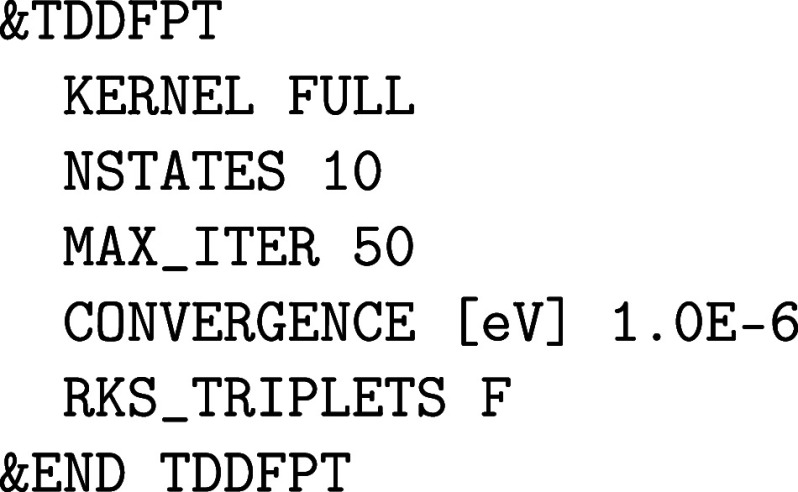


The kernel matrix is controlled by the KERNEL keyword, where one can select between the full kernel that is appropriate for GGA or hybrid functional computations, or the semiempirical sTDA kernel.^192,193^ If the FULL kernel is selected, the underlying functional can be specified via the &XC section. The choice of XC functional can differ from the ground state functional unless the ADMM method is employed. The sTDA kernel omits XC contributions and approximates both Coulomb and exchange terms using pairwise semiempirical operators γ^J^ and γ^K^, which are dependent on the interatomic distance *R*_*MN*_ between atoms *M* and *N*. Hence

81 $$\begin{array}{ll} \gamma^{J}\left(M,N\right) & ={\left(\frac{1}{{R_{MN}}^{\alpha }+\eta^{-\alpha }}\right)}^{1/\alpha }\\ \gamma^{K}\left(M,N\right) & ={\left(\frac{1}{{R_{MN}}^{\beta }+{\left(a_{XC}\eta \right)}^{-\beta }}\right)}^{1/\beta } \end{array}$$

are relying on the four global parameters, the chemical hardness η, the Fock-exchange mixing parameter *a*_XC_ and the powers α for Coulomb, and β for the exchange interactions. The proportion of exact exchange, controlled by adjusting the parameter *a*_XC_, is critical for a well-balanced treatment of exact-exchange in the ground and excited state potential surfaces. This is controlled by the keyword FRACTION in the &sTDA section, and it is recommended to use a relatively small amount, e.g. *a*_XC_ = 0.1–0.2. The parameters α and β are controlled by the MATAGA_NISHIMOTO_CEXP and MATAGA_NISHIMOTO_XEXP keywords, respectively.
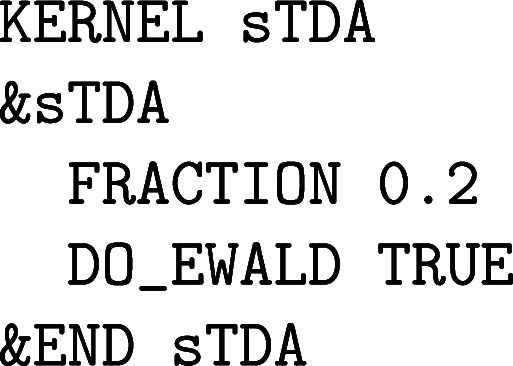


It should be noted that the definition of the operators in eq 81 differs from the original method for compatibility with the ADMM definition. For periodic systems, the Ewald summation for Coulomb contributions should be enabled with the DO_EWALD keyword, which also activates the &DFT%POISSON section.

Additional key parameters include NSTATES, which defines the number of excitation energies to compute, and CONVERGENCE that sets the convergence threshold for the Davidson algorithm. The RKS_TRIPLETS option allows switching from the default singlet excitation energy calculations to triplet excitations. The RESTART keyword enables restarting a TDDFT calculation if a valid restart file (.tdwfn) is available. Furthermore, a separate grid can be defined for real-space integration in the TDDFT calculation via the &TDDFPT%MGRID subsection, though a consistent setup for ground and excited-state calculations is generally recommended.

Upon convergence of the Davidson algorithm, CP2K outputs the excitation energies in eV, the transition dipole moments, and the oscillator strengths for each calculated excited state. The &DIPOLE_MOMENTS%DIPOLE_FORM keyword allows modifying the form of the dipole transition integrals, with options such as BERRY (for fully periodic systems), LENGTH (for molecular systems), and VELOCITY (for both). When using the length form, the reference point for calculating dipole moments can be adjusted through the REFERENCE keyword, allowing for selection between COM (center of mass), COAC (center of atomic charges), USER_DEFINED (user-specified coordinates), or ZERO (origin of the coordinate system). If USER_DEFINED is selected, the keyword REFERENCE_POINT must be defined in terms of the atomic coordinates.

Natural transition orbitals (NTOs) can be printed by setting PRINT_LEVEL to medium in the &GLOBAL section or by enabling it in &PRINT%NTO_ANALYSIS. For an NTO analysis, unoccupied orbitals must be generated, whose number included in the analysis is adjusted using the LUMO keyword. One can specify a list of states for which NTOs should be printed using the STATE_LIST keyword or apply a threshold for the screening of the states based on their oscillator strengths with the INTENSITY_THRESHOLD keyword. NTOs can be outputted as CUBE_FILES or in Molden format, the latter being controlled by the MOS_MOLDEN keyword.

The excited-state gradients can be calculated by setting RUN_TYPE ENERGY_FORCE in the &GLOBAL section. The specific excited state for which the gradients will be computed must be defined by adding the &EXCITED_STATES subsection within the &DFT section, e.g.:
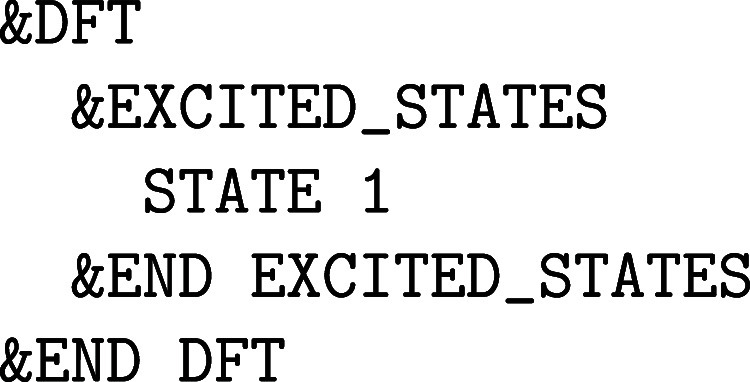


The parameters for the gradient calculation can be adjusted in the &TDDFPT%LINRES subsection, allowing to specify the PRECONDITIONER for the optimization and MAX_ITER for the maximum number of iterations. Similarly, the fluorescence energy can be calculated by optimizing the first excited state. This is achieved by setting &GLOBAL%RUN_TYPE GEO_OPT and defining the &MOTION%GEO_OPT subsection, and specifying the reference excited state as outlined above. Additionally, support for excited-state gradient calculations using the GAPW method has recently been introduced.^194^

Furthermore, SOC can be taken into account by adding a perturbative correction via the &PROPERTIES%BANDSTRUCTURE%SOC subsection.^195^ As triggered by the keyword RKS_TRIPLETS, the TDA eigenvalue problem is thereby solved for both singlet and triplet closed-shell references, yielding the corresponding excited state manifolds with excitation amplitudes **X**_TDA_^(*n*)^, and thereon-based, auxiliary many-electron WFs are generated, with the manifolds $|\Psi_{S,m_{S}}^{\left(n\right)}\rangle$ being here labeled according to the total spin of the system *S* and the corresponding spin angular momentum quantum number *m*_S_. Using quasi-degenerate perturbation theory, the unperturbed excitation energies $\Omega_{S,m_{S}}^{\left(n\right)}$ , as obtained from eq 78, are corrected by adding an additive correction based on a one-electron SOC Hamiltonian ${ĥ}^{\text{SO}}$ , so that

HS,mS,S′,mS′(nm)=δ(nm)δSS′δmSmS′ΩS,mS(n)⁣⁣⁣(82)+⟨ΨS,mS(n)|ĥSO|ΨS′,mS′(m)⟩⁣⁣⁣(83)

Two choices are available for ${ĥ}^{\text{SO}}$ , however, for all-electron GAPW computations, van Wüllen’s model potential based on the zeroth-order regular approximation (ZORA) is chosen by default, requiring no change in the CP2K input.^196,197^ Restricting the description to valence electrons, a SOC-corrected PP can be used,^32^ as described in Subsection [Sec sec3], by specifying GTH_SOC_POTENTIALS as the &DFT%POTENTIAL_FILE_NAME in the CP2K input file. To print the resulting SOC-corrected energies, spin–orbit matrix elements as defined in eq 83, and oscillator strengths, the following &SOC_PRINT subsection must be added to the &TDDFPT%PRINT section of TDDFT:
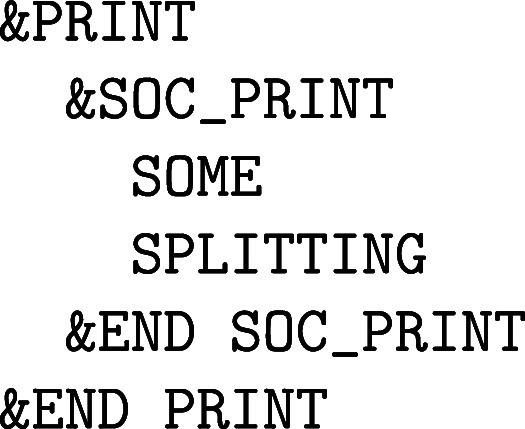


Adding the keywords UNIT_EV or UNIT_WN furthermore determines whether the chosen unit of the printout is in eV or cm^–1^, respectively.

### 6.2 Bethe–Salpeter Equation

The BSE is a method for computing electronic excitation energies Ω^(*n*)^, *n* = 1, 2, ... and optical absorption spectra.^198–203^

#### 6.2.1 Electronic Excitation Energies

For a closed-shell system, the entries of *A* and *B* of eq 78 are

84 $$\begin{array}{l} A_{ia,jb}=\left(\varepsilon_{a}^{GW}-\varepsilon_{i}^{GW}\right)\delta_{ij}\delta_{ab}+\alpha^{\left(S/T\right)}v_{ia,jb}-W_{ij,ab}\\ B_{ia,jb}=\alpha^{\left(S/T\right)}v_{ia,bj}-W_{ib,aj} \end{array}$$

where δ_*ij*_ is the Kronecker delta, *v*_*pq*,*rs*_ the bare Coulomb interaction and *W*_*pq*,*rs*_ the statically screened Coulomb interaction, with *p*, *q*, *r*, *s* being KS orbital indices. The user needs to set α^S^ = 2 for computing singlet excitations and α^T^ = 0 for computing triplet excitations.^198,204^

A BSE calculation requires occupied KS orbitals φ_*i*_(**r**) and empty KS orbitals φ_*a*_(**r**) from a DFT calculation, where *i* = 1, ..., *N*_occ_ and *a* = *N*_occ_ + 1, ..., *N*_occ_ + *N*_empty_, as well as the *GW* eigenvalues ε_*i*_^*GW*^ and ε_*a*_^*GW*^, respectively. In CP2K/Quickstep, it is possible to use *G*_0_ *W*_0_, ev*GW*_0_ or ev*GW* eigenvalues, see details in the *GW* section and in ref 119. Thus, also *GW* and DFT input parameters might have an impact on BSE excitation energies Ω^(*n*)^. We recommend calculating the BSE based on ev*GW*_0_@PBE eigenvalues,^35^ as discussed in ref 125.

The TDA constrains *B* = 0, such that Ω_TDA_^(*n*)^ and **X**_TDA_^(*n*)^ can be computed from the Hermitian eigenvalue problem

85 $$AX_{TDA}^{\left(n\right)}=\Omega_{TDA}^{\left(n\right)}X_{TDA}^{\left(n\right)}$$

Diagonalizing *A* in the TDA of eq 84, or the full block-matrix *ABBA* of eq 78 takes on the order of ${\left(N_{occ}N_{empty}\right)}^{3}$ floating-point operations. This translates into a computational scaling of *O*(*N* ^6^) for a BSE calculation of system size *N*.

The eigenvectors (**X** ^(*n*)^, **Y** ^(*n*)^) of eq 78 with elements *X*_*ia*_^(*n*)^ and *Y*_*ia*_^(*n*)^ enter the WF of the electronic excitation^203,205^

86 $$\Psi_{excitation}^{\left(n\right)}\left(r_{e},r_{h}\right)=\sum_{ia}X_{ia}^{\left(n\right)}\varphi_{i}\left(r_{h}\right)\varphi_{a}\left(r_{e}\right)+Y_{ia}^{\left(n\right)}\varphi_{i}\left(r_{e}\right)\varphi_{a}\left(r_{h}\right)$$

i.e. *X*_*ia*_^(*n*)^ and *Y*_*ia*_^(*n*)^ describe the transition amplitude between an occupied orbital φ_*i*_ and an empty orbital φ_*a*_ for the *n*-th excitation. In TDA, we can interpret the excitation in terms of electrons and holes as follows: an electron leaves a hole behind in the (formerly) occupied orbital φ_*i*_(**r**_h_) and is afterward located in the empty orbital φ_*a*_(**r**_e_).

#### 6.2.2 Optical Absorption Spectrum

Within the BSE framework, we can compute the photoabsorption cross-section tensor σ_μ,μ′_(ω) with (μ, μ′ ∈ {*x*, *y*, *z*}) as^206,207^

87 $$\sigma_{\mu ,\mu^{\prime }}\left(\omega \right)=-\frac{4\pi \omega }{c}Im\left[\sum_{n}\frac{2\Omega^{\left(n\right)}d_{\mu }^{\left(n\right)}d_{\mu^{\prime }}^{\left(n\right)}}{{\left(\omega +i\eta \right)}^{2}-{\left(\Omega^{\left(n\right)}\right)}^{2}}\right]$$

with broadening η and the transition moments of the Singlet solution given by^202^

88 $$d_{\mu }^{\left(n\right)}=\sqrt{2}\sum_{i,a}\left\langle \varphi_{i}\left|\mathrm{\mu ̂}\right|\varphi_{a}\right\rangle \left(X_{ia}^{\left(n\right)}+Y_{ia}^{\left(n\right)}\right)$$

When the molecules are not aligned, e.g. for gas phase and liquids, we can compute the photoabsorption cross section from the spatial average of eq 86

89 σ̅(ω)=13∑μ∈{x,y,z}σμ,μ(ω)=−4πωcIm[∑nf(n)(ω+iη)2−(Ω(n))2]

where we have introduced the oscillator strengths^202^

90 $$f^{\left(n\right)}=\frac{2}{3}\Omega^{\left(n\right)}\sum_{\mu \in \left\{x,y,z\right\}}{\left|d_{\mu }^{\left(n\right)}\right|}^{2}$$

#### 6.2.3 Measures for the Size of an Excited State

The WF of an excited state Ψ_excitation_^(*n*)^(**r**_e_,**r**_h_) of eq 85 and its spatial properties can be analyzed by introducing measures for its spatial extent following ref 205. To that end, we define the corresponding expectation value for a generic operator

91 $${\left\langle Ô\right\rangle }_{exc}^{\left(n\right)}=\frac{\left\langle \Psi_{excitation}^{\left(n\right)}\left|Ô\right|\Psi_{excitation}^{\left(n\right)}\right\rangle }{\left\langle \Psi_{excitation}^{\left(n\right)}|\Psi_{excitation}^{\left(n\right)}\right\rangle }$$

For example, we can compute the distance between the electron and the hole as

92 $$d_{h\to e}=\left|{\left\langle r_{h}-r_{e}\right\rangle }_{exc}\right|$$

This can be used to characterize a charge-transfer state, where the electron and hole sit on different parts of the molecule and therefore *d*_h→e_ is nonvanishing.

Further, we can compute the exciton size

93 $$d_{exc}=\sqrt{{\left\langle |r_{h}-r_{e}{|}^{2}\right\rangle }_{exc}}$$

which quantifies the spatial extent of the combined electron–hole pair. It increases when the distance between the electron and hole *d*_h→e_, or their respective sizes, increases.

A typical BSE calculation starts with RUN_TYPE ENERGY and the following prototypical *GW*-BSE section:
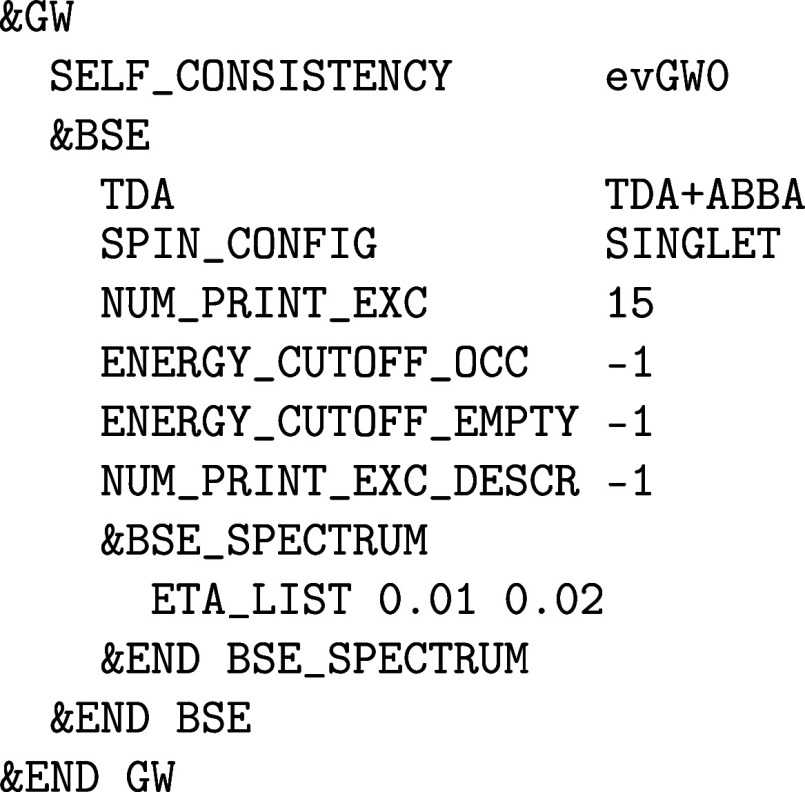


Therein, the following keywords are available:

- SELF_CONSISTENCY: determines which *GW* self-consistency (*G*_0_ *W*_0_, ev*GW*_0_ or ev*GW*) is used to calculate the single-particle *GW* energies ε_*p*_^*GW*^ necessary in the BSE calculation of eq 83. We recommend using evGW0 for BSE runs.^125^
- TDA: specifies if the TDA and/or diagonalization of the full ABBA-matrix is employed. OFF: Generalized diagonalization of *ABBA* of eq 78. ON: use the TDA of eq 84 and diagonalize only *A*. TDA+ABBA: compute excitation energies Ω^(*n*)^ and Ω_TDA_^(*n*)^ from eqs 78 and 84, respectively.
- SPIN_CONFIG: choose between SINGLET for computing singlet excitation energies (α^S^ = 2) and TRIPLET for computing triplet excitation energies (α^T^ = 0). The standard is SINGLET as an electronic excitation directly after photoexcitation is a singlet due to angular momentum conservation; triplet excited states can form by intersystem crossings.
- NUM_PRINT_EXC: number of excitation energies Ω^(*n*)^ to be printed.
- ENERGY_CUTOFF_OCC (in eV): only use indices *i* of occupied MOs in the interval ε_*i*_^DFT^ ∈ $\left[\varepsilon_{HOMO}^{DFT}-ENERGY\text{\_}CUTOFF\text{\_}OCC,\varepsilon_{HOMO}^{DFT}\right]$ to set up the matrices *A* and *B* in eq 83. A small ENERGY_CUTOFF_OCC reduces computation time and memory consumption, but can affect the computed excitation energies Ω^(*n*)^. Usage of ENERGY_CUTOFF_OCC is recommended for molecules with more than 30 atoms. We recommend a convergence test by increasing ENERGY_CUTOFF_OCC and observing the effect on Ω^(*n*)^.^202^
- ENERGY_CUTOFF_EMPTY (in eV): analogously to ENERGY_CUTOFF_OCC, but for the empty states, i.e. only empty states in the interval ε_*i*_^DFT^ ∈ $\left[\varepsilon_{LUMO}^{DFT},\varepsilon_{LUMO}^{DFT}+ENERGY\text{\_}CUTOFF\text{\_}EMPTY\right]$ are used in the BSE calculation.
- NUM_PRINT_EXC_DESCR: number of excitations *n*, for which the exciton descriptors are printed, e.g. *d*_exc_^(*n*)^ of eq 92.
- BSE_SPECTRUM: Activates the computation and printing of the photoabsorption cross section tensor, i.e. its spatial average σ̅(ω) of eq 88 and its elements σ_μ,μ′_(ω) (μ, μ′ ∈ {*x*, *y*, *z*}) of eq 86 are printed.

In addition to the upper *GW*-BSE keywords, the settings within the &DFT section influence the BSE excitation energies Ω^(*n*)^:

- XC_FUNCTIONAL: choose between one of the available XC functionals. The starting point can have a profound influence on the excitation energies.^208^ Motivated by the discussion in ref 125, we strongly recommend to use BSE@ev*GW*_0_@PBE, i.e. the PBE functional^35^ as the DFT starting point.
- BASIS_SET: the all-electron aug-cc-pVDZ basis set^44,209^ should be sufficient for most organic molecules, but needs to be checked with respect to convergence.

The memory consumption of the BSE method is large, i.e. approximately $100N_{occ}^{2}N_{empty}^{2}$ Bytes. The estimated memory consumption, *N*_occ_, as well as *N*_empty_ are all printed in the BSE output. The BSE implementation is very well parallelized, i.e. one can use several nodes that can also provide the required memory. We have compared the excitation energies from the BSE implementation in CP2K against the FHI-aims code^202^ and found excellent agreement on the order of 5 meV mean absolute error for the first 10 excitations of Thiel’s data set.^210^

## 7 Excited State Dynamics

In RT-TDDFT, instead of solving the static SE, the aim is to solve the time-dependent SE

94 $$i\frac{\partial }{\partial t}\Psi \left(r,t\right)=Ĥ\left(r,t\right)\Psi \left(r,t\right)$$

Contrary to the time-independent case, there is no variational principle for the total energy, and the total energy has to be replaced by the quantum mechanical action

95 $$A\left[\Psi \right]=\int_{t_{0}}^{t_{f}}dt\left\langle \Psi \left(t\right)\left|i\frac{\partial }{\partial t}-Ĥ\left(t\right)\right|\Psi \left(t\right)\right\rangle$$

for which the function Ψ­(*t*) that makes the action stationary will be its solution.

### 7.1 Real-Time Propagation and Ehrenfest Dynamics

The resulting time-dependent KS equations read as

96 $$i\frac{\partial }{\partial t}\psi_{i}\left(r,t\right)=\left[-\frac{\nabla^{2}}{2}+v_{KS}\left(r,t\right)+F\left(t\right)\right]\psi_{i}\left(r,t\right)$$

with ψ_*i*_ being the KS orbitals

97 $$\rho \left(r,t\right)=\sum_{i}f_{i}{\left|\psi_{i}\left(r,t\right)\right|}^{2}$$

the time-dependent density, and **F**(*t*) an optional time-dependent external field. By applying an external electric field, we intend to simulate the interaction between the electrons and an external radiation. The light is then treated classically using either the length-gauge for nonperiodic systems or the velocity-gauge for periodic systems.

A rather general derivation for (nonadiabatic quantum-classical) Ehrenfest dynamics can be obtained starting from the action of a system.^211^ For a situation in which the electrons are treated quantum mechanically, while the nuclei are treated classically, the total action can be written as the sum of the two environments *A* = *A*_c_ + *A*_q_, where

98 $$A_{c}=\int_{t_{0}}^{t_{f}}dt\left[\sum_{A}\frac{M_{A}}{2}{Ṙ}_{A}-U\left(R,t\right)\right]$$

and *A*_q_ the quantum mechanical action as defined above. The equations of motion are then derived by making the action stationary

99a $$\frac{\delta A}{\delta \langle \Psi \left(r,t\right)|}=0$$

99b $$\frac{\delta A}{\delta \langle R\left(t\right)|}=0$$

Evaluating these expressions in the framework of TDDFT, the equations of motion become

100 $$M\mathrm{R̈}=-\frac{\partial }{\partial R}U\left(R,t\right)-\sum_{j}\left\langle \Psi^{j}\left|\frac{\partial }{\partial R}V_{\mathrm{int}}\left(r,R\right)\right|\Psi^{j}\right\rangle$$

for the nuclear motion, while the time-dependent Schrödinger equation as given above is used for the electrons. Yet, in a numerical calculation, the WFs are often replaced by a finite basis set representation in terms of the LCAO. For PWs, the equations remain the same, since they do not depend on the nuclear coordinates. Using Gaussian basis functions for the expansion of the WFs, however, introduces an implicit dependence of the WFs on the nuclear positions. Since in AIMD the nuclear coordinates are a function of time, the time-derivative in the quantum mechanical action has to be replaced by the total time-derivative

101 $$\frac{d}{dt}=\frac{\partial }{\partial t}+\sum_{A}\frac{\partial R_{A}}{\partial t}\frac{\partial }{\partial R_{A}}$$

Due to the introduction of a finite basis, the independent variables for making the action constant become the expansion coefficients *C*_*j*α_(*t*) of the MO *j* in the contracted basis function ϕ_α_(**r**, *t*), i.e.

$${Ċ}_{j\alpha }=-\sum_{\alpha \beta }S_{\beta \gamma }^{-1}\left(iH_{\beta \gamma }+B_{\beta \gamma }\right)C_{j\gamma }$$

where

$$S_{\alpha \beta }=\left\langle \phi_{\alpha }|\phi_{\beta }\right\rangle ,B_{\alpha \beta }=\left\langle \phi_{\alpha }|\frac{d}{dt}\phi_{\beta }\right\rangle$$

Hence, in Ehrenfest dynamics an additional contribution due to the derivative of the basis functions becomes part of the Hamiltonian used in the exponential operator. Instead of being purely imaginary, the matrix in the exponential of the propagator becomes fully complex.

To run real-time propagation (RTP), where the nuclei are fixed and only the electronic degrees of freedom are propagated, or genuine Ehrenfest dynamics, the corresponding RUN_TYPE in the &GLOBAL section has to be set to RT_PROPAGATION or EHRENFEST_DYN, respectively. Furthermore, the &MOTION%MD section has to be present to specify the discretized TIMESTEP and the desired number of STEPS. It is crucial to set an appropriate time step to propagate the electronic degrees of freedom, which is much smaller than for MD and is typically on the order of attoseconds. All other input parameters related to the RT-TDDFT run are specified in the &DFT%REAL_TIME_PROPAGATION section. The simulation needs to start from the electronic density at *t*_0_ specified by the keyword INITIAL_WFN. This can be either the ground state obtained by means of an initial SCF optimization denoted by SCF_WFN, or by providing the restart file of a previously computed WF via RESTART_WFN. Alternatively, the propagation can also be restarted from a RTP or Ehrenfest dynamics run by RT_RESTART and providing the correct restart file. Note that the RTP restart file has a format different from the SCF restart file. The path to the restart file is in both cases provided by the usual &DFT%RESTART_FILE_NAME keyword.

Three different propagators are available in CP2K, the enforced time-reversible symmetry propagator (ETRS), the exponential midpoint (EM) propagator, and the Crank-Nicholson propagator, which can be seen as a first-order Padé approximation of the EM propagator.^212^ The ETRS approach starts with an exponential approximation to the evolution operator Û(t,0)=exp{−itĤ(0)} , to then compute the final time-reversible and unitary propagator self-consistently. In the RTP scheme (fixed ionic positions), the self-consistent solution involves only the calculation of the new KS matrix for the propagated coefficients. For Ehrenfest dynamics, the iterative procedure is embedded in the integrator of the nuclear equations of motion. Hence, each iteration step for Ehrenfest dynamics involves an RTP step and the evaluation of the nuclear forces. Due to this, more iterations are needed to reach self-consistency for the propagator. The convergence criterion implemented in CP2K is defined as

102 $${∥\Delta C^{T}S\Delta C∥}_{\max}<\epsilon$$

with Δ*C* being the difference of coefficient matrices in two successive steps, and ϵ given by EPS_ITER.

In terms of computational cost, the most expensive part is the evaluation of the matrix exponential in the propagator. Four different methods, among those listed in ref 213, have been implemented in CP2K, i.e. diagonalization, the Taylor expansion, the Padé approximation, and the Arnoldi subspace iteration. The method is selected using the MAT_EXP keyword. The Arnoldi method often provides superior performance. Comparing the theoretical scaling, the Arnoldi method is expected to be about 5 times faster than the Padé or Taylor approaches. However, the Padé approximation can sometimes be a faster and more stable choice than the Arnoldi method (e.g., in the case of large integration timesteps). Since the propagation is based on an iterative procedure, it is convenient to apply an extrapolation scheme to speed up convergence.^11^ For Ehrenfest dynamics, an extrapolation based on the product of the density and the overlap matrix turns out to be a good choice. However, the quality of the different extrapolation approaches strongly depends on the system applied and the simulation settings. Therefore, which method to use and the extrapolation order need to be tested on the particular system of interest. For very large systems, the density matrix-based method can be used to achieve linear scaling by activating the keyword DENSITY_PROPAGATION.^214^

One of the most relevant application domains for RT-TDDFT is the study of light–matter interactions, e.g. in the field of spectroscopy, excited state dynamics, and radiation damage. To mimic these phenomena, at any time during the propagation, it is possible to apply a time-dependent electric field **F**(*t*). The applied field is in general modulated by an envelope function *E*_env_(*t*) and is defined as

103 $$E\left(t\right)=PE_{env}\left(t\right)\cos\left(\omega_{0}t+\phi \right)$$

where **P** is the field polarization, ω_0_ is the carrying frequency at which the field oscillates, and ϕ its initial phase. The characteristic of the applied field, as well as its time extension, is provided through the section &DFT%EFIELD. The time-dependent electric field defined within this section can only be used in combination with RT-TDDFT. By default, the coupling between the electric field and the electronic degrees of freedom is described within the length-gauge, by adding to the Hamiltonian the dipole coupling term *e* **E**(*t*)·**r**. This approach is only valid for isolated molecular systems, and not when periodic boundary conditions are applied. The velocity-gauge form of the equations suitable for periodic systems is obtained through a gauge transformation involving the vector potential^215–217^

104 $$A\left(t\right)=c\int^{t}dt^{\prime }E\left(t^{\prime }\right)$$

In the time-dependent KS equations, the vector potential **A**(*t*) appears in the kinetic energy term and, in the case where nonlocal PPs are used, the gauge field also transforms the electron–ion interaction. To use this representation, the VELOCITY_GAUGE keyword has to be used within the aforementioned REAL_TIME_PROPAGATION section. The total energy varies over time as the applied external field interacts with the system. To monitor the time evolution of the field and the various terms that contribute to the total electronic energy, which can be activated via the &REAL_TIME_PROPAGATION%PRINT print key.

Once the RTP has started, the evolution of the electronic structure can be monitored by means of several descriptors, such as the time-dependent dipole moment, the time-dependent total electronic density, and the spin density, which can be printed out as a series of cube files for every arbitrary number of propagation steps. As for ground state calculations, the output of these quantities is activated by the corresponding &DFT%PRINT print key.

The electron current density can be obtained as

105 $$j\left(r,t\right)=\frac{1}{2}\sum_{i}f_{i}\left(\psi_{i}^{*}\left(r,t\right)\pi \psi_{i}\left(r,t\right)+c.c.\right)$$

where

106 $$\pi =\left[r,H\right]=-i\nabla +A\left(t\right)+\left[V_{PP},r\right]$$

Integrating **j**(**r**, *t*) in the simulation cell yields the macroscopic current

107 $$J\left(t\right)=-\frac{1}{V}\int drj\left(r,t\right)$$

where *V* is the cell volume. The print key &REAL_TIME_PROPAGATION%PRINT%CURRENT_INT activates the calculation and the output of the macroscopic current. This quantity can then be used to calculate the frequency-dependent conductivity, which is defined as the ratio of the current’s Fourier transform to that of the applied electric field

108 $$\sigma_{ij}\left(\omega \right)=\frac{F\left[J_{i}\right]\left(\omega \right)}{F\left[E_{j}\right]\left(\omega \right)}$$

The projection of the time-dependent orbitals onto some previously selected reference states is particularly useful for verifying the variation in population of specific states, while monitoring density differences is usually helpful for detecting charge-transfer processes. By projecting time-dependent MOs on some reference states (e.g., the initial MOs) using the print key &PRINT%PROJECTION_MO the overlap between the propagated orbital ψ_*i*_(**r**, *t*) = ∑_α_ *C*_*i*α_(*t*)­ϕ­(**r**) and any reference orbital ψ_*m*_^ref^ is calculated. When the reference orbitals are the ground state virtual orbitals, the quantity

$$N_{exc}\left(t\right)=\sum_{m}^{unocc}\sum_{i}^{occ}{∥\left\langle \psi_{m}^{ref}|\psi_{i}\left(t\right)\right\rangle ∥}^{2}$$

is an estimate of the number of electrons that have been excited into the unoccupied space.

### 7.2 Real-Time Bethe−Salpeter Propagation

Although the exact solution of time-dependent KS equations of eq 95 can describe the nonlinear-response of a system to an external field *F̂*(*t*), using approximate XC kernels often leads to inaccuracies. For instance, the adiabatic LDA fails to describe excitonic energies accurately.^203,206,218^

Hence, choosing an approximate self-energy operator is more suitable for the description of nonlocal screening effects present in the excitonic spectra.^218^ In the RT-BSE scheme implemented in CP2K,^219^ the Coulomb-hole plus screened-exchange (COHSEX) self-energy is employed.^51,220–223^ Moreover, instead of state propagation, solution of the equation of motion for the single-particle density matrix ρ̂(*t*)­

109 $$\frac{\partial \mathrm{\rho ̂}}{\partial t}=\frac{-i}{\hbar }\left[{Ĥ}^{eff}\left(t\right),\mathrm{\rho ̂}\left(t\right)\right]$$

is implemented, where the effective Hamiltonian is given by^220^

110 Ĥeff(t)=ĤG0W0+Û(t)+V̂Hartree[ρ̂(t)]−V̂Hartree[ρ̂(t0)]+Σ̂COHSEX[ρ̂(t)]−Σ̂COHSEX[ρ̂(t0)]

Therein, ${Ĥ}^{G_{0}W_{0}}$ is the *G*_0_ *W*_0_ Hamiltonian, *Û*(*t*) is the applied external field, ${\mathrm{V̂}}^{Hartree}\left[\mathrm{\rho ̂}\left(t\right)\right]$ is the Hartree potential (which depends on the single-particle density matrix) and ${\mathrm{\Sigma ̂}}^{COHSEX}\left[\mathrm{\rho ̂}\left(t\right)\right]$ is the COHSEX self-energy. This form of the effective Hamiltonian leads to oscillations of ρ̂(t) formally equivalent to solving the Casida equation of eq 78 with Bethe–Salpeter entries shown in eq 83,^220^ hence the name of the method.

In order to run a RT-BSE calculation (so far only implemented for molecules, i.e. without PBC, and for static nuclei), a similar setup as for running an RT-TDDFT propagation calculation is employed, namely

- set RUN_TYPE to RT_PROPAGATION
- include a &MD section to set the size of the TIMESTEP and the total number of STEPS
- specify all options necessary to run an accurate *G*_0_ *W*_0_ calculation for molecules (in particular the subsection &PROPERTIES%BANDSTRUCTURE%GW should be specified)
- include the &REAL_TIME_PROPAGATION section with all relevant entries and add subsection &RTBSE.

Note that the RT-BSE method employs the RI approximation for the Hartree and COHSEX terms,^127^ so an appropriate RI_AUX basis should be set. Example RT-BSE calculations for organic molecules are also available in repositories of the RT-BSE implementation paper.^219^ A typical RT-BSE input with the most important &REAL_TIME_PROPAGATION section will look as follows:
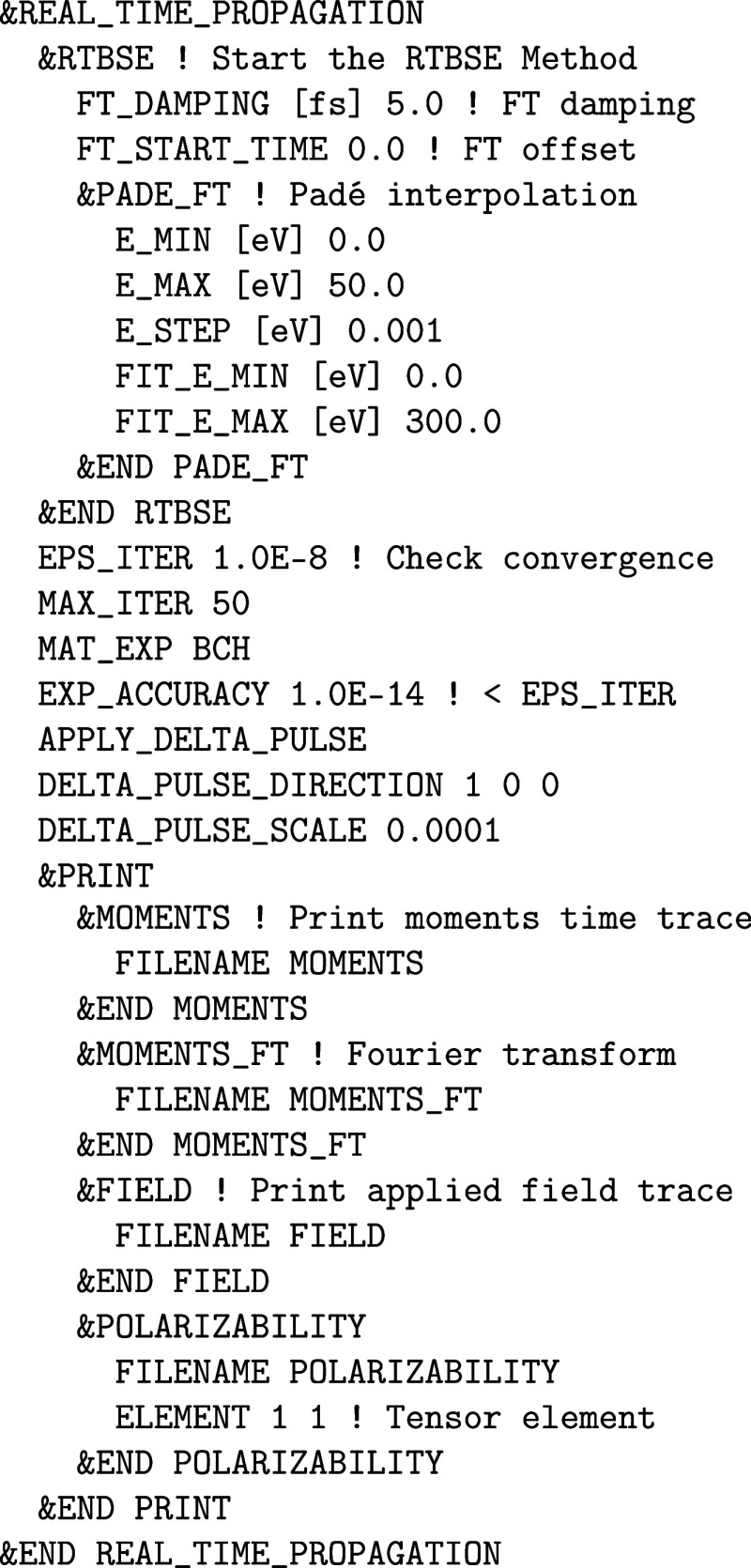


The most relevant keywords and subsections in the above section entail:

- EPS_ITER: the equation of motion of eq 109 is solved by exponential evolution operators applied to the single-particle density matrix ρ̂. These are applied self-consistently by an enforced time reversal symmetry scheme described previously,^212,214,224^ so that

111 ρ̂(t+Δt)≈e−i/ℏĤeff(t+Δt)Δt/2×e−i/ℏĤeff(t)Δt/2ρ̂(t)×ei/ℏĤeff(t)Δt/2×ei/ℏĤeff(t+Δt)Δt/2

The self-consistency is present since the effective Hamiltonian ${Ĥ}^{eff}\left(t+\Delta t\right)$ depends on the single-particle density matrix at time *t* + Δ*t*, to which we need to propagate. The self-consistency threshold is controlled by EPS_ITER. Smaller threshold implies more stable propagation, but at the same time usually requires more iterations. Usually, one can choose EPS_ITER similar to EPS_SCF, although a larger tolerance might be sufficient for consistent results - one should test the convergence of EPS_ITER and TIMESTEP pair. Note that the maximum among the absolute values of elements of ρ̂(t+Δt)−ρ̂(t) in the AO basis is used as the metric for the threshold.

- MAX_ITER: limits the maximum number of the self-consistent iterations in the ETRS scheme.
- MAT_EXP: the matrix exponentiation in RT-BSE is implemented by two approaches—the (Baker–Campbell–Hausdorff) BCH^212^ and the EXACT diagonalization. The keyword MAT_EXP controls the choice between these two.
- EXP_ACCURACY: for the BCH method, it is also necessary to specify the matrix exponentiation accuracy, which should be stricter than the ETRS threshold EPS_ITER.
- APPLY_DELTA_PULSE: in order to observe any oscillations, the external field *Û*(*t*) needs to be specified. Either the explicit time-dependent field can be supplied in the &DFT%EFIELD subsection, or a delta pulse can be triggered by inclusion of this keyword.^225^
- DELTA_PULSE_DIRECTION: sets the Cartesian direction of the delta pulse. The resulting vector is always normalized by the code.
- DELTA_PULSE_SCALE: sets the magnitude of the delta pulse directly (in atomic units). The change in the metric of the single-particle density matrix after the application of the delta pulse is reported by the code - if the change is larger than 1 in atomic units, then the ETRS loop might struggle to converge—we recommend decreasing the DELTA_PULSE_SCALE in such cases.
- &MOMENTS: activates printing of the time-dependent dipole moment
112 $$\mu_{j}\left(t\right)=Tr\left(\mathrm{\rho ̂}\left(t\right)\left(\mathrm{r̂}-r_{CC}\right)\right)$$
  where μ_*j*_ is the *j*th component of the dipole moment μ­(*t*) and **r**_CC_ is the reference point—in RT-BSE, this is the center of atomic charges.
- FT_DAMPING: Damping γ applied during Fourier transform to stabilize it.^226^ Example is given in the &MOMENTS_FT section. If not given explicitly, γ is determined so that factor of e^–4^ is applied at the end of the time trace of the observables, starting from FT_START_TIME.
- FT_START_TIME: offset along the time axis for the purposes of Fourier transform. Defaults to zero. Useful for real-time pulses defined in &EFIELD section. For example, for GAUSSIAN_ENV with nonzero T0 parameter, setting FT_START_TIME to the same value will result in the envelope being a real function in frequency space.
- &MOMENTS_FT: activates printing of the Fourier transform of the dipole moment **μ**(*t*), which is stabilized by the application of FT_DAMPING γ, so that
113 $$\mu_{j}\left(\omega \right)=\int dte^{i\left(\omega +i\gamma \right)t}\mu_{j}\left(t+t_{0}\right)$$
  where *t*_0_ is the defined by the START_TIME.
- &FIELD: activates printing of the time-dependent trace of the external field. When no &EFIELD subsection within the &DFT section is present, outputs just zeros (for example when only the delta pulse is applied.)
- &POLARIZABILITY: activates printing of the polarizability tensor, defined in terms of the Fourier transform of the dipole moment and the Fourier transform of the electric field as^227,228^
114 $$\alpha_{jk}\left(\omega \right)=\frac{\mu_{j}\left(\omega \right)}{E_{k}\left(\omega \right)}$$

The polarizability is related to the dipole strength function,^227,228^ which characterizes the absorption spectrum of the system. Control over which element is printed is given by the ELEMENT keyword. The polarizability calculation also uses the settings from the &MOMENTS_FT section.

- &PADE_FT: section controlling the Padé interpolation^229–231^ of the Fourier transforms. A new frequency grid spanning from E_MIN to E_MAX with E_STEP steps is created, on which the Fourier transforms are reevaluated using the Padé fitting of the original transforms. The original transforms are restricted to energies within FIT_E_MIN and FIT_E_MAX. By default, no restriction is applied—entire frequency range of the original transform is used to construct the Padé parameters.

The code automatically saves the state of the system after each successful time step, so that the propagation can be continued after interruption. Use INITIAL_WFN RT_RESTART in the REAL_TIME_PROPAGATION section to enable the restart. Note that the total propagation time determines the energy resolution in the resulting Fourier transform, while the time step determines the maximum frequency/energy accessible to the Fourier transform. In other words, if a finished RT-BSE calculation does not yield the required energy resolution, it is possible to simply increase the number of STEPS and continue the propagation. Note that the &MOMENTS and &FIELD should not be suppressed (for example by an insufficient PRINT_LEVEL) in order for the program to be able to read the time traces from the previous run.

### 7.3 Surface Hopping via NEWTON-X

The interface with the NEWTON-X program package enables to perform on-the-fly nonadiabatic molecular dynamics (NAMD) simulations using Tully’s fewest switches surface hopping algorithm.^232–234^ The total electronic WF

115 $$|\Psi \left(R\left(t\right)\right)\rangle =\sum_{\left(n\right)}d^{\left(n\right)}\left(t\right)|\Psi^{\left(n\right)}\left(R\left(t\right)\right)\rangle$$

is thereby relying on the TDDFT module of CP2K, thus the singly excited states |Ψ^(*n*)^(**R**(*t*))⟩ are defined based on the excitation amplitudes *X*_*ia*_^(*n*)^ of the eigenvalue problem of eq 78, i.e.

116 $$|\Psi^{\left(n\right)}\rangle =\sum_{ia}X_{ia}^{\left(n\right)}|\Phi_{ia}\rangle$$

with the singly excited Slater determinants |Φ_*ia*_⟩. The Lagrangian of eq 78 furthermore defines the excited state nuclear gradients, as outlined in Section 6. The therein defined force **F**, given as the negative derivative of the variational Lagrangian with respect to the nuclear coordinates **R**

117 $$F_{\alpha }\left(t\right)=-\nabla_{\alpha }L\left(R\left(t\right)\right)$$

determines the acceleration **a** of nuclei α as

118 $$a_{\alpha }\left(t\right)=\frac{F_{\alpha }\left(t\right)}{M_{\alpha }}$$

with *M*_α_ indicating the corresponding nuclear mass. Corresponding CP2K inputs thus require choosing RUN_TYPE ENERGY_FORCE with a suitable &TDDFPT section and to print excited state nuclear gradients and amplitudes by adding &PRINT subsections to the &FORCE_EVAL and &TDDFPT sections, respectively:
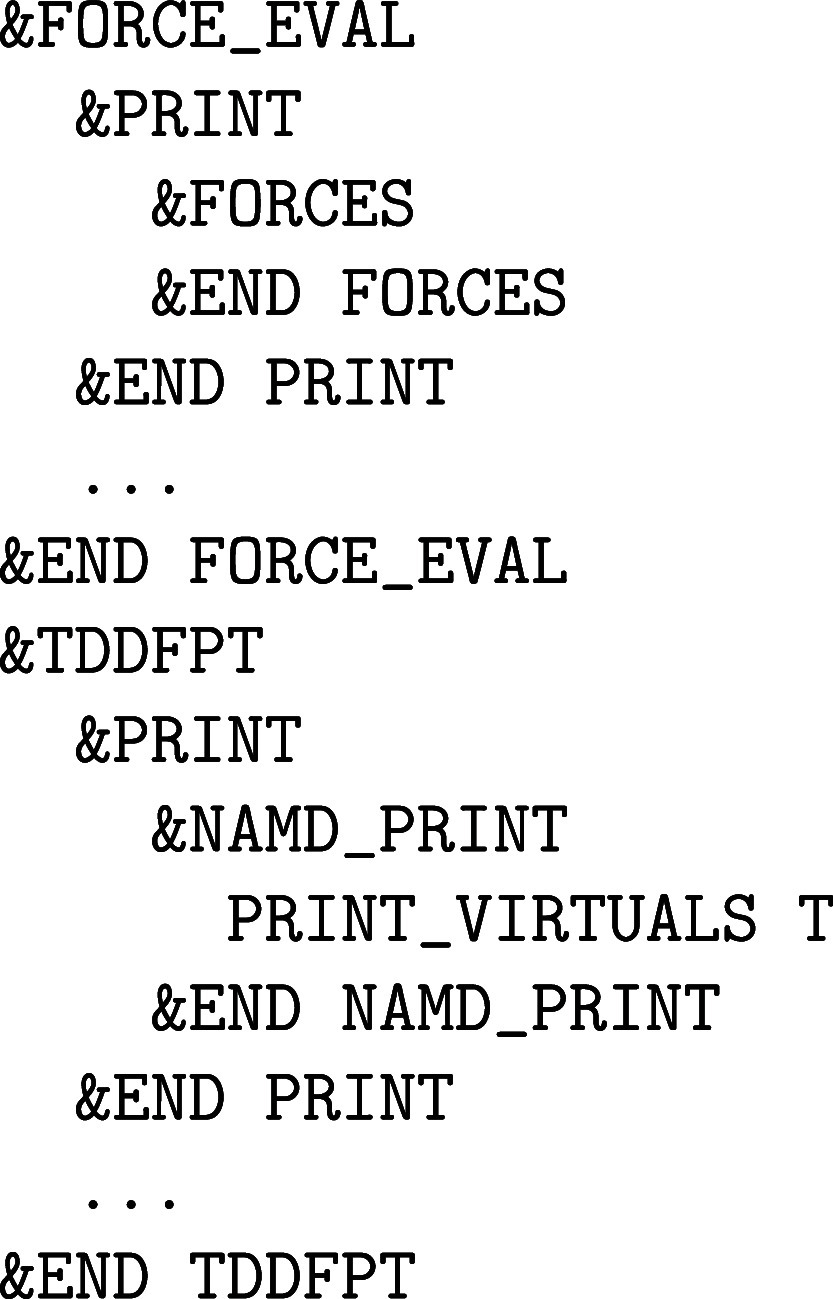


The keyword PRINT_VIRTUALS implies that the excited state amplitudes are printed within the MO basis, no longer referring to Sternheimer atomic-orbital based quantities. Real-time propagation of the coefficients *d* ^(*n*)^(*t*) of the total electronic WF, as defined in eq 115, requires furthermore computing nonadiabatic coupling elements σ_(*mn*)_(*t*) via

119 $$i\frac{dd^{\left(m\right)}\left(t\right)}{dt}=\sum_{\left(n\right)}d^{\left(n\right)}\left(t\right)\left[\delta_{\left(mn\right)}E_{\left(n\right)}\left(t\right)-i\sigma_{\left(mn\right)}\left(t\right)\right]$$

with *E*_(*n*)_(*t*) representing the total energy of excited state *n*, i.e. *E*_(*n*)_(*t*) = *E*_GS_(*t*) + Ω^(*n*)^(*t*). Input options for efficient time-derivative couplings are set within corresponding NEWTON-X input files, requiring no further specifications within the CP2K input file. To generate initial geometries and velocities based on a Wigner probability distribution, a print statement has to be added to the &VIBRATIONAL_ANALYSIS section to enable printing of Cartesian normal modes:
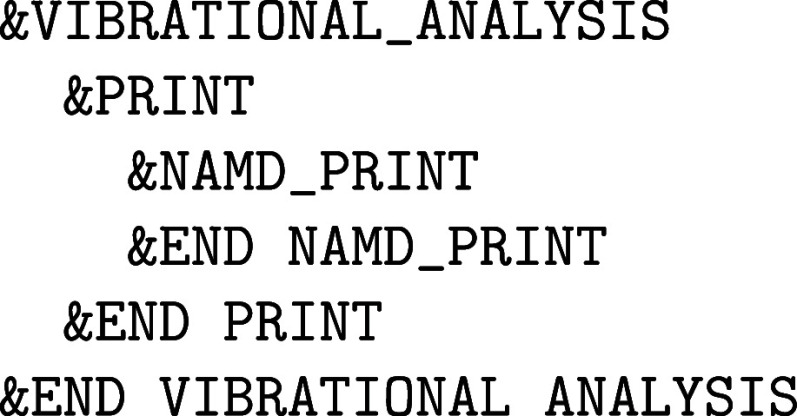


## 8 X-ray Spectroscopy

The prediction of properties that depend on the core electronic density requires the explicit calculation of all-electron WFs and the use of large basis sets, close to the basis set limits. This is the case for inner-shell spectroscopy, where excitation processes of core orbitals involve the rearrangement of the all-electron charge distribution. In CP2K/Quickstep, all-electron calculations require the GAPW approach, as described in Section 2.

The X-ray absorption process is related to the incoming photon being absorbed by core-level electrons, which get resonantly promoted to an excited state. The process has a certain probability depending on the properties of the initial and final states. Within the dipolar approximation, certain selection rules apply, such as total spin conservation and total angular momentum change by ±1. Due to the very local atomic character of the process, for excitation of a 1s level, only unoccupied states with a local p-character are probed. The spectroscopic process is ultrafast (attosecond) so that the nuclear movement during the excitation can be neglected.

### 8.1 Transition Potential Method

A KS orbital is by definition the WF of a fictitious noninteracting system introduced to reproduce the electron density of the real interacting system. Hence, KS orbitals are considered formal auxiliary quantities without specific physical meaning. Nevertheless, the KS eigenvalues are commonly used to describe physical quantities such as ionization energies and excitation energies, and the orbitals are used to obtain the transition moment. To compute excitation energies in less approximate ways, the ΔSCF KS method has been devised, comparing two systems with one or more excited electrons, taking into account full relaxation.

The incoming radiation is represented by the electromagnetic field

120 $$A\left(r,t\right)A_{0}e\;\cos\left(k\cdot r-\omega t\right)$$

The resulting transition probability between the initial and final state is

121 $$P_{if}=\frac{\pi e^{2}}{2\hbar m^{2}c^{2}}A_{0}^{2}{\left|\left\langle f\left|e^{ik\cdot r}e\cdot p\right|i\right\rangle \right|}^{2}$$

which within the dipole approximation simplifies to *P*_*if*_ ≈ |⟨*f*|**e**·**p**|*i*⟩|, when using the velocity form. This form is equivalent to the length form *P*_*if*_ ≈ (*E*_*f*_ – *E*_*i*_)|⟨*f*|**μ**|*i*⟩|, where **μ** is the dipole operator, when the WFs are exact eigenstates. The single-electron excitation approximation makes it possible to rewrite the initial state as a core WF and the final state as an unoccupied/free electron WF, thereby assuming that all other electrons do not participate. The corresponding matrix element can be rewritten as a single-electron matrix element. The transition potential (TP) method adopts this independent particle approach, but the orbital energies and WFs are taken as a solution of the KS equation with a modified core potential on the absorbing atom. The modified potential employed in standard applications is the TP introduced by Slater and is based on the creation of half core-hole (HCH). Other types of core-hole potentials can also be employed with satisfactory results, such as the full core-hole (FCH). The actual final location of the promoted electron is not taken into account for the determination of the spectra, assuming that it is immediately delocalized in the conduction band and its contribution is nearly equivalent, irrespective of the specific final state. In this manner, only a single electronic structure calculation is needed to determine the entire spectrum.

The procedure consists of running an initial ground state calculation, identifying the core state that has to be excited, changing the occupation number of the corresponding KS orbital, and restarting the SCF optimization while keeping the occupation of the excited core state at the modified value. The excitation energies and transition moments are finally evaluated from the KS energies and MOs obtained with the modified occupation

122 $$\hbar \omega_{if}=\varepsilon_{f}^{TP}-\varepsilon_{i}^{TP},P_{if}={\left|\left\langle \psi_{f}^{TP}\left|\nabla \right|\psi_{i}^{TP}\right\rangle \right|}^{2}$$

It is good practice, after the first ground state optimization, to localize the core orbitals to facilitate the identification of the target core state. The localization is activated via the &DFT%XAS%LOCALIZE section, and is carried out for STATE_SEARCH states, starting from the lowest:
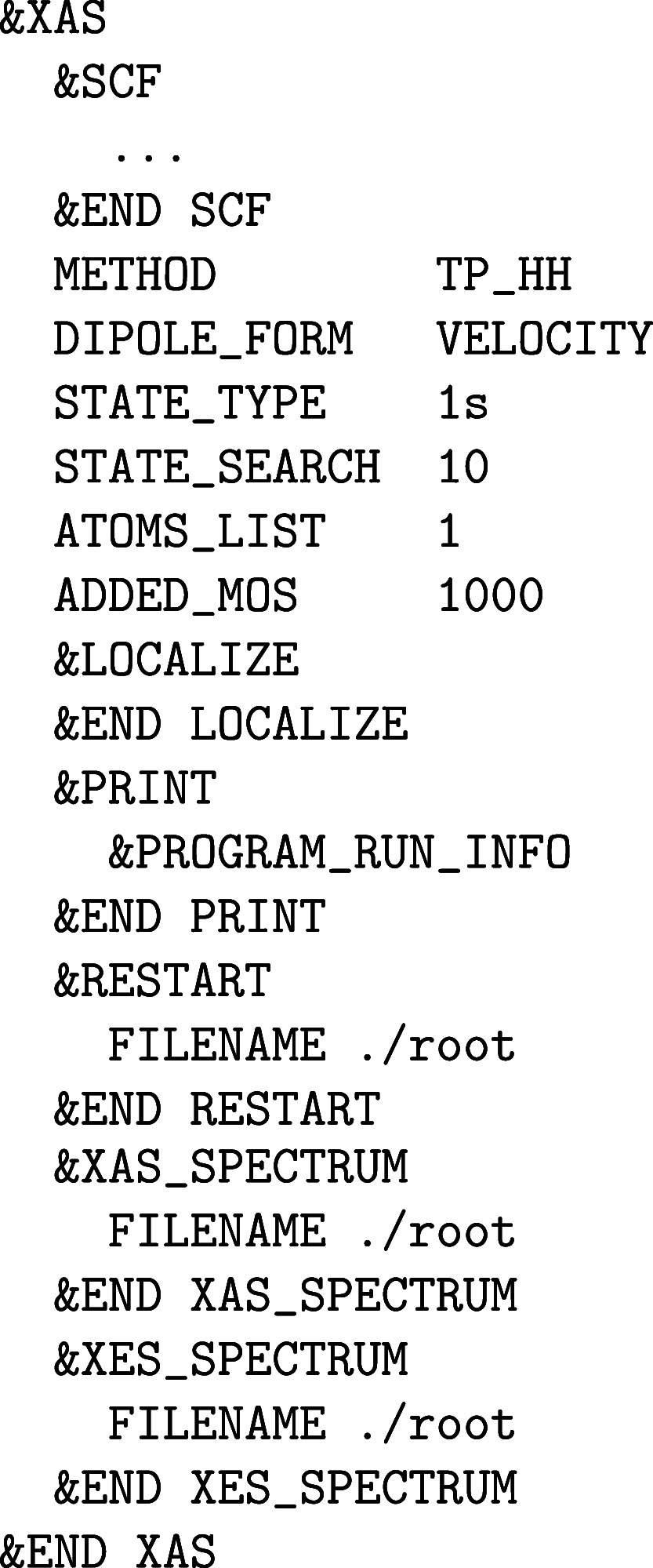


By default, the TP calculation uses the same settings as specified for the ground state. Any different settings given in the &XAS%SCF subsection override the original ones. Anyway, TP calculations run only with the diagonalization method because KS orbitals and their occupations are needed. The emptied core state is defined by the atom, as specified in ATOMS_LIST, and the orbital character STATE_TYPE. An independent TP calculation is performed for each atom of the list. The METHOD keyword defines how the occupation of the core orbital is modified, whereas ADDED_MOS are the additional virtual orbitals that will be computed to obtain a spectrum over a wider energy range. The TP calculation simultaneously provides XAS, core-to-empty transitions, XES, and valence-to-core transitions. Since each TP calculation entails a full SCF optimization, the usual electronic structure output, such as the projected density of states and the cube files of the MOs, are available. These quantities are generally very useful for the analysis of the transition character and rationalize the electronic properties.

### 8.2 Linear-Response Time-Dependent Density Functional Theory

Within LR-TDDFT theory, optical absorption energies are calculated as solutions of the generalized eigenvalue problem presented in Section 6.1. A full diagonalization yields the complete spectrum, with electronic transitions from all occupied to all unoccupied states, including X-ray absorption near-edge structure (XANES), also known as near-edge X-ray absorption fine structure (NEXAFS), from core states to low-lying unoccupied states. However, this approach is not suitable for large systems, as its computational cost scales as $O\left(N^{6}\right)$ with system size (diagonalization of a *N*_occ_ *N*_empty_ matrix).

For efficiency, the CP2K/Quickstep LR-TDDFT implementation for XAS relies on the following three approximations.^197^ The first is the core–valence separation (CVS) approximation.^235^ Due to large differences in energy and localization, the core and valence states are only weakly coupled. This reduces the dimensions of the eigenvalue problem from *N*_occ_ *N*_empty_ to *N*_core_ *N*_empty_, where only occupied core states are considered. In the so-called sudden approximation,^236^ excitations from various core states are further decoupled. Smaller eigenvalue problems of size *N*_empty_ for s-type core states or 3*N*_empty_ for p-type states can be solved independently, leading to further computational savings. However, this approximation relies on the assumption that the core states are highly localized in space.

The localization of core states is further exploited for the efficient calculation of 4-center 2-electron ERIs, using a core-specific RI scheme. Coulomb integrals involving the core states *I*, *J* are computed as

123 $$\left(pI|Jq\right)\approx \left(pI|\mu \right){\left(\mu |\nu \right)}^{-1}\left(\nu |Jq\right)$$

where μ and ν correspond to RI basis functions centered on the same atom where *I* and *J* are localized. The same holds for exact-exchange ERIs in hybrid DFT calculations, i.e. (*pq*|*IJ*) ≈ (*pq*|μ)­(μ|ν)^−1^(ν|*IJ*). Finally, ERIs involving the XC kernel are computed as follows

124 $$\left(pI\left|f_{xc}\right|Jq\right)\approx \left(pI|\kappa \right){\left(\kappa |\lambda \right)}^{-1}\left(\lambda \left|f_{xc}\right|\mu \right){\left(\mu |\nu \right)}^{-1}\left(\nu |Jq\right)$$

Yet, since all RI basis elements are centered on the excited atom, *f*_*xc*_[*n*] can be evaluated by a projection of the total electronic density onto the RI basis, expressed as a linear combination

125 ρ(r)=∑pqPpqφp(r)φq(r)≈∑pq∑μνPpq(pqν)Sμν−1χν(r)=∑νdνχν(r),

where *P*_*pq*_ is the density matrix in the AO basis, while (*pq*ν) represents the overlap integrals between two general AOs and a RI basis element, and *S*_μν_^–1^ is the inverse overlap matrix element of the RI functions centered on the excited atom.

Using LR-TDDFT to compute XAS spectra is based on perturbation theory on top of a ground state. Therefore, the quality of the underlying converged SCF calculation is critical. Since the core electrons of the excited atoms need to be explicitly described, the GAPW method is necessary. Excited atoms must be described with all-electron basis sets, while PPs may be used for all other atoms. A RI basis can be provided for any atomic kind in the system using the RI_XAS keyword, in the &KIND subsection (e.g., BASIS_SET RI_XAS def2-TZVP-RIFIT). If not provided, a default RI basis is generated on-the-fly using the robust method of Stoychev.^81^ For hybrid DFT simulations, the ADMM approximation can be used for the SCF calculation, specifically in its ADMM2 flavor. The RUN_TYPE keyword in the &GLOBAL input section must be set to ENERGY. In addition, LR-TDDFT-based XAS calculations can be performed for both molecular and periodic systems.^237^

The input subsection governing XAS LR-TDDFT calculations is &XAS_TDP, which is within the &DFT section. Some keywords are related to general LR-TDDFT: TAMM_DANCOFF, which controls the TDA (enabled by default), DIPOLE_FORM to calculate the electronic dipole in either the length or velocity-gauge, and EXCITATIONS to enable the calculation of singlet/triplet excitations in closed-shell systems or spin-conserving/spin-flip excitations in open-shell systems. The remaining input is specific to XAS calculations.

Because all approximations used in the LR-TDDFT method of the &XAS_TDP section rely on the localization of core states, it is crucial to select them properly and check their properties. This is done via the &XAS_TDP%DONOR_STATE subsection, which sets the rules for searching for the most suitable core states among the low-energy MOs. First, excited atoms are defined either BY_INDEX or BY_KIND with the DEFINE_EXCITED keyword. In the former case, an INDEX_LIST must be provided with the indices of the excited atoms. In the latter case, a KIND_LIST is provided, specifying which atomic kind is to be excited. The STATE_TYPES keyword then defines which core-level to excite from (1s, 2s or 2p), for each atom index/kind. Finally, the N_SEARCH keyword specifies the *N* lowest-energy MOs to screen for the donor core states previously defined. Note that in some cases, excited atoms might be equivalent under symmetry (e.g., carbon atoms in an acetylene molecule C_2_H_2_). In such a case, the two lowest-energy canonical MOs will be linear combinations of s-type states centered on each atom and effectively delocalized. To ensure that the underlying approximations of the LR-TDDFT XAS method are valid, the *N* candidate donor states can be localized using the LOCALIZE keyword. Localization can be expensive for a large number of MOs: therefore, it is recommended to keep the value of N_SEARCH to a minimum. An example &DONOR_STATE subsection is given below for the CO_2_ molecule:
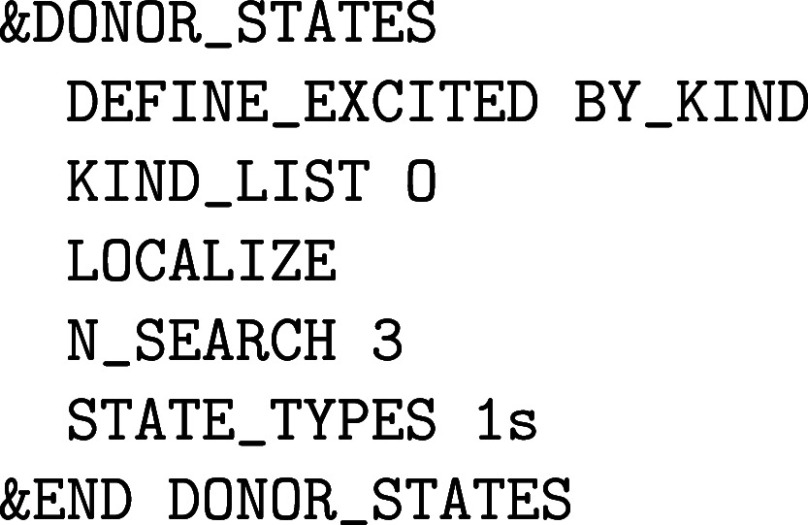


In the above example, the three lowest-energy MOs of the system are localized and used as candidates for O 1s donor states. The 2 candidates that best overlap with a minimal STO basis representation of an O 1s core state are selected. The overlap, as well as the Mulliken charge analysis, is reported in the calculation output. These figures should be checked to ensure the suitability of donor states.

Another important input subsection of the LR-TDDFT method is &XAS_TDP%KERNEL, which defines the XC kernel. Both the XC functional and exact exchange for hybrid DFT calculations are specified here. This subsection must always be explicitly included, even if the kernel is the same as the ground state functional. All recommendations for periodic calculations with HF apply equally for the &EXACT_EXCHANGE subsection. Note that only global hybrids are currently supported. Below is an example &KERNEL sections for a periodic PBE0 hybrid calculation, with the truncated Coulomb potential and a cutoff radius of 6 Å:
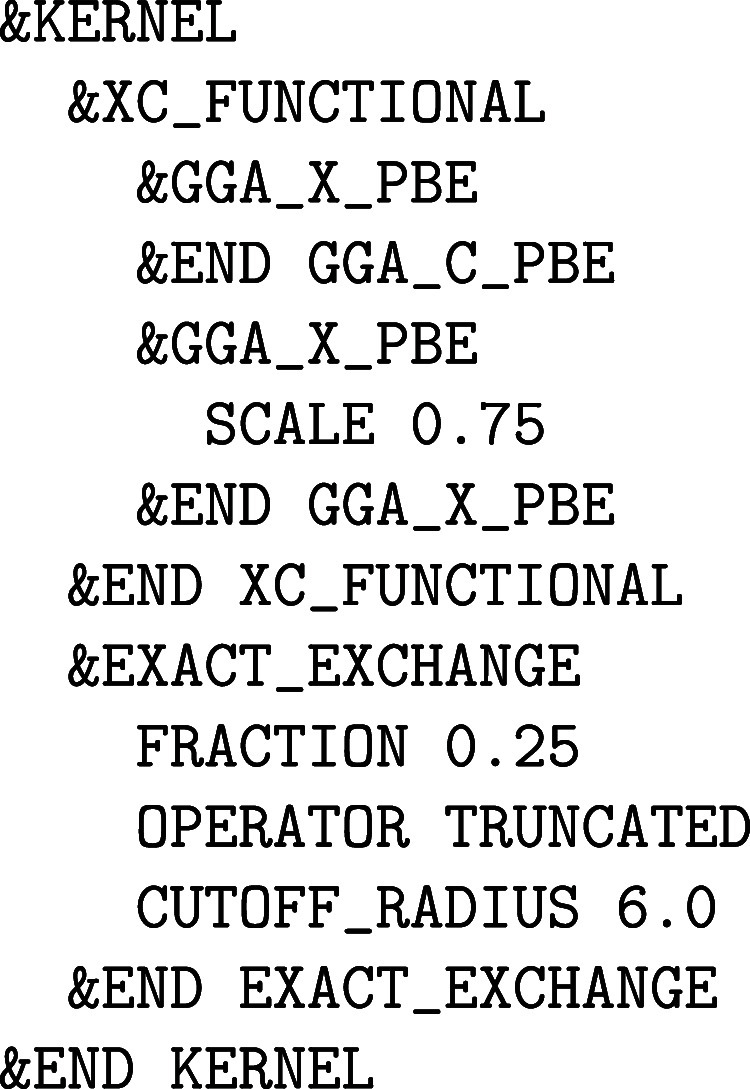


Also related to the XC kernel is the &XAS_TDP%GRID keyword. As shown in eq 125, *f*_xc_ is evaluated on a projection of the density on the RI basis elements centered on the excited atom. The integral is then performed numerically on an atomic grid. By default, the grid dimensions are the same as the GAPW settings for the SCF calculation. It is, however, recommended to use a finer grid. For example,
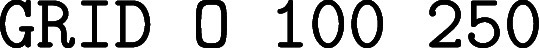
would define grids with 100 angular points (Lebedev scheme) and 250 radial grid points for excited oxygen atoms. Note that the GAPW default is a 50 × 50 grid.

Finally, SOC calculations for L-edge spectroscopy can be enabled by adding the SPIN_ORBIT_COUPLING keyword. The SOC is added perturbatively on top of an LR-TDDFT calculation using the auxiliary many-electron WF (AMEW) framework.^238^ For spin-restricted systems, both single- and triplet-excitations must be calculated. In the open-shell case, spin-conserving and spin-flip excitations must be calculated. For example,
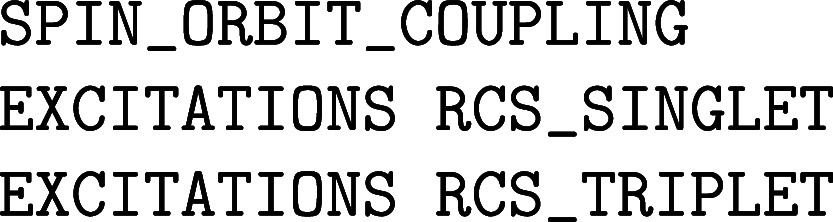
would turn on SOC for LR-TDDFT-based XAS calculations on top of a restricted closed-shell ground state.

It is generally recommended to run LR-TDDFT XAS calculations with hybrid functionals containing a large fraction of exact exchange to reduce the self-interaction error (e.g., PBEh (α = 0.45) or BHHLYP). For such simulations, the computational bottleneck is often still the SCF cycle. Yet, large performance gains can be achieved using the ADMM approximation. Diagonalization costs can also become high for large systems with multiple excited atoms (e.g., all oxygen atoms in a liquid-water simulation). Hence, the iterative &OT_SOLVER can be used to reduce the time spent diagonalizing multiple matrices. Note that, due to its relatively high initialization cost, the OT solver should not be used for a single excited state. Generally, the automatically generated RI basis is very robust and accurate. However, its size is typically larger than some existing preoptimized RI bases (e.g., def2-TZVP-RIFIT). If such a basis exists, using it might save computing time as well.

If unphysical excitation energies are obtained, there are a few possible culprits. The most likely reason is that the donor core states are not properly prepared. They may not be localized or fall outside the range specified by &DONOR_STATES%N_SEARCH. One should check the output file for the overlap and the Mulliken charge analysis (both should be close to 1.0, or even higher for the overlap of 2p states). Another possibility is that the density projection in eq 125 is inaccurate. This sometimes happens when a heavier atom described with an all-electron basis is in the vicinity of the excited atom: because the RI basis functions are centered on the latter, they might fail at describing the core states of the former. The easiest solution is to describe the heavier atom with a PP. If this is not possible, e.g. because of interest in excitations from both atoms, one can use the RI_REGION keyword of the &KERNEL subsection. This allows for the projection of the density on a larger RI basis, with contributions from all atoms within a sphere around the excited atom. In such a case, the use of a fine radial grid is recommended, in order to describe the sharp features of the neighboring atom. Finally, the issue could be numerical, and the GRID, or various general parameters could need tightening, or the usage of a better basis set.

The LR-TDDFT XAS method and its implementation are extensively discussed in ref 197. Various accuracy and performance benchmarks were performed, and all input files are openly available in ref 239.

### 8.3 Real-Time Time-Dependent Density Functional Theory

Spectroscopic properties can also be obtained by means of RT-TDDFT. The absorption spectrum ranging from ultraviolet–visible (UV–vis) to X-rays can be computed by studying the time dependence of the induced electric dipole moment, or the induced current density in condensed matter systems, after subjecting the system to an external electric field in the form of a very narrow function in time (delta kick). Such a step-function-like external potential will excite all the modes in the system. A corresponding sample input section looks like this:
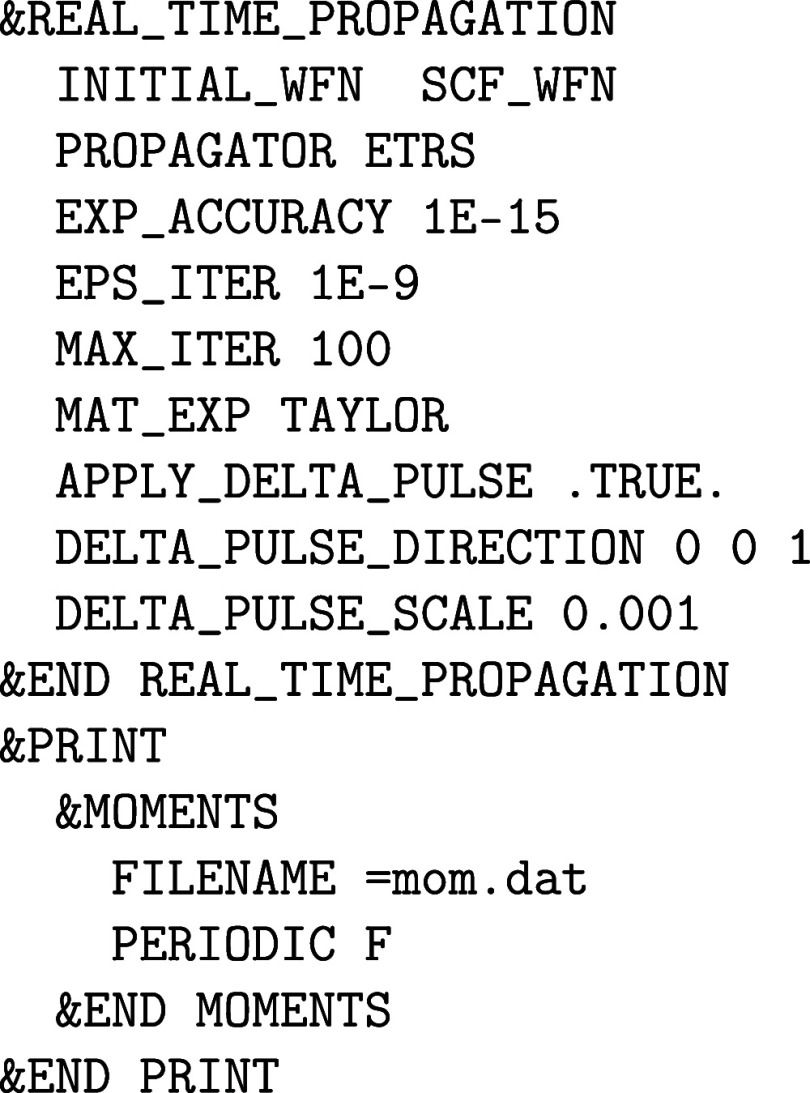


This approach has been extensively studied for years to simulate UV–vis absorption spectra. The first completely general implementation of RT-TDDFT for core states was done in 2012 by K. Lopata et al. within the NWChem code.^240^ The absorption spectrum of a system can be obtained by performing three simulations employing the RTP procedure, each applying the field polarized along one Cartesian axis. For nonperiodic simulations, one applies a field in the form of a Dirac delta function, i.e. **E**_δ_ = κδ­(*t*)**e**, where κ is the strength and **e** the polarization. The time-dependent electric dipole moment is obtained from the propagated electronic charge density

126 μ(t)=∫dr⁡n(r,t)r

Assuming the linear regime, i.e. **μ**(ω) = **α**(ω)·**E**(ω), the molecular polarizability tensor is then obtained by Fourier transforming the time-dependent dipole moment

127 $$\alpha_{ij}\left(\omega \right)=\frac{1}{\kappa }\mu_{ij}\left(\omega \right)$$

where *i* is the vector component and *j* the polarization direction. The absorption spectrum is finally obtained by computing the trace of the polarizability tensor

128 $$S\left(\omega \right)=\frac{4\pi \omega }{3c}Tr\left[I\left[\alpha \left(\omega \right)\right]\right]$$

According to time-dependent perturbative approaches, the polarizability is real for nonresonant frequencies and diverges at resonances. Therefore, the resonant frequencies are characterized by a nonzero imaginary value and a change of sign for the real part. By analyzing both components of the polarizability together, it is thus possible to verify whether the peaks in the spectrum correspond to actual electronic transitions. This behavior of the polarizability can be helpful to understand whether the spectra obtained via RTP can be considered converged with respect to different simulation parameters, such as the time step and total propagation time.

The velocity-gauge RT-TDDFT formalism is required to apply a time-dependent field to periodic systems. As discussed in a previous section, the vector potential appears in the kinetic term and, when PPs are used, the gauge field also transforms the electron–ion interaction. Although the length-gauge and velocity-gauge forms yield the same result for finite systems, the velocity-gauge representation is periodic for spatially uniform external electric fields. Integrating the time-dependent current density **j**(**r**, *t*) in the simulation cell yields the macroscopic current

129 $$J\left(t\right)=-\frac{1}{V}\int dr\;j\left(r,t\right)$$

In the linear-response regime, by applying a very short pulse, the Fourier transform of the time-dependent current yields the frequency-dependent conductivity σ_*ij*_(ω) and the frequency-dependent dielectric function.

130 $$\epsilon \left(\omega \right)=1+\frac{4\pi iTr\left[\sigma \left(\omega \right)\right]}{\omega }$$

The imaginary part of the frequency-dependent dielectric function corresponds to the optical absorption spectrum in a condensed matter system. An example input looks as follows:
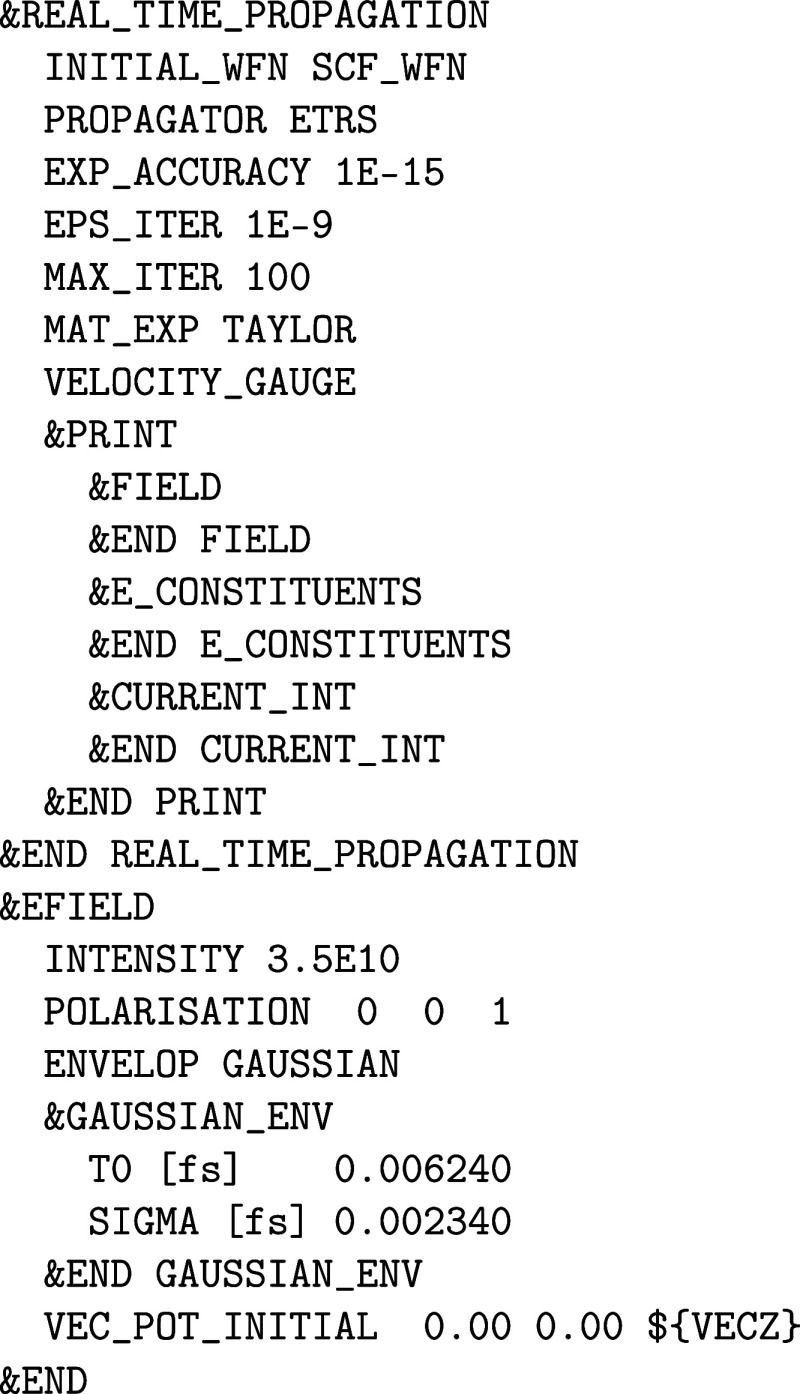


## 9 Energy Decomposition Analysis

Intermolecular bonding arises from a complex interplay of electrostatic interactions between permanent charges and molecular multipole moments, polarization effects, Pauli repulsion, donor–acceptor orbital interactions (also referred to as covalent, charge-transfer, or delocalization interactions), and weak dispersion forces. Energy decomposition analysis (EDA) quantifies the contribution of each of these components to the total binding energy, providing deeper insight into the physical origins of intermolecular bonds.

The CP2K/Quickstep implementation of EDA is based on absolutely localized molecular orbitals (ALMOs), which are molecular orbitals confined entirely to individual molecules or ions within a larger system.^241,242^ ALMO EDA separates the total interaction energy (TOT) into a frozen density (FRZ), polarization (POL) and charge-transfer (CT) terms, i.e.

131 $$\Delta E_{TOT}=\Delta E_{FRZ}+\Delta E_{POL}+\Delta E_{CT}$$

The frozen interactions term is defined as the energy required to bring isolated molecules into the system without any relaxation of their MOs, apart from modifications associated with satisfying the Pauli exclusion principle, hence

132 $$\Delta E_{FRZ}\equiv E\left(R_{FRZ}\right)-\sum_{x}E\left(R_{x}\right)$$

where *E*(*R*_*x*_) is the energy of the isolated molecule *x* and *R*_FRZ_ is the density matrix of the system constructed from the unrelaxed MOs of the isolated molecules. ALMO EDA is closely related to the block-localized WF EDA,^243^ because both approaches use the same variational definition of the polarization term as the energy lowering due to the relaxation of each molecule’s ALMOs in the field of all other molecules in the system

133 $$\Delta E_{POL}\equiv E\left(R_{ALMO}\right)-E\left(R_{FRZ}\right)$$

The strict locality of ALMOs is utilized to ensure that the relaxation is constrained to include only intramolecular variations. This approach gives an upper limit to the true polarization energy,^244^ and its mathematical and algorithmic details have been described by several authors.^245,246^ The remaining portion of the total interaction energy, the CT term, is calculated as the difference in the energy of the relaxed ALMO state and the state of fully delocalized and optimized orbitals (*R*_SCF_), i.e.

134 $$\Delta E_{CT}\equiv E\left(R_{SCF}\right)-E\left(R_{ALMO}\right)$$

Therefore, Δ*E*_CT_ includes the energy lowering due to electron transfer from the occupied ALMOs on one molecule to the virtual orbitals of another molecule Δ*E*_CT_^(pair)^, as well as the further energy change caused by many-body higher-order induction Δ*E*_HO_ that accompanies such an occupied-virtual orbital mixing and is typically small for intermolecular interactions, thus

135 $$\Delta E_{CT}=\Delta E_{CT}^{\left(pair\right)}+\Delta E_{HO}$$

A distinctive feature of ALMO EDA is that both the amount of the electron density transferred between a pair of molecules and the corresponding energy lowering can be computed via^247^

136a $$\Delta Q_{CT}^{\left(pair\right)}=\sum_{x,y>x}\left\{\Delta Q_{x\to y}+\Delta Q_{y\to x}\right\}$$

136b $$\Delta E_{CT}^{\left(pair\right)}=\sum_{x,y>x}\left\{\Delta E_{x\to y}+\Delta E_{y\to x}\right\}$$

### 9.1 Implementation

Within CP2K, ALMO EDA is restricted to closed-shell fragments. The linear-scaling optimization of ALMOs serves as its underlying computational engine,^248^ which can be applied to both gas-phase and condensed phase systems. It has also been extended to fractionally occupied ALMOs enabling investigation of interactions between metal surfaces and molecular adsorbates.^249^ Another unique feature of our implementation is the ability to control the spatial range of CT between molecules using the cutoff radius *R*_c_ (see below). Additionally, ALMO EDA in combination with CP2K’s efficient AIMD engine allows us to attenuate or switch off CT interactions in AIMD simulations, thus measuring their contribution to the dynamical properties of molecular systems.^250,251^ A single-point ALMO EDA calculation can be set up by creating a regular input file for DFT calculation and then modifying it in several steps.

#### 9.1.1 Step 1. Define Fragments

First of all, all atoms in the system must be assigned to nonoverlapping subsets called fragments. In other words, the system must be partitioned into fragments. A fragment can include a single atom, several nonbonded atoms, a single molecule (that is, several bonded atoms), or several molecules. This partitioning tells the code about localization regions of ALMOs when calculating the Δ*E*_FRZ_ and Δ*E*_POL_ energy terms.

To define fragments, the ALMO code uses the CP2K subroutines that analyze bonding between atoms using distance thresholds. By default, all atoms that are considered bonded to each other are assigned to the same fragment. This approach enables a very flexible definition of fragments through a combination of (a) the &GENERATE subsection of the &&FORCE_EVAL%SUBSYS%TOPOLOGY section, (b) the fifth column in the &SUBSYS%COORD section, and (c) an optional connectivity file. The information about each fragment is printed at the beginning of output files under the “ALMO SETTINGS” marker.

The &GENERATE subsection automatically determines bonding between atoms by comparing interatomic distances to the sum of the atomic radii. This is the most convenient strategy to define bonding between atoms and thus to create fragments. The default behavior of &GENERATE can be changed by modifying its keywords. For example, regular molecules can be partitioned into atomic fragments using low values of the BONDPARM_FACTOR. Low values prevent atoms from being combined into molecular fragments, as shown in the following input:
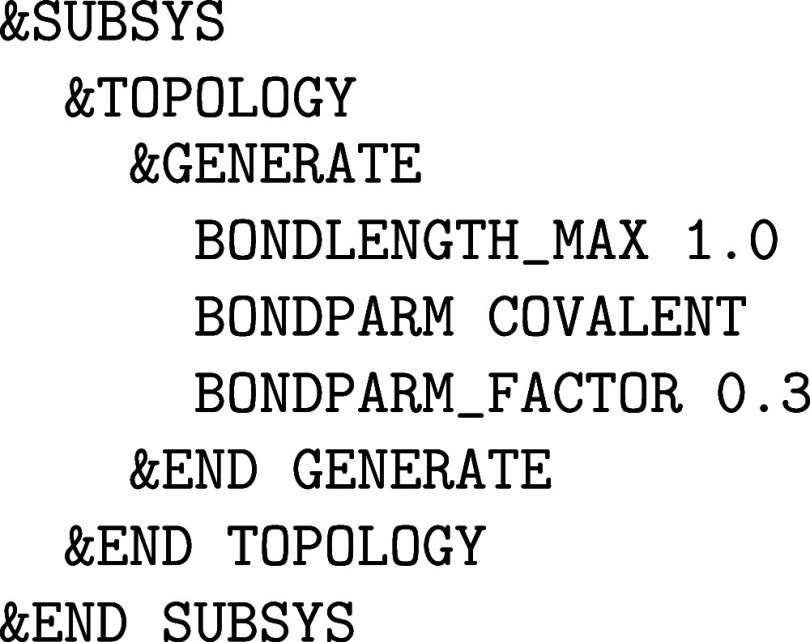


Partitioning of the system can be refined using the fifth column in the &COORD section that assigns fragment labels to each atom. The main principle of this partitioning scheme is that atoms with different fragment labels cannot be combined into a fragment. On the other hand, atoms with the same label may still be separated into fragments as guided by the &GENERATE subsection. Finally, the bonding between atoms can be explicitly specified in a connectivity file. In this case, all bonded atoms are assigned to the same fragment.

#### 9.1.2 Step 2. Assign Electrons to Fragments

All electrons of a neutral atom are assumed to belong to the atom’s fragment. This default behavior can be changed by adding (removing) electrons to (from) atoms using the &BS subsection in the &SUBSYS%KIND section. In the following example, one electron is removed from the 1s orbitals of hydrogen atoms to create H^+^ fragments. Meanwhile, two electrons are added to the 2p orbitals of oxygen atoms to create O^2–^ fragments. Since both H^+^ and O^2–^ are intended to be closed-shell fragments, the same modification has to be performed for the &ALPHA and &BETA subsections of the &BS section. Note that ALMO EDA can even handle zero-electron fragments, such as H^+^.
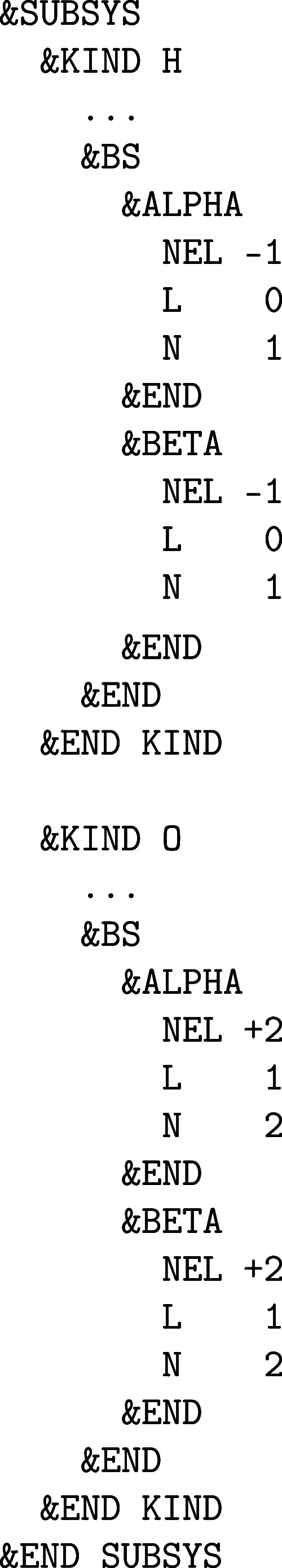


After atoms and electrons are assigned to fragments, the Gaussian basis set functions, all of which are centered on the atoms in CP2K/Quickstep, are also assigned to the same fragments as their atoms. This last partitioning defines the structure of the block-diagonal matrix of ALMO coefficients.

#### 9.1.3 Step 3. Turn ALMO SCF On

To run ALMO EDA calculations, set the ALMO_SCF keyword in the &DFT%QS section to TRUE. This tells CP2K to optimize ALMO coefficients in the ALMO SCF loop instead of optimizing MO coefficients in the conventional SCF loop. That is, the &DFT%SCF section of the input does not control ALMO EDA calculations. Instead, the ALMO SCF loop is controlled by the &DFT%ALMO_SCF section.

#### 9.1.4 Step 4. Compute Δ*E*_FRZ_

To request the calculation of the FRZ term, set &ALMO_SCF%ALMO_SCF_GUESS to MOLECULAR. This instructs CP2K to perform a series of conventional SCF calculations for individual fragments in the periodic box of the entire system. These calculations are controlled by the &SCF section of the input file, and, therefore, it is important that all keywords in this section (e.g., EPS_SCF) are set to ensure sufficient accuracy of the energies of the individual fragments. CP2K/Quickstep then combines the MO of individual fragments into the block-diagonal matrix of the ALMO coefficients and evaluates the energy of this state without any optimization of the coefficients. The computed energies are printed as described in Table 1.

**Table 1:** ALMO EDA Energies in CP2K Output Files

state	CP2K print out	definition	EDA terms
(0)	“single-molecule energy”	*∑*_*x*_ *E*(*R*_*x*_) in eq ([Disp-formula eq132])	Δ*E*_FRZ_ = (1) – (0)
(1)	“energy of the initial guess”	*E*(*R*_FRZ_) in eq ([Disp-formula eq132])	Δ*E*_POL_ = (2) – (1)
(2)	“ENERGY OF BLOCK-DIAGONAL ALMOs”	*E*(*R*_ALMO_) in eq ([Disp-formula eq133])	Δ*E*_CT_^(pair)^ = (3) – (2)
(3)	“CORRECTED ENERGY”	eq ([Disp-formula eq135])	Δ*E*_HO_ = (4) – (3)
(4)^a^	“ENERGY|”	*E*(*R*_SCF_) in eq ([Disp-formula eq134])	Δ*E*_CT_ = (4) – (2)

a If FULL_X or XALMO_X methods are used then the “ENERGY|” line contains the energy of state (3).

#### 9.1.5 Step 5. Compute Δ*E*_POL_

Subsequent optimization of the block-diagonal ALMO coefficients can be performed using various algorithms, which are selected using the ALMO_ALGORITHM keyword and controlled by the corresponding subsections of the &ALMO_SCF section. Although DIIS-accelerated diagonalization of the diagonal blocks of the projected KS Hamiltonian matrix typically converges the ALMO SCF in just a few iterations,^245,246^ this method does not guarantee convergence and can fail for strongly interacting fragments. In problematic cases, preconditioned conjugate gradient or trust region algorithms can be employed instead of the DIIS-accelerated diagonalization. The polarization energy does not depend on the algorithm and is printed as described in Table 1.

#### 9.1.6 Step 6. Compute Δ*E*_CT_

The calculation of the CT term is controlled by the DELOCALIZE_METHOD keyword in the &ALMO_SCF section. In order to compute Δ*E*_CT_ defined in eq 134, set this keyword to FULL_SCF. In order to separate CT into pair contributions defined in eqs 136a and 136b, respectively, DELOCALIZE_METHOD should be set to FULL_X_THEN_SCF. If only the pair contributions are of interest and the higher-order term is expected to be negligible, set DELOCALIZE_METHOD to FULL_X.

For very large systems, the computation of the CT terms can be significantly speeded up by evaluating them only between fragment neighbors within the user-defined localization radius. This is done by specifying the localization cutoff distance with &ALMO_SCF%XALMO_R_CUTOFF_FACTOR and by setting DELOCALIZE_METHOD to XALMO_SCF, or to XALMO_X. The latter two settings are equivalent to using FULL_SCF and FULL_X for fully delocalized orbitals. Note that pair CT components can be calculated with XALMO_X, but cannot be obtained with XALMO_SCF, respectively.

The computed CT energies are printed as described in Table 1. The printout of the pair CT terms can be activated by adding the &ANALYSIS subsection inside &ALMO_SCF and specifying output file names inside the &PRINT subsection. Each message passing interface process will print its own part of the pair list into a separate file. These output files have three columns. The first column is the index of the fragment that accepts electrons. The second column is the index of the fragment that donates electrons. The third column is the amount of electron density transferred in units of the elementary charge or the corresponding CT energy in Hartrees.

#### 9.1.7 Input File Example

The relevant input sections for an ALMO EDA calculation involve:
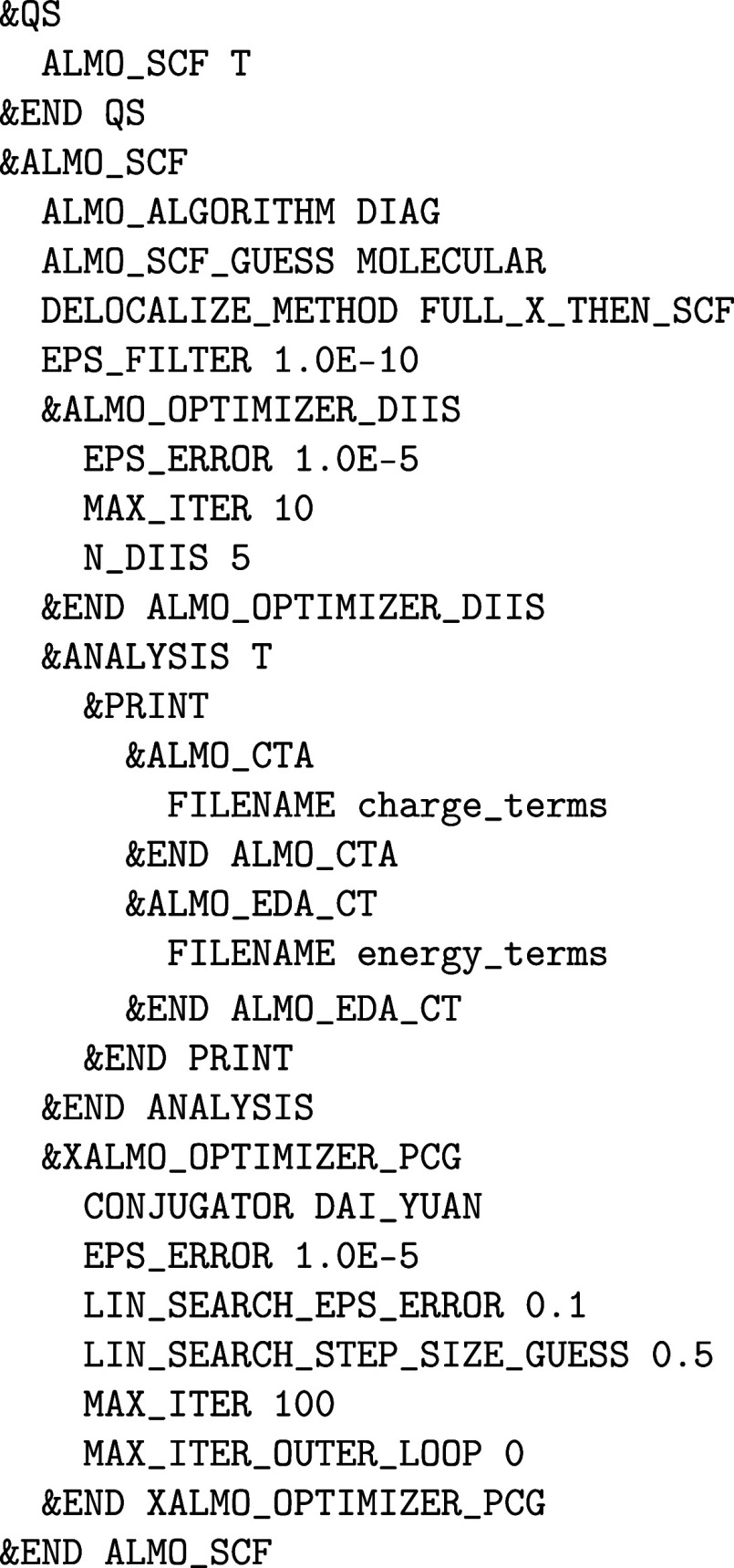


#### 9.1.8 Illustrative Applications

ALMO EDA has been applied to study intermolecular interactions in a variety of gas and condensed phase molecular systems,^252^ as well as their interfaces.^253,254^ The implementation of ALMO EDA in CP2K has played a crucial role in advancing our understanding of hydrogen bonding in liquid water,^242,255–257^ ice^258^ and water confined to low dimensions.^259–261^ These studies have systematically shown that the small amount of electron density transferred between molecules has a profound effect on the structure,^257,262^ spectroscopic response,^256,262–265^ and dynamical properties^250,251,266–268^ of liquid water.

## 10 Finite Temperature Effects

For the purpose of explicitly including finite temperature effects, CP2K implements a large variety of MC and MD algorithms that can be activated via the &MOTION%MC and &MOTION%MD sections, respectively.^5,269–273^ The most distinctive feature of CP2K, however, is the ability to conduct efficient AIMD simulations,^10,13^ and in combination with linear-scaling algorithms, even for rather large-scale systems.^77,274,275^ Yet, in spite of its name, initially CP2K only contained Born–Oppenheimer MD,^276–279^ since the original Car–Parrinello algorithm is not implemented.^280^ Instead, the second-generation Car–Parrinello method is available,^11,12^ but since this has been extensively covered elsewhere,^281–286^ the present focus is solely on MLPs and NQE by means of PIMD.

### 10.1 Neural Network and Machine Learning Interaction Potentials

The rise of MLPs in recent years provides efficient and accurate representations of potential energy surfaces from electronic structure reference data.^287–294^ This has enabled the simulation community to reach previously inaccessible spatiotemporal scales through atomistic simulations of complex, reactive systems at near ab initio accuracy as hitherto only known from explicit (on-the-fly) AIMD^9,13^ simulations. High-dimensional neural network potentials (NNPs)^288,295^ combined with atom-centered symmetry functions to describe atomic environments^296^ have been the first methodology to provide a scalable and accurate representation of potential energy surfaces using artificial neural networks. Introduced in 2007 by Behler and Parrinello, this framework has been used to provide fundamental insight into a plethora of challenging systems, from reactive gas-phase clusters,^297,298^ to phase behavior of liquids like water,^299,300^ chemical reactions at interfaces,^301^ high pressure systems,^302–304^ complex materials,^305^ battery interfaces,^306^ and phase-transitions,^307,308^ to name but a few.^288,293,309^ Although many other methods have been proposed over the years, NNPs remain a mainstay in the toolbox of computational scientists. Their conceptually simple and computationally efficient design and their systematic extensions to treat long-range effects via environment-dependent charges^310^ and self-consistent charge equilibration^311,312^ to target remaining limitations within the same framework makes them a versatile and robust tool to address problems of ever increasing complexity.^293^ For the purpose of this tutorial, we concentrate here on the original formulation, which is deeply implemented in CP2K and very simple to use by newcomers in the field.

To represent a potential energy surface by NNPs, the atomistic structure is first transformed using atom-centered symmetry functions into translationally and rotationally invariant descriptors of atomic environments.^296^ These serve as input for atomic neural networks that output auxiliary components of the total potential energy, which is then obtained as a sum of contributions from all atoms in the system. The resulting permutationally invariant structure-energy relation can be analytically differentiated to obtain the nuclear forces, for example, to drive MD, and is scalable to essentially arbitrary system sizes.^295^ Model training is achieved by optimizing the parameters (weights and biases) of the atomic neural networks to reproduce reference energies and optionally forces. Besides the original RuNNer code of Behler, other training codes like n2p2 and RubNNet4MD are available and yield models that can be directly used in CP2K. For a light introduction into NNPs and other MLPs we refer the reader to ref 313, as well as to previous reviews on this topic.^314,315^

The implementation of NNPs in CP2K is fully compatible with the original RuNNer code and thus n2p2 format and can be used in the same way as other potential energy models. The NNP module in CP2K is part of the &FORCE_EVAL section and can be used in combination with other methods such as DFT, traditional force fields, or QM/MM methods. Inside the &NNP subsection, the keyword NNP_INPUT_FILE_NAME reads the NNP from a file, which can be generated using the RuNNer code or other compatible training codes such as n2p2 or the RubNNet4MD package.^316^ It is noted only in passing that the atomic cluster expansion (ACE) MLP technique^317^ has already been interfaced with CP2K and that its tree graph extension (grACE)^318^ is addressed as this article is written. Similarly, well-developed interfaces to CP2K also exist for DeePMD-kit,^319^ NequIP^320^ and Allegro,^321^ all of which can be activated via their respective specialized subsections in &FORCE_EVAL%MM%FORCEFIELD%NONBONDED. Contrary to the native NNP implementation of CP2K, the corresponding libraries must be linked during compile time, as described in detail in Section 11.1.

The NNP is then used to compute the energy and forces of the system, where the analytical stress tensor is also available for constant-pressure simulations. Parallization is achieved using particle decomposition and scales well up to thousands of cores and number of atoms. An example of the input section for the NNP module is shown here:
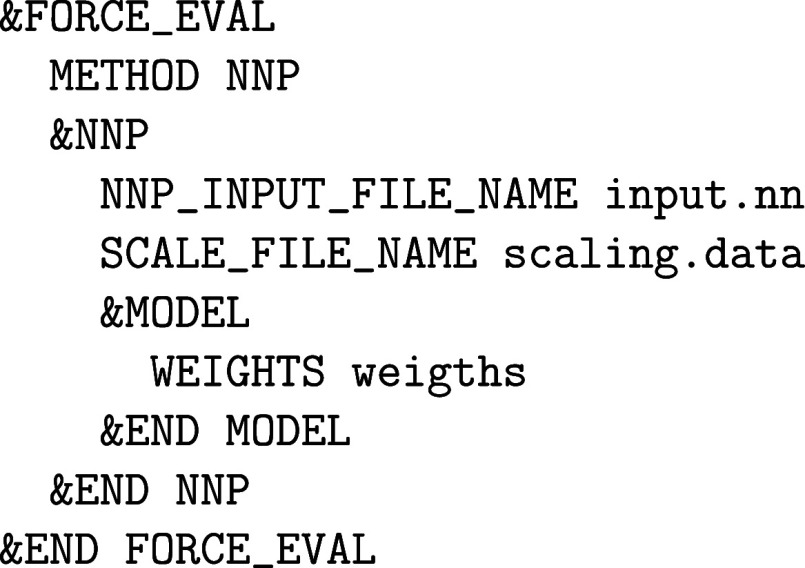


The original NNP formalism can also be extended to so-called ensemble or committee models, where multiple NNPs are combined for boosted accuracy and error estimation. Individual committee members are trained separately from independent random initial conditions to a subset of the total training set.^322^ If the resulting committee shares the same atom-centered symmetry functions as descriptors for the atomic environments,^296^ there is only a small, often negligible, computational overhead in production runs. While the committee average provides more accurate predictions than the individual NNPs, the committee disagreement, defined as the standard deviation between the committee members, grants access to an estimate of the error of the model. This committee disagreement provides an objective measure of the error of the underlying model,^323^ which can be used in active learning protocols to improve the model systematically.^324–326^ This has been utilized extensively in recent times for the automated development of NNPs for various systems,^327^ while current research showed that it leads to more diverse configuration sampling, but not necessarily to more accurate models.^328^ This committee approach is also implemented in CP2K.^322^

Committee NNPs (C-NNPs) can be used in the same way as single NNPs in CP2K, with the addition of the &NNP%MODEL section for each committee member. The committee members are defined by the number of &MODEL subsections in the input file, where each subsection contains the weights of the NNP. It is important to ensure that these models have been trained with the same set of descriptors because they are assumed to be shared between all committee members. An example of the input section for a C-NNP setup with three committee members is shown here:
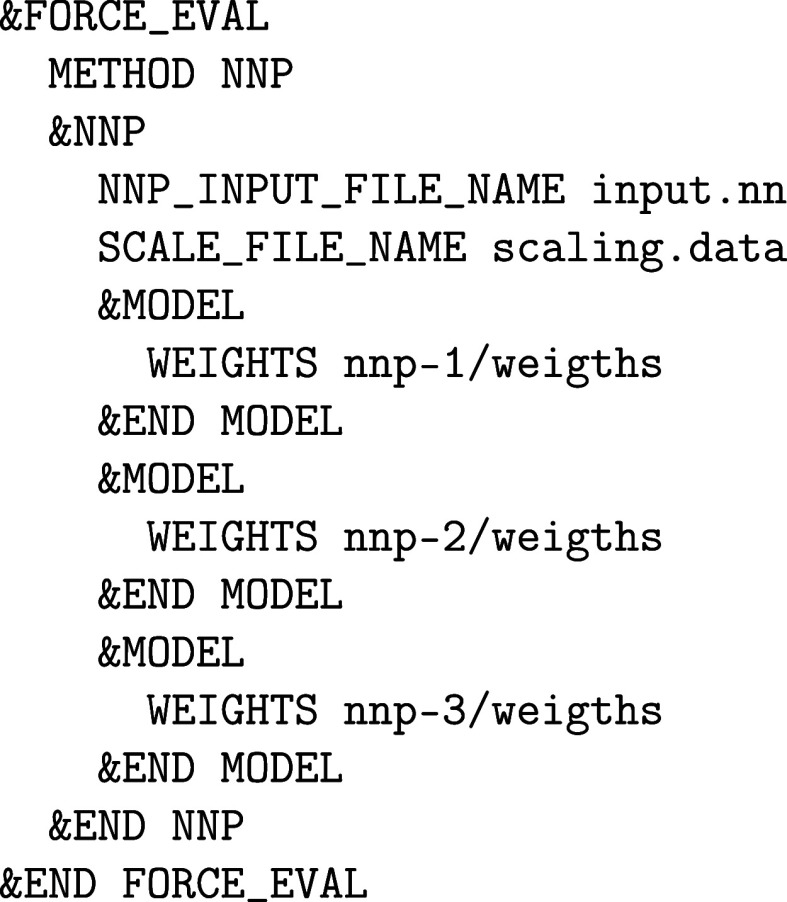


Besides these features, the NNP module in CP2K also provides the possibility to print the energy and force disagreement between committee members, as well as configurations which are in the extrapolation regime of the model. This can be used to identify regions of the potential energy surface where the model is inaccurate in order to improve it systematically. These outputs are controlled using the &PRINT subsection in the &NNP section. An example of the input section for the printing of the committee energy disagreement is shown in the following:
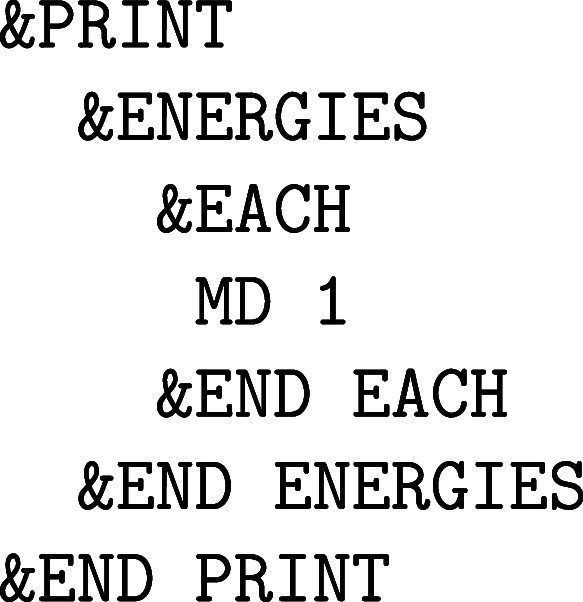


Similarly, the printing of forces, force disagreement, and extrapolation points can also be enabled (FORCES, FORCE_SIGMA, EXTRAPOLATION).

In addition, there is an option to bias the committee disagreement to stabilize simulations by restraining the system dynamics to regions where the committee members agree. This can be achieved by adding a harmonic repulsive wall acting on the committee disagreement after a chosen threshold, which adds a bias to the total potential energy of the system. An example of the input section for the biasing of the committee energy disagreement looks like:
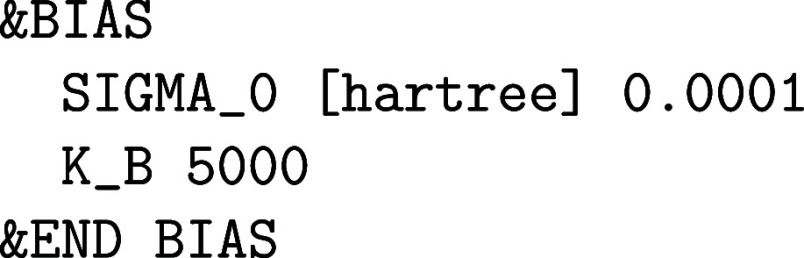


Moreover, &BIAS%ALIGN_NNP_ENERGIES can be used to subtract an energy shift from individual committee members to reduce misalignment effects, while bias energies can be printed with the &BIAS%PRINT section using &BIAS_ENERGY; further details of this feature can be found in ref 322.

Finally, we note that the parameters of a C-NNP for water are available within the CP2K data repository and can be accessed via the NNP/bulkH2O-jcp2020-cnnp/nnp-X/ path in the input file. The model has been trained against revPBE0-D3 hybrid functional reference data and is suitable for water over a wide range of temperatures and pressures. Further details of the development can also be found in ref 322. We use this model to illustrate the easy usability of the NNP implementation in CP2K by showing results for liquid water at 300 K in Figure 2a–c, respectively. From the comparison of the structural and dynamical properties of liquid water, we can see that the C-NNP model provides a faithful representation of the system, with the radial distribution functions and vibrational density of states being in perfect agreement with the reference AIMD data. The computational efficiency of the C-NNP model is also demonstrated by the comparison of the time per MD step for different system sizes, which shows that the C-NNP model (solid lines) is orders of magnitude faster than the reference AIMD simulations (dashed line). This makes the C-NNP model a powerful tool for the study of complex systems at finite temperatures.

**Figure 2:**
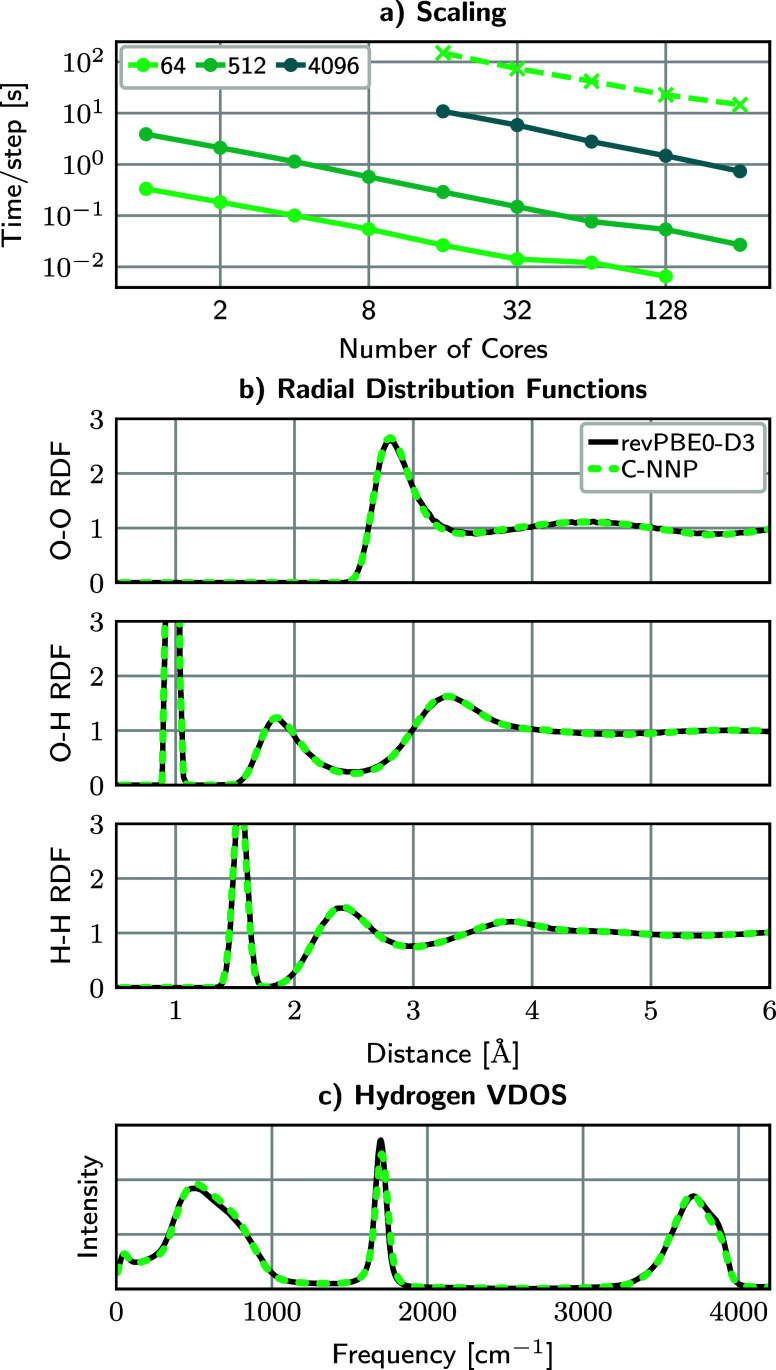
Comparison of NNP and AIMD results for liquid water at 300 K as obtained from CP2K. (a) Time per MD step for 64, 512, and 4096 water molecules with the C-NNP model (solid lines with circles) and 64 water molecules with the revPBE0-D3 hybrid functional (dashed line with crosses) reported on a logarithmic scale. (b) Radial distribution functions and (c) vibrational density of states of the hydrogen atoms. Data in panel (b,c) are partially reused from ref 322. Copyright 2020, American Institute of Physics.

### 10.2 Nuclear Quantum Effects

The quantum nature of nuclei can have a significant impact on the properties of materials, especially at low temperatures.^329–343^ A very elegant way to include these NQEs in molecular simulations is the imaginary time path-integral (PI) formalism, which is based on the Feynman–Kac formulation of quantum statistical mechanics.^7,9,344–346^ In the PI formalism, the quantum mechanical system is mapped onto a classical system of *P* replica, connected by harmonic springs to adjacent replica under cyclic boundary conditions in the simplest case (leading to the so-called primitive Trotter approximant). Replicas (a.k.a. “beads”) of the same index are coupled through a scaled version of the potential energy surface, which is determined by the Trotter number *P*. This approach allows for the calculation of quantum statistical observables by sampling the classical phase space of the *P*-fold system. For more details on the PI method, we refer the reader to refs 7,9,347–350 .

In CP2K, PI simulations are available via the &PINT section, which provides a flexible and modular environment for the inclusion of the quantum nature of the nuclei. In this PIMD framework, the equations of motion resulting from the effective potential are integrated numerically to sample the PI formulation of the canonical (*NVT*) partition function of the system.^7,9,351,352^ This implementation mainly supports the normal mode transformation for the efficient integration of the stiff harmonic ring-polymer modes.^353^ In addition to the reversible reference system propagator algorithm (RESPA) multiple time step integrator (NRESPA with HARM_INT NUMERIC),^354^ an exact integrator for normal mode coordinates is also available via HARM_INT EXACT.^355^ An example input section for a PIMD simulation is shown here for a system of *P* = 32 replica at 250 K and 1000 steps of 0.25 fs:
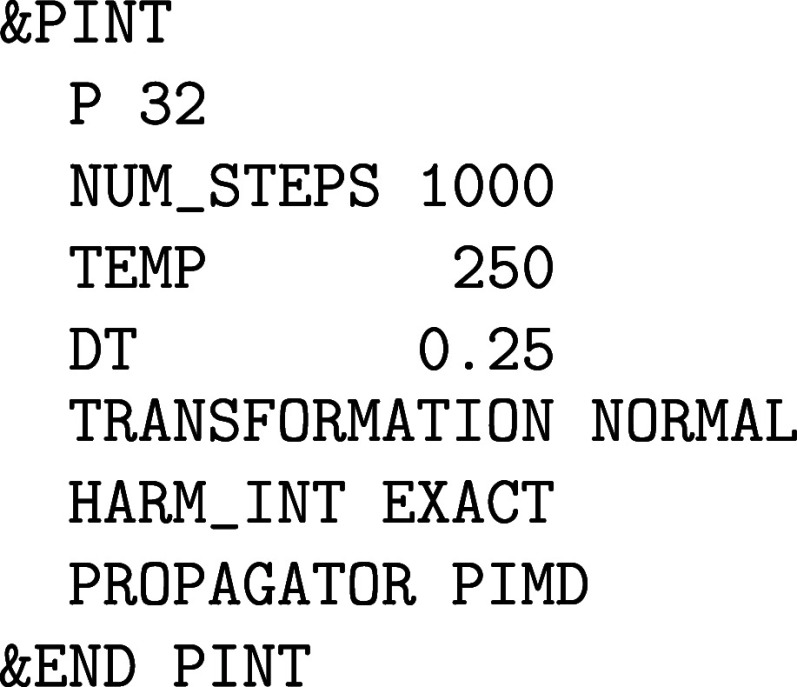


For standard PIMD simulations in the canonical ensemble, the temperature can be controlled using two thermostatting techniques, namely the massive Nosé-Hoover chain (mNHC) thermostat,^356,357^ applied separately to all degrees of freedom mostly in normal mode coordinates, and the PI Langevin equation (PILE) thermostat.^355^ They are enabled via dedicated subsections that change the ensemble from the microcanonical (*NVE*) to the canonical ensemble, if specified. The Nosé–Hoover chain thermostat is controlled by the &NOSE subsection, whereas the PILE thermostat is controlled by the &PILE subsection. While the Nosé-Hoover chain thermostat only requires the definition of the chain length, the PILE thermostat needs the definition of the coupling constant λ and the relaxation time τ as demonstrated here:
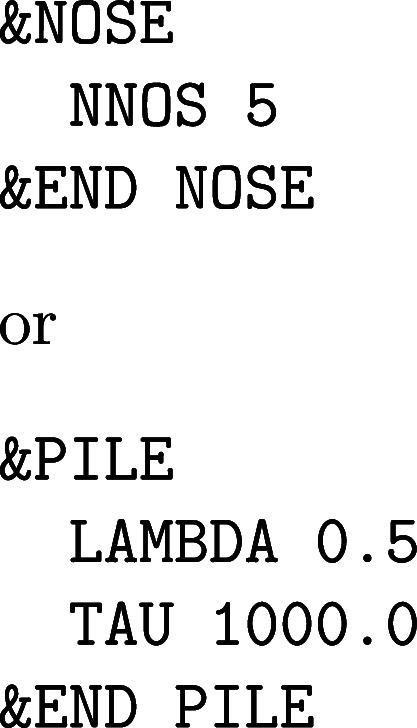


In addition, there are also quantum thermostats available that are specifically designed to improve the convergence with respect to the number of replicas in PI simulations, discussed in more detail in Section 10.3.

Finally, it is also possible to constrain the centroid or all replica degrees of freedom using the CP2K-wide constraint framework defined by the &MOTION%CONSTRAINT section. Within that section, the PIMD_BEADWISE_CONSTRAINT F|T keyword switches between beadwise and centroid constraints. The KT_CORRECTION keyword in the &PINT section can be used to correct for the loss of temperature due to constrained degrees of freedom for Nosé-Hoover chains and numerical integration. Constraints and restraints, which are available through the same framework, can be used to obtain free energy profiles, including NQEs through thermodynamic integration, umbrella sampling and umbrella integration, respectively.^358,359^

### 10.3 Quantum Convergence

The PI formalism is exact in the limit of infinite discretization, i.e. *P* → ∞. However, in practice, some finite *P* value must be selected while the properties of interest converge with increasing *P*. Unfortunately, this convergence is system, temperature and mass dependent, making it computationally demanding to treat systems in the deep quantum regime, where unpleasantly large *P* values are required. For example, the bead convergence of structural properties of hydrogen-bonded systems requires roughly *P* ≈ 32 at 300 K, *P* ≈ 128 at 100 K, and *P* ≈ 8192 at about 1 K, as analyzed in ref 360. Due to these reasons, different techniques have been developed to accelerate the convergence of PI simulations.^361^ In particular, colored noise thermostats^362–364^ in combination with generalized Langevin dynamics and quantum thermal baths have been shown to significantly reduce the required number of beads for convergence down to the ultracold temperature regime.^360,365^ Two such thermostats, the PI generalized Langevin equation thermostat (PIGLET)^363^ and the PI quantum thermal bath (PIQTB) thermostat,^364^ are available in CP2K. The main idea behind both of these approaches is to impose a frequency-dependent temperature *T*(ω), which matches the quantum fluctuations of a harmonic oscillator. Due to zero-point energy leakage, these thermostats require rather harsh coupling constants, rendering them incompatible with approximate quantum dynamics methods like centroid MD (CMD) and ring-polymer MD (RPMD), described in more detail in Section 10.4.

The PIGLET thermostat, based on the generalized Langevin equation, is enabled with the &PINT%PIGLET thermostat subsection. This formalism requires dedicated matrices to impose the correct frequency-dependent thermostatting for each combination of temperature and number of beads. A large variety of matrices can be downloaded from https://gle4md.org/index.html?page=matrix, requiring to use the raw format for data consistency. The matrices file must be specified in the &PIGLET subsection using the MATRICES_FILE_NAME keyword. The PIQTB formalism, using a quantum thermal bath thermostat for each ring-polymer normal mode, is also based on the one-body density matrix as PIGLET but has the advantage of working continuously for any combination of temperature and number of beads. It is enabled using the &QTB thermostat section after having specified the underlying PIMD simulation, we show an example input section for the PIQTB thermostat below. In addition to the coupling constant λ and the relaxation time τ, the angular cutoff frequency for the normal modes needs to be specified by suitable values for TAUCUT and LAMBCUT, respectively. The example values below have been shown to work well for hydrogen-bonded systems^360^ and should also be transferable to other systems. They have been used for the bead convergence study in Figure 3a.
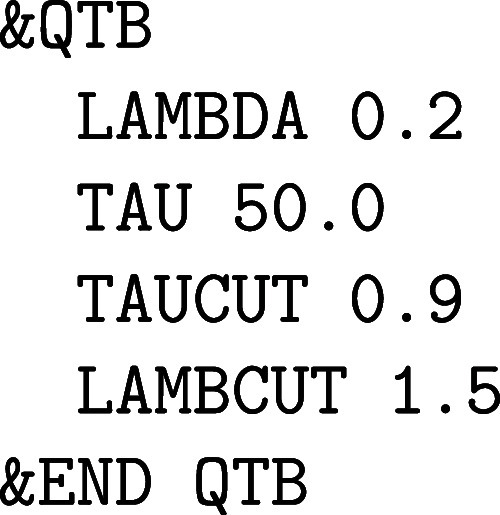


**Figure 3:**
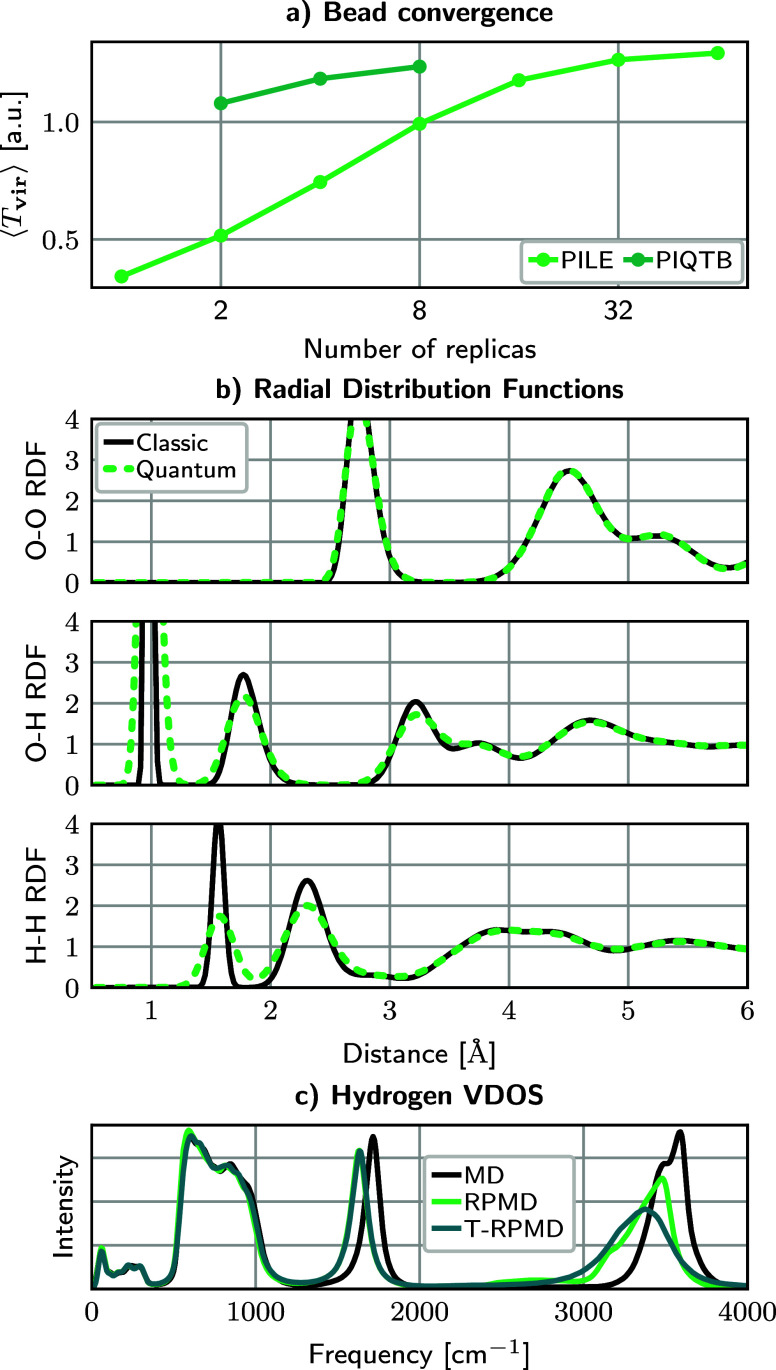
Impact of NQEs on the structure and dynamics of hexagonal ice at 250 K. (a) Bead convergence of the virial kinetic energy estimator using the PILE and PIQTB thermostat. (b) Radial distribution functions with classical and quantum nuclei and (b) vibrational density of states obtained with classical MD, RPMD and TRPMD, respectively. All simulations rely on using the C-NNP trained with revPBE0-D3 reference data,^322^ as available in the CP2K data repository (see also Section 10.1).

Both of these quantum thermostats have been shown to significantly reduce the number of beads required for convergence in PI simulations from ambient down to ultralow temperatures.^360^ We note in passing that they can also be used in the limit of *P* = 1 in CP2K, where they impose the frequency-dependent enhancement of the quantum fluctuations of a harmonic oscillator on the classical system to mimic quasi-classical quantum fluctuations. For PIGLET, this is supported via a dedicated thermostat section &GLE, while for PIQTB, the same thermostat section &QTB is simply used in combination with setting *P* = 1.

### 10.4 Approximate Quantum Dynamics

Within the standard PI formalism, MD is usually only performed as a sampling tool for the quantum partition function in the spirit of an extended Lagrangian formalism.^7,9,351,352^ However, there are several PI formulations that allow for the approximate description of quantum dynamics, such as CMD^366^ and RPMD.^367^ Both of these techniques can be seen as approximations to Matsubara dynamics,^368^ which is the exact quantum dynamics in the PI formalism. For more details on the theoretical footing of these methods, we refer the reader to refs 369–371 . Both CMD and RPMD are available in CP2K and are the only practical methods that allow one to approximately include NQEs on dynamics in (chemically complex) condensed phase systems. Unfortunately, both methods have been shown to suffer from fundamental artifacts, namely CMD from the curvature problem and RPMD from the chain resonance problem;^372^ thermostated RPMD (TRPMD) has been introduced to reduce the resonance problem by adding friction^373^ (see below). We note in passing that more recently, Brownian chain MD (BCMD)^374^ has been introduced, which is a short-time step version of the PI hybrid MC (PIHMC) technique^375^ applied to all noncentroid modes (whereas the centroids are propagated using Newtonian dynamics). Interestingly, BCMD has been shown^374^ to eliminate the resonance problem of RPMD and to alleviate the curvature problem of CMD.

The RPMD formalism requires switching the keyword PROPAGATOR in the &PINT section to RPMD. First, RPMD simulations in the *NVT* ensemble should be performed to generate initial configurations and velocities for subsequent *NVE* simulations to generate the actual RPMD dynamics. To speed-up structural thermalization, it is possible to start with standard PIMD *NVT* simulations and then switch to RPMD, resampling the velocities, while keeping the equilibrated configuration. In addition, CP2K also supports the TRPMD method,^373^ which is a variant of RPMD that applies a stochastic thermostat to all ring-polymer modes, except for the centroid mode. This idea is an ad hoc approach to remove spurious resonances of the ring-polymer modes with the true modes of the system, first discussed in detail in ref 372, by adding noise. We note that the TRPMD approach usually leads to peak broadenings and line shape distortions in vibrational spectra due to the influence of the thermostat. In CP2K, TRPMD can be simply enabled by setting the TAU parameter in the &PILE subsection to a negative value. An example input section for TRPMD to reproduce the results of Figure 3c is shown here, where RPMD dynamics would be enabled by removing the &PILE subsection:
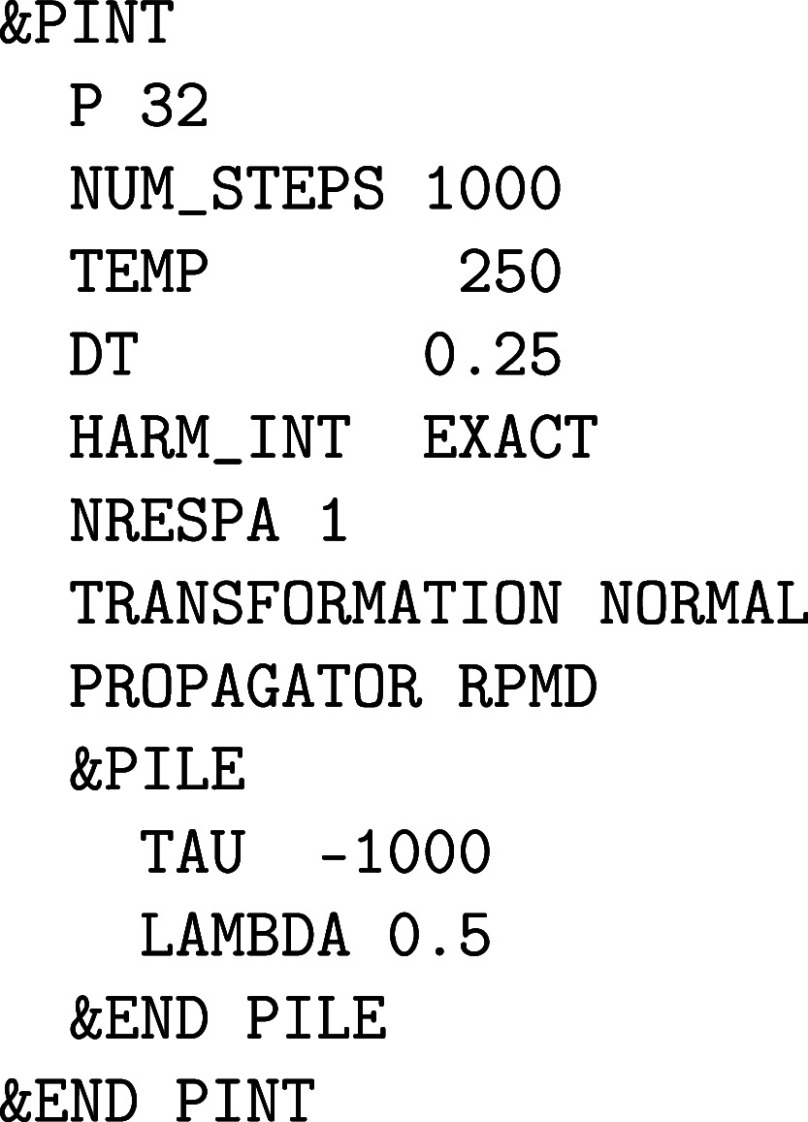


To conduct CMD simulations, the keyword &PINT%PROPAGATOR has to be set to CMD. Partially adiabatic CMD (paCMD) shifts the noncentroid degrees of freedom to a high-frequency regime in order to sample the average force of the centroid used for the propagation of the centroid. In CP2K, paCMD is controlled by the GAMMA parameter in the &NORMALMODE subsection which is a mass-scaling factor for the noncentroid degrees of freedom.^372^ It is crucial to reduce the time step in CMD simulations given the very high frequency of the noncentroid degrees of freedom. We recall that CMD results in artificial temperature-dependent red-shifts of vibrational frequencies due to the well-known curvature problem.^372,376^

Despite their known limitations and approximations,^370,372^ CMD and RPMD are powerful tools to generate approximate quantum dynamics in the PI formalism. They can be used to study a wide range of condensed phase systems, as well as of course gas phase systems, to compute not only vibrational spectra, but also rate constants, relaxation times, and transport properties. In particular, these methods can be easily combined with the above-described NNP module to study the quantum dynamics of complex systems, as recently demonstrated for liquid water at coupled-cluster (CC) accuracy^300^ including quantification of H/D isotope effects on translational and orientational dynamics,^377^ as well as on hydrogen bond kinetics.^378^

We illustrate the capabilities of the PI formalism in CP2K with a study of hexagonal ice I_h_ at 250 K using the C-NNP model introduced in Section 10.1. Bead convergence of the virial kinetic energy estimator, which illustrates the quantum nature of the system, is shown in Figure 3a. While the virial kinetic energy converges with roughly *P* ≈ 32 at 250 K using the PILE thermostat, we get a significant improvement in convergence using the PIQTB thermostat presented in Section 10.3. We note here that although 32 replica suffice to converge NQEs in liquid water at 300 K (as explicitly demonstrated in Figure S1 of ref 377 using the CCSD­(T) C-NNP from ref 300), that number of beads is on the smaller side for crystalline water at 250 K and certain properties require more replica for convergence. The radial distribution functions in Figure 3b reveal the well-known structural differences imprinted by the quantum nature of the system. Finally, the vibrational density of states obtained from standard MD with classical nuclei, as well as from RPMD and TRPMD in Figure 3c shows the impact of NQEs on the vibrational spectrum. While all quantum spectra are red-shifted compared to the classical spectrum, the RPMD spectrum features some additional peak modulations (namely a red-wing shoulder of the OH stretching peak and artificial vibrational intensity between that stretch and the bending band) due to artificial resonances of the fictitious ring-polymer modes with the physical vibrational modes of the system. The TRPMD spectrum, on the other hand, is seen to reduce the chain resonance problem, but shows an unphysical broadening of the peaks due to the influence of the thermostat on the ring-polymer modes as alluded to in Section 10.4. In summary, the PI formalism in CP2K provides a powerful tool to study the quantum nature of nuclei in a wide range of systems, in particular if combined with NNPs.

### 10.5 Bosonic Quantum Solvation at Ultralow Temperatures

So far, our discussion of NQEs has been restricted to particles that are assumed to be distinguishable even if they are identical. This implies that all nuclei in PIMD-based simulations of such systems can be numbered akin to classical particles, which results in Maxwell–Boltzmann (MB) quantum statistics. In MB quantum statistics, all nuclei are subject to quantum delocalization and tunneling effects according to their mass, in stark contrast to classical particles. In nature, however, identical particles are fundamentally indistinguishable. Their many-body WF, or finite-temperature density matrix ρ­(*R*,*R* ^′^,β), must be totally symmetric or antisymmetric in case of identical bosonic or Fermionic particles, according to the spin-statistics theorem. The density matrix can be formally symmetrized or antisymmetrized by introducing a sum over all possible permutations {P} of identical particles, i.e.

137 $$\rho_{FD}^{BE}\left(R,R^{\prime },\beta \right)=\frac{1}{N!}\sum_{P}{\left(\pm 1\right)}^{P}\rho \left(R,PR^{\prime },\beta \right)$$

where the positive and negative sign refer to bosons and Fermions, respectively; *R* are all particle coordinates and β = 1/*k*_B_ *T*. The resulting statistics are called Bose–Einstein (BE) and Fermi–Dirac (FD) quantum statistics; we refer to ref 7 in the context of the molecular simulation approach to quantum statistical mechanics.

In the framework of numerical PI simulations of BE and FD statistics, this implies that the discretized density matrix used to compute the partition function and all properties needs to be explicitly symmetrized or antisymmetrized with respect to the exchange of all identical particles.^7,346^ Therefore, an additional sum must be sampled in permutation space since the final coordinates of some particle along its quantum path might be some permutation of the initial coordinates, as illustrated here for the partition function that describes three identical particles:
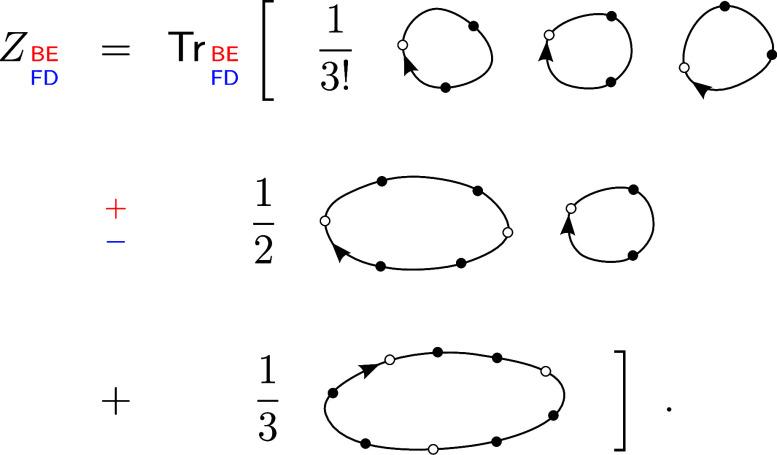


In case of bosonic systems such as ^4^He, all terms contribute with a positive sign to the symmetrized trace,^379^ while the odd permutations carry a negative sign in the Fermionic case; we mention in passing that this gives rise to the infamous “sign problem” in equilibrium PI simulations of many-body Fermionic systems such as electrons.^380,381^

The pioneering bisection PIMC method to sample the bosonic permutations, which are required in PI simulations of superfluid ^4^He, has been introduced four decades ago using a Lévy construction;^382,383^ we refer to ref 349 for an exhaustive review on this technique. Two decades later, the continuous/space worm algorithm for PIMC simulations of bosons in the grand-canonical ensemble has been devised to greatly improve the sampling efficiency, in particular for large bosonic systems using open path (“worm”) sampling that involves off-diagonal elements of the density matrix.^384,385^ In CP2K, a canonical worm algorithm with specific sampling extensions, which are relevant for finite bosonic clusters of a given particle number subject to strong interactions of the ^4^He atoms with impurities, has been introduced and implemented, as described in the appendix of ref 386. It therefore represents the default technique to simulate bosonic exchange and thus superfluid properties when using CP2K.

As a technical aside, we recall that sampling bosonic exchange is characterized by nonpolynomial complexity and, therefore, gets increasingly demanding the larger the bosonic system is, even when using efficient worm sampling. For efficiency reasons, not only cubic, but also truncated octahedral boundary conditions have been implemented in CP2K for the solvation supercell. The latter type allows for a smaller overall volume and thus a smaller necessary number of bosonic solvent species to describe a fixed spherical internal volume. For superfluid fraction calculations, the winding number estimator (see below) has been properly generalized for the noncubic supercell shape.^387^ In CP2K, the truncated octahedral supercell is activated as follows in conjunction with the use of canonical worm sampling:
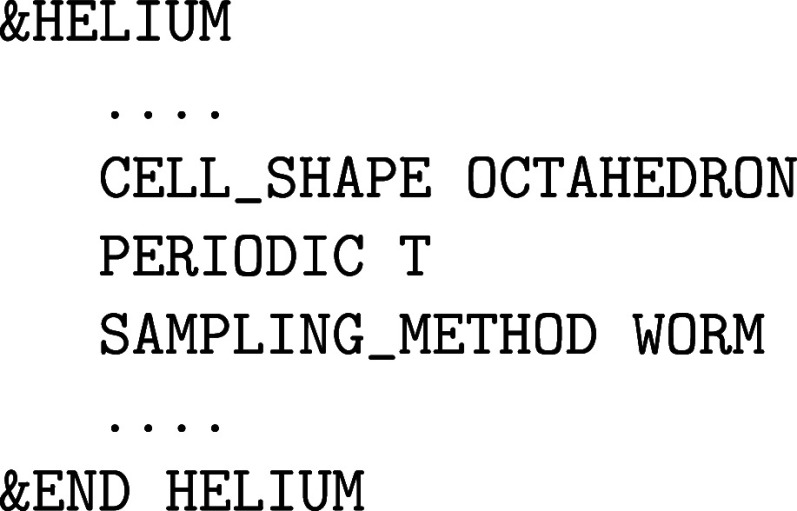


Apart from sampling the exchange of all identical particles via path permutations, PI simulations of superfluid helium require one to reach temperatures on the order of 1 K or less. In traditional PIMC or PIMD simulations, some high-temperature density matrix is used that, by applying it many times in a product form, brings the system down to the desired temperature. Due to their simplicity, one-body high-temperature density matrices together with the primitive Trotter approximant are employed in most simulations including NQEs using PIMD-based methods, as discussed in Sections 10.2 and 10.3, in the framework of mNHC, PILE, PIGLET, PIQTB, CMD, RPMD or TRPMD thermostats, all of which are implemented in CP2K.

In the realm of PIMC simulations at ultralow temperatures, say on the order of 1 K or even less, the numerical pair density matrix approach has been introduced decades ago^347^ to greatly reduce the number of PI beads or replica that are needed to converge the discretized PI to its continuum limit in the case of two-body interactions,^388^ as reviewed earlier.^349^ Transcending primitive approximants, the high-temperature many-body density matrix is represented in this case in terms of one- and two-body contributions to the total PI action, where the latter can be exactly computed for all individual pairs of bosons that interact by a known two-body (pair) potential. In CP2K, we provide a numerical fit^349^ of the ^4^He···^4^He pair density matrix computed at a “high temperature” of 80 K, using a very accurate two-body interaction potential,^389^ which has been computed on a regular grid and subsequently spline-tabulated for its efficient use. Based on this pair density matrix, a PI discretization of only *P* = 80 replica suffices to perform converged PIMC simulations of bulk superfluid helium at a temperature of 1 K using the CP2K internal implementation:
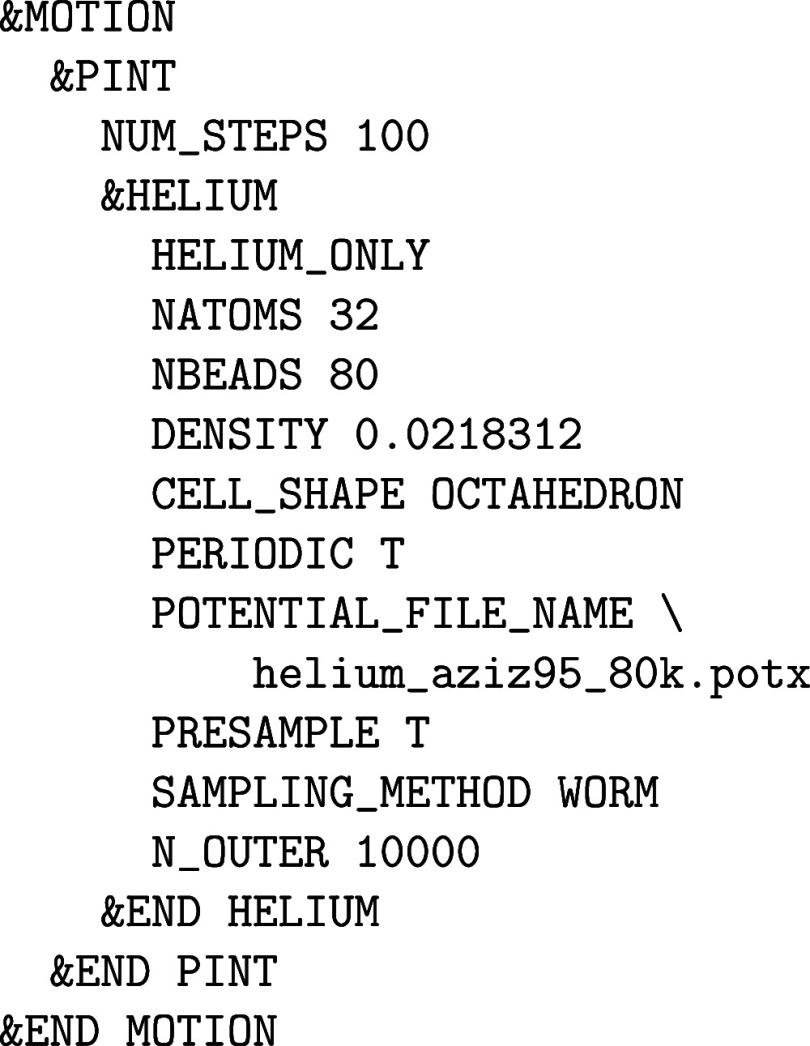


Note that for converged observables, many more steps than the 100 selected above need to be simulated, and the necessary pair density file helium_aziz95_80k.potx can be found in the tests/Pimd/untested_inputs directory of CP2K.

The superfluid fraction *f*_s_ is a key property (order parameter) that characterizes the phase transition from the normal liquid state of bosons, where *f*_s_ = 0, to its superfluid phase, where *f*_s_ → 1 as *T* → 0 K. The CP2K output also allows one to compute, in a postprocessing step, the superfluid fraction of bulk systems and the associated superfluid density ρ_s_ using the winding number estimator^349,383^

138 $$f_{s}=\frac{\rho_{s}}{\rho_{tot}}=\frac{m_{He}\left\langle W^{2}\right\rangle }{\beta \hbar^{2}N_{He}}$$

where *W* is the winding number that counts how many times a given path wraps around the periodic supercell along a given direction multiplied by its box length in that direction; all other variables are self-explanatory. The output of this quantity is controlled via the &WINDING_NUMBER subsection inside the &PINT%HELIUM%PRINT section. The output consists of the numerical value of the prefactor of eq 137 as the initial comment line and the vectorial output of *W* for each requested time step. Alternatively, one can turn on the output of the mean squared of the simulation so far via the &WINDING_NUMBER_2_AVG subsection. Also, this output contains the necessary prefactor value. Note that in both cases, the prefactor in the files is without the 1/*N*_He_ factor. After finite-size extrapolation, eq 137 provides the rigorous expectation value of *f*_s_ in bosonic bulk systems subject to periodic boundary conditions.^390^

We illustrate in Figure 4b the behavior of the superfluid fraction, as well as that of the heat capacity in Figure 4a, featuring the characteristic λ-shape as a function of temperature of bulk ^4^He once it gets cooled down from the normal liquid state below the λ-transition where superfluidity sets in note that *f*_s_ approaches unity at about 1 K. This calculation has been carried out using 32 ^4^He atoms (the complete input can also be found in the tests/Pimd/untested_inputs directory of CP2K) for the point at 1.6 K (20 independent runs of this type enter the plotted data for each temperature).

**Figure 4:**
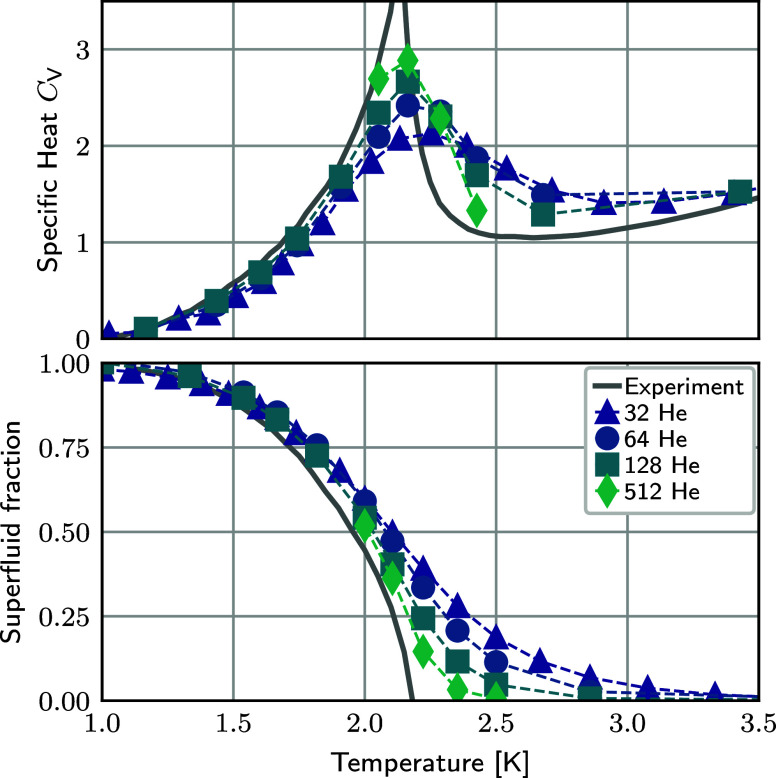
Temperature dependence of (a) the heat capacity and (b) the superfluid fraction of bulk ^4^He obtained using the canonical worm algorithm to sample bosonic exchange and the winding number estimator to analyze superfluidity (see text). The numerical pair density matrix is the one provided with the CP2K package and has been computed at 80 K using the He···He potential from ref 389, thus *P* × *T* = 80 K is kept constant for the temperature scan. The present bosonic PIMC data were obtained from only 32 atoms (in a truncated octahedral supercell according to the sample input provided in the text) and are compared to published results of ref 386 generated from 64, 128, and 512 atoms in the corresponding periodic truncated octahedron using CP2K and the same methodology. For reference, the respective experimental data^391,392^ are shown by gray lines; note that deviations of the computed properties from experiment are mainly due to finite-size effects close to the superfluid phase transition given such small system sizes.^390^

For finite systems, as particularly relevant in the case of molecular impurities solvated in bosonic environments at very low temperatures to be introduced in the following, there is no rigorous expression available to compute the superfluid fraction. In CP2K, the area estimator^393^ is the default for good reasons, i.e.

139 $$f_{s}=\frac{\rho_{s}}{\rho_{tot}}=\frac{4m_{He}^{2}\left\langle {\left(\mathrm{n⃗}\cdot \mathrm{A⃗}\right)}^{2}\right\rangle }{\beta \hbar^{2}I_{c}}$$

where n⃗·A⃗ is the projected area with

140 $$\mathrm{A⃗}=\frac{1}{2}\sum_{i=1}^{N_{He}}\sum_{s=1}^{P}{\mathrm{r⃗}}_{i}^{\left(s\right)}⊗{\mathrm{r⃗}}_{i}^{\left(s+1\right)}$$

along some direction n⃗ and

141 $$I_{c}=\left\langle \frac{1}{P}\sum_{i=1}^{N_{He}}\sum_{s=1}^{P}m_{i}\left(\mathrm{n⃗}⊗{\mathrm{r⃗}}_{i}^{\left(s\right)}\right)\cdot \left(\mathrm{n⃗}⊗{\mathrm{r⃗}}_{i}^{\left(s+1\right)}\right)\right\rangle$$

is the respective classical moment of inertia.

Obtained from linear-response theory of macroscopic systems, the area estimator works convincingly for large systems but, unfortunately, provides a clearly nonzero superfluid fraction in the limit of simulating a single boson, which is obviously an unphysical artifact. The reason is that this estimator is based on computing the mean-squared area of paths in some plane, which is nonvanishing even for one boson where no exchange paths exist. As a remedy, the exchange estimator has been introduced.^394,395^ It computes the so-called exchange superfluid fraction by essentially renormalizing the projected area obtained in BE simulations with the one computed from corresponding MB simulations, where no paths exchange and yet result in nonzero n⃗·A⃗ contributions. In CP2K, the exchange estimator can be obtained after performing simulations identical to those sampling BE statistics, while switching off the MC permutation moves in the worm algorithm to provide the respective MB averages necessary to correct the area estimator (see Appendix C.1 in ref 396 for details).

The sampling scheme of the hybrid PIMD/bosonic PIMC (HPIMD/MC) algorithm^386,387,397^ to treat quantum solvation of fully flexible and reactive molecular species in bosonic environments (such as those provided by ^4^He), as available in the CP2K package, is presented in Figure 5.

**Figure 5:**
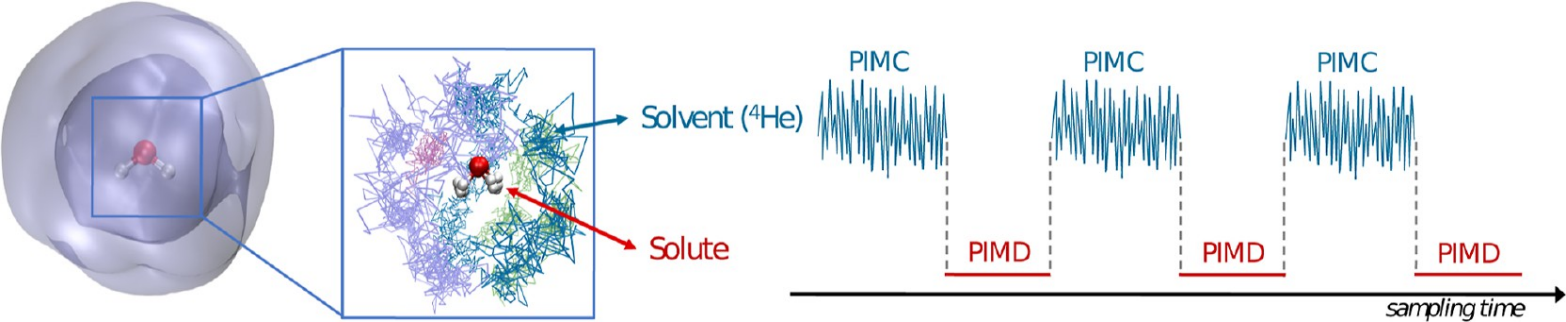
For an illustration of the HPIMD/MC method, as implemented in CP2K.^386^ The fully flexible and reactive solute species are propagated using PIMD techniques (such as PIQTB in conjunction with the one-body density matrix), while the bosonic solvent (for instance many ^4^He atoms either forming a finite cluster as illustrated here, or hosted within a periodic supercell) is sampled using bosonic PIMC (for instance based on the canonical worm algorithm in conjunction with the numerical pair density matrix) at a common temperature *T* (see text). The interactions can be described using high-dimensional NNPs with CCSD­(T) accuracy, as described in Section 10.1.

In this approach, the molecular species and the bosonic solvent are sampled using PIMD and PIMC subject to permutation moves, respectively. After completing one PIMD step of the molecule, where the forces on the molecule are obtained from the intramolecular potential *U*_mol_, as well as those coming from the solute–solvent interaction potential *U*_int_, i.e.

142 $${\mathrm{F⃗}}_{PIMD}=-{\nabla ⃗}_{{\mathrm{r⃗}}_{mol}}U_{mol}\left(\{{\mathrm{r⃗}}_{mol}\}\right)-{\nabla ⃗}_{{\mathrm{r⃗}}_{mol}}U_{\mathrm{int}}\left(\left\{{\mathrm{r⃗}}_{mol}\right\},\left\{{\mathrm{r⃗}}_{He}\right\}\right)$$

a bosonic PIMC simulation is then performed for the solvent species alone, for example ^4^He. This includes the exchange moves to establish BE statistics of the bosonic solvent environment, while the molecule is kept frozen after the previous PIMD step, as illustrated in Figure 5. It is important to carry out sufficiently many bosonic PIMC steps such that the bosonic environment can adapt to the current configuration of the molecule in both the real and permutation space before the next PIMD step is performed. The required bosonic PIMC chains are generated based on the change of the potential

143 $$U_{PIMC}=U_{He}\left(\{{\mathrm{r⃗}}_{He}\}\right)+U_{\mathrm{int}}\left(\left\{{\mathrm{r⃗}}_{mol}\right\},\left\{{\mathrm{r⃗}}_{He}\right\}\right)$$

due to the solute–solvent interactions *U*_int_ together with the solvent–solvent pair potential interactions (such as $U_{He}\left(\{{\mathrm{r⃗}}_{He}\}\right)$ to describe helium^389^) as a evaluated during the Metropolis step. At this point, the bosonic permutations can be sampled in CP2K using either the bisection method,^349^ or our canonical worm algorithm,^385,386^ the latter being recommended for most cases. At the end of the PIMC chain, the next PIMD step is carried out using the new solvent position to compute ${\mathrm{F⃗}}_{PIMD}$ , followed by the next bosonic PIMC sweep, and so forth. This particular PIMD–PIMC coupling scheme and propagation algorithm establish the correct quantum *NVT* ensemble of the total solute-in-solvent system.^386^ The HPIMD/MC approach implemented in CP2K has been generalized to also deal with molecular bosons, such as para-H_2_ or ortho-D_2_ molecules, in the framework of the adiabatic hindered rotor (AHR)^395^ averaging technique.^398^

At this point, a decision has to be made with respect to the intramolecular potential energy surface *U*_mol_ and the solute–solvent interaction potential *U*_int_ to describe fully flexible and reactive molecular solutes in a quantum solvent, while an accurate solvent–solvent pair potential $U_{He}\left(\{{\mathrm{r⃗}}_{He}\}\right)$ is readily available in parametrized form.^389^ In the original HPIMD/MC approach,^387^ the ab initio PI philosophy^9,399,400^ has been adapted to compute *U*_mol_ on-the-fly, while *U*_int_ has been fitted to accurate CC quantum chemistry data in the classic sense of a physics-based interaction potential;^401,402^ this approach has been successfully applied to various systems^397,403,404^ using CP2K. This technique has been superseded by the use of NNPs, as explained in Section 10.1, which offer two advantages at the same time, namely allowing for highly efficient PI sampling and providing high accuracy of the intra- and intermolecular interactions.^360^ The current NNP-based HPIMD/MC approach in CP2K thus allows one to converge the PI even at ultralow temperatures (using techniques summarized in Section 10.3) and achieves essentially basis set converged CCSD­(T) accuracy;^360^ we note in passing that such CC quality PIMD simulations, dubbed CCMD, have been generalized more recently to condensed phase systems.^300,377^

The generation and use of NNPs in CP2K have been reviewed herein in Section 10.1, see there for general background and input options, which transfers to applications of the HPIMD/MC method. But in this case, additional challenges arise since the intramolecular potential energy surface of finite molecular systems, as well as intermolecular interaction potentials are required at very high (“quantum chemical”) accuracy, while analytic gradients and thus forces are not efficiently available in the framework of CC theories. The situation is distinctly different from usual applications of MLPs in general and NNPs in particular to condensed phase systems, such as water and ice presented in Section 10.1, which are based on computationally economic periodic DFT calculations, where analytic forces are readily available.

These challenges posed by HPIMD/MC have been addressed using hierarchical MLP sampling approaches to cope with intermolecular interactions and thus solvation^405^ combined with active learning protocols^298^ to efficiently parametrize both *U*_int_ and *U*_mol_ in the framework of NNPs, which are deeply interfaced with CP2K. In particular, NNP training codes such as the RuNNer, n2p2 or RubNNet4MD packages^316^ have been used in conjunction with quantum chemistry packages that offer efficient implementations of CCSD­(T) theory, such as Molpro^406^ and ORCA^407^ to generate on the order of 10 000 to 100 000 total energies of finite molecular systems in the relevant size regime to train, test and validate the MLPs for use in CP2K-based HPIMD/MC and CCMD simulations.^298,300,360,396,398,405,408–414^ The accuracy of the NNPs *U*_mol_ and interaction potentials *U*_int_ at the CCSD­(T) level of theory for the methane molecule (CH_4_), the protonated methane complex (CH_5_^+^), the hydronium cation (H_3_O^+^), and the Zundel cation (H_5_O_2_^+^) is illustrated in Figures 6 and 7, respectively. This rigorous end-to-end testing of the MLP representations of CCSD­(T) references energies of diverse systems in terms of NNPs validates the accuracy of the overall methodology used in CP2K to perform HPIMD/MC and CCMD simulations.^298,300,360,396,398,405,408–414^

**Figure 6:**
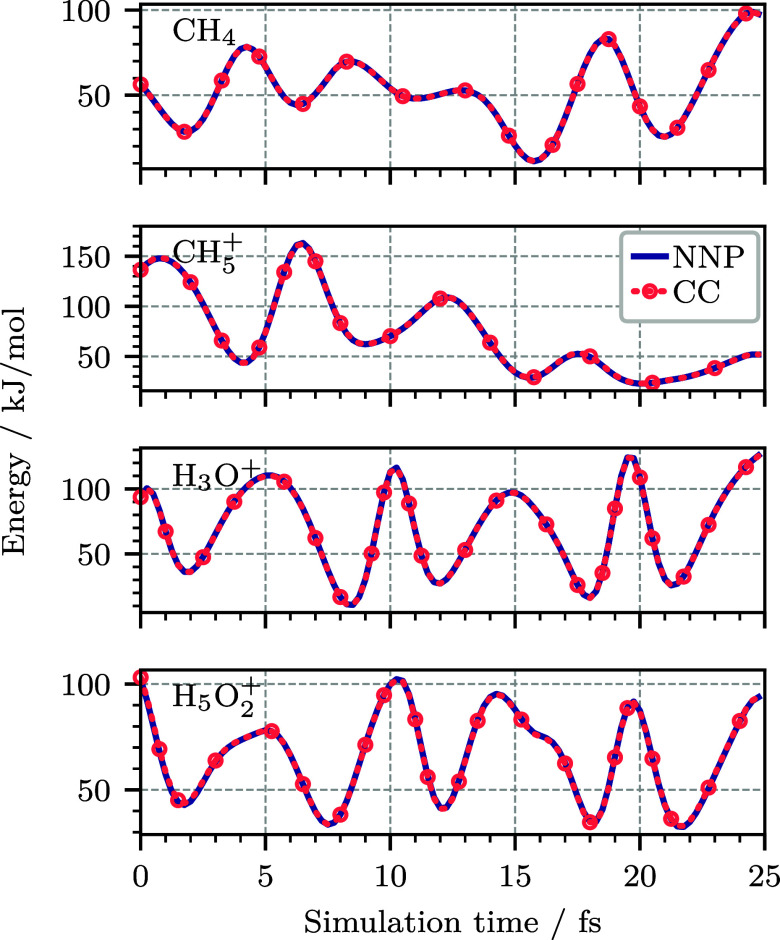
Potential energy *U*_mol_ along one Trotter replica of quantum PIMD trajectories of (from top to bottom) CH_4_, CH_5_^+^, H_3_O^+^, H_5_O_2_^+^, respectively, all at 1.67 K using NNPs trained with CCSD­(T*)-F12a/aug-cc-pVTZ reference energies obtained from Molpro. The corresponding CC single-point data were obtained by recomputing the energies at each and every step of the depicted NNP trajectories and are shown as red dotted lines. All energies are reported relative to the equilibrium structures of the respective global minima. Reproduced from Figure 6 of ref 386. Copyright 2020, American Institute of Physics.

We note in passing that dipole moment surfaces of finite molecular systems have also been generated with CCSD­(T) accuracy using a very similar systematic NN-based training approach,^415^ as implemented in Version 2 of the RubNNet4MD package.^316^ Using CP2K, this enables the calculation of infrared (IR) absorption cross sections based on approximate RPMD (or TRPMD or CMD) quantum dynamics, as described in Section 10.4, in order to include NQEs in such vibrational spectra consistently at CCSD­(T) quality.^414^

The HPIMD/MC method implemented in CP2K allows one to investigate fully flexible and reactive solutes, such as molecules, complexes or clusters, in finite and bulk-like bosonic environments, such as superfluid ^4^He or para-H_2_ species, at relevant temperatures on the order of 1 K (Figure 7). Here, a typical CP2K input section is provided to simulate a protonated water molecule embedded in a helium cluster consisting of 8 ^4^He atoms (i.e., H_3_O^+^·^4^He_8_) at a temperature of 1 K:
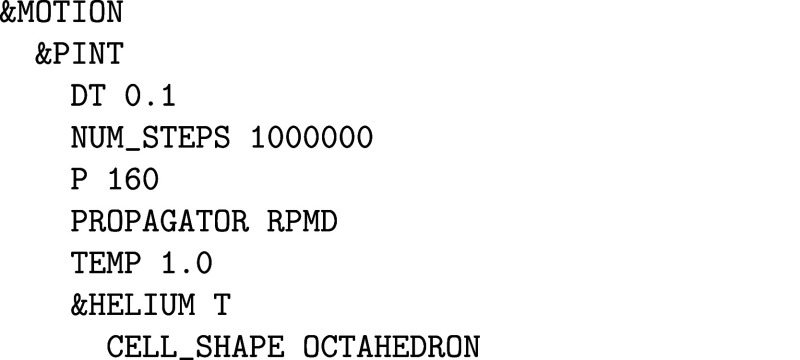
 
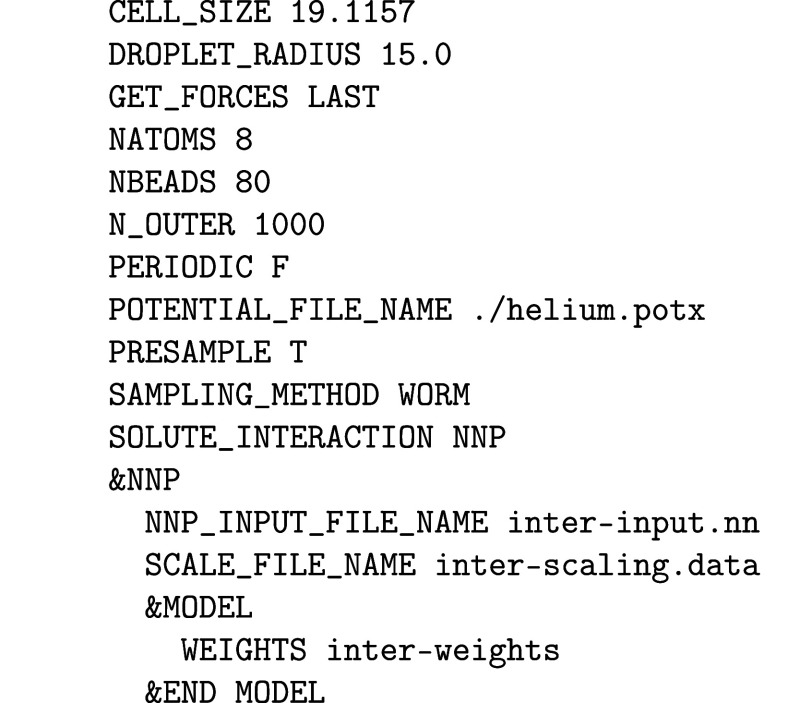
 
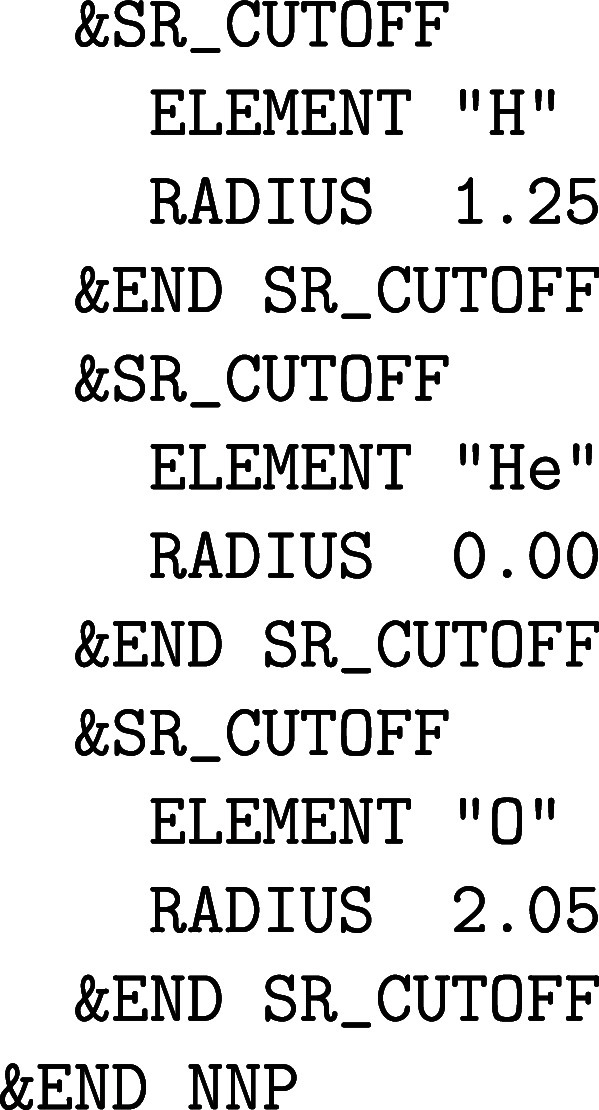
 
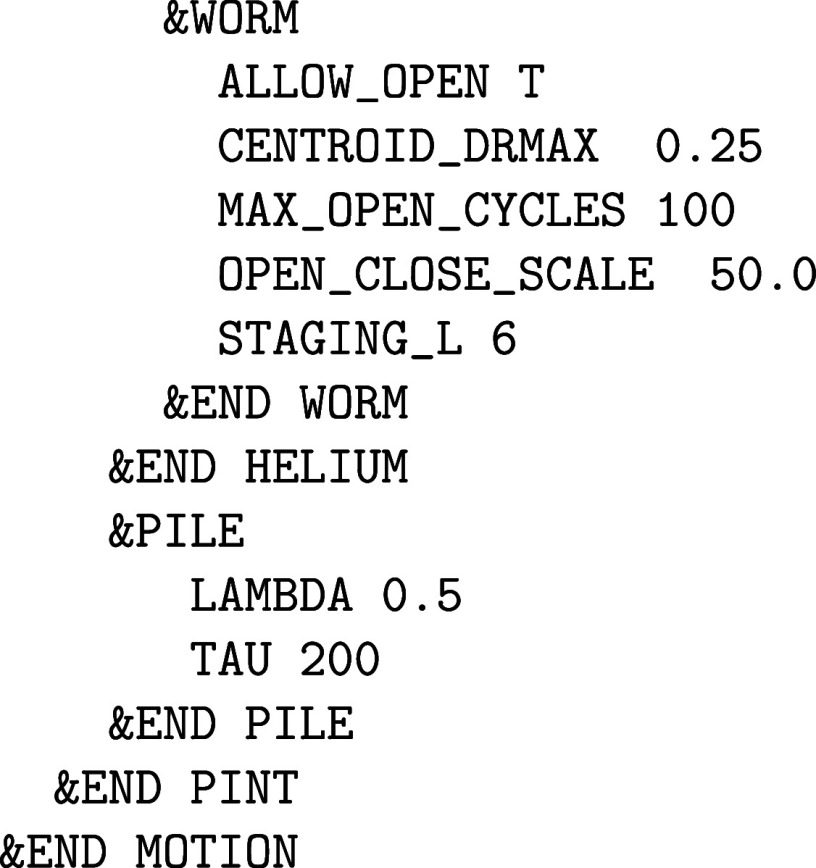
 
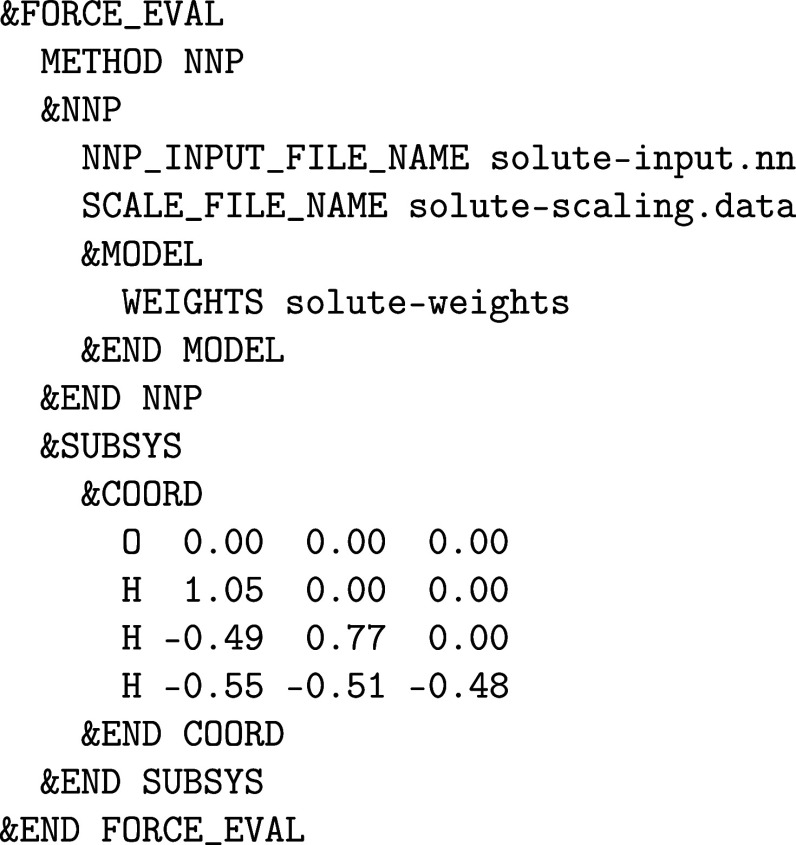


**Figure 7:**
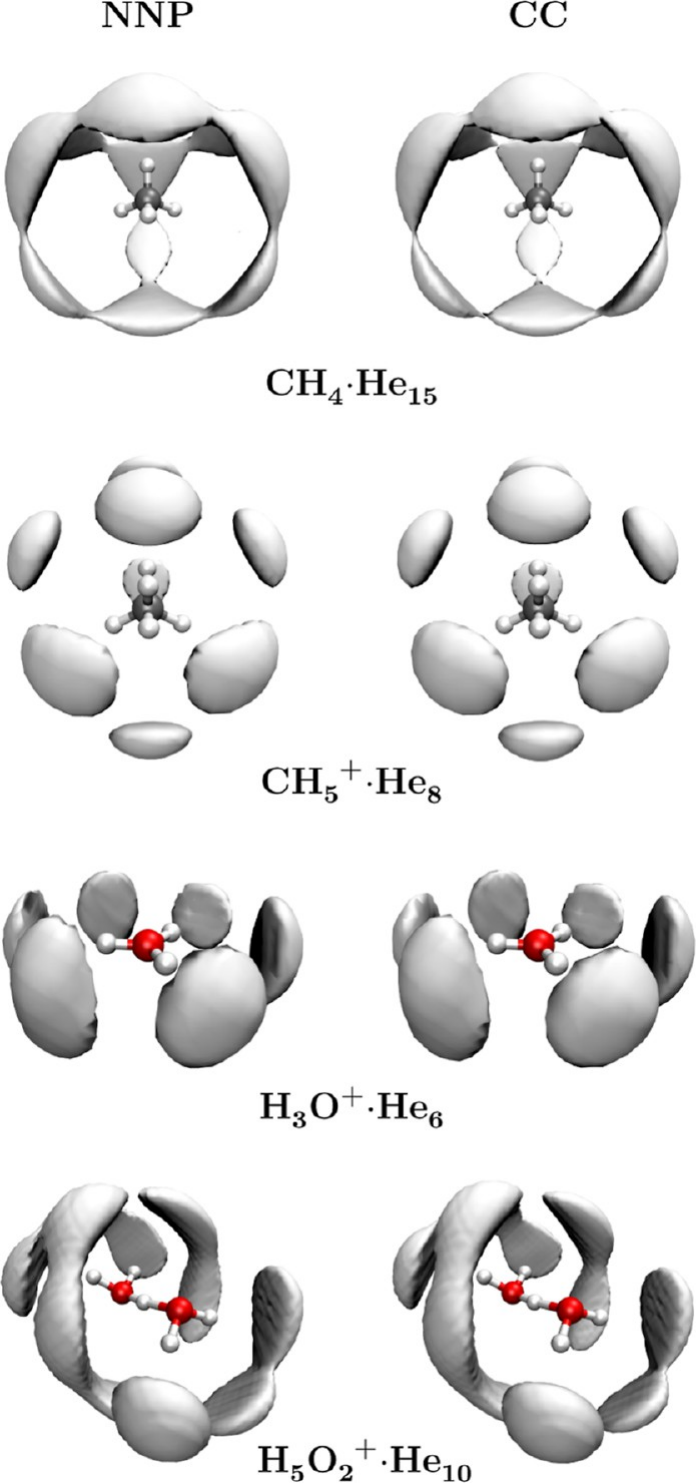
Spatial distributions functions stemming from all *N* helium atoms around a fixed molecular impurity (i.e., *X*·He_*N*_), as sampled from PIMC simulations (without bosonic exchange) at 1.67 K. The solute···helium interaction energies *U*_int_ used in the PIMC sampling of the helium atoms are interpolated from values that have been precomputed on a grid using either the NNP (left column) or single-point CC calculations (right column) on the identical grid both based on counterpoise-corrected CCSD­(T*)-F12a/aug-cc-pVTZ energy differences obtained from Molpro. Reproduced from Figure 7 of ref 386. Copyright 2020, American Institute of Physics.

This simulation uses the numerical ^4^He···^4^He pair density matrix at 80 K obtained from the very accurate two-body interaction potential of Aziz to describe *U*_He_;^389^ the resulting PIMC discretization of the bosonic environment relies on 80 replica. The employed intramolecular and intermolecular potentials *U*_mol_ and *U*_int_, are NNP-based representations of reference energies at the CCSD­(T) level of theory (obtained from the Molpro package^406^) that have been trained using the RubNNet4MD package^316^ and are available from the CP2K GitHub repository. For PIMD sampling of the solute using the RPMD approach, a bead number of 160 is used. This relies on the striding approach, which allows one to couple two PIs with different Trotter discretizations, as visualized in Figure 8. In HPIMD/MC, the efficient pair density matrix approach allows one to converge the solvent PI using a rather small replica number (here 80 at 1 K), which is not sufficient to represent the solute PI. Using a stride of two and thus 160 replicas to represent the solute interactions, versus 80 for the solvent–solvent interactions and thus the solute–solvent interactions, as explained in section III.C of ref 386 and illustrated schematically in Figure 8, is appropriate. The intramolecular O–H, as well as the intermolecular O–He and H–He distance distribution functions resulting from the input example provided are presented in Figure 9. The corresponding superfluid fraction of the bosonic helium environment, as obtained with the exchange estimator, amounts to *f*_s_ ≈ 0.326 ± 0.012. This technique is available in CP2K and can be readily used to study the effects of bosonic quantum solvation on fully flexible and reactive molecular solute species embedded in ^4^He and also in para-H_2_ environments at temperatures on the order of 1 K,^398^ where quantum exchange occurs and thus BE statistics emerge, resulting in strictly nonzero superfluid fractions and densities.^396,403,404,411,413^

**Figure 8:**
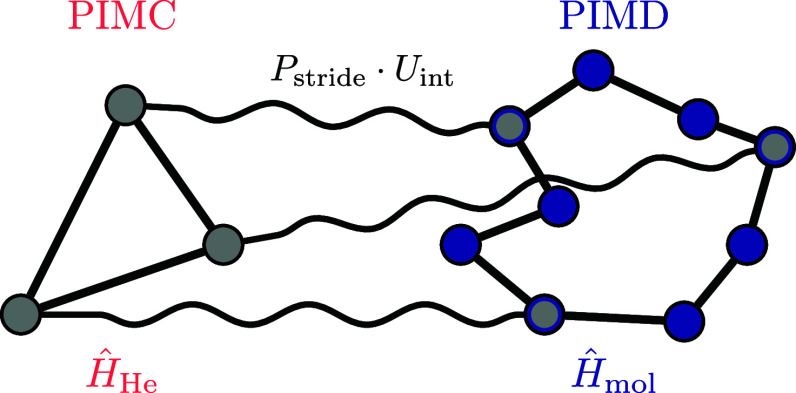
Illustration of PI striding for bosonic HPIMD/MC simulations, as implemented in CP2K: coupling of the PI representation of one helium atom (left) discretized with three Trotter replica (in practice sampled using PIMC based on the pair density matrix representation) to the PI representation of the solute molecule (right) discretized with nine beads (sampled using PIMD based on the one-body density matrix in conjunction with PIQTB thermostatting, as detailed in Section 10.3), thus corresponding to a striding length of three, *P*_stride_ = 3. Reproduced from Figure 3 of ref 386. Copyright 2020, American Institute of Physics.

**Figure 9:**
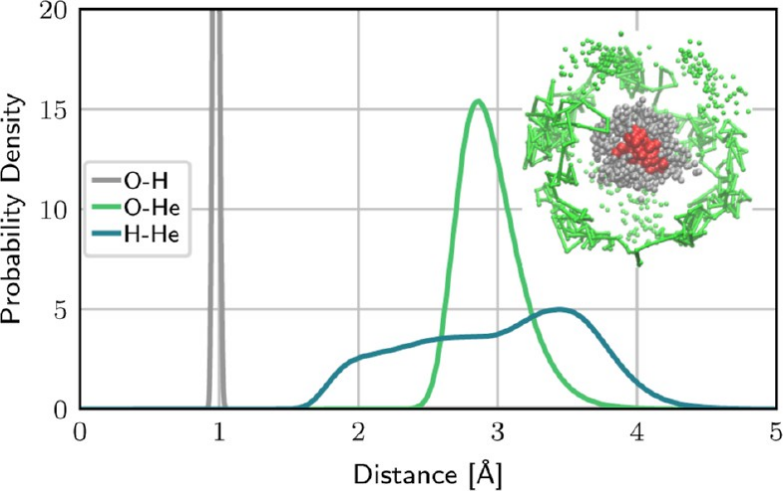
Distance distributions functions of H_3_O^+^·^4^He_8_ at 1 K with BE sampling of the helium microsolvation environment. The intramolecular O–H, as well as the intermolecular O–He and H–He distance distributions are presented. The inset depicts the beads of the hydronium ion in terms of balls (red/gray for O/H), while all ^4^He atoms involved in a bosonic exchange cycle are visualized by green strings and all others by green balls.

### 10.6 Vibrational Spectroscopy

Vibrational spectroscopy is an important field of chemistry and physics since a very long time. As molecular vibrations (or phonons in solid state materials) are highly sensitive to the chemical environment of a certain molecule, vibrational spectra allow one to study the interactions present in the sample in great detail.

There exist two fundamentally different approaches for predicting vibrational spectra: the so-called “static-harmonic approximation”, and the “time-correlation function (TCF)” techniques. Though CP2K directly implements the former approach, it is able to efficiently compute all necessary quantities of the latter method too, so that vibrational spectra can be easily obtained from postprocessing tools such as ASE^416^ and TRAVIS.^417,418^

#### 10.6.1 Static-Harmonic Approach

It is possible to predict the vibrational modes of an atomistic system by approximating it as a system of harmonic oscillators. Assuming a reasonably smooth potential energy surface, the potential energy *V*(**x**) of a set of *N* atoms in close proximity to some point **x** ^0^ with respect to the atoms’ 3*N* Cartesian coordinates **x** = (*x*_1_, ..., *x*_3*N*_) can be approximately expressed in terms of a second-order multidimensional Taylor expansion

144 V(x)≈V(x0)+∑i=13N(∂V∂xi)x0(xi−xi0)+12∑i=13N∑k=13N(∂2V∂xi∂xk)x0(xi−xi0)(xk−xk0)

If the point **x** ^0^ is an energy minimum, the gradients ${\left(\frac{\partial^{2}V}{\partial x_{i}}\right)}_{x^{0}}$ vanish, and only the second derivatives remain, i.e.

145 $$V\left(x\right)\approx V\left(x^{0}\right)+\frac{1}{2}\sum_{i=1}^{3N}\;\sum_{k=1}^{3N}{\left(\frac{\partial^{2}V}{\partial x_{i}\partial x_{k}}\right)}_{x^{0}}\left(x_{i}-x_{i}^{0}\right)\left(x_{k}-x_{k}^{0}\right)$$

Based on this approximation, the force *F*_*i*_ acting on coordinate *x*_*i*_ can be expressed as

146 $$F_{i}=-\left(\frac{\partial V}{\partial x_{i}}\right)=-\frac{1}{2}\sum_{k=1}^{3N}{\left(\frac{\partial^{2}V}{\partial x_{i}\partial x_{k}}\right)}_{x^{0}}\left(x_{k}-x_{k}^{0}\right)$$

By inserting eq 146 into Newton’s equation

147 $$F_{i}=m_{i}a_{i}=m_{i}\left(\frac{d^{2}x_{i}}{dt^{2}}\right),,i=1,...,3N$$

one obtains a system of equations of motion

148 $$m_{i}\left(\frac{d^{2}x_{i}}{dt^{2}}\right)=-\sum_{k=1}^{3N}{\left(\frac{\partial^{2}V}{\partial x_{i}\partial x_{k}}\right)}_{x^{0}}\left(x_{k}-x_{k}^{0}\right)$$

To express these equations more compactly, it is desirable to switch to the set of so-called mass-weighted Cartesian coordinates **q** = (*q*_1_, ..., *q*_3*N*_) defined as

149 $$q_{i}:=x_{i}\sqrt{m_{i}}$$

in which eq 148 now reads as

150 $$\frac{d^{2}q_{i}}{dt^{2}}=-\sum_{k=1}^{3N}H_{i,k}q_{k},,i=1,...,3N$$

with the short-hand notation *H*_*i*,*k*_ for the mass-weighted Hessian matrix

151 $$H_{i,k}:=\frac{1}{\sqrt{m_{i}m_{k}}}{\left(\frac{\partial^{2}V}{\partial x_{i}\partial x_{k}}\right)}_{x^{0}}$$

As the above approximation defines a purely (see eq 145), one can assume that the motion of each coordinate *q*_*i*_ can be described by a sinusoidal time evolution

152 $$q_{i}\left(t\right)=q_{i}^{0}+A\cdot \cos\left(\omega t\right)$$

with some amplitude *A* and angular frequency ω. Inserting this into eq 150 yields

153 $$\omega^{2}q_{i}=\sum_{k=1}^{3N}H_{i,k}q_{k},,i=1,...,3N$$

which can be rewritten as a matrix eigenvalue problem for the mass-weighted Hessian matrix **H** with the eigenvalues ω^2^

154 $$\omega^{2}q=Hq$$

In other words, solving this eigenvalue problem directly yields the vibrational frequencies ω of the atomic system as the square roots of the eigenvalues. Furthermore, the eigenvectors **q** of the matrix represent the directions in which the atoms are displaced within each normal mode. Therefore, a full set of normal modes, each with a vibrational frequency and an atom displacement vector, can be obtained via this approach. Additional care has to be taken to project out the invariants (i.e., translational and rotational invariances due to the conservation of momentum and angular momentum) of the mass-weighted Hessian matrix, so that typically only (3*N* – 6) normal modes are obtained.

Such a normal-mode analysis is fully implemented in CP2K. Yet, as explained above, the approach only works for minimum structures, so it should be applied only to geometries that resulted from a converged optimization with very similar computational details. The normal-mode analysis can be requested as follows:
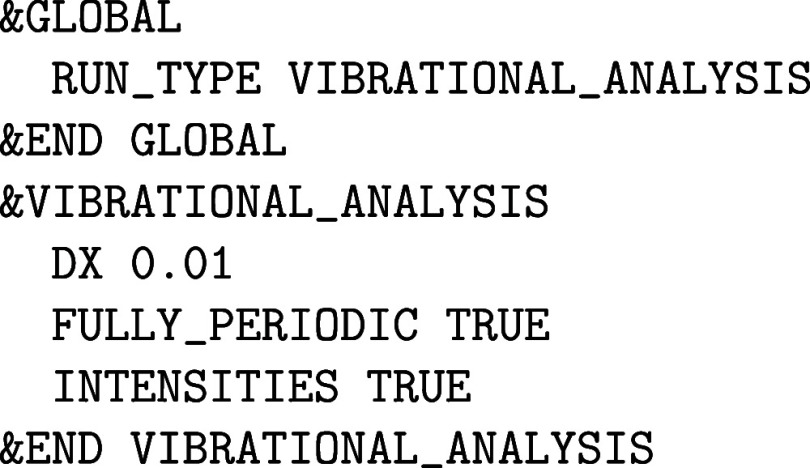


Note that CP2K computes the second derivatives of the energy with respect to the displacement (Hessian matrix) numerically via central finite differences, so that for a system of *N* atoms, 6*N* individual energy and force computations will be required. The DX parameter sets the displacement for the finite differences. Please also note that a very tight SCF threshold (EPS_SCF 1.E-8 in combination with EPS_DEFAULT 1.E-16) is required to reduce numerical noise. The FULLY_PERIODIC keyword inside the VIBRATIONAL_ANALYSIS section should be used for periodic bulk phase systems in order to switch off the pruning of the rotational degrees of freedom from the Hessian. Activating the keyword INTENSITIES facilitates the computation of IR or Raman intensities, but requires the explicit calculation of dipoles or polarizabilities by means of &DFT%PRINT%MOMENTS or &PROPERTIES%LINRES%POLAR, which are described in detail later. Moreover, adding the subsection &MODE_SELECTIVE permits running a so-called mode selective vibrational analysis, which constrains the calculation to either a single FREQUENCY, RANGE of frequencies, or a list of INVOLVED_ATOMS.

The derivation above only shows how to obtain the vibrational frequencies of the normal modes. However, in order to predict real vibrational spectra, the intensities of each mode also need to be computed. This is typically performed by computing derivatives of some more or less complicated electromagnetic properties with respect to the atom displacements **q** obtained for each mode. For example, the IR intensity of a certain mode is proportional to the change in the electric dipole moment that occurs when the atoms are displaced along that mode. More details on computing such properties are discussed in the following subsection.

Despite still being the standard approach in the literature, computing spectra via the static-harmonic approach comes with several shortcomings:

- Due to the harmonic approximation of the potential energy surface, all anharmonic effects are neglected.^419^ If the system possesses features such as strong hydrogen bonds or hindered rotations, the harmonic approximation of certain modes will be poor, and so will the quality of the predicted spectrum.
- The spectrum can only be computed for one minimum energy structure at a time. If there exist several conformers of the same molecule, they need to be considered separately. If the system can hardly be described by minimum-energy structures, such as bulk phase liquids, it will be hard to obtain reasonable spectra from the outset.
- The method works best for molecules or small clusters in vacuum. Solvent effects on the spectrum can be crudely approximated either via continuum solvation models such as COSMO,^420^ PCM,^421^ or the SCCS implicit solvation method described in Section 4.1,^135–137^ as well as by microsolvation, but the solvent effect cannot be captured comprehensively.
- The approach only yields a discrete line spectrum; no line widths or band shapes can be obtained. Hence, to predict realistic spectra, empirical line broadening needs to be applied.

#### 10.6.2 Time-Correlation Function Approach

Apart from employing the static-harmonic approximation, there exists the possibility to compute vibrational spectra directly from MD simulations. In this approach, the spectra are obtained as the Fourier transform of some TCFs along the simulation trajectory, an idea that is at least 60 years old now,^422–424^ and is called the TCF formalism. This approach comes with several advantages over the static-harmonic concept:

- Condensed phase systems can be handled; it is possible to explicitly capture the effects of solvent and entropy on the spectrum.
- Some anharmonic effects, such as line broadening, approximate overtones, and combination bands, are reproduced.
- Realistic band shapes are obtained instead of a discrete line spectrum.
- Intrinsic conformer sampling takes place during the MD simulation.
- No minimum energy structure is required to compute the spectrum.

As CP2K has its main strengths in the description of condensed phase systems, including AIMD, it is particularly well suited to compute vibrational spectra via the TCF formalism. Therefore, the remaining part of this subsection will focus exclusively on this approach. The basic workflow is simple: perform an AIMD simulation, compute some electromagnetic properties along the trajectory, and use some postprocessing software to obtain a spectrum from that time series.

#### 10.6.3 Computing Electromagnetic Moments

Computing vibrational spectra, both via the static-harmonic approach and via the TCF approach, requires knowledge of certain electromagnetic moments of the system, e.g. the electric dipole moment. In the former case, these moments are calculated for excursions of the minimum structure along the normal modes, but they are computed for snapshots along an MD simulation trajectory in the latter case. For nonperiodic systems, electric moments can be readily derived as expectation values from electron structure calculations by applying the corresponding moment operator to the converged WF.

Under PBCs, however, the standard moment operators are ill-defined, as they often rely on the position operator. The challenge can be easily seen by, e.g. considering the total dipole moment of a simple chain of anions and cations in a periodic cell: the dipole moment depends on the cell origin, but it should not.^425^ Resta and King-Smith and Vanderbilt proposed a possible solution, the so-called modern theory of polarization,^426–429^ which is based on a Berry phase.^430,431^ In short, the ill-defined position operator *X̂* for the WF Ψ_0_ under PBCs is replaced by the following expectation value for the position^429^

155 $$\left\langle X\right\rangle =\frac{L}{2\pi }Im\;\ln\left\langle \Psi_{0}\left|\exp\left(i\frac{2\pi }{L}\mathrm{X̂}\right)\right|\Psi_{0}\right\rangle$$

Due to the ambiguity of the complex logarithm function, there exist infinitely many valid results from this equation, all equidistantly separated. This means that the dipole moment is also no longer uniquely defined under PBCs, but only up to a modulus. However, for most applications, this approach works very well.

In CP2K/Quickstep, the total electric dipole moment from the modern theory of polarization can be computed and printed by adding the following section to the input:
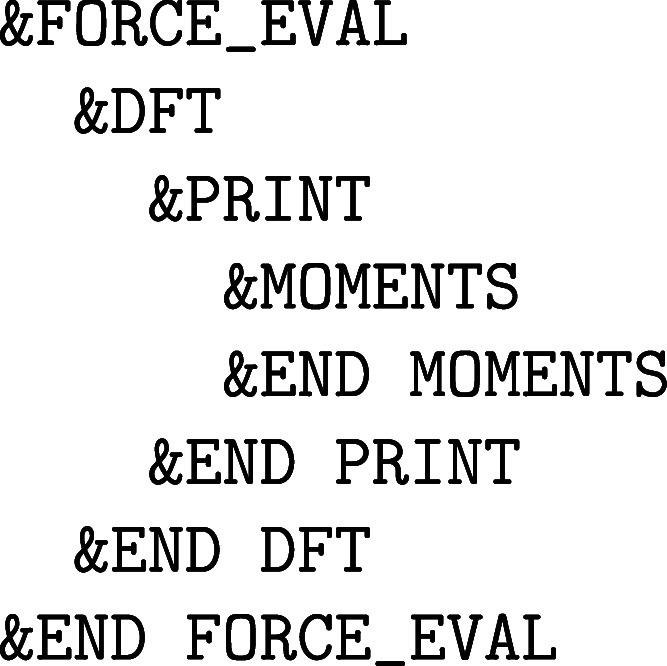


Note that despite the general name of the &MOMENTS subsection and the availability of a MAX_MOMENT keyword, it is implemented only for electric dipole moments in the periodic case.

#### 10.6.4 Total vs Molecular Moments

There are several reasons for considering molecular moments instead of system-wide “total” electromagnetic moments. First, it allows for separation of the spectral contributions of the constituents of a mixture, so that, e.g., the solvent spectrum can be suppressed. Second, the sampling of the spectrum is improved and the spectrum contains less noise when the TCF approach is used, as will be explained on the example of the dipole moment in the following. When assuming that the sum of the molecular dipole moments equals the total dipole moment, which is an approximation in the periodic case although the deviation is small,^432^ it can be shown that using the total dipole moment is mathematically equivalent to using all autocorrelations of the individual molecules’ dipole moments together with all cross-correlations between different molecules. It is often argued that cross-correlations between molecules that are not neighbors in space only introduce noise to the spectrum, as the motions of such molecules are not at all correlated. Therefore, it is often a good idea to consider only molecular autocorrelation functions and to neglect all cross-correlation terms between different molecules.

#### 10.6.5 Orbital Localization

One widely used approach to assign electric dipole moments to individual molecules is the localization of MOs in space.^433,434^ For nonperiodic systems, there exist some well-known and computationally efficient methods such as the Boys,^435^ as well as the Pipek–Mezey localization schemes,^436^ among others.

In periodic systems, orbital localization is considerably more involved. One commonly used method is the so-called Wannier localization.^437–442^ It applies a unitary transformation **U** to the set of occupied KS orbitals |ψ_*i*_⟩ so that another set of MOs $|{\mathrm{\psi ̃}}_{n}\rangle$ is obtained, which are called Wannier orbitals or MLWFs^443^

156 $$|{\mathrm{\psi ̃}}_{n}\rangle =\sum_{i}U_{i,n}|\psi_{i}\rangle$$

The unitary transformation **U** is constructed in a way so that the so-called spread functional

157 $$\Omega =\sum_{n}\;\sum_{I}f\left({\left|z_{I,n}\right|}^{2}\right)$$

158 $$z_{I,n}=\left\langle \psi_{n}\left|O^{I}\right|\psi_{n}\right\rangle$$

is minimized.^443^ Here, **O** ^*I*^ is a class of suitable spread operators that are well-defined in periodic space, such as

159 $$O^{I}=\exp\left(iG_{I}\cdot r\right)$$

where **G**_*I*_ are the reciprocal lattice vectors of *I* = *x*, *y*, *z* and *f* an appropriate function. Common choices for *f* are^438,440,443,444^

160 $$f_{1}\left({\left|z_{I,n}\right|}^{2}\right)=\sqrt{{\left|z_{I,n}\right|}^{2}}=\left|z_{I,n}\right|$$

161 $$f_{2}\left({\left|z_{I,n}\right|}^{2}\right)=\log\left({\left|z_{I,n}\right|}^{2}\right)$$

162 $$f_{3}\left({\left|z_{I,n}\right|}^{2}\right)={\left|z_{I,n}\right|}^{2}$$

Note that both the Boys-Foster localization^435^ and the Pipek–Mezey localization^436^ for nonperiodic systems can be expressed in terms of the above equations with the choice of *f* ≡ *f*_3_. For Boys-Foster, the operator **O** ^*I*^ is simply defined as **O** ^*I*^ = **r**_*I*_ with the conventional position operator **r**_*I*_.

One traditionally applied approach in quantum chemistry for localizing MOs is the method of two-by-two orbital rotations first introduced by Edmiston and Ruedenberg.^445^ Unfortunately, the analytical expression for the optimal angle of these rotations can only be derived for the choice of *f* ≡ *f*_3_,^443^ i.e. for the Silvestrelli-Marzari-Vanderbilt,^438–440^ the Boys-Foster,^435^ and the Pipek–Mezey functional.^436^ For the choice of *f* ≡ *f*_1_, which is commonly used for performing Wannier localization, one has to resort to iterative numerical methods such as a generalized Jacobi rotation scheme,^446^ or CP2K’s own so-called “crazy angle” algorithm. Yet, all of these methods require considerable amounts of extra computer time for the localization and are not guaranteed to converge. A discussion of these limitations can be found in the next subsection. A practical alternative to bypass the computational effort to localize the MOs in every AIMD time step is to propagate the matrix **U** of eq 156 by means of a second-generation Car–Parrinello-like “Wannier dynamics”.^11,447^ In CP2K this is enabled by setting &LOCALIZE%USE_HISTORY to TRUE.^448,449^

The centroids of the Wannier orbitals are called Wannier centers, or maximally localized Wannier centers (MLWCs); they can be seen as the positions of electron pairs in a simple picture. As those are located relatively closely to the atoms, it is well possible to assign Wannier centers to individual molecules. Based on these Wannier centers, the molecular dipole moment **μ** ^Mol^ can be expressed as

163 $$\mu^{Mol}=-2e\sum_{i=1}^{N}r_{i}+e\sum_{j=1}^{M}Z_{j}R_{j}$$

where *N* is the number of Wannier centers in the molecule, **r**_*i*_ is the position of the *i*-th Wannier center, *M* is the number of atoms in the molecule, whereas **r**_*j*_ and *Z*_*j*_ are the position and the nuclear charge of the *j*-th atom, respectively, and *e* is the elementary charge.^432^ The sum of all molecular dipole moments computed by this protocol is often a good approximation to the total dipole moment of the system.

In CP2K/Quickstep, the Wannier localization of the MOs can be requested by adding the following section:
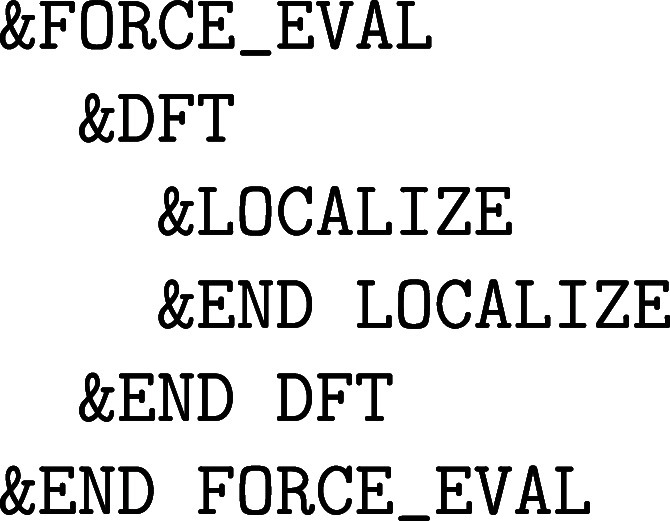


The following keywords inside the &LOCALIZE subsection are most relevant for practical use:

- METHOD: how to minimize the spread functional. Default is JACOBI (slow, but very robust), but should be set to CRAZY (a lot faster, but less robust) in most cases. See next keyword.
- JACOBI_FALLBACK: do a JACOBI minimization (“fallback”), if CRAZY did not converge.
- MAX_ITER: maximum number of iterations for minimizing the spread functional. Default is 10 000, which is almost always enough. Maybe reduce it to not waste time if it is not going to converge anyway.

Several types of results can be obtained from the localization procedure. This can be defined via subsections inside the &LOCALIZE%PRINT section. The following subsections are often useful:

- &WANNIER_CENTERS: outputs the “Wannier centers”, i.e. the centroids of the MLWFs to a XYZ file with the specified name. Also works in AIMD runs, where it just writes a consecutive Wannier center trajectory. If the IONS+CENTERS keyword is set, the atom coordinates are added to that file, which can make analysis easier.
- &WANNIER_SPREADS: outputs the remaining spread of the Wannier orbitals. This can be useful to, e.g. approximate polarizabilities,^450–452^ as shown below.
- &WANNIER_CUBES: outputs the actual Wannier orbitals on a Cartesian grid as cube files. Useful for visualization.
- &WANNIER_STATES: outputs the Wannier orbitals in the basis of the AOs, i.e. the corresponding coefficient matrix.

#### 10.6.6 Voronoi Integration

The Wannier localization comes with a few well-understood shortcomings:

- the CPU time required for localization can be substantial and scales unfavorably with system size. For a 1000 atom system, the localization typically takes almost as long as the energy and force calculation, i.e. slowing down the simulation by approximately a factor of 2.
- All known methods for computing the Wannier localization are iterative and are not guaranteed to converge. It happens in practice that for certain AIMD timesteps, neither the crazy angle algorithm, nor the Jacobi fallback converge, so that the electric moments are missing for these timesteps.
- Only molecular electric dipoles can be derived from the Wannier centers; higher-order momenta such as quadrupoles, as for instance required for Raman optical activity (ROA) spectra, are not directly accessible.
- Wannier localization enforces integer molecular charges. Any charge transfer effects between molecules cannot be captured, which leads to artificially increased dipole moments in some cases.
- Systems with delocalized electrons, such as aromatic molecules or metals, may entail convergence issues. Also, for example, the simulated IR spectrum of liquid benzene contains artificial bands that are a consequence from the Wannier localization.^453^

To avoid the computationally demanding localization procedure, one simple idea is to work with the total electron density on a Cartesian grid instead, which is always present in any PW-based electronic structure code including the GPW/GAPW method of CP2K/Quickstep, and to partition it with respect to the atom positions to obtain molecular electromagnetic moments. Even though there exist many such approaches, we will discuss here the so-called Voronoi integration.^453,454^

The Voronoi tessellation is a mathematical tool which partitions an Euclidean space containing some points (Voronoi sites) into nonoverlapping subsets.^455,456^ Each Voronoi site corresponds to exactly one such subset, known as a Voronoi cell, which contains all spatial points that are closer to this Voronoi site than to any other Voronoi site. In mathematical form, this is written as

164 $$C_{i}:=\{x\in R^{n}|∥x-p_{i}∥\leq ∥x-p_{j}∥\forall j\in \left\{1...k\right\},\;j\neq i\},,i\in \left\{1...k\right\}$$

where $R^{n}$ stands for any Euclidean space with the norm ∥ ∥, in which *k* Voronoi sites, each with position $p_{i}\in R^{n}$ , are given, and the $C_{i}\subseteq R^{n}$ are the resulting Voronoi cells.

By considering atoms in three-dimensional space as Voronoi sites, this concept has widely been applied in different fields of computational chemistry. To name a few advantages of the method, the Voronoi tessellation of a set of atoms is uniquely defined and can be calculated with moderate computational efforts. The Voronoi tessellation can easily be adapted to systems with PBCs, and is therefore well suited for bulk phase systems. Finally, the method does not possess any empirical parameters to tune, and therefore gives a uniquely defined picture.

Voronoi tessellation has already been used since long to partition the total electron density, by placing a simple plane midway between two atoms.^457,458^ However, certain limitations do arise from the properties of the standard Voronoi tessellation. As all atoms are treated in the same way, Voronoi polyhedra of light atoms like hydrogen will, on average, have the same size as those around heavier atoms like iodine. From a mathematical point of view, this is not a problem, but from a chemical perspective, this is completely unreasonable. If, for instance, the electron density within the Voronoi cell of a hydrogen atom is integrated, the hydrogen atom would always end up with a heavily negative partial charge, because way too much electron density would be considered as belonging to this hydrogen atom.

To overcome this problem, radii need to be introduced into the Voronoi tessellation, allowing to treat different atom types differently. Several ways to do so have been proposed. Many such approaches have been proposed in the literature. Here, we employ the so-called “radical Voronoi tessellation”, which, in the two-dimensional case, is also known as power diagram.^459^ Note that the term “radical” is not related to chemical radicals. In this technique, a radius is assigned to each atom, allowing to model the sizes of the atoms in a chemically reasonable sense. In contrast to other similar approaches,^459,460^ the radical Voronoi tessellation does not suffer from the “vertex error”, i.e. it does not contain holes. When integrating electron density, this is important to keep the total charge of the system constant. As another advantage, the Voronoi sites, around which the cells are constructed, can be kept on the atoms and do not have to be shifted to obtain a chemically reasonable partitioning.

The definition of the radical Voronoi tessellation as a generalization of the classical tessellation reads as

165 $$C_{i}^{r}:=\{x\in R^{n}|{∥x-p_{i}∥}^{2}-r_{i}^{2}\leq {∥x-p_{j}∥}^{2}-r_{j}^{2}\forall j\in \left\{1...k\right\},j\neq i\},,i\in \left\{1...k\right\}$$

with radius *r*_*i*_ for Voronoi site *i*. While in the classical case the face between two adjacent Voronoi cells is always placed in the middle between the corresponding Voronoi sites, its position is now determined by the difference of the squared radii. The definition of the radical Voronoi tessellation in eq 165 shows that the tessellation will not change if the set of radii {*r*_*i*_} is transformed to a new set {*r*_*i*_^′^} by the map

166 $$r_{i}^{\prime }:=\sqrt{r_{i}^{2}+C},,i\in \left\{1...k\right\}$$

with some constant C∈R . Due to this relation, the absolute value of the radii does not have a direct meaning.

The crucial parameters in the radical Voronoi tessellation are the radii assigned to the atoms. It was recently shown that vdW radii yield a reasonable separation of molecules in the bulk phase,^453^ and that the resulting molecular electromagnetic “Voronoi” moments can readily be used to calculate vibrational spectra of bulk phase systems from AIMD simulations.^461–463^

As soon as the Voronoi tessellation has been computed, the molecular electromagnetic moments can be obtained via simple integration of the total electron density ρ within each molecule’s Voronoi cell, e.g. for the electric dipole and quadrupole moment

167a $$\mu^{Mol}=\sum_{i=1}^{N_{Mol}}q_{i}r_{i}-\int_{Mol}ds\rho \left(s\right)s$$

167b $$Q_{jk}^{Mol}=\sum_{i=1}^{N_{Mol}}q_{i}\left(3r_{i,j}r_{i,k}-{∥r_{i}∥}^{2}\delta_{jk}\right)-\int_{Mol}ds\rho \left(s\right)\left(3s_{j}s_{k}-{∥s∥}^{2}\delta_{jk}\right)$$

where **r**_*i*_ and *q*_*i*_ are the position and core charge of atom *i*, respectively.

The Voronoi integration in CP2K is provided by the libvori library,^464^ which internally relies on Voro++.^465^ It can be switched on by adding the following section to the input:
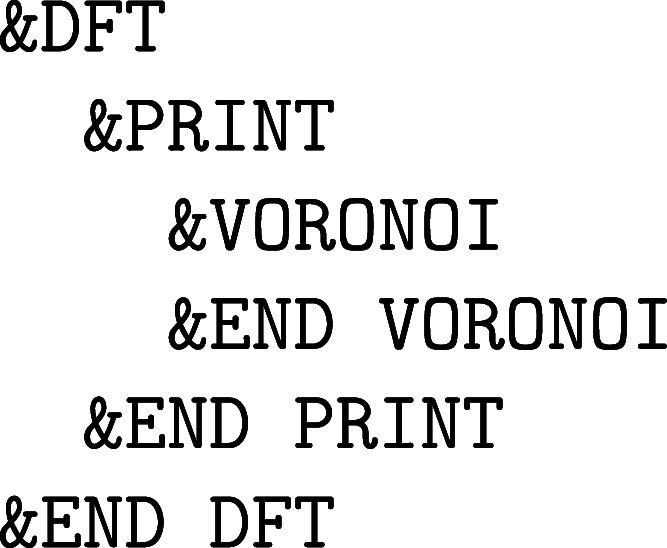


The following keywords inside of the &VORONOI section are most relevant for practical use:

- VORONOI_RADII: chooses the radii for the radical Voronoi tessellation. Can be set to UNITY (all radii are 1, i.e. classical Voronoi), VdW (vdW radii are used), COVALENT (covalent radii are employed),^466^ or USER. Default is VdW.
- USER_RADII: in case of VORONOI_RADII USER, specifies the radii (one number per atom in the system).
- OUTPUT_TEXT: outputs a .voronoi text file with all properties in a human-readable format. The file name can be set via FILENAME. On by default.
- OUTPUT_EMP: outputs a binary .emp file with all electromagnetic properties. Can be read by TRAVIS^418,467^ to compute vibrational spectra. Off by default.
- JITTER: randomly displaces all Voronoi sites a tiny bit to avoid problems with highly symmetric structures. The amount can be controlled by the keyword JITTER_AMPLITUDE. On by default.

The resulting quantities obtained from the Voronoi integration are per atom. For each atom, the Voronoi charge, the center-of-charge vector, the electric dipole vector, and the trace-free electric quadrupole tensor are printed. If molecular properties are required, they can easily be combined as follows by looping over all atoms of the molecule

168a $$q^{Mol}=\sum_{i=1}^{N_{Mol}}q^{i}$$

168b $$\mu^{Mol}=\sum_{i=1}^{N_{Mol}}\mu^{i}+q^{i}\left(r^{i}-r^{Ref}\right)$$

168c $$Q_{jk}^{Mol}=\sum_{i=1}^{N_{Mol}}Q_{jk}^{i}+\mu_{j}^{i}\left(r_{k}^{i}-r_{k}^{Ref}\right)+\mu_{k}^{i}\left(r_{j}^{i}-r_{j}^{Ref}\right)+q^{i}\left(r_{j}^{i}-r_{j}^{Ref}\right)\left(r_{k}^{i}-r_{k}^{Ref}\right),$$

where **r** ^*i*^, *q* ^*i*^, **μ** ^*i*^, and **Q** ^*i*^ are the position, Voronoi charge, electric dipole moment, and electric quadrupole tensor of atom *i*, respectively. The choice of the coordinate origin **r** ^ref^ leaves the molecular dipole moment invariant as long as the molecular Voronoi charge is zero, and leaves the molecular quadrupole moment invariant as long as the molecular dipole moment is zero. Otherwise, these quantities depend on the choice of a reference point.

#### 10.6.7 Polarizabilities

After discussing the electric dipole moment, we cover a second type of important electromagnetic property, namely the electric polarizability, or more correctly, the static electric dipole-electric dipole polarizability. In contrast to the dipole moment, it is a response property and therefore can not be directly obtained from the WF of the system.

One straightforward approach to electric polarizabilities is to use finite differences with respect to an external electric field.^173^ In linear approximation, the dipole moment **μ**_ind_ induced by an electric field **E** can be expressed as

169 $$\mu_{ind}=\alpha E$$

with the second-order electric polarizability tensor **α**. This leads to the central finite differences

170 $$\alpha_{i,j}=\frac{\mu_{i}^{j+}-\mu_{i}^{j-}}{\left|E^{j+}-E^{j-}\right|},,i,j=x,y,z$$

where **E** ^*j*+^ and **E** ^*j*–^ are the field vectors of the external electric field applied in the positive and negative *j* direction, respectively. Moreover, μ_*i*_^*j*+^ and μ_*i*_^*j*–^ are the *i* components of the dipole moment under the influence of these two fields, and α_*i*,*j*_ is the (*i*, *j*) component of the polarizability tensor. By performing six additional SCF calculations with positive and negative fields in the *x*, *y*, and *z* direction, the full polarizability tensor can thus be obtained. The strength of the electric field |E| needs to be chosen so that the system is still within the linear regime of polarizability, i.e. eq 170 is still a good approximation. Reasonable choices for organic liquids were found to be $\left|E\right|\approx 5.0\cdot 10^{-4}$ a.u. = 2.57 × 10^8^ V m^–1^,^453^ or even up to 5.0 × 10^–3^ a.u. = 2.57 × 10^9^ V m^–1^.^461^ If one uses molecular electric dipole moments in these calculations, e.g. from Wannier centers or Voronoi integration, the molecular polarizability tensor for each molecule in the system can be obtained.

In CP2K/Quickstep, a periodic electric field, i.e. without any discontinuities and suitable for bulk phase systems, can be applied using the &PERIODIC_EFIELD section. See the following example for a field in the positive *x*-direction, as controlled by the POLARIZATION keyword:
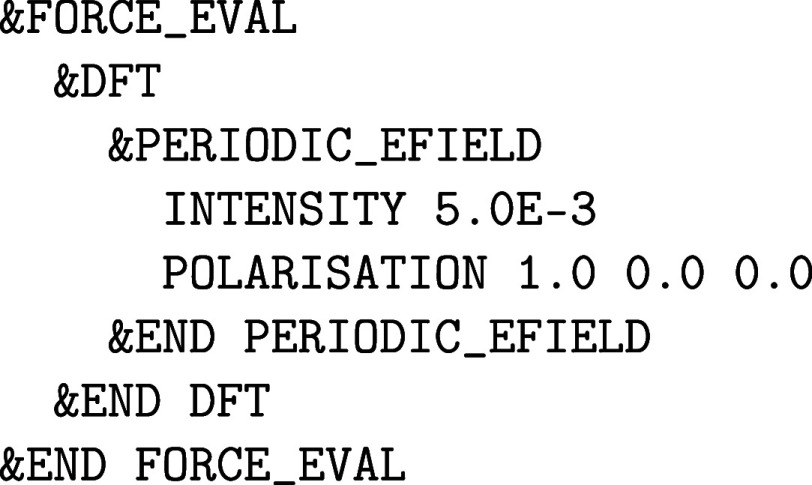


However, &PERIODIC_EFIELD requires certain derivatives of the MOs, which are only available via the OT method, so that electric polarizabilities from finite differences can currently not be obtained with diagonalization-based mixing schemes, or with electronic smearing.

When molecular polarizabilities are computed according to eq 171, the changes in the local electric field of a molecule by the polarization of the neighboring molecules are omitted. This effect can be captured by considering the dipole–dipole interaction tensor computed by Ewald summation under PBCs,^468^ as explained in refs 469,470 , respectively. However, a recent study of water has shown that this has only a minor influence on the resulting spectra.^471^

Another approach to electric polarizabilities is the previously described variational DFPT. Originally proposed and implemented in the CPMD code by Putrino and Sebastiani,^167,173^ it is also available in CP2K.^472^ It does not suffer from possible nonlinearities, such as in finite difference approaches, and is less prone to numerical noise, but it can be slow and numerically less stable, and it does not allow direct access to molecular polarizability tensors. Such a calculation can be requested by using the following input:
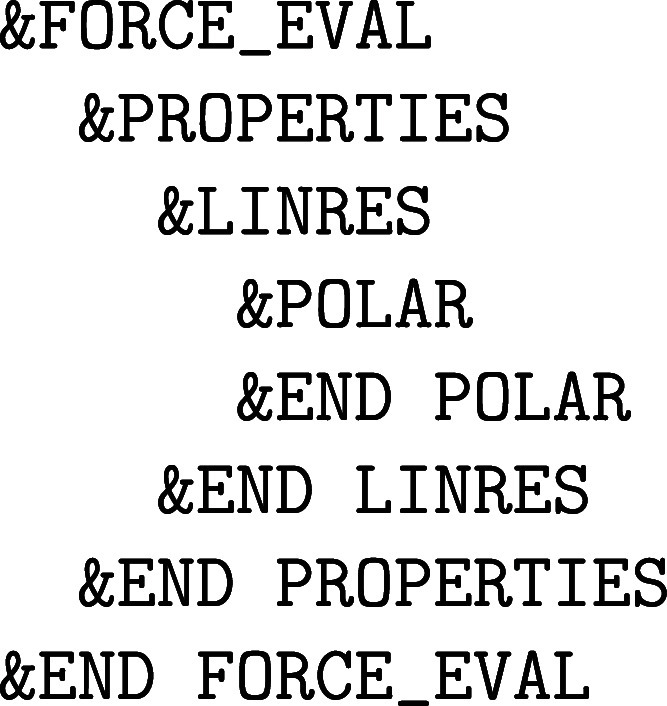


However, there exist further approaches to computing molecular polarizabilities, such as one by Partovi-Azar and Kühne to approximate the polarizability tensor based on the spatial spread of the Wannier centers.^450,451^

#### 10.6.8 Frequency-Dependent Polarizabilities

The electric polarizability is a frequency-dependent property. For many applications, this dependency is completely neglected, and the static (i.e., zero-frequency limit) polarizability is used, such as in predicting Raman spectra. But, such effects need to be explicitly considered when predicting the correct intensities in resonance Raman spectra, for instance. In such cases, one needs to compute the dynamic (i.e., frequency-dependent) polarizability. Doing so turned out to be quite intricate. As the dynamic polarizability involves electronic excitations, it is generally not sufficient to solve the time-independent Schrödinger equation to obtain it. Several approaches to compute this property have been presented in the literature. Many of them are based on the vibronic theory of Albrecht and co-workers,^473–475^ or on the time-dependent formalism of Heller and co-workers.^476–478^ Another method, based on LR-TDDFT, was published by Jensen and Schatz.^479,480^

Recently, a different approach has appeared in the literature that uses RT-TDDFT^481–483^ to obtain the dynamic polarizability of the sample.^484^ In contrast to the methods mentioned above, the real-time approach offers the advantage of including all electronic excitations into the calculation, so that the full frequency range is covered and no subset of low-lying excitations needs to be selected. Furthermore, this approach intrinsically includes nonlinear effects that are neglected in perturbative methods such as LR-TDDFT. And last but not least, it allows access to molecular polarizability tensors in bulk phase systems, which is otherwise not possible.

Based on this approach, a protocol for the calculation of molecular dynamic polarizability tensors in condensed phase systems using CP2K has been developed and published,^462^ which can be used in the TRAVIS program package.^418,467^ The protocol automatically creates all required CP2K input files and works as follows. The initial WF is optimized under the influence of an external periodic electric field, which is switched off in the beginning of the RT-TDDFT run, so that the electron density starts to fluctuate (step response). During the RT-TDDFT run, the temporal development of the total electron density is processed with the Voronoi integration scheme to yield time series of molecular electric dipole vectors **μ**(*τ*) as a function of RT-TDDFT time τ. The Fourier transform of the three dipole vector components yields three entries of the molecular dynamic polarizability tensor. To obtain the full tensor α_*ij*_(ω) for each molecule (with ω the incident laser frequency), three RT-TDDFT runs are performed from initial WFs optimized under external fields in the *x*, *y* and *z* directions

171 αij(ω)=1|E|∫0Tdτ⁡(μi(τ)−μi0,j)×exp((−cτT)2)exp(−iωτ)

Please note the use of a Gaussian window function with parameter *c* for the Fourier transform of the RT-TDDFT time series. Furthermore, *T* is the total RT-TDDFT simulation time, **μ** ^0,*j*^ denotes the initial molecular dipole moment after WF optimization under an external electric field in *j* direction, and |**E**| is the absolute value of the external electric field. Please also note that the dynamic polarizability tensor obtained from eq 172 is complex-valued, with dispersion as the real part and absorption as the imaginary part. Speaking of polarizabilities typically refers to the dispersion, i.e. the real part.

#### 10.6.9 Supported Types of Spectra

Computing time series of electromagnetic properties (dipole moment, polarizabilities, etc.) by means of AIMD permits obtaining vibrational spectra by a postprocessing tool. The basic idea is simple: calculate the required cross-correlation functions of the electromagnetic properties along the simulation trajectory, and then Fourier transform these correlation functions to yield the spectrum. In practice, there are several more subtleties and tricks that should be considered to obtain high-quality spectra. Examples are signal processing techniques such as applying window functions and zero padding, but also exploitation of time-reversal symmetry to enhance sampling or finite-difference correction. Using the TRAVIS postprocessing tool,^418,467^ IR, Raman, vibrational circular dichroism (VCD), Raman optical activity (ROA) and resonance Raman spectra can be easily computed for periodic condensed phase systems.

##### 10.6.9.1 Storing Compressed Density Cubes

Many of the electromagnetic properties described above can be obtained from the total electron density of a periodic system via Voronoi integration, which can be performed on-the-fly during the CP2K AIMD run. If for some reason the electron density data shall be kept along the trajectory, huge trajectories of Gaussian cube files, which are in the order of many terabytes, would result.^485^ For that purpose, CP2K offers to write these volumetric electron density trajectories in bqb file format,^486^ which utilizes a lossless compression algorithm that reaches a compression ratio of up to 40:1 by exploiting both the spatial and temporal smoothness of the electron density time series. To switch on this feature, use an input similar to the following example:
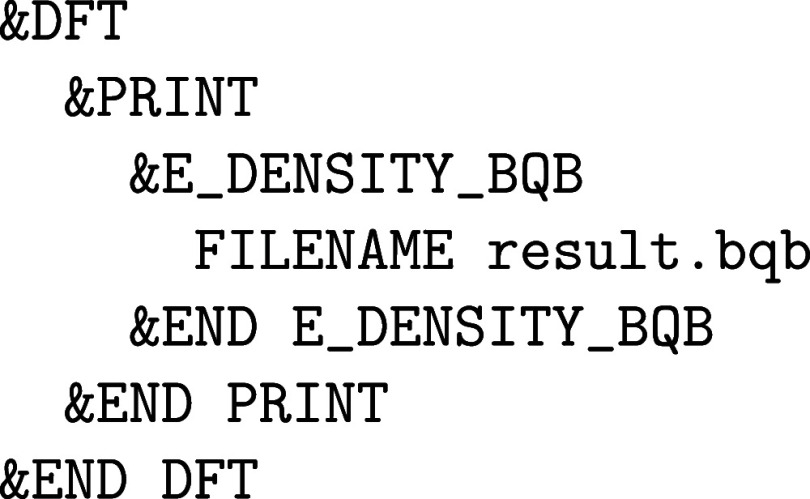


Apart from trajectories, single cube files can also be compressed by this approach, albeit with a slightly lower compression ratio. The resulting .bqb files can be read, processed, and unpacked by the TRAVIS program package.^418,467^

##### 10.6.9.2 Normal Mode Analysis from Ab Initio Molecular Dynamics

When computing vibrational spectra in the static-harmonic approximation via RUN_TYPE VIBRATIONAL_ANALYSIS as shown above, each spectral band is obtained together with a corresponding normal mode so that the spectral features can be easily assigned to specific molecular vibrations. However, when computing spectra from AIMD simulations via the TCF formalism, this cannot so easily be achieved because the spectrum is obtained as a superposition of all vibrational modes in the system without additional information on specific molecular motions that contribute to a certain spectral feature.

To overcome this limitation, several approaches for the extraction of normal modes from MD simulations have been reported in the literature.^487,488^ These approaches include the instantaneous normal-mode analysis (INMA), where the Hessian of the system is calculated in certain timesteps along the trajectory.^489–491^ Moreover, it includes the principal mode analysis (PMA), where an eigenvalue problem, derived from cross-correlation functions of particle positions and velocities, is solved.^490,492,493^ Subsequently, the generalized normal coordinate scheme of Mathias et al. has been developed.^494,495^ It is similar to the PMA approach, but does not require the equipartition theorem to be fulfilled. This can be computed using TRAVIS^418,467^ and a short summary of the approach will be given below.

The concept of normal modes generally relies on the assumption that the molecule undertakes small oscillations around a fixed reference structure, which is usually a minimum on the potential energy surface. However, in a MD trajectory, there will also be translational and rotational motion, which needs to be removed prior to the normal coordinate analysis. For this purpose, the trajectories are transformed into the Eckart frame of ref 496 by finding a rotation matrix and a translation vector in each time step such that the mass-weighted root-mean-square distance to a reference structure is minimized. The assumption of small oscillations around one single reference structure breaks down if there are conformational changes within the trajectory. In this context, the procedure needs to be extended to several reference structures.^495^ Either these can differ only in the ordering of equivalent atoms (e.g., if a methyl group rotates, where the hydrogen atoms are indistinguishable), or they belong to structurally different minima on the potential energy surface (e.g., if a butyl group changes between trans and gauche conformation). In the former case, all conformations can be mapped to a single minimum by considering the permutations of equivalent atoms, whereas in the latter case, the normal coordinate analysis results in independent normal modes for all minima. If there exists more than one reference structure, the probability of a molecule corresponding to a reference structure has to be defined for all snapshots, all molecules, and all reference structures.

For each reference structure, a “matrix” of all atomic velocity cross-correlation functions (i.e., each matrix element is a function) is computed. This matrix shall be diagonalized. As the matrix elements are not numbers, standard diagonalization techniques cannot be applied. Still, it is possible to minimize the integral over the off-diagonal functions by a modified Jacobi algorithm,^446,494^ leading to an orthogonal transformation matrix that minimizes the cross-correlation spectra. This transformation matrix consists of the set of new coordinate vectors that characterize the normal modes. Since the trace of a matrix is not changed by an orthogonal transformation, the total power spectrum of the system is not modified by this procedure, but the modes are localized in frequency space. More details can be found in ref 497.

## 11 Technical Aspects

CP2K is built via CMake and requires a modern Fortran and C compiler; several are known to work, among others, the GNU, Intel, and Cray compilers. A list of supported compiler versions is maintained on the CP2K wiki.[Fn fn1] Furthermore, many configurations are continuously tested on the CP2K dashboard.^498^

### 11.1 Installation

#### 11.1.1 Prerequisites and Dependencies

CP2K uses several external libraries, listed in Table 2. The table also indicates which libraries can make use of GPU acceleration via CUDA, HIP, or OpenCL. In this case, the corresponding GPU computing libraries have to be present, and the provided MPI distribution needs to support them. Table 2 also lists the purposes of external libraries. Most of the libraries are optional and are only required for performance improvements, or to enable specific functionalities. The second column in Table 2 categorizes the dependencies as internal, required, and optional. For MPI parallelization, an MPI implementation and ScaLAPACK are additionally required.

**Table 2:** List of Internal (Int.), Required (Req.), Optional (Opt.), and Required for Parallel Version (Par.) CP2K Dependencies^a^

library	req ?	GPU	purposes
BLAS/LAPACK	req		general
DBCSR^90^	req	C,H,O	sparse matrix
DBM	int.	C, H	sparse matrix
grid	int.	C, H	integration
MPI	par.		general
ScaLAPACK	par.		general
FFTW3 ^499^	opt.		FFT
FPGA^500–503^	opt.	O	PW-FFT
COSMA^103^	opt.	C, H	matrix multipl.
SPLA^91^	opt.	C, H	matrix multipl.
LIBXSMM^504^	opt.		DBCSR, DBM
LIBINT^105^	opt.		HF exchange
LIBXC^94^	opt.		XC functionals
ELPA^505^	opt.	C	diagonalization
cuSOLVERMp^506^	opt.	C	diagonalization
DLA-Future^507^	opt.	C, H	diagonalization
SIRIUS^508^	opt.	C, H	separate PW code
libvori^464^	opt.		Voronoi integration
DFT-D4 ^509^	opt.		dispersion corr.
tblite^510^	opt.		GFN2-xTB
PW	int.	C, H	solvation models
libgrpp^511^	int.		ECPs
PLUMED^512^	opt.		sampling methods
spglib^513^	opt.		symmetry detection
LibTorch	opt.		ML library
DeePMD-kit^319^	opt.		ML potentials
ACE^318^	opt.		ML potentials
NequIP^320^	opt.		ML potentials
Allegro^321^	opt.		ML potentials
Smeagol^514^	opt.		NEGF transport
TREXIO^515^	opt.		IO formats
GreenX^516^	opt.		Green’s functions

a In order to use GPUs, either CUDA (C), HIP (H), or OpenCL (O) can be employed.

#### 11.1.2 Building CP2K

There are four suggested ways for the installation of CP2K: (1) via distribution packages, (2) via containers, (3) from the source with CMake, and (4) with the Spack package manager.

The quickest installation methods are via a Linux or macOS distribution,[Fn fn2] or through ready-made containers[Fn fn3] run with Apptainer, Podman, or Docker. These methods also include the ability to run CP2K with MPI parallelization and GPU acceleration. Both installation methods require no build steps or tuning, and containers also offer optimized versions for essential platforms.[Fn fn4] ^,^ [Fn fn5] Generally, a distribution package or container, if available, is the easiest way to install CP2K. However, beware that many packages include only a small subset of the optional dependencies.

Installation via CMake is recommended for users with a strong technical background.[Fn fn6] Using CMake, it is possible to fully customize the CP2K installation, enable any of the optional dependencies, and fine-tune the various compilation options.

The Spack package manager^517^ is recommended for advanced users, or HPC clusters to obtain a feature-complete installation of CP2K.[Fn fn7] Spack is especially suited for the installation and management of multiple versions of CP2K together with different versions of its dependencies. One can also use Spack to only install CP2K’s dependencies and then build CP2K itself manually via CMake.

Many HPC centers also provide CP2K as a software module. Such preinstalled binaries are generally preferable because they typically have been carefully tuned to ensure good performance on the specific hardware.

#### 11.1.3 Sanity Checks after Installation

For CMake and Spack installations, as well as MPI-parallel and GPU-enabled containers, it is highly recommended to perform some sanity checks before starting extensive calculations with CP2K.

It is recommended to check that (1) CP2K was installed correctly and produces correct results, (2) that MPI and OpenMP parallelization work, and (3) for GPU installations, that the GPU is actually used. The Python script do_regtest.py provided as part of the CP2K source code can be used to test all three aspects. The script can be invoked as follows: ./do_regtest.py /cp2k/path/bin/ psmp

In order to run the tests, it may be required to define the data directory (located in the CP2K directory) via export CP2K_DATA_DIR = /Path/to/data. For GPU builds, its usage should be checked with the help of the nvidia-smi (or similar) command-line tool during the execution of the tests.

The CP2K test suite consists of more than 4000 regtests. However, although these are all complete and syntactically correct CP2K input files, they should not be misunderstood as a source for meaningful settings, as the parameters are often chosen to minimize execution time of a specific functionality instead of reliability of the resulting data. For that purpose, the examples of the present work at https://github.com/cp2k/cp2k-examples are recommended instead. A detailed description of CP2K’s regression testing is available at https://www.cp2k.org/dev:regtesting. Depending on the build options, the relevant tests will be executed. For each test, an input file is run, and then parts of the output are compared against reference values. When a test crashes midway with a runtime failure, this clearly indicates a serious problem with the binary. It is more ambiguous when a test is complete, but the result does not agree with the reference value within the given threshold. In such cases, additional investigation is required to discern whether the threshold was simply a bit too tight, the test did not converge due to numerical instabilities, or there is an actual problem with the binary.

Persistent errors can be reported to CP2K developers using the GitHub issue tracker,[Fn fn8] the dedicated Google group,[Fn fn9] or GitHub discussions.[Fn fn10] These resources also contain other known problems and possible solutions.

Beside directly contributing your code, as the constraint DFT implementation of Holmberg and Laasonen for instance,^518,519^ or interfacing to CP2K (e.g., PLUMED,^520^ phonopy,^521^ Libra,^522^ PyRETIS,^523–525^ Wannier90,^526^ Green-X,^516^ Newton-X,^527^ DeepMD-kit,^528^ MiMiC^529^), it is possible to connect to CP2K via community interfaces (e.g., ASE,^530^ i-Pi,^531,532^ or Spicy^533^) or domain-specific file formats (e.g., MD-TRACKS,^534^ ZEOBUILDER,^535^ TAMkin,^536^ IOData,^537^ openPMD,^538,539^ TREXIO,^515^ Multiwfn^540^), as well as compiling CP2K as a library (e.g., ISA,^541,542^ PIMD,^543,544^ phonopy,^545^ OpenMolcas,^546^ or Qiskit Nature,^547^ MixPI^548^).

### 11.2 Performance Aspects

In addition to checking for functionality, the tests can also be used to get a first idea of performance. At the end of each CP2K run, the essential timings will be listed. For the provided test cases, they can be compared with the CP2K dashboard.^498^ Additionally, a folder with benchmarks is available in the distribution in the folder cp2k/benchmarks. The timings for different systems are listed at,^549^ which is periodically updated for new relevant hardware platforms. Both lists should be consulted to get realistic reference values for the expected execution times on the given hardware. A large discrepancy from the reference values suggests suboptimal builds, or a failure to actually use GPUs even though they are present.

A TIMING section like the following can be found at the end of each CP2K output file:
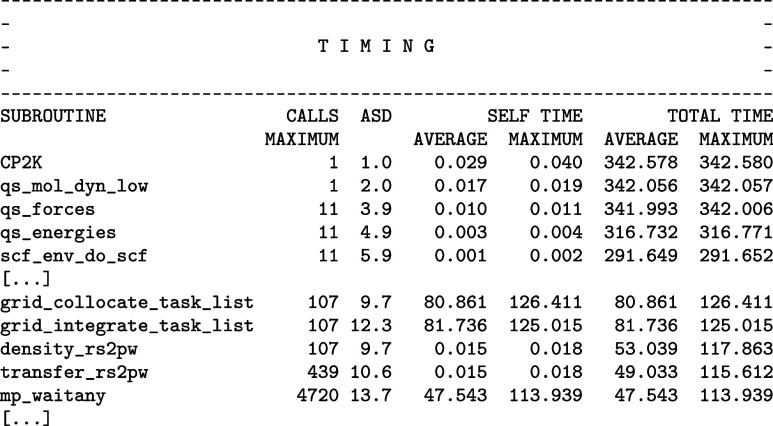


It lists different subroutines and in the second column the number of calls is given. The column ASD indicates the nesting level of the code. The timings are split into two groups: SELF TIME is the time the routine needs without nested subroutine calls inside, whereas TOTAL TIME includes them. For each group there are two columns: MAXIMUM gives the time of the MPI rank with the longest execution time, the AVERAGE value is the average over all ranks. A large difference between these two indicates load balancing issues. Routines and library calls that are computationally expensive can be identified from the SELF TIME column. In the example a significant amount is spent in the integration of the DFT grid (i.e., grid_* routines). The first part of the routine name relates the routine to different parts of the code. For example, “mp_” indicates that it is part of the MPI part, related to communication.

Depending on the type of computation, additional performance statistics appear earlier in the output file. In most calculations, the DBCSR library will be used, which reports its own statistics like:
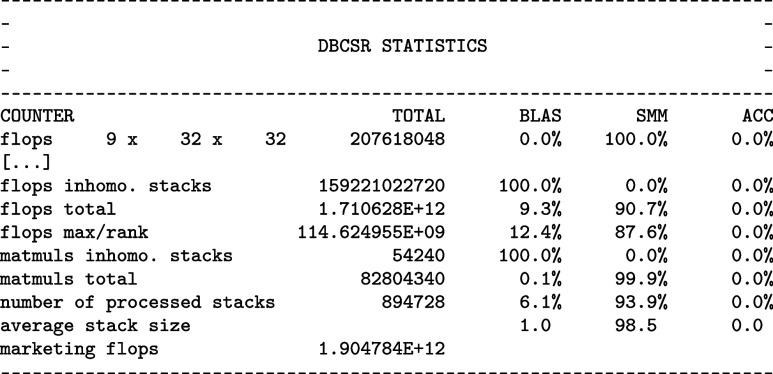


Besides the number of flops (floating point operations per second) listed in the column TOTAL the percentage of operations executed with different modules is listed. DBCSR can use different libraries for basic linear algebra operations, which is indicated in the last three columns. This can be different BLAS implementations like cuBLAS, the LIBXSMM^504^/libsmm libraries (SMM) or execution on GPUs (ACC).

The general recommendation for faster execution is to switch to GPUs when available and whenever most of the runtime is reported for libraries that have a GPU option. If there are considerable runtime shares that cannot be replaced by GPUs, then using them will still entail a gain in performance, yet in a limited and inefficient way.

If this is not advised, then the only general alternative is to scale up the number of MPI ranks for multiple compute nodes and OpenMP threads for CPU cores inside of the compute nodes. Often it is beneficial to balance this toward more MPI ranks and fewer OpenMP threads (for the same product of MPI ranks × OpenMP threads). There is no general optimum, and performance experiments of your own for the type of computations performed may be required to reach the sweet spot.

For a series of many independent calculations like in parameter studies or high-throughput computation projects,^55^ there is a general recommendation, however: reduce the parallelism of each individual run and execute more separate cases concurrently (trivial parallelization). Make sure that each run has its exclusive computing resources. This will bring the best parallel efficiency, at least up to the point where memory usage or other constraints will prevent it. In most cases, a sensible compromise between the extremes will be best for practical purposes.

## Data Availability

The data underlying this study are openly available at https://github.com/cp2k/cp2k-examples

## Footnotes

a
https://www.cp2k.org/dev:compiler_support.

b
https://manual.cp2k.org/trunk/getting-started/distributions.html.

c
https://github.com/cp2k/cp2k-containers.

d Docker hub https://hub.docker.com/r/cp2k/cp2k.

e NVIDIA NGC catalog https://catalog.ngc.nvidia.com/orgs/hpc/containers/cp2k.

f
https://manual.cp2k.org/trunk/getting-started/build-from-source.html.

g
https://manual.cp2k.org/trunk/getting-started/build-with-spack.html.

h CP2K issue tracker: https://github.com/cp2k/cp2k/issues.

i CP2K Google group: https://groups.google.com/g/cp2k.

j CP2K GitHub discussions: https://github.com/cp2k/cp2k/discussions.
